# Design, Synthesis, and Characterization of Stapled Oligosaccharides

**DOI:** 10.1021/jacs.2c06882

**Published:** 2022-09-29

**Authors:** Manuel G. Ricardo, Emelie E. Reuber, Ling Yao, José Danglad-Flores, Martina Delbianco, Peter H. Seeberger

**Affiliations:** † Department of Biomolecular Systems, Max-Planck-Institute of Colloids and Interfaces, Am Muehlenberg 1, 14476 Potsdam, Germany; ‡ Institute of Chemistry and Biochemistry, Freie Universität Berlin, Arnimallee 22, 14195 Berlin, Germany

## Abstract

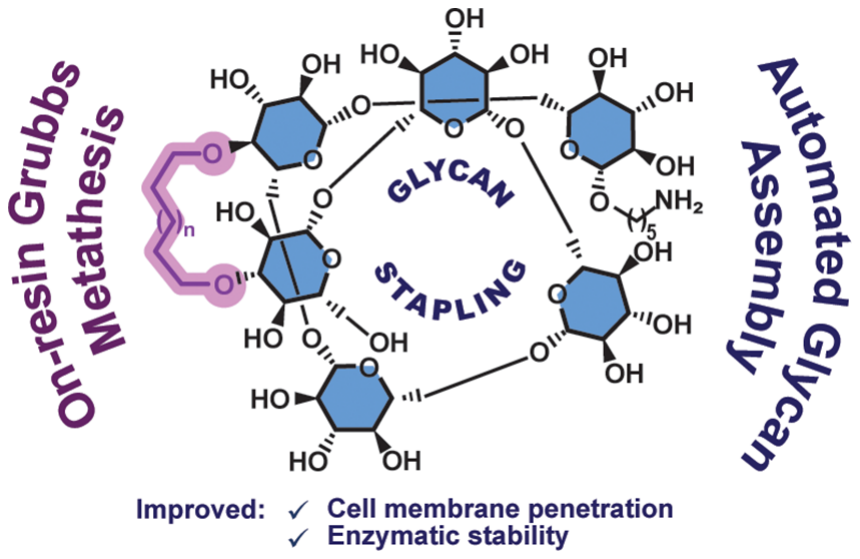

Stapling short peptides to lock specific conformations and thereby obtain superior pharmacological properties is well established. However, similar concepts have not been applied to oligosaccharides. Here, we describe the design, synthesis, and characterization of the first stapled oligosaccharides. Automated assembly of β-(1,6)-glucans equipped with two alkenyl side chains was followed by on-resin Grubbs metathesis for efficient ring closure with a variety of cross-linkers of different sizes. Oligosaccharide stapling increases enzymatic stability and cell penetration, therefore opening new opportunities for the use of glycans in medicinal chemistry.

## Introduction

The chemical synthesis of short fragments of proteins, nucleic acids, and polysaccharides, complex macromolecules that are at the heart of all biological processes, has been key to gain a better understanding of these biopolymers.^1,2^ However, small oligomers are more flexible, and their biological behavior may differ from the parent macromolecule.^3−5^ In addition, the use of peptides in pharmacological applications is severely hampered by their low metabolic stability and poor capacity to cross biological membranes.^6,7^ Rigidified synthetic oligomers exhibit improved biological parameters.^8−11^

Cyclization is a common strategy employed by nature to reduce conformational space and bestow different biomolecules with specific features.^12,13^ Synthetic chemists have used cyclization to endow synthetic peptides with superior pharmacological features, including receptor binding affinity, cell-membrane permeability, and metabolic stability.^14−16^ The so-called “stapling”, originally referred to the cyclization of two amino acid residues in a peptide chain by ring-closing metathesis (RCM), is a straightforward approach to prepare short helical peptides.^17,18^ Now, a variety of stapling techniques are available to generate synthetic cell-accessible miniproteins^19^ and peptide ligands that mimic protein–protein interactions.^20−22^ Similarly, stapling of oligonucleotide backbones enhances their stability and hybridization properties.^23^

Cyclic carbohydrates, such as cyclodextrins, are based on repetitive monosaccharides connected by glycosidic linkages.^24^ Cyclization methods that enable the generation of nonsugar cross-linkers have been utilized for the synthesis of natural products.^25,26^ However, the possibility to tune the 3D orientation of carbohydrates by cyclization remains mostly unexplored, with applications only in the area of locally constrained small oligomers.^13,27^ Inspired by the work in the fields of peptides and oligonucleotides, we aimed to design, synthesize, and characterize stapled oligosaccharides in an effort to create carbohydrates with improved enzymatic stability and cell penetration properties.

## Results and Discussions

### Glycan Stapling Design

**Figure 1:**
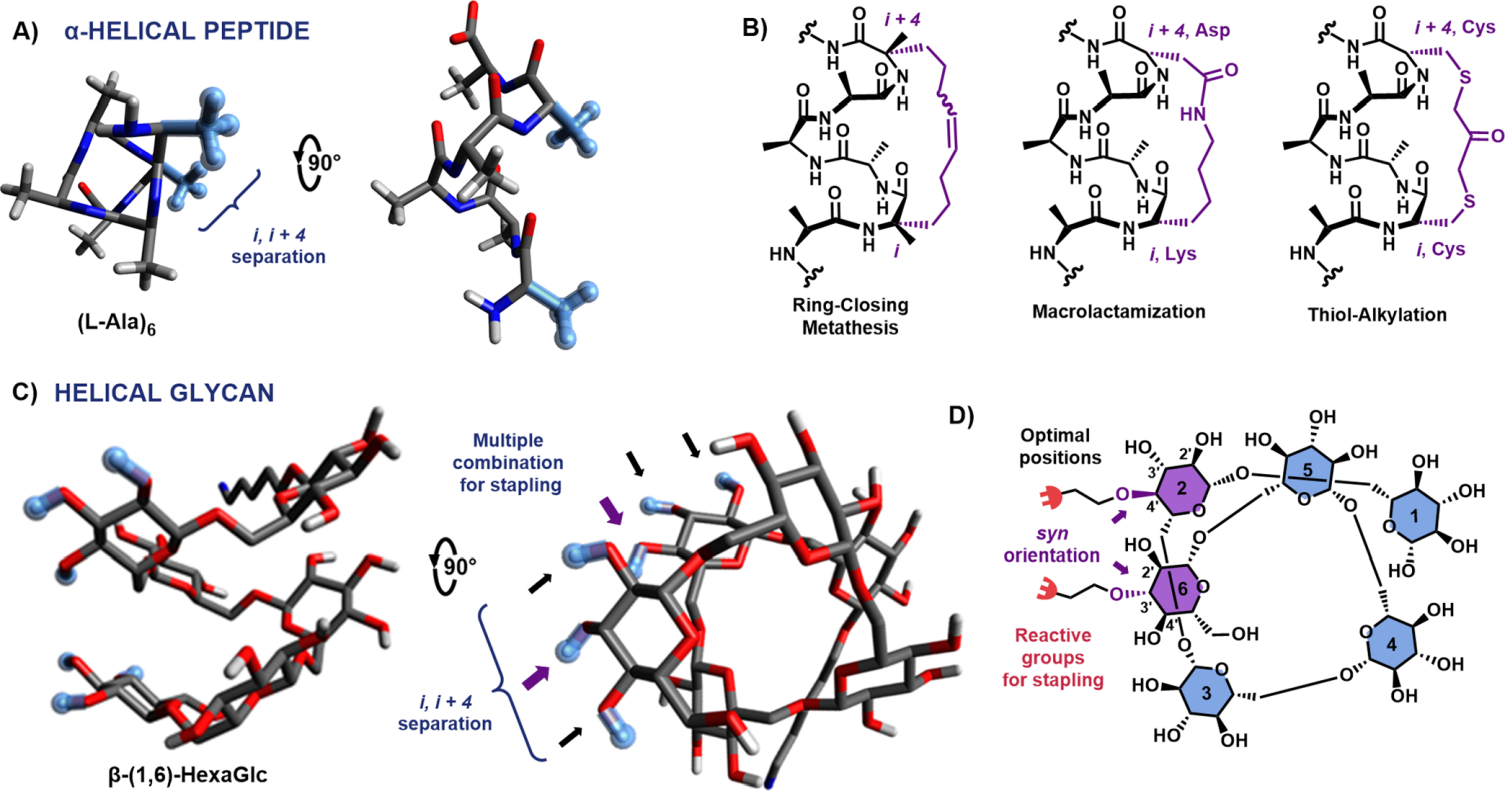
Peptide stapling vs glycan stapling: structural and chemical considerations. (A) α-Helical peptide model consisting of an l-Ala-hexamer. The amino acids commonly used for stapling are highlighted in light blue. (B) Examples of chemical strategies used for peptide stapling. (C) Helical structure of a β-(1–6)-glucose hexamer (minimal energy conformation^5^) highlighting the monosaccharides located at the same face and the multiple combinations available for stapling. (D) Schematic stereochemical representation of the optimal combination of residues proposed for glycan stapling of helical β-(1–6)-glucans.

Like peptides, glycans can adopt helical structures (Figure 1A,C). α-(1,4)-Glucose (amylose), β-(1,3)-glucose (curdlan), and β-(1,6)-glucans are naturally occurring helical polysaccharides (Figure 1C).^5,28^ The compact conformation, along with the expedient assembly of β-(1,6)-glucose linkages, makes this substrate an ideal candidate for the development of a stapling technique.

Stapling any oligomer requires careful positioning of two functional handles in proximity and properly oriented toward each other. For example, in α-helical peptides, two amino acid residues separated at *i*, *i* + 4 (as well as *i*, *i* + 7) along the sequence (Figure 1A,B) are in the same region and serve as a basis for cyclization chemistries, such as RCM,^17^ macrolactamization,^29^ and bis-thiol alkylation.^30^

Similar spatial rules for the functionalization of glycans did not exist. In addition, unlike peptides, glycans have a minimal variety of functional groups that can be utilized in chemoselective transformations. Thus, it is necessary to functionalize two of the hydroxyl groups with additional side chains bearing complementary functional groups (Figure 1D). In the energy-minimized structure of the example oligosaccharide (see Figure 1C,D),^5^ monosaccharide residues positioned at *i*, *i* + 4 (e.g., Glc2 and Glc6) are in close proximity. Still, each monosaccharide offers three possible sites for modifications (C2-OH, C3-OH, and C4-OH), with stereochemistry playing an essential role in the orientation. A thorough inspection of the 3D structure clearly suggests C3-OH on Glc6 and C4-OH on Glc2 as the best stereochemical combination (see Figure 1D). These functional groups (a) are in close proximity, (b) permit the beneficial syn orientation, and (c) do not affect the C2-OH position that needs to be temporarily protected as an ester to ensure the desired β-glycosylation during backbone assembly.

**Figure 2:**
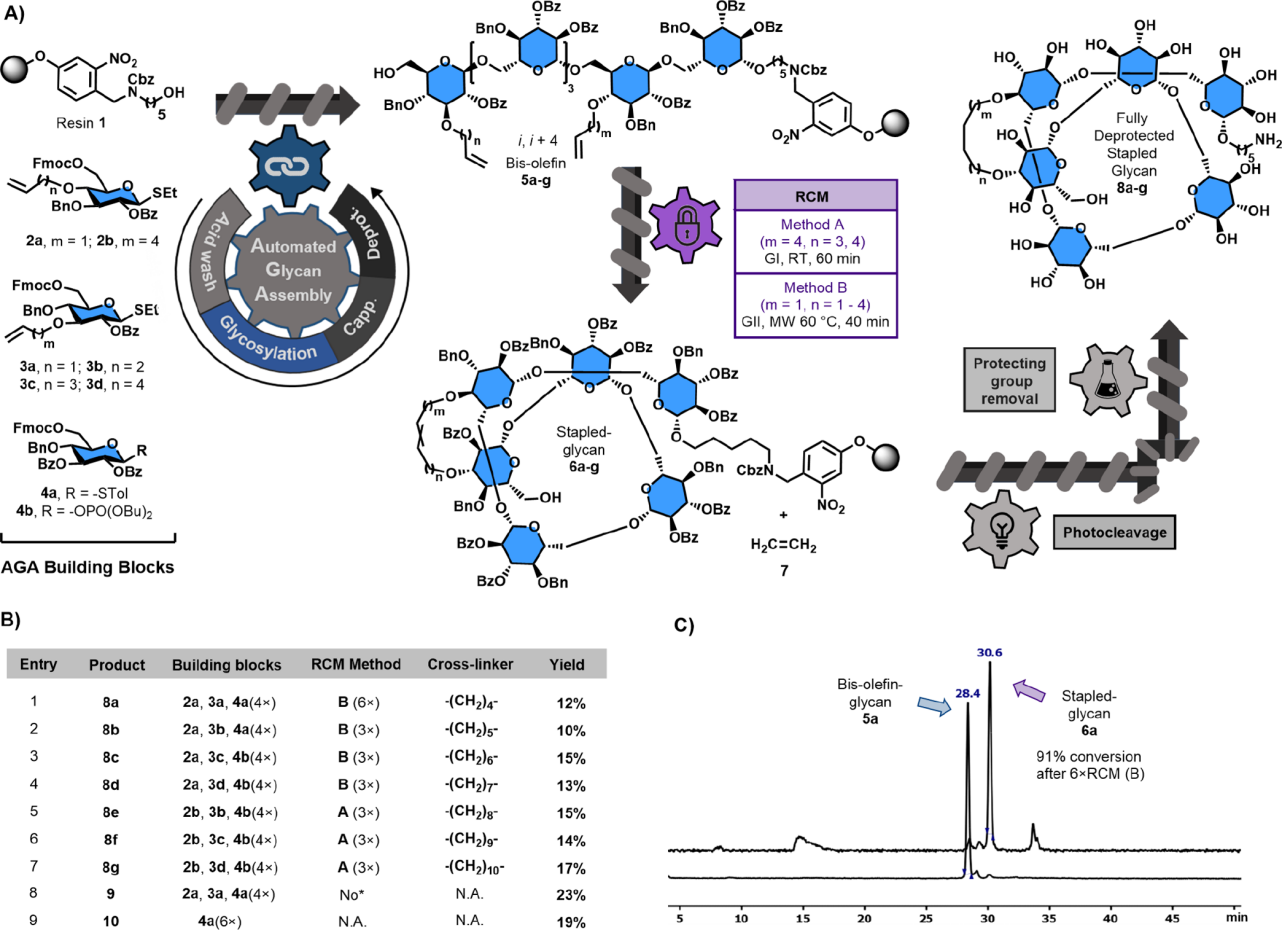
Synthesis of stapled oligosaccharides employing Grubbs metathesis. (A) Schematic representation of the substrates, AGA, and off-resin methodologies to afford stapled glycans. (B) Summary of BBs, methods, and final yields of all stapled and linear glycans. (C) HPLC traces (after microcleavage) of the glycan with the shortest cross-linkerbefore (**5a**) and after (**6a**) RCM. *No RCM was executed to obtain an acyclic glycan bearing hydrocarbon linkages. GI: Grubbs’ first-generation catalyst. GII: Grubbs’ second-generation catalyst.

RCM is among the most reliable methods in generating complex cyclic molecules.^31^ Many chemical features, such as the variety of catalysts, tolerance to many functional groups, high conversion, wide solvent compatibility with solid-phase methods, and an inert hydrocarbon cross-linker, have made RCM a standard method for the late-stage derivatization of peptides, even implemented in automated protocols.^32^ Thus, we selected RCM for the glycan stapling. A minor drawback is that usually a mixture of cis/trans olefins is obtained, requiring a reduction after cyclization. This does not apply to glycan stapling because the final hydrogenolysis step for benzyl ether cleavage will conveniently reduce the hydrocarbon linker as well (Figure 2A).

### AGA of Bis-Olefin Glycans

Automated glycan assembly (AGA) using polystyrene resin equipped with photocleavable linker **1** was performed on a 0.015 mmol scale in a home-built synthesizer.^33^ Glycan elongation relied on sequential cycles of acidic wash, glycosylation, capping, and deprotection (Figure 2A). In most cases, the glycosylation step was based on thioglycoside activation (**2a,b**; **3a–d**; and **4a**), using a sixfold excess of the building block (BB) to ensure complete couplings. Glycosyl phosphate activation was used for **4b** in fourfold excess of BB. The glycosylation required rigorous temperature control, −20 °C (10 min) → 0 °C (20 min) for thioglycosides and −30 °C (5 min) → −10 °C (30 min) for the glycosyl phosphates. Glucose BBs were designed to contain a fluorenylmethoxycarbonyl (Fmoc) temporary protecting group at the C6-OH, while benzyl (Bn) ethers and benzoyl (Bz) esters served as permanent protecting groups. Bz-protection of the C2-OH ensured selective β-glycosylation. Two different sets of glucose BBs containing terminal alkenes of different lengths (**2a,b** and **3a–d**) were synthesized (see the Supporting Information) and incorporated in specific positions within the hexasaccharide sequence. BB **2a,b** (bearing an alkene at C4) was always incorporated in the second position and BB **3a–d** (alkene at C3) was incorporated in the sixth position, while BB **4a,b** was introduced in the remaining positions. Different combinations of BBs **2** and **3** were considered to generate cross-linkers with sizes ranging from 4×CH_2_ (highest rigidity, *m* = 1 and *n* = 1) to 10×CH_2_ (highest flexibility, *m* = 4 and *n* = 4), as detailed in Figure 2B.

Despite the similarity among the series of alkene-modified BBs, some optimizations of the glycosylations were required to guarantee complete conversion. To monitor the reaction, 20–30 beads were taken from the reaction vessel and subjected to microcleavage, high-performance liquid chromatography (HPLC)–mass spectrometry (MS), and matrix-assisted laser desorption ionization time-of-flight mass spectrometry (MALDI-TOF-MS) analysis. Accordingly, it was observed that for the shortest alkenes, allyl (**2a** and **3a**) and 3′-butenyl (**3b**), standard glycosylation conditions (6 equiv of BB and 10 equiv of *N*-iodosuccinimide, NIS) afforded the desired hexamer (**5a,b**) quantitatively. However, for longer alkenyl chains (**2b** and **3c–d**), side reactions started to compete with the glycosylation reaction, since NIS can react with the alkenes and promote the formation of side products.^34^ Using equimolar amounts of NIS with respect to the BB (6 equiv) and reducing the temperature to −25 °C (25 min) → −10 °C (10 min) suppressed the side reaction and resulted in full conversion after two glycosylation cycles. To avoid NIS interference in the subsequent coupling steps, the repetitive glucose BB was introduced in the form of glycosylic phosphate **4b** that is activated by trimethylsilyl trifluoromethanesulfonate and does not have any undesired influence on the glycans bearing long olefin side chains.

### On-Resin Grubbs Metathesis

With bis-olefin oligosaccharides in hand, a module for automated RCM was developed. Analogous to previous studies on peptides, the flexibility of the alkenyl chains (related to the ring size) had a crucial influence on the reaction rate, and therefore, different experimental conditions were required for successful stapling. Initial attempts were performed with the bis-olefin **5d** to generate the cross-linker with the average size of 7×CH_2_ (entry 4), hoping to provide a general method that could be expanded to other molecules. Unfortunately, following the original protocol (method A) described for peptide stapling (Grubbs catalyst 1st gen., dichloroethane, room temperature, argon bubbling),^17,35^ no substantial formation of the desired macrocyclic glycan was observed. Nevertheless, when applying the same conditions to the stapling of glycans with longer alkenes (entries 5, 6, and 7), the desired product was obtained with a notable conversion after a single cycle. The execution of a second cycle increased the conversion substantially, and after three cycles, the bis-olefin glycan was completely consumed.

The generation of cross-linkers with seven methylene groups or less requires the participation of allyl groups in the RCM reaction since all of them were constructed using BB **2a** (*m* = 1). Its small size apparently prevents the interaction between the fully protected glycan and the bulky catalyst. Inspired by the synthesis of highly constrained cyclic peptides,^35^ a more elaborate strategy was designed based on microwave (MW) heating and the use of a Grubbs catalyst with higher reactivity. Accordingly, the same bis-olefin glycan **5d** was placed in a home-built MW-assisted synthesizer^36^ and different experimental conditions were screened. Increasing the temperature to 60 °C using MW heating in the presence of a second-generation Grubbs catalyst for 40 min (method B, three cycles) enabled the formation of shorter cross-linked glycan **6d**, as well as **6c** and **6b**. The better conversion in the MW-assisted RCM is showcased by the successful formation of glycan **6a** (4×CH_2_, *m* = 1 and *n* = 1) from bis-olefin **5a** containing two allyl residues. Six cycles of method B (Figure 2C), afforded the stapled glycan with the shortest cross-linker as the major product (91% conversion).

### Off-Resin Modifications

Photocleavage of the stapled oligosaccharides was the most efficient when two consecutive cleavage cycles were implemented. The fully protected glycans were subjected to a sequential global deprotection consisting of methanolysis and final hydrogenolysis that simultaneously ensured the reduction of the olefin in the cross-linker. The robustness of the overall synthetic manipulations was determined by analysis of the crude HPLC traces (see the Supporting Information), with the formation of the desired stapled glycan in high purity, affording global yields of around 15% after a single purification step.

### Cell Penetration of Stapled Versus Linear Glycans

Lipinski’s rule of five (Ro5) suggests that a molecular weight of below 500 Da, less than 10 H-bond donors and 5 H-bond acceptors, renders a small molecule capable of crossing cell membranes.^37^ The size of bio-oligomers in combination with their intrinsic low lipophilicity and the high number of hydrogen bonds are detrimental when targeting the cell interior.^38^ The introduction of hydrophobic cross-linkers by stapling or amide alkylation aids the cellular uptake of peptides but has not been explored in oligosaccharides to date.^15,39^

**Figure 3:**
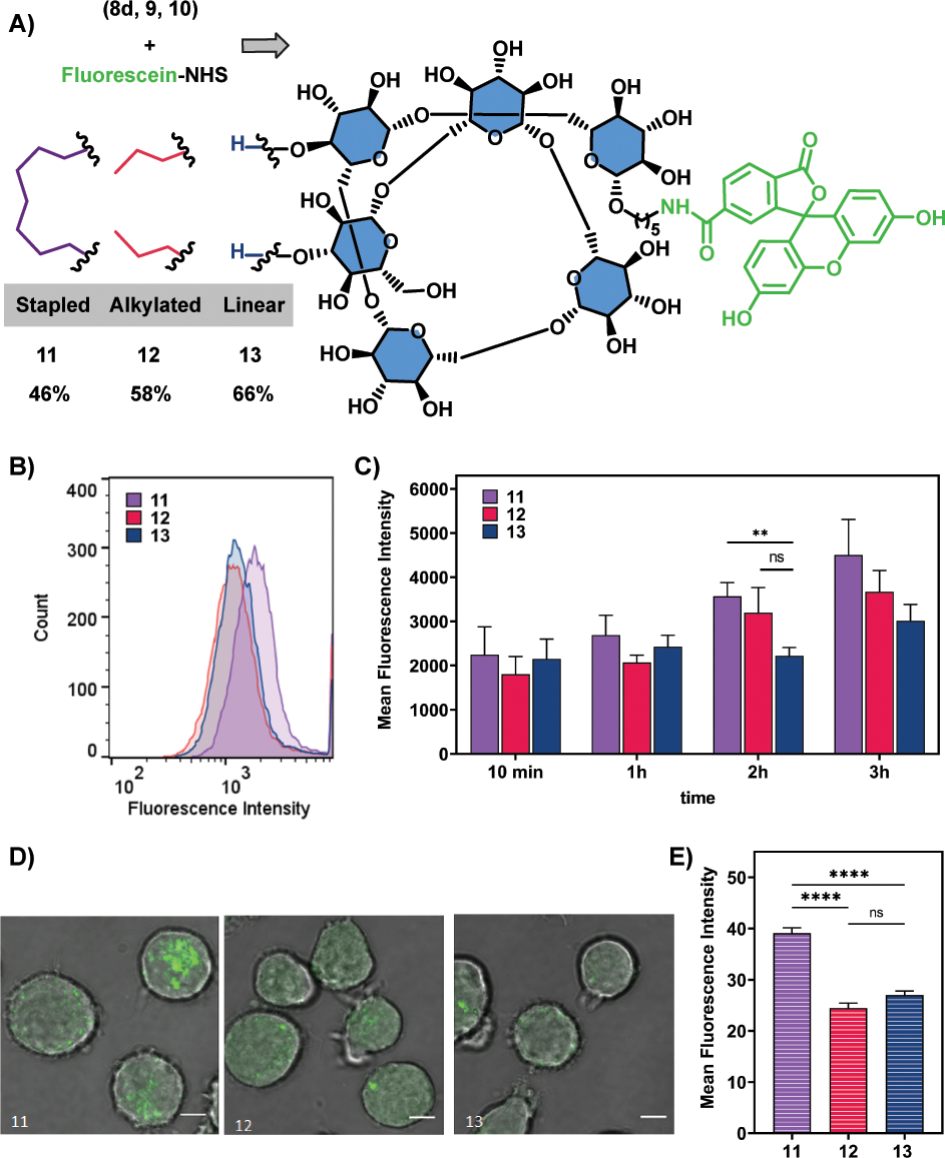
Cell penetration studies. (A) Reaction of glycans **8d**, **9**, and **10** with fluorescein-NHS (1.5 equiv) and Et_3_N (3 equiv) in DMF (2 mL) for 2 h, in the formation of the fluorescein-labeled glycans **11** (46% yield), **12** (58% yield), and **13** (66% yield). (B) Representative flow cytometry histogram of cell penetration after 3 h of incubation at 37 °C of Jurkat cells with glycans **11**, **12**, and **13**. (C) Quantification of flow cytometry of the cell penetration study of glycans **11**, **12**, and **13**. Values represent mean ± SEM. (D) Confocal fluorescence microscopy images of Jurkat cells incubated with the glycans **11**, **12**, and **13** for 3 h at 37 °C. Scale bars correspond to 5 μm. (E) Quantification of confocal fluorescence microscopy. Values represent mean ± SEM. Differences were tested for significance using one-way ANOVA followed by Tukey’s post hoc test with (****) *p* < 0.0001.

To gain insights into the consequences of chemical modifications over the cell-penetration properties of glycans, stapled glycan **8d** was compared with two linear counterparts **9** and **10**. Glycan **9** (Figure 2B, entry 8) contains two propyl chains in the same positions as the cyclized **8d**. Linear glycan **10** is a native β-(1–6)-glucose hexamer (Figure 2B, entry 9). These three glycans were coupled to fluorescein-NHS to generate the fluorescently labeled glycans (stapled **11**, alkylated **12**, and linear **13**) needed for the study (Figure 3A).

Fluorescence-activated cell sorting experiments were envisioned to indicate if **11**, **12**, and **13** penetrate cells differently. For that, Jurkat cells were incubated with the respective glycans for 10 min, 1, 2, and 3 h at 4 or 37 °C . Although at 4 °C, no cell penetration was observed (see Figure S134), at 37 °C (Figure 3B,C), there were cell penetration and differences after 2 h of incubation. Although the alkyl chains in **12** did not offer a clear advantage to linear **13** (Figure 3B), stapled **11** penetrated cells significantly better.

To corroborate these findings, confocal microscopy studies were performed (Figure 3D,E). Using similar experimental conditions, Jurkat cells were incubated for 3 h with the glycans at 37 °C and analyzed by confocal microscopy. Simple visualization (Figure 3D for a zoomed picture, for the full image see Figure S136), suggests that stapled glycan **11** penetrates cells best. Quantification (Figure 3E) demonstrates that although alkylation did not influence cell penetration, stapling was clearly advantageous. Beyond the effect of increasing lipophilicity, the conformational constraints imposed by stapling play a crucial role in cell penetration, as was seen for peptides.^40^

### Influence of Stapling on Enzymatic Stability

Enzymatic degradation is a concern when biopolymers are used in vivo. Cyclization increases peptide half-life inside cells.^15^ The presence of the cross-linker disturbs access to the hydrolyzable sites and introduces constraints that contribute to rendering the molecules enzymatically more stable.^18^

**Figure 4:**
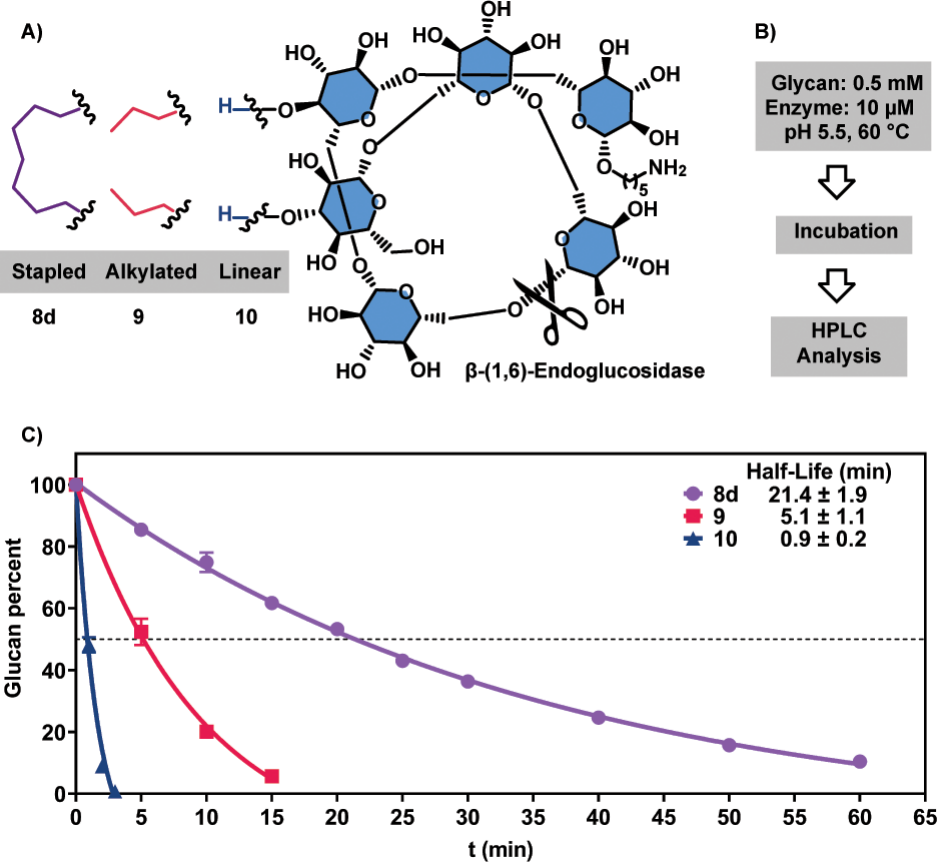
Analysis of the enzymatic stability of stapled and linear glycans. (A) Schematic representation of the hydrolysis of **8d**, **9**, and **10** with a β-endoglucosidase. (B) Optimal experimental conditions for comparative hydrolysis. (C) Enzymatic hydrolysis rates of stapled glycan **8d** (purple), alkylated glycan **9** (red), and linear glycan **10** (blue) during enzymatic degradation, highlighting the different half-lives.

To study the effect of stapling on enzymatic stability of glycans (Figure 4A,B), stapled **8d**, alkylated **9**, and linear glycan **10**, were incubated in the presence of a β-glucosidase, and degradation was monitored by HPLC-MS. Using β-endoglucosidase instead of a β-exoglucosidase should offer key insights, as we target modifications that involve residues along the sequence. Therefore, we utilized a thermostable β-endoglucosidase that selectively hydrolyzes β-(1,6)-glucans with the optimal condition reported to be at 80 °C and pH 5.5.^41^ Acyclic glycans **9** and **10** were completely degraded after only a few minutes under these conditions. At 60 °C, the hydrolysis rate dropped and a comparative study became possible. Unmodified glycan **10** was the least stable with a half-life of 0.9 min (Figure 4C). Alkylated oligosaccharide **9** showed a fivefold increased half-life. Alkylated and cyclized glycan **8d** increased the enzymatic stability 23-fold. Improved stability to hydrolytic enzymes mirrors findings reported for helical peptides and suggests that confining glycans to compact structures alters the conformation required for the interaction with the enzyme.

## Conclusions

We present the design and synthesis of stapled glycans with linkers of different lengths. Chemical modification of oligosaccharides by stapling increased the capability of glycans to cross cell membranes and slows enzymatic degradation drastically. This fundamental approach can be extended to the stabilization of different oligosaccharides and will serve as a basis for other stapling methods. Structural studies to evaluate the effect of the staple on glycan conformation will help to understand the structure–function relationship in glycans. The concepts developed here open possibilities for the future creation of constrained oligosaccharides with potential applications in drug and vaccine development.

## Supporting Information Available

All experimental procedures of synthesis and biological evaluation and characterization data, including HPLC, HR-MS, and NMR (PDF)

## Abbreviations

AGA: automated glycan assembly
BB: building block
NIS: *N*-iodosuccinimide
MW: microwave
RCM: ring-closing metathesis
Ro5: rule of five
