# Supplementary material for: Design, Synthesis, and Characterization of Stapled Oligosaccharides
Source: J Am Chem Soc. 2022 Sep 29;144(40):18429–34. doi: 10.1021/jacs.2c06882 (PMC9562281; doi:10.1021/jacs.2c06882)
Supplement: Supplementary file 1 — ja2c06882_si_001.pdf [file ja2c06882_si_001.pdf]

SUPPORTING INFORMATION

Design, Synthesis and Characterization of Stapled Oligosaccharides

Manuel G. Ricardo,[⊥] Emelie E. Reuber,[⊥,§] Ling Yao,[⊥] José Danglad-Flores,[⊥] Martina Delbianco,[⊥] Peter H. Seeberger*[,⊥,§]

Contents

1. General information 1
2. Building block synthesis 2
3. Automated glycan assembly 51
 3.1. Preparation of reagent solutions 51
 3.2. Modules for Automated Solid-Phase Synthesis 52
 3.3. Post-solid-phase manipulations 56
 3.4 Oligosaccharide synthesis 58
  Synthesis and analytical data of **8a** 58
  Synthesis and analytical data of **8b** 62
  Synthesis and analytical data of **8c** 66
  Synthesis and analytical data of **8d** 70
  Synthesis and analytical data of **8e** 74
  Synthesis and analytical data of **8f** 78
  Synthesis and analytical data of **8g** 82
  Synthesis and analytical data of **9** 86
  Synthesis and analytical data of **10** 90
  Synthesis and analytical data of **11** 92
  Synthesis and analytical data of **12** 95
  Synthesis and analytical data of **13** 98
4. FACS analysis of glycan-treated cells 101
5. Confocal microscopy 102
6. Enzymatic stability assay 104
7. References 106

1. General information

All chemicals used were reagent grade and used as supplied unless otherwise noted. The automated glycan syntheses and ring-closing metathesis were performed on home-built synthesizers developed at the Max Planck Institute of Colloids and Interfaces.[1,2] Analytical thin-layer chromatography (TLC) was performed on Merck silica gel 60 F254 plates (0.25 mm). Compounds were visualized by UV irradiation or dipping the plate in a staining solution (sugar stain: 10% H_2SO_4 in EtOH; CAM: 48 g/L ammonium molybdate, 60 g/L ceric ammonium molybdate in 6% H_2SO_4 aqueous solution). Flash column chromatography was carried out by using the forced flow of the indicated solvent on Fluka Kieselgel 60 M (0.04 – 0.063 mm). Analysis and purification by normal and reverse-phase HPLC were performed by using an Agilent 1200 series. Products were lyophilized using a Christ Alpha 2-4 LD plus freeze dryer. 1H, 13C, and HSQC NMR spectra were recorded on a Varian 400-MR (400 MHz), Varian 600-MR (600 MHz), or Bruker Biospin AVANCE700 (700 MHz) spectrometer. Spectra were recorded in $CDCl_3$ by using the solvent residual peak chemical shift as the internal standard ($CDCl_3$: 7.26 ppm 1H, 77.0 ppm 13C) or in D_2O using the solvent as the internal standard in 1H NMR (D_2O: 4.79 ppm 1H). High-resolution mass spectra were acquired using a 6210 ESI-TOF mass spectrometer (Agilent) and a MALDI-TOF autoflexTM (Bruker). MALDI and ESI mass spectra were run on IonSpec Ultima instruments.

2. Building block synthesis

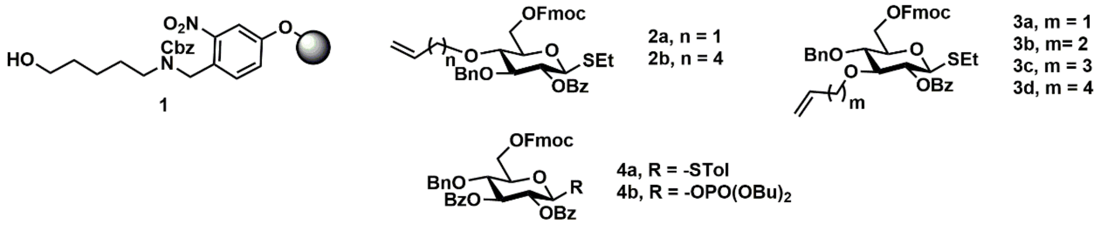

Photolabile resin **1** was synthesized according to previous protocols[3] and BB **4a** was acquired from a commercial source (GlycoUniverse GmbH & Co. KGaA, Germany).

2.1. Synthesis of 4-O-alkenyl glucose BBs (2a,b)

*Ethyl 2-O-benzoyl-3-O-benzyl-4,6-O-benzylidene-1-thio-β-D-glucopyranoside (**2-1**)*

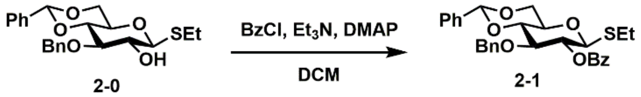

Similar to a previous report,[4] **2-0** (7.8 g, 19.4 mmol) is dissolved in DCM (40 mL). Et_3N (13.6 mL, 97.0 mmol) and DMAP (0.47 g, 3.88 mmol) are added and the mixture is placed in an ice bath. At 0 °C, BzCl (3.37 mL, 7.5 mmol) is added dropwise and the reaction is stirred initially for 30 min, and then it is left to reach room temperature and stirred for additional 12 h. The reaction mixture is diluted with chloroform (50 mL), transferred to a separatory funnel, and washed with a 10% soln. of citric acid (2×20 mL) and brine (2×20 mL). The organic layer is dried over Na_2SO_4 and evaporated at reduced pressure. The crude product is purified by column chromatography (hex/EA 10:1) to afford **2-1** (8.05 g, 82% yield) as a white solid. R*f* (hex/EA 6:1) = 0.65. ^{1}H NMR (400 MHz, $CDCl_3$), δ = 8.06 – 8.01 (m, 2H), 7.62 (ddt, *J* = 8.8, 7.0, 1.3 Hz, 1H), 7.56 – 7.39 (m, 7H), 7.23 – 7.06 (m, 5H), 5.65 (s, 1H), 5.41 – 5.33 (m, 1H), 4.85 (d, *J* = 11.9 Hz, 1H), 4.73 (d, *J* = 11.9 Hz, 1H), 4.65 (d, *J* = 10.0 Hz, 1H), 4.44 (dd, *J* = 10.5, 5.0 Hz, 1H), 3.97 – 3.83 (m, 3H), 3.59 (ddd, *J* = 9.9, 8.8, 4.9 Hz, 1H), 2.75 (qd, *J* = 7.4, 3.5 Hz, 2H), 1.25 (t, *J* = 7.5 Hz, 3H). ^{13}C NMR (101 MHz, $CDCl_3$), δ = 165.18, 137.78, 137.20, 133.23, 129.95, 129.75, 129.09, 128.40, 128.33, 128.19, 128.08, 127.60, 126.03, 101.30, 84.33, 81.69, 79.21, 74.24, 71.87, 70.74, 68.67, 24.05, 14.83.

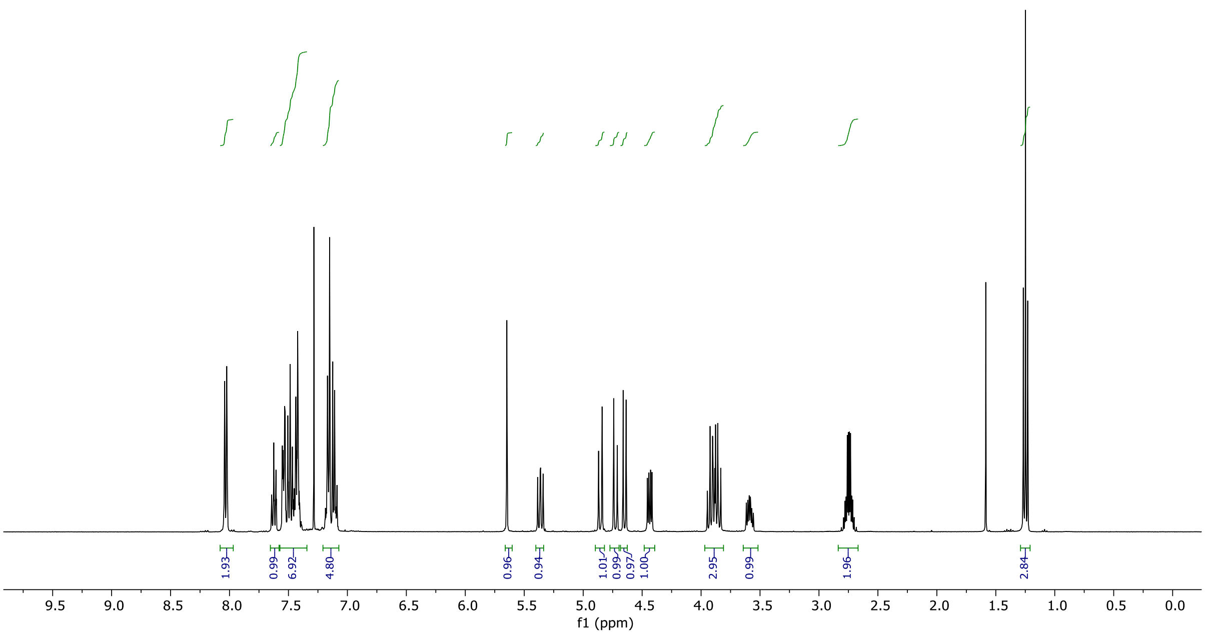

Figure S1. ^{1}H NMR (400 MHz, $CDCl_3$) spectrum of **2-1**.

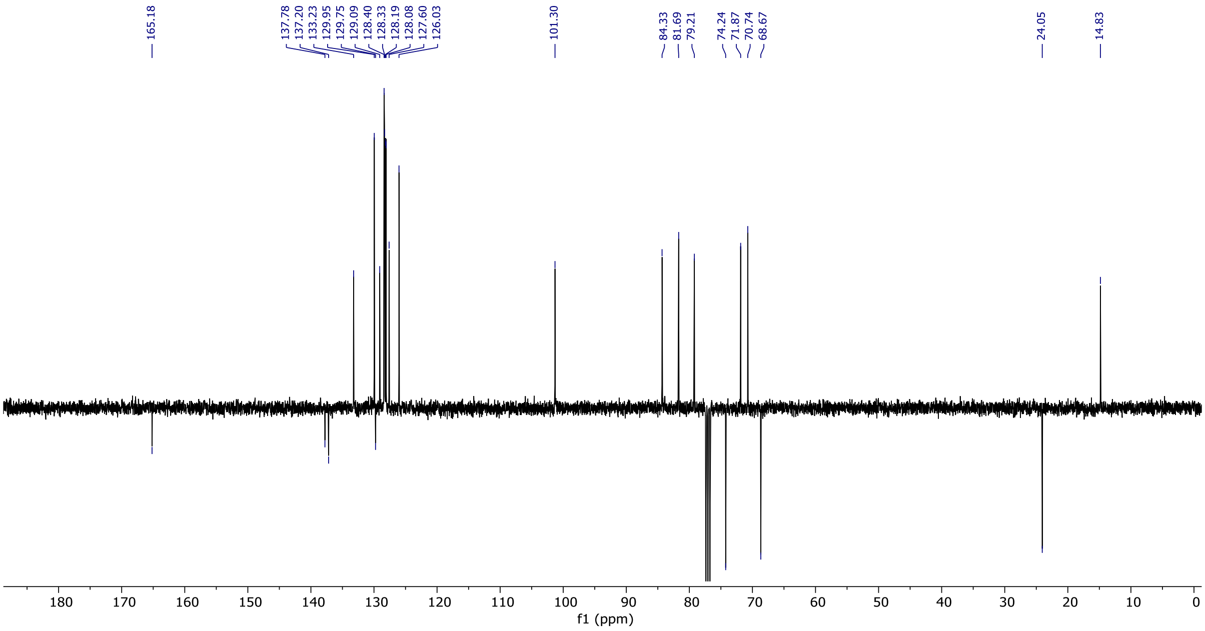

Figure S2. ^{13}C APT NMR (101 MHz, $CDCl_3$) spectrum of **2-1**.

Ethyl 2-O-benzoyl-3-O-benzyl-6-O-trityl-1-thio-β-D-glucopyranoside ***(2-3)***

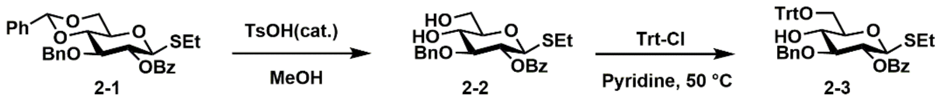

Similar to a previous report,[5] **2-1** (5.85 g, 11.6 mmol) is suspended in MeOH (40 mL), and in the presence of TsOH (0.45 g, 2.3 mmol) the reaction mixture is subjected to ultrasonic bath for 30 min. The clear solution is quenched with Et_3N (1 mL) and the volatiles are evaporated

under reduced pressure. The crude is dissolved with Trt-Cl (6.0 g, 23.1 mmol) in pyridine (50 mL), and the reaction mixture is stirred at 50°C overnight.[6] The reaction is quenched with MeOH (5 mL) and the volatiles are evaporated at reduced pressure. The crude is dissolved in ethyl acetate (50 mL) and washed with a 10% soln. of $NaHCO_3$ (2×20 mL) and brine (2×20 mL). The organic layer is dried over Na_2SO_4, evaporated at reduced pressure. The crude product is purified by column chromatography (hex/EA 4:1) to afford **2-3** (5.4 g, 71% yield) as a white solid. R*f* (hex/EA 4:1) = 0.48. ^{1}H NMR (400 MHz, $CDCl_3$), δ = 8.11 – 8.06 (m, 2H), 7.65 – 7.59 (m, 1H), 7.50 (dq, *J* = 6.5, 1.6 Hz, 7H), 7.40 – 7.33 (m, 6H), 7.28 (tt, *J* = 5.9, 1.3 Hz, 4H), 5.35 (dd, *J* = 10.0, 9.1 Hz, 1H), 4.74 (d, *J* = 1.5 Hz, 2H), 4.60 (d, *J* = 10.0 Hz, 1H), 3.90 (td, *J* = 9.2, 2.6 Hz, 1H), 3.71 (t, *J* = 9.0 Hz, 1H), 3.58 – 3.49 (m, 1H), 3.48 – 3.39 (m, 2H), 2.83 (dd, *J* = 12.5, 7.4 Hz, 1H), 2.78 – 2.68 (m, 1H), 2.60 (d, *J* = 2.7 Hz, 1H), 1.30 (t, *J* = 7.4 Hz, 3H). ^{13}C NMR (101 MHz, $CDCl_3$), δ = 165.34, 143.64, 137.94, 133.22, 129.90, 128.68, 128.46, 128.40, 128.02, 127.97, 127.80, 127.20, 87.06, 83.64, 83.33, 78.33, 74.83, 72.09, 72.06, 64.01, 23.68, 15.05.

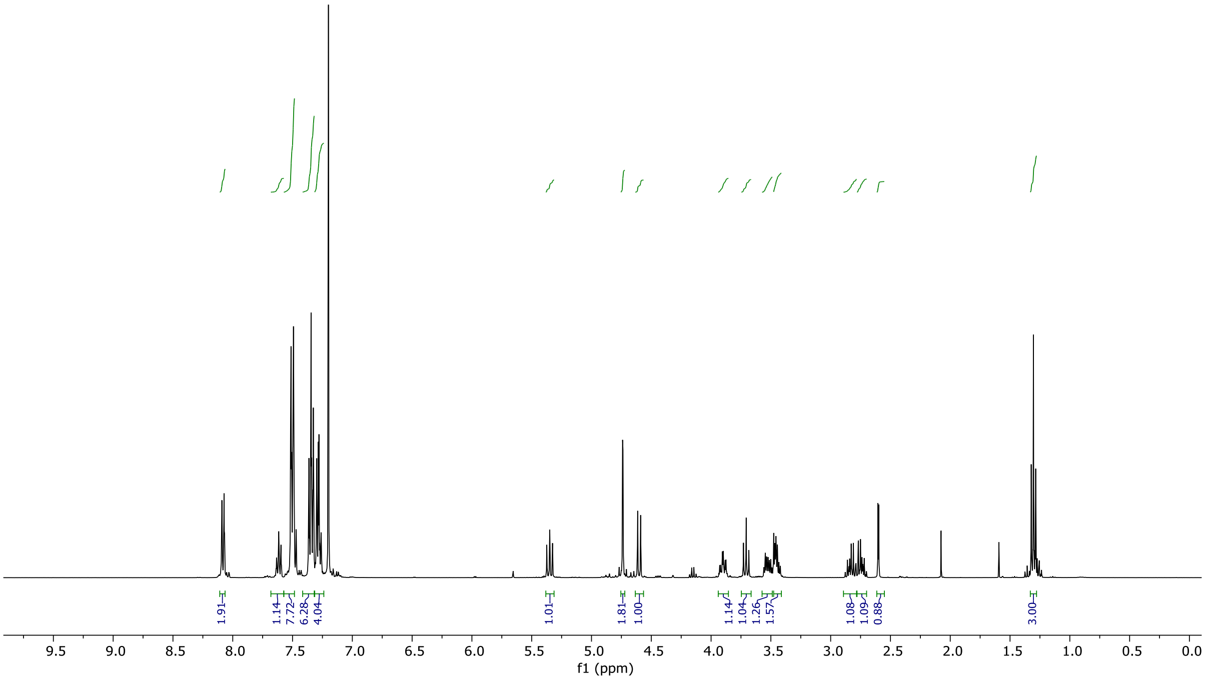

Figure S3. ^{1}H NMR (400 MHz, $CDCl_3$) spectrum of **2-3**.

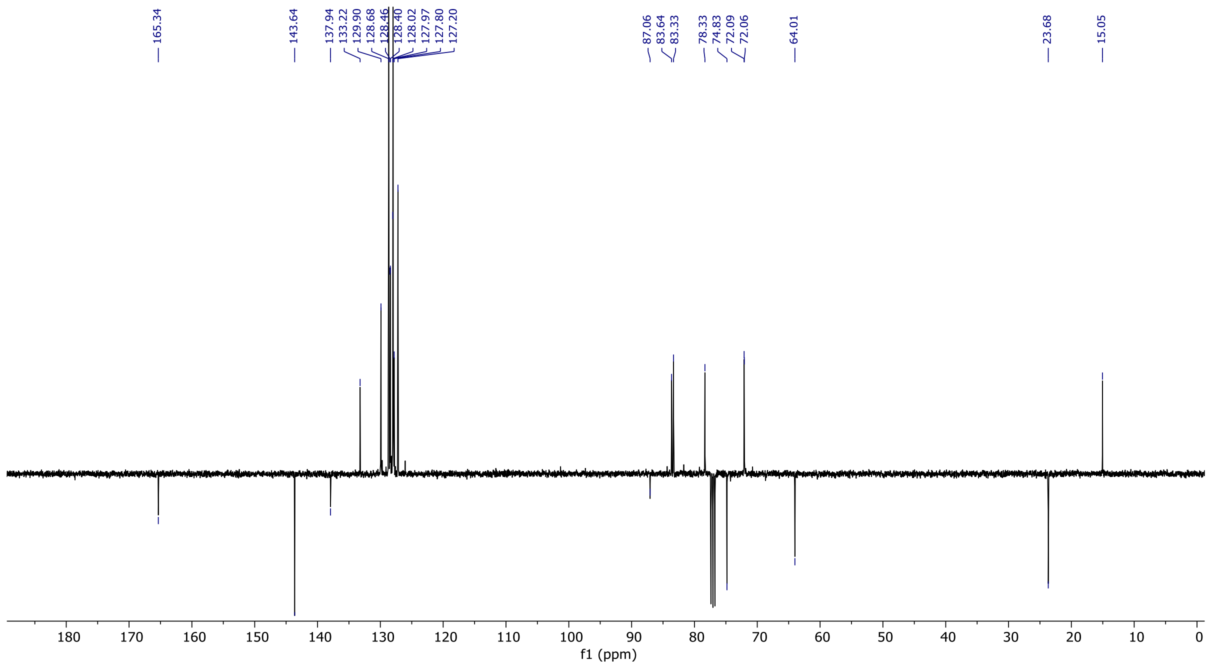

Figure S4. ^{13}C APT NMR (101 MHz, $CDCl_3$) spectrum of **2-3**.

*Ethyl 4-O-alkenyl-2-O-benzoyl-3-O-benzyl-1-thio-β-*D*-glucopyranoside (**2-5**)*

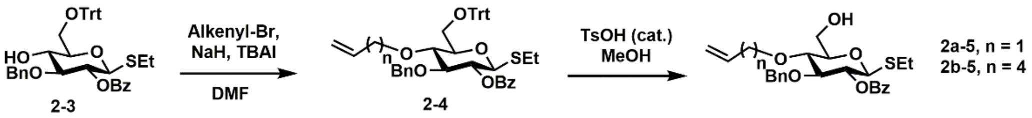

General procedure:[5] **2.3** (2.0 g, 3.0 mmol) dissolved together with alkenyl bromide (6.0 mmol) and a catalytic amount of TBAI (110 mg, 0.3 mmol) in DMF (20 mL). The reaction mixture is placed in an ice bath, and at 0°C, NaH (60% in mineral oil, 0.24 g, 6.0 mmol) is added portion-wise. At this temperature, the mixture is stirred for 2 h and then left to reach RT and stirred for an additional 10 h. The reaction is quenched with a 10% soln. of citric acid (10 mL) and diluted with ethyl acetate (50 mL). The organic layer was washed with water (2×20 mL), brine (2×20 mL), and dried over Na_2SO_4. After removing the solvent, the crude is suspended in MeOH/DCM 3:1 (40 mL), and in the presence of TsOH (115 mg, 0.6 mmol) the reaction mixture is subjected to an ultrasonic bath for 30 min. The clear solution is quenched with Et_3N (1 mL) and the volatiles are evaporated under reduced pressure. The crude product is purified by column chromatography (hex/EA 3:1) to afford **2-5** as a white solid.

*Ethyl 4-O-allyl-2-O-benzoyl-3-O-benzyl-1-thio-β-*D*-glucopyranoside (**2a-5**)*

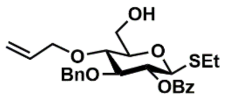

2a-5

2-3 (2.0 g, 3.0 mmol) and allyl bromide (0.52 mL, 6.0 mmol) are allowed to react according to the general procedure detailed above to afford **3e-2** (0.94 g, 68% yield) as a white solid. R*f* (hex/ EA 2:1) = 0.51. ^1H NMR (400 MHz, $CDCl_3$), δ =

8.07 – 8.02 (m, 2H), 7.65 – 7.57 (m, 1H), 7.51 – 7.45 (m, 2H), 7.22 – 7.13 (m, 5H), 5.94 (ddt, *J* = 17.2, 10.4, 5.8 Hz, 1H), 5.35 – 5.25 (m, 2H), 5.23 – 5.19 (m, 1H), 4.76 (d, *J* = 11.1 Hz, 1H), 4.67 (d, *J* = 11.1 Hz, 1H), 4.59 (d, *J* = 10.0 Hz, 1H), 4.36 (ddt, *J* = 12.3, 5.6, 1.4 Hz, 1H), 4.25 – 4.18 (m, 1H), 3.97 (dd, *J* = 12.0, 2.7 Hz, 1H), 3.87 – 3.76 (m, 2H), 3.60 (dd, *J* = 9.8, 8.9 Hz, 1H), 3.47 (ddd, *J* = 9.8, 4.7, 2.7 Hz, 1H), 2.73 (qd, *J* = 7.4, 2.9 Hz, 2H), 1.25 (t, *J* = 7.5 Hz, 3H). ^{13}C NMR (101 MHz, $CDCl_3$), δ = 165.26, 137.71, 134.40, 133.22, 129.86, 129.81, 128.44, 128.27, 128.05, 127.70, 117.55, 83.83, 83.72, 79.71, 77.53, 75.25, 73.95, 72.28, 62.04, 24.13, 14.91.

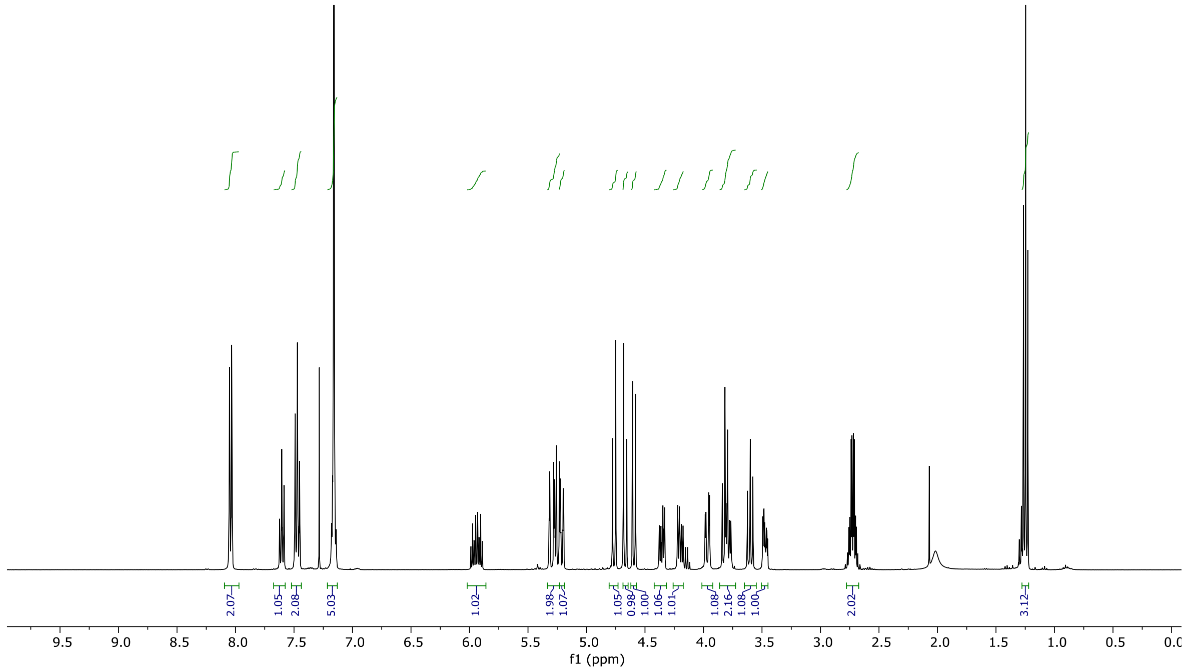

Figure S5. ^{1}H NMR (400 MHz, $CDCl_3$) spectrum of **2a-5**.

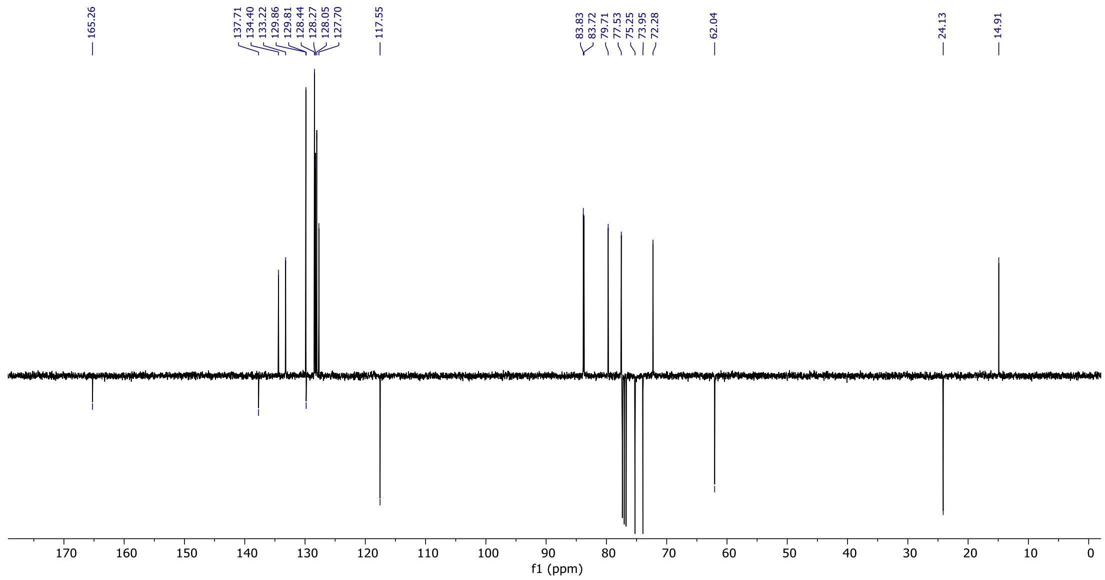

Figure S5. ^{13}C APT NMR (101 MHz, $CDCl_3$) spectrum of **2a-5**.

*Ethyl 2-O-benzoyl-3-O-benzyl-4-O-(hex-5-en-1-yl)-1-thio-β-D-glucopyranoside (**2b-5**)*

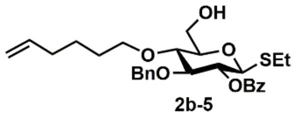

2b-5

2-3 (2.0 g, 3.0 mmol) and 6-bromo-1-hexene (0.80 mL, 6.0 mmol) are allowed to react according to the general procedure detailed above to afford **2b-5** (1.1 g, 73% yield) as a white solid. R*f* (hex/ EA 2:1) = 0.59. ^{1}H NMR (400 MHz, $CDCl_3$), δ = 8.08 – 8.00 (m, 2H), 7.65 – 7.55 (m, 1H), 7.50 – 7.43 (m, 2H), 7.20 – 7.13 (m, 5H), 5.80 (ddt, *J* = 16.9, 10.2, 6.7 Hz, 1H), 5.25 (dd, *J* = 10.1, 9.1 Hz, 1H), 5.06 – 4.93 (m, 2H), 4.76 (d, *J* = 11.1 Hz, 1H), 4.67 (d, *J* = 11.1 Hz, 1H), 4.58 (d, *J* = 10.1 Hz, 1H), 3.96 (dd, *J* = 12.0, 2.6 Hz, 1H), 3.90 – 3.74 (m, 3H), 3.63 (dt, *J* = 9.1, 6.7 Hz, 1H), 3.56 – 3.50 (m, 1H), 3.44 (ddd, *J* = 9.8, 4.6, 2.6 Hz, 1H), 2.72 (qd, *J* = 7.4, 2.9 Hz, 2H), 2.07 (dtt, *J* = 8.0, 6.7, 1.4 Hz, 2H), 1.65 – 1.54 (m, 2H), 1.51 – 1.40 (m, 2H), 1.25 (t, *J* = 7.5 Hz, 3H). ^{13}C NMR (101 MHz, $CDCl_3$), δ = 165.26, 138.54, 137.78, 133.19, 129.85, 128.42, 128.26, 127.96, 127.67, 114.72, 83.85, 83.70, 79.83, 78.13, 75.19, 73.25, 72.28, 62.09, 33.56, 29.80, 25.42, 24.11, 14.91.

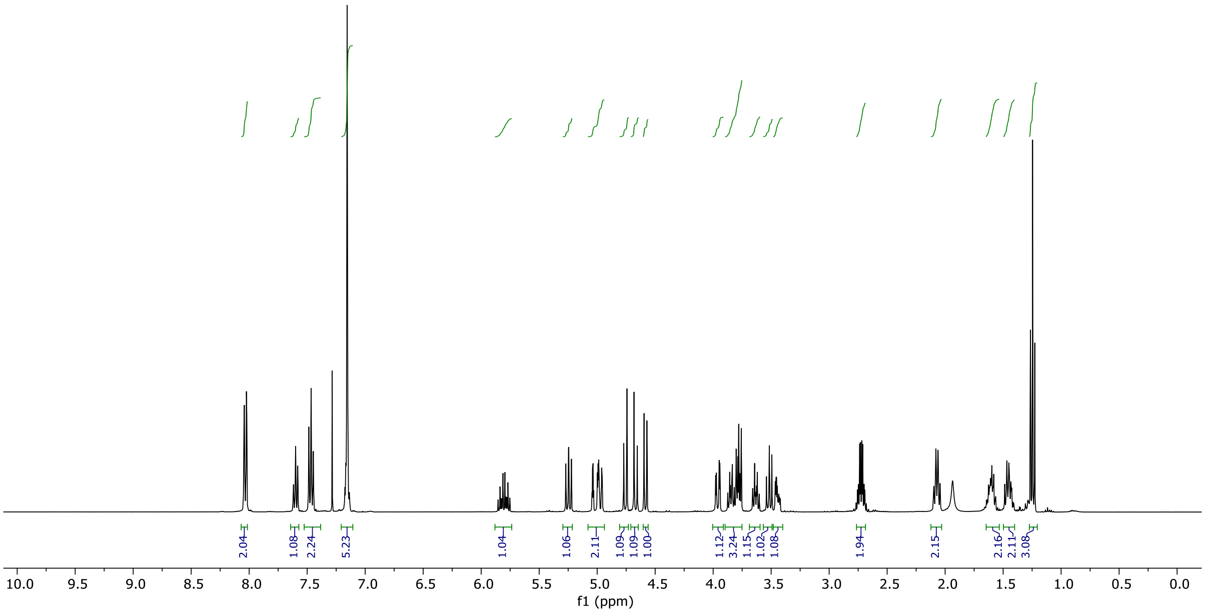

Figure S6. ^{1}H NMR (400 MHz, $CDCl_3$) spectrum of **2b-5**.

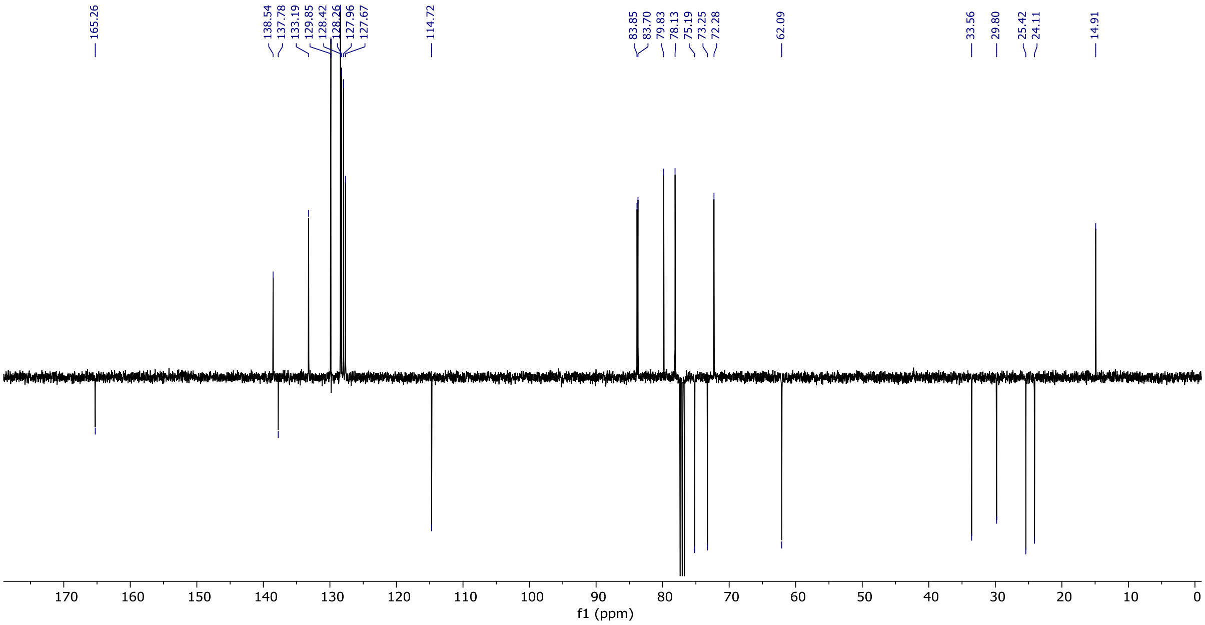

Figure S7. ^{13}C APT NMR (101 MHz, $CDCl_3$) spectrum of **2b-5**.

*Ethyl 4-O-alkenyl-2-O-benzoyl-3-O-benzyl-6-O-(9-fluorenylmethoxycarbonyl)-1-thio-β-D-glucopyranoside (**2-6**)*

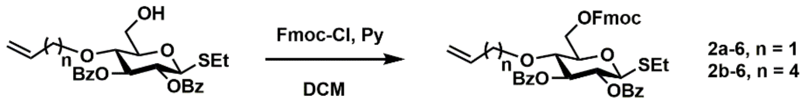

General procedure: Similar to a previous report,[2] **2-5** (2.0 mmol) is dissolved in DCM (30 mL), pyridine (0.32 mL, 4.0 mmol) is added and the mixture is placed in an ice bath. At 0 °C, Fmoc-Cl (0.62 g, 2.4 mmol) is added portion-wise and the reaction is stirred initially for 30 min, and then it is left to reach room temperature and stirred for an additional 4 h. The reaction mixture is diluted with chloroform (50 mL), transferred to a separatory funnel, and washed with a 10% soln. of citric acid (2×20 mL) and brine (2×20 mL). The organic layer is dried over Na_2SO_4 and evaporated at reduced pressure. The crude product is purified by column chromatography (hex/EA 6:1) to afford **2-6** as a white solid.

*Ethyl 4-O-allyl-2-O-benzoyl-3-O-benzyl-6-O-(9-fluorenylmethoxycarbonyl)-1-thio-β-D-glucopyranoside (**2a-6**)*

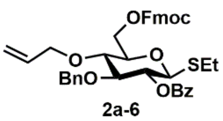

2a-6

2a-5 (1.2 g, 2.6 mmol), Fmoc-Cl (0.81 g, 3.12 mmol) and pyridine (0.42 mL, 5.2 mmol) are allowed to react in DCM (30 mL) according to the general procedure detailed above to afford **2a-6** (1.54 g, 86% yield) as a white solid. R*f* (hex/ EA 3:1) = 0.50. R*f* (hex/EA 4:1) = 0.50. HR-MS *m/z* = 717.2142 $[M+Na]^+$, calcd for $C_{40}H_{38}NaO_9S$: 717.2174. ^1H NMR (400 MHz, $CDCl_3$), δ = 8.09 – 8.03 (m, 2H), 7.80

(dt, *J* = 7.6, 1.0 Hz, 2H), 7.68 – 7.64 (m, 2H), 7.63 – 7.57 (m, 1H), 7.47 (ddd, *J* = 14.5, 7.0, 1.3 Hz, 4H), 7.36 (tt, *J* = 7.5, 1.4 Hz, 2H), 7.18 (dq, *J* = 3.1, 1.5 Hz, 5H), 5.92 (ddt, *J* = 17.2, 10.3, 5.8 Hz, 1H), 4.76 (d, *J* = 11.0 Hz, 1H), 4.69 (d, *J* = 11.0 Hz, 1H), 4.59 (d, *J* = 10.0 Hz, 1H), 4.55 (dd, *J* = 11.6, 2.0 Hz, 1H), 4.44 (dd, *J* = 7.5, 2.3 Hz, 2H), 4.41 – 4.38 (m, 2H), 4.38 – 4.34 (m, 1H), 4.30 (t, *J* = 7.4 Hz, 1H), 4.20 – 4.11 (m, 1H), 3.84 (t, *J* = 8.8 Hz, 1H), 3.67 (ddd, *J* = 9.9, 5.3, 2.0 Hz, 1H), 3.60 (dd, *J* = 9.9, 8.6 Hz, 1H), 2.81 – 2.64 (m, 2H), 1.24 (t, *J* = 7.4 Hz, 3H). ^{13}C NMR (101 MHz, $CDCl_3$), δ = 165.24, 155.05, 143.39, 143.31, 141.31, 137.60, 134.15, 133.24, 129.87, 129.80, 128.46, 128.31, 128.07, 127.94, 127.78, 127.23, 125.24, 125.20, 120.09, 117.84, 84.07, 83.63, 77.42, 77.22, 75.35, 74.02, 72.18, 70.06, 66.68, 46.72, 24.09, 14.94.

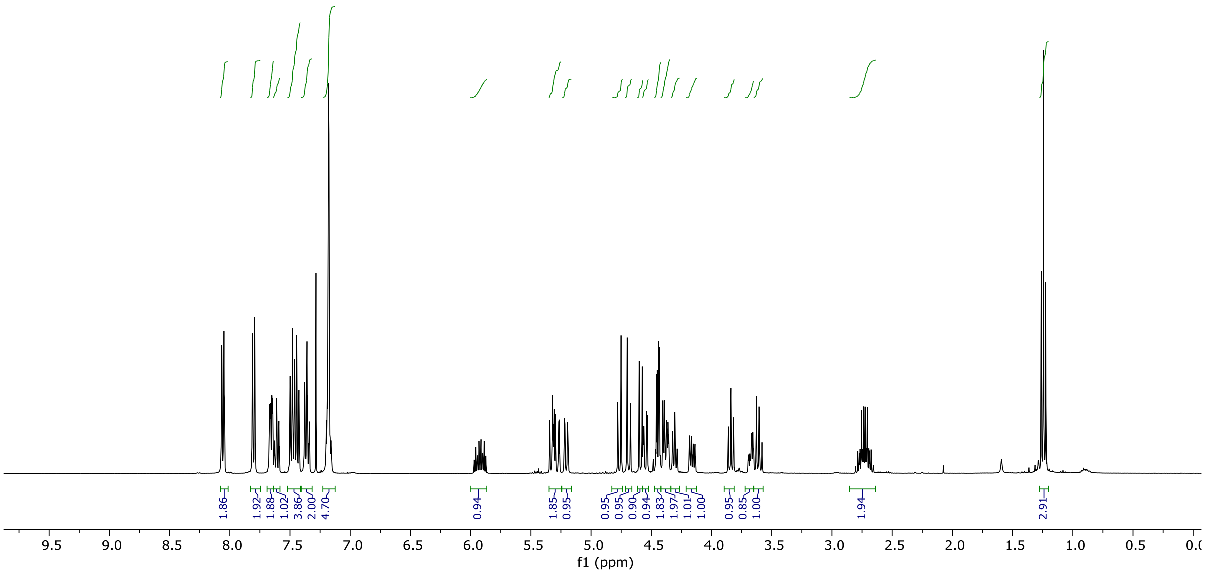

Figure S8. ^{1}H NMR (400 MHz, $CDCl_3$) spectrum of **2a-6**.

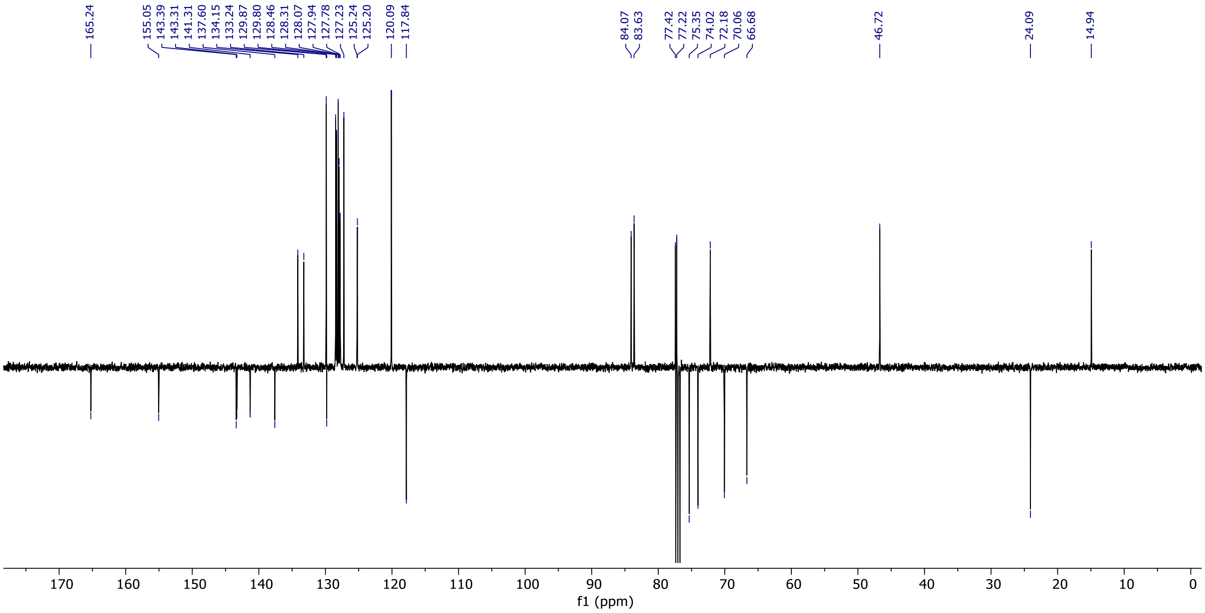

Figure S9. ^{13}C APT NMR (101 MHz, $CDCl_3$) spectrum of **2a-6**.

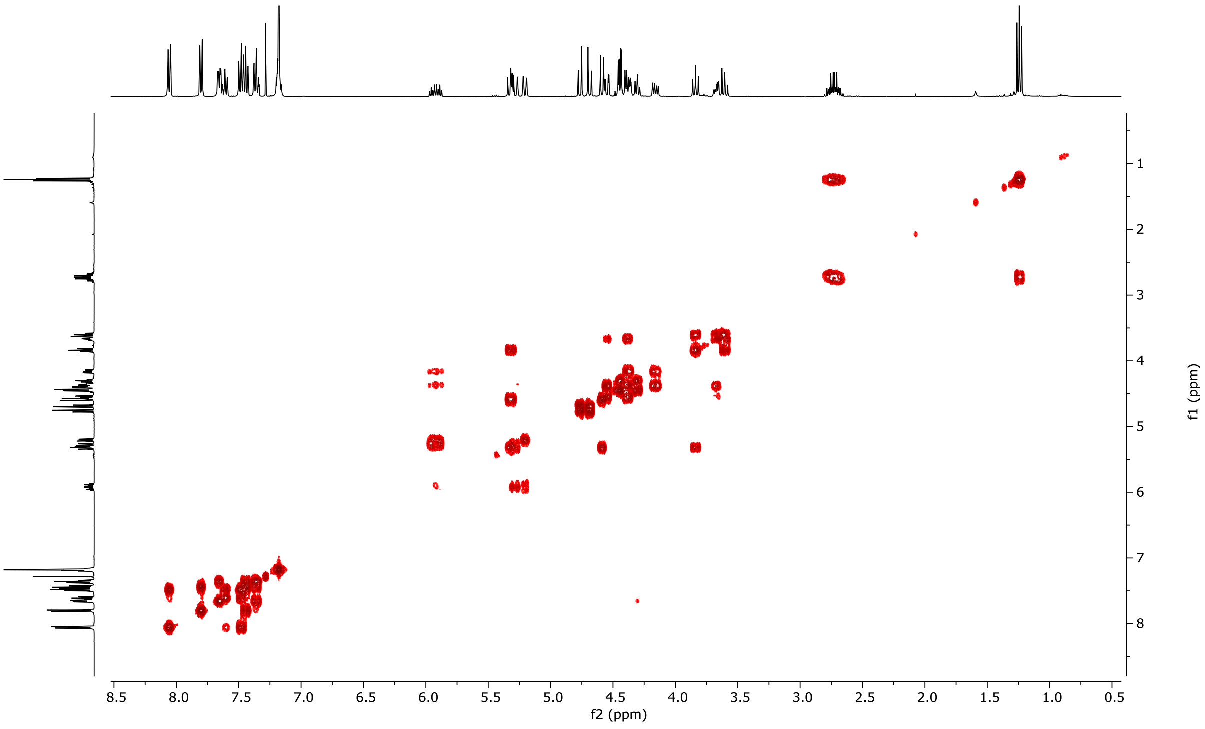

Figure S10. COSY NMR (400 MHz, $CDCl_3$) spectrum of **2a-6**.

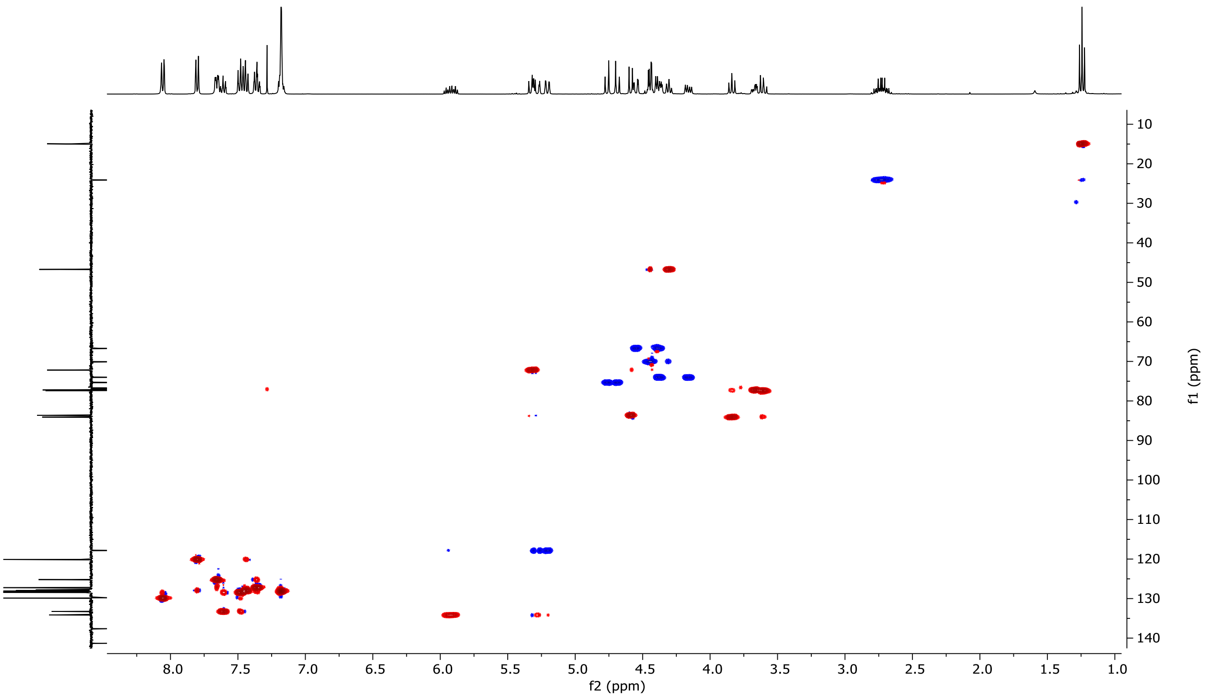

Figure S11. HSQC NMR (400MHz, $CDCl_3$) spectrum of **2a-6**.

*Ethyl 2-O-benzoyl-3-O-benzyl-6-O-(9-fluorenylmethoxycarbonyl)-4-O-(hex-5-en-1-yl)-1-thio-β-D-glucopyranoside (**2b-6**)*

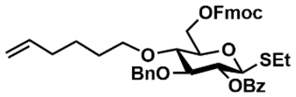

2b-6

2b-5 (1.5 g, 3.0 mmol), Fmoc-Cl (0.93 g, 3.6 mmol) and pyridine (0.48 mL, 6.0 mmol) are allowed to react in DCM (30 mL) according to the general procedure detailed above to afford **2b-6** (1.60 g, 74% yield) as a white solid. R*f* (hex/ EA 3:1) = 0.58. R*f* (hex/EA 4:1) = 0.50. HR-MS *m/z* = 759.2624 $[M+Na]^+$, calcd for $C_{43}H_{44}NaO_9S$: 759.2604 ^1H NMR (400 MHz, $CDCl_3$), δ = 8.09 – 8.03 (m, 2H), 7.80 (dt, *J* = 7.6, 0.9 Hz, 2H), 7.70 – 7.64 (m, 2H), 7.64 – 7.55 (m, 1H), 7.52 – 7.41 (m, 4H), 7.36 (tdd, *J* = 7.5, 2.5, 1.2 Hz, 2H), 7.21 – 7.14 (m, 5H), 5.79 (ddt, *J* = 16.9, 10.2, 6.7 Hz, 1H), 5.31 (dd, *J* = 10.0, 9.1 Hz, 1H), 5.05 – 4.93 (m, 2H), 4.76 (d, *J* = 11.0 Hz, 1H), 4.69 (d, *J* = 11.0 Hz, 1H), 3.88 (dt, *J* = 9.1, 6.5 Hz, 1H), 3.80 (t, *J* = 9.0 Hz, 1H), 3.69 – 3.61 (m, 1H), 3.59 – 3.49 (m, 2H), 2.73 (ttd, *J* = 12.5, 7.5, 5.0 Hz, 2H), 2.13 – 1.99 (m, 2H), 1.66 – 1.55 (m, 2H), 1.49 – 1.39 (m, 2H), 1.24 (t, *J* = 7.4 Hz, 3H). ^{13}C NMR (101 MHz, $CDCl_3$), δ = 165.24, 155.06, 143.39, 143.32, 141.31, 138.48, 137.67, 133.21, 129.91, 129.87, 129.82, 128.44, 128.35, 128.30, 127.98, 127.94, 127.73, 127.22, 125.25, 125.20, 120.08, 114.76, 84.04, 83.60, 78.11, 77.39, 75.28, 73.38, 72.19, 70.08, 66.71, 46.71, 33.54, 29.77, 25.39, 24.06, 14.93.

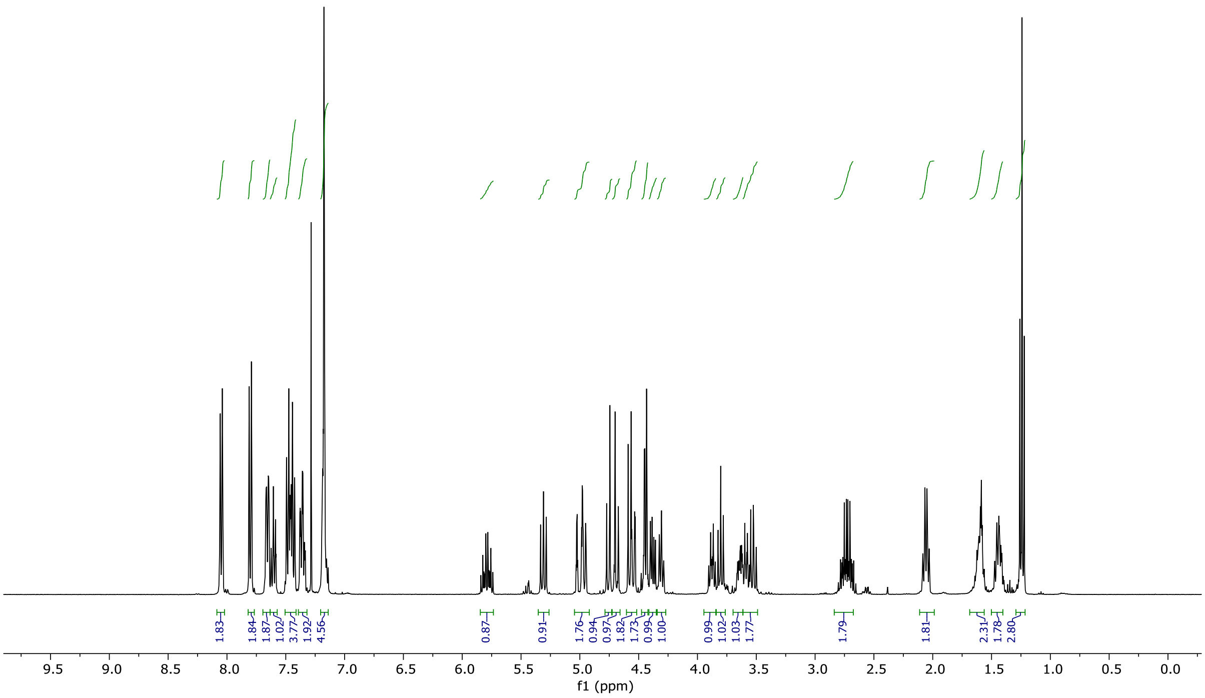

Figure S12. ^{1}H NMR (400 MHz, $CDCl_3$) spectrum of **2b-6**.

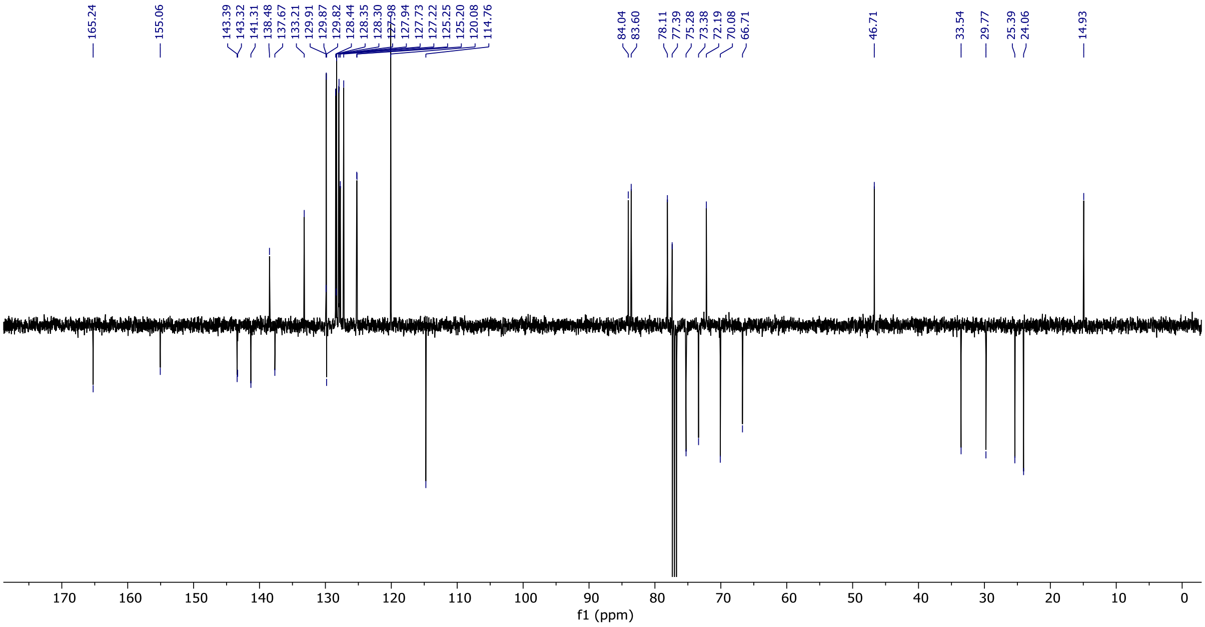

Figure S13. ^{13}C APT NMR (101 MHz, $CDCl_3$) spectrum of **2b-6**.

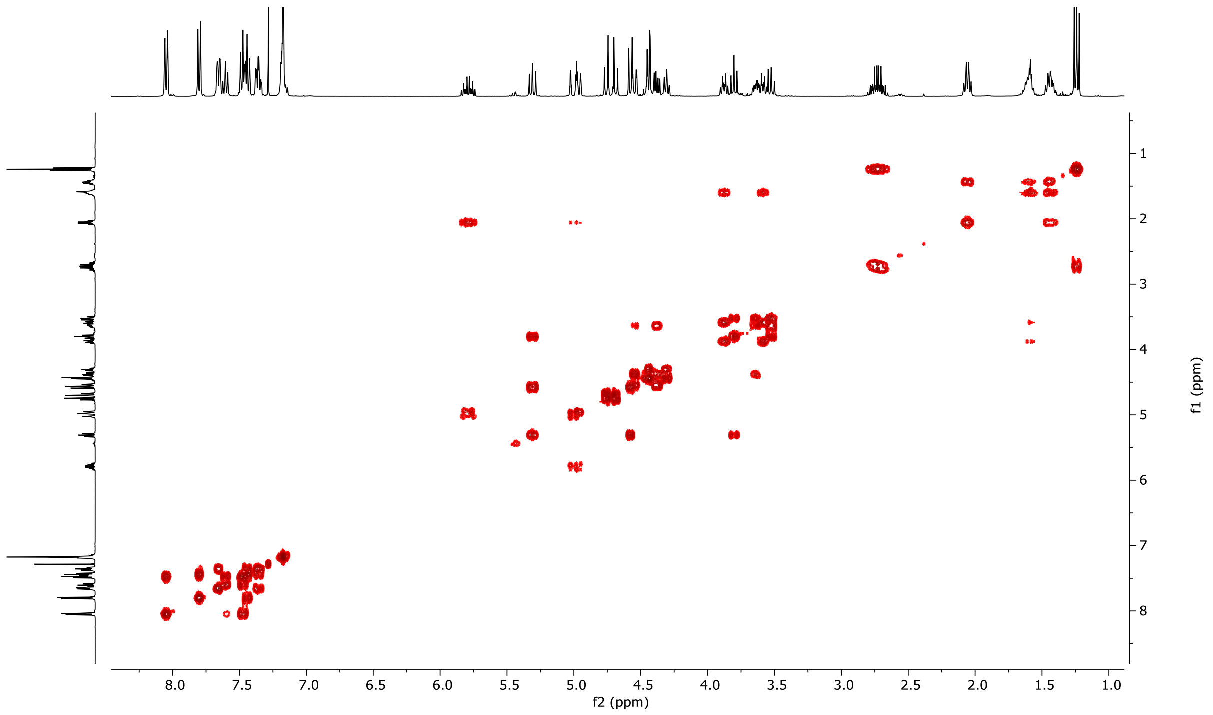

Figure S14. COSY NMR (400 MHz, $CDCl_3$) spectrum of **2b-6**.

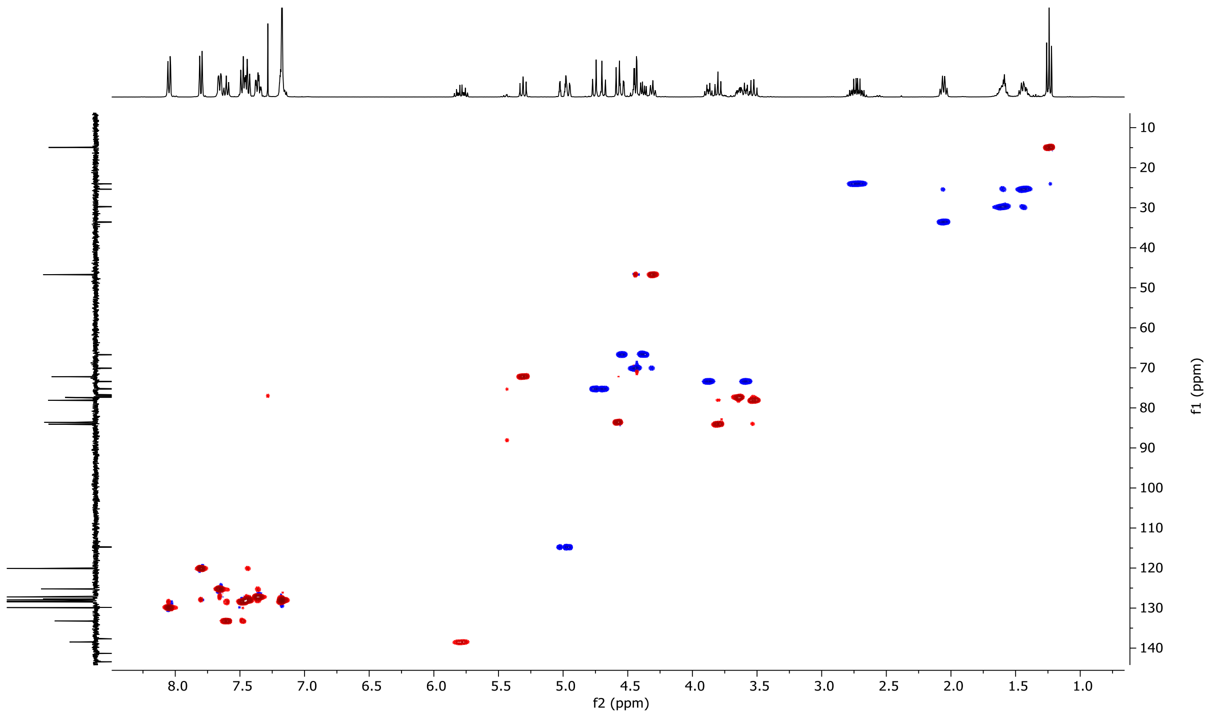

Figure S15. HSQC NMR (400 MHz, $CDCl_3$) spectrum of **2b-6**.

2.2. Synthesis of 3-O-alkenyl glucose BBs (3a-d)

*Ethyl 3-O-alkenyl-4,6-O-benzylidene-1-thio-β-D-glucopyranoside (**3-1**)*

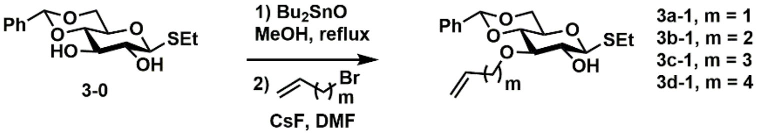

General procedure: Similar to a previous report,[7] **3-0** (2.0 g, 6.4 mmol) is dissolved in MeOH (80 mL), Bu_2SnO (2.0 g, 8.0 mmol) is added and the reaction mixture is refluxed for 12 h. The solvent is removed under reduced pressure and crude is re-dissolved in DMF (30 mL). Caesium fluoride (1.32 g, 8.64 mmol) and the corresponding alkenyl bromide (12.8 mmol) are added and the reaction was stirred at 60 °C overnight (for some examples the reaction is stirred longer times, as indicated). DMF is removed at reduced pressure, chloroform is added and the suspension is filtered through Celite. The filtrate is transferred to a separatory funnel and washed twice with brine. The organic layer is dried over Na_2SO_4 and evaporated at reduced pressure. The crude product is purified by column chromatography (hex/DCM/EA 6:1:1) to afford **3-1** as a white solid.

*Ethyl 3-O-allyl-4,6-O-benzylidene-1-thio-β-D-glucopyranoside (**3a-1**)*

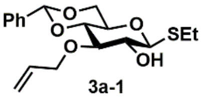

The tin-ketal obtained from **3-0** (2.0 g, 6.4 mmol) and Bu_2SnO (2.0 g, 8.0 mmol) is allowed to react with allyl bromide (1.1 ml, 12.8 mmol) and CsF (1.32 g, 8.64 mmol) in DMF (30 mL) at 60 °C overnight, according to the protocol detailed above. The crude product is purified by column chromatography (hex/DCM/EA 6:1:1) to afford **3a-1** (1.76 g, 78% yield) as a white solid. R*f* (hex/DCM/EA 3:1:1) = 0.40. ^{1}H NMR (400 MHz, $CDCl_3$), δ = 7.55 – 7.49 (m, 2H), 7.44 – 7.36 (m, 3H), 5.99 (ddt, *J* = 17.2, 10.4, 5.8 Hz, 1H), 5.58 (s, 1H), 5.34 (dq, *J* = 17.2, 1.7 Hz, 1H), 5.22 (dq, *J* = 10.3, 1.3 Hz, 1H), 4.51 (d, *J* = 7.2 Hz, 1H), 4.49 – 4.43 (m, 1H), 4.37 (dd, *J* = 10.5, 5.0 Hz, 1H), 4.32 (ddt, *J* = 12.7, 6.0, 1.4 Hz, 1H), 3.79 (t, *J* = 10.3 Hz, 1H), 3.72 – 3.48 (m, 4H), 2.87 – 2.70 (m, 2H), 2.62 (d, *J* = 2.0 Hz, 1H), 1.35 (t, *J* = 7.4 Hz, 3H). ^{13}C NMR (101 MHz, $CDCl_3$), δ = 137.21, 134.82, 129.02, 128.28, 125.99, 117.50, 101.24, 86.59, 81.30, 81.26, 73.70, 72.85, 70.76, 68.65, 24.61, 15.27.

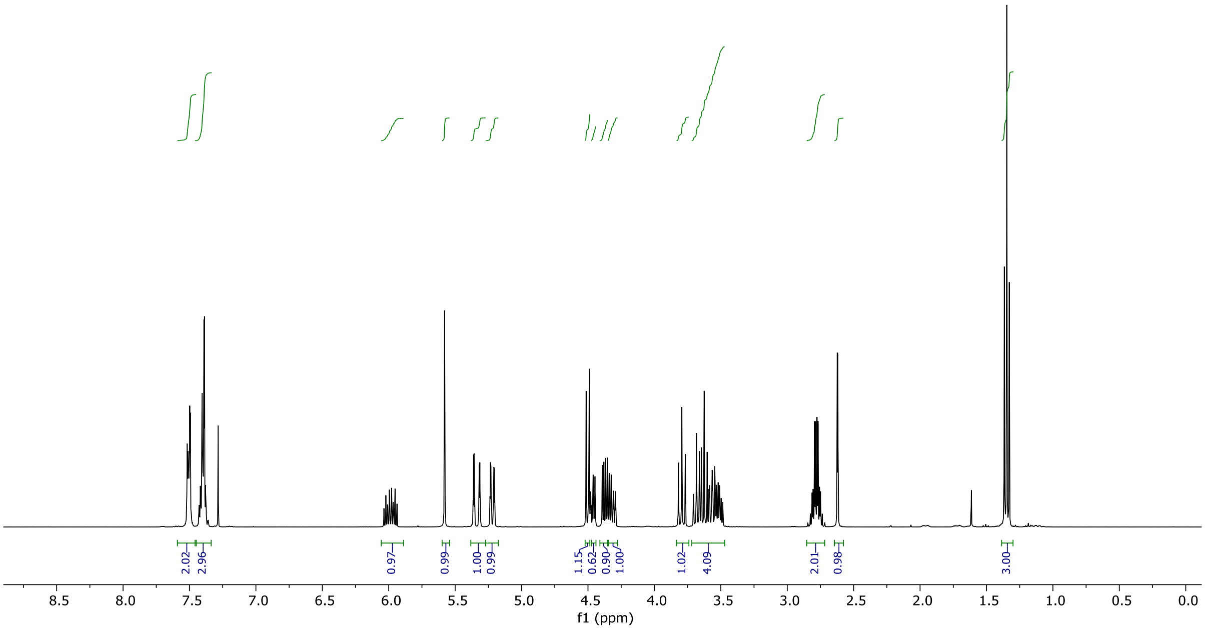

Figure S16. ^{1}H NMR (400 MHz, $CDCl_3$) spectrum of **3a-1**.

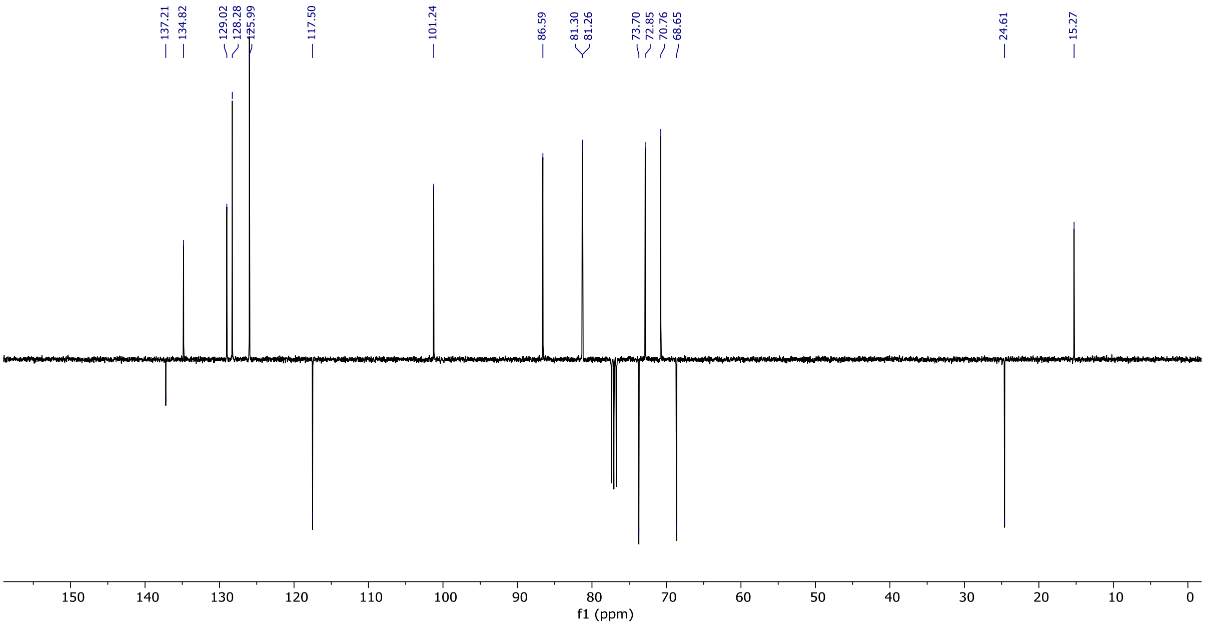

Figure S17. ^{13}C APT NMR (101 MHz, $CDCl_3$) spectrum of **3a-1**.

*Ethyl 4,6-O-benzylidene-3-O-(but-3-en-1-yl)-1-thio-β-D-glucopyranoside (**3b-1**)*

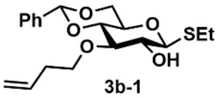

3b-1

The tin-ketal obtained from **3-0** (2.0 g, 6.4 mmol) and Bu_2SnO (2.0 g, 8.0 mmol) is allowed to react with 4-bromo-1-buten (1.3 mL, 12.8 mmol) and CsF (1.32 g, 8.64 mmol) in DMF (30 mL) at 60 °C for 24 h. Then, additional CsF (1.32 g, 8.64 mmol) and 4-bromo-1-butene (1.3 mL, 12.8 mmol) are added and the reaction is stirred for another 24 h. The crude product treated according to the protocol detailed above and purified

by column chromatography (hex/DCM/EA 6:1:1) to afford **3b-1** (0.98 g, 42% yield) as a white solid. R*f* (hex/DCM/EA 3:1:1) = 0.41. ^{1}H NMR (400 MHz, $CDCl_3$), δ = 7.53 – 7.48 (m, 2H), 7.43 – 7.38 (m, 3H), 5.87 (ddt, *J* = 17.0, 10.3, 6.7 Hz, 1H), 5.58 (s, 1H), 5.19 – 5.12 (m, 1H), 5.08 (ddt, *J* = 10.2, 2.2, 1.3 Hz, 1H), 4.54 – 4.48 (m, 1H), 4.37 (dd, *J* = 10.5, 5.0 Hz, 1H), 4.03 (dt, *J* = 9.8, 6.4 Hz, 1H), 3.87 – 3.75 (m, 2H), 3.69 – 3.62 (m, 1H), 3.58 – 3.47 (m, 3H), 2.78 (qd, *J* = 7.4, 3.0 Hz, 2H), 2.45 – 2.34 (m, 2H), 1.35 (t, *J* = 7.5 Hz, 3H). ^{13}C NMR (101 MHz, $CDCl_3$), δ = 137.21, 135.53, 129.00, 128.27, 125.97, 116.79, 101.17, 86.34, 82.52, 81.19, 72.82, 72.03, 70.81, 68.65, 34.56, 24.57, 15.22.

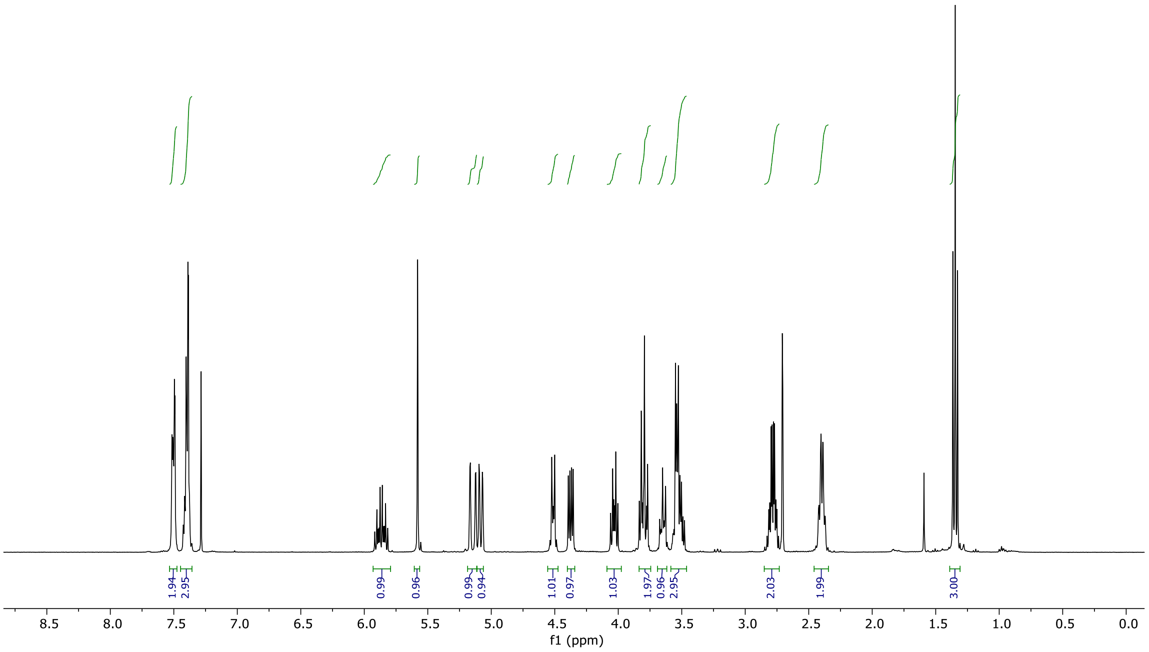

Figure S18. ^{1}H NMR (400 MHz, $CDCl_3$) spectrum of **3b-1**.

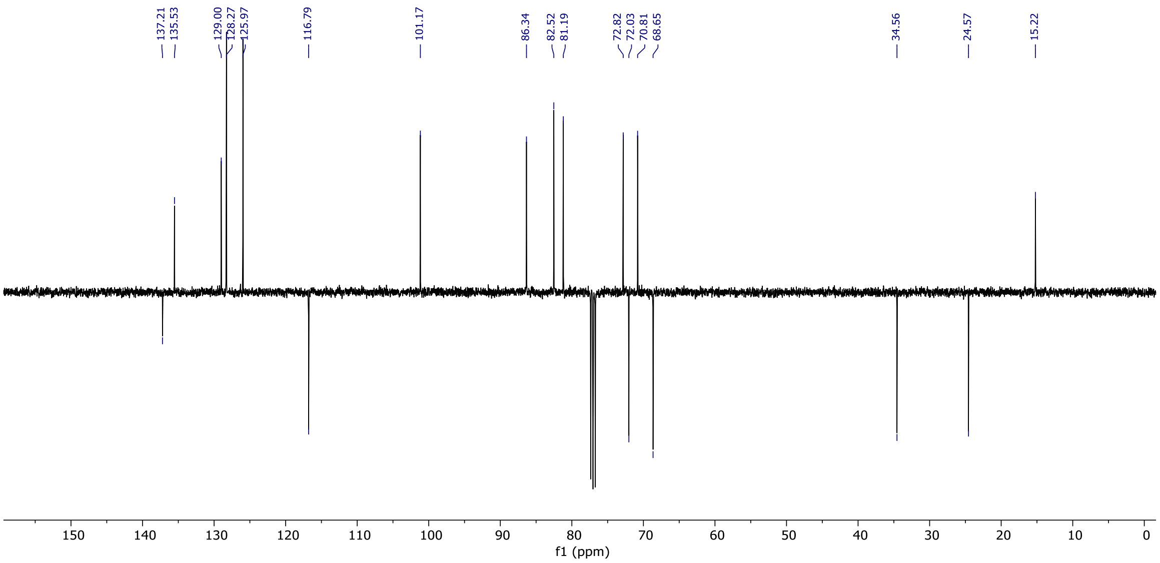

Figure S19. ^{13}C APT NMR (101 MHz, $CDCl_3$) spectrum of **3b-1**.

*Ethyl 4,6-O-benzylidene-3-O-(pent-4-en-1-yl)-1-thio-β-D-glucopyranoside (**3c-1**)*

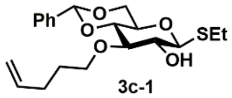

3c-1

The tin-ketal obtained from **3-0** (2.0 g, 6.4 mmol) and Bu_2SnO (2.0 g, 8.0 mmol) is allowed to react with 5-bromo-1-penten (1.5 mL, 12.8 mmol) and CsF (1.32 g, 8.64 mmol) in DMF (30 mL) at 60 °C for 24 h. Then, additional CsF (1.32 g, 8.64 mmol) and 5-bromo-1-pentene (1.5 mL, 12.8 mmol) are added and the reaction is stirred for another 24 h. The crude product treated according to the protocol detailed above and purified by column chromatography (hex/DCM/EA 6:1:1) to afford **3c-1** (1.56 g, 64% yield) as a white solid. R*f* (hex/ EA 3:1) = 0.50. ^{1}H NMR (400 MHz, $CDCl_3$), δ = 7.53 – 7.47 (m, 2H), 7.45 – 7.36 (m, 3H), 5.82 (ddt, *J* = 16.9, 10.2, 6.6 Hz, 1H), 5.58 (s, 1H), 5.02 (dq, *J* = 17.1, 1.7 Hz, 1H), 4.96 (ddt, *J* = 10.2, 2.2, 1.3 Hz, 1H), 4.54 – 4.47 (m, 1H), 4.37 (dd, *J* = 10.5, 4.9 Hz, 1H), 3.96 (dt, *J* = 9.5, 6.4 Hz, 1H), 3.84 – 3.73 (m, 2H), 3.69 – 3.60 (m, 1H), 3.57 – 3.46 (m, 3H), 2.78 (qd, *J* = 7.4, 3.0 Hz, 2H), 2.62 (d, *J* = 1.6 Hz, 1H), 2.21 – 2.13 (m, 2H), 1.79 – 1.69 (m, 2H), 1.35 (t, *J* = 7.5 Hz, 3H). ^{13}C NMR (101 MHz, $CDCl_3$), δ = 138.41, 137.22, 128.98, 128.25, 125.99, 114.77, 101.18, 86.54, 82.24, 81.24, 72.91, 72.49, 70.82, 68.66, 30.26, 29.30, 24.60, 15.26.

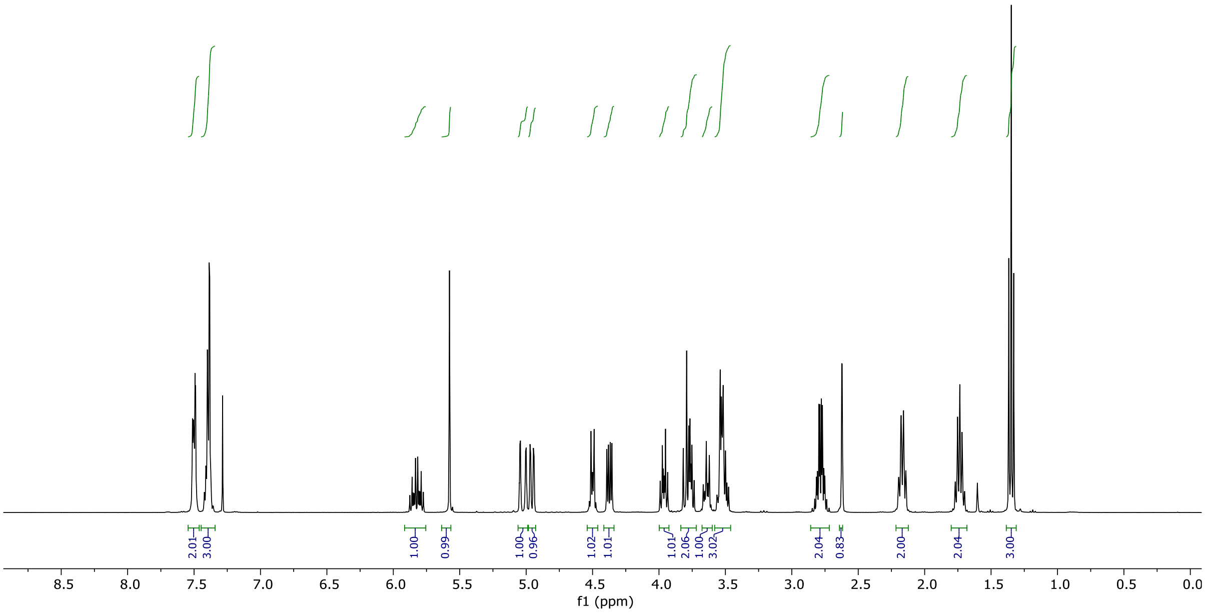

Figure S20. ^{1}H NMR(101 MHz, $CDCl_3$) spectrum of **3c-1**.

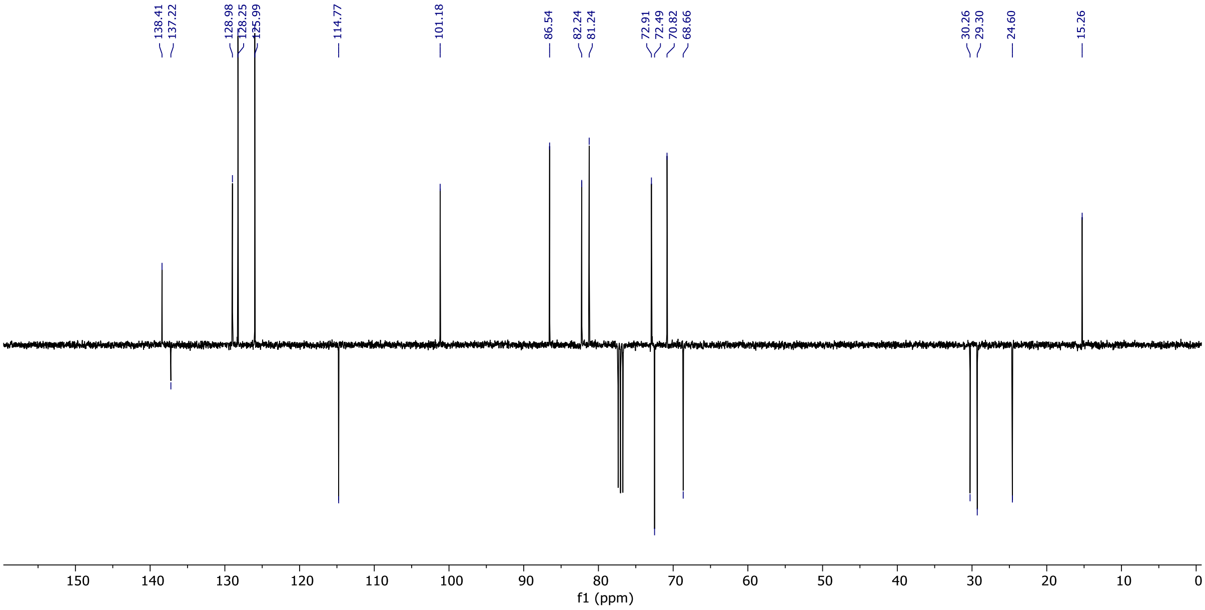

Figure S21. ^{13}C APT NMR (101 MHz, $CDCl_3$) spectrum of **3c-1**.

*Ethyl 4,6-O-benzylidene-3-O-(hex-5-en-1-yl)-1-thio-β-D-glucopyranoside (**3d-1**)*

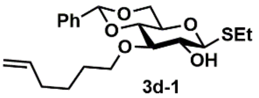

3d-1

The tin-ketal obtained from **3-0** (2.0 g, 6.4 mmol) and Bu_2SnO (2.0 g, 8.0 mmol) is allowed to react with 6-bromo-1-hexen (1.7 ml, 12.8 mmol) and CsF (1.32 g, 8.64 mmol) in DMF (30 mL) at 60 °C overnight, according to the protocol detailed above. The crude product is purified by column chromatography (hex/DCM/EA 6:1:1) to afford **3d-1** (1.81 g, 72% yield) as a white solid. R*f* (hex/ EA 3:1) = 0.52. ^{1}H NMR (400 MHz, $CDCl_3$), δ = 7.54 – 7.47 (m, 2H), 7.43 – 7.37 (m, 3H), 5.79 (ddt, *J* = 16.9, 10.2, 6.7 Hz, 1H), 5.58 (s, 1H), 5.03 – 4.97 (m, 1H), 4.97 – 4.93 (m, 1H), 4.55 – 4.46 (m, 1H), 4.37 (dd, *J* = 10.5, 4.9 Hz, 1H), 3.98 – 3.90 (m, 1H), 3.83 – 3.73 (m, 2H), 3.69 – 3.59 (m, 1H), 3.56 – 3.47 (m, 3H), 2.78 (qd, *J* = 7.5, 3.3 Hz, 2H), 2.61 – 2.57 (m, 1H), 2.07 (tdt, *J* = 7.9, 6.6, 1.4 Hz, 2H), 1.71 – 1.57 (m, 3H), 1.54 – 1.44 (m, 2H), 1.35 (t, *J* = 7.4 Hz, 3H). ^{13}C NMR (101 MHz, $CDCl_3$), δ = 138.74, 137.24, 128.99, 128.26, 125.98, 114.53, 101.20, 86.57, 82.21, 81.22, 73.03, 72.95, 70.84, 68.66, 33.49, 29.66, 25.32, 24.59, 15.27.

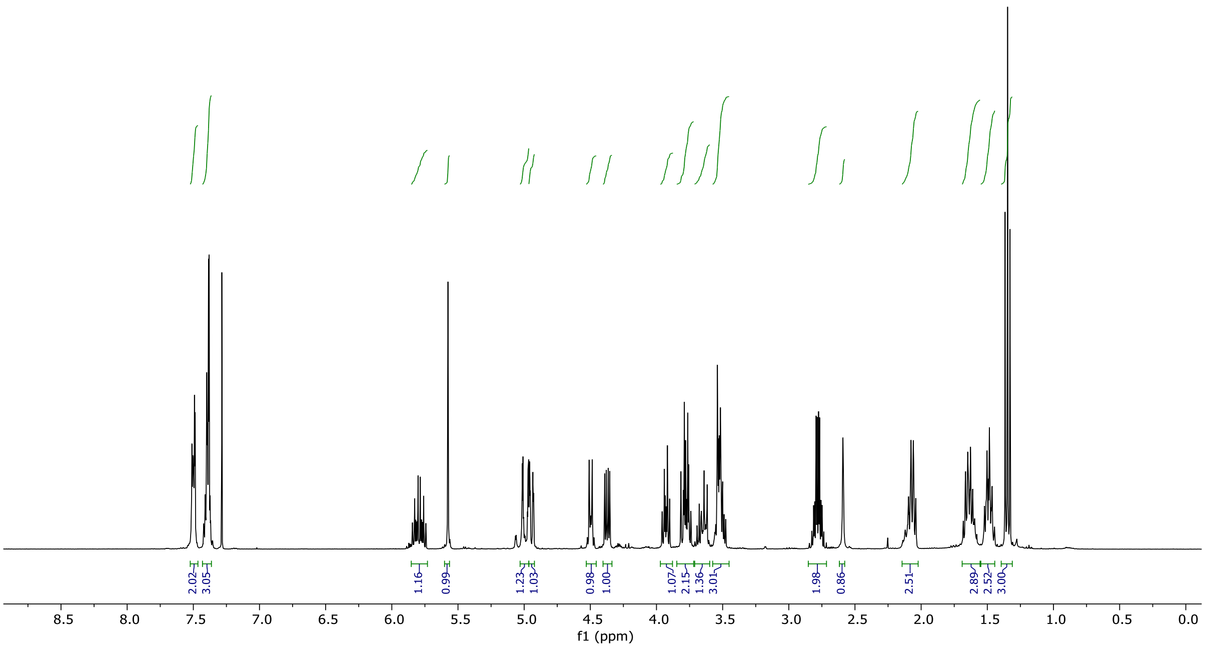

Figure S22. ^{1}H NMR (101 MHz, $CDCl_3$) spectrum of **3d-1**.

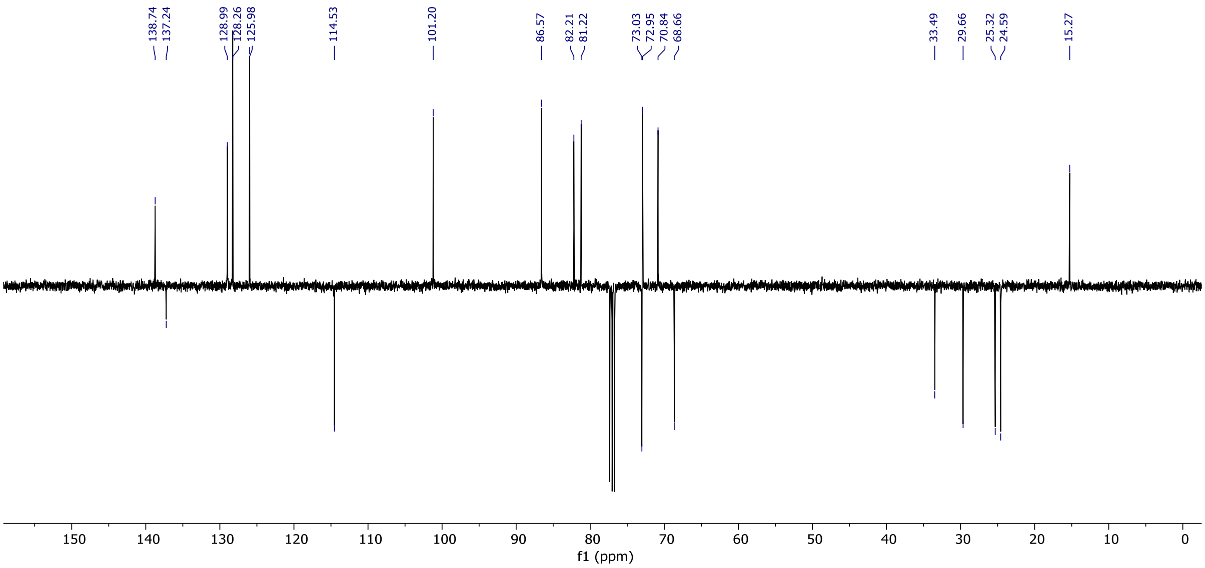

Figure S23. ^{13}C APT NMR (101 MHz, $CDCl_3$) spectrum of **3d-1**.

*Ethyl 3-O-alkenyl-2-O-benzoyl-4,6-O-benzylidene-1-thio-β-D-glucopyranoside (**3-2**)*

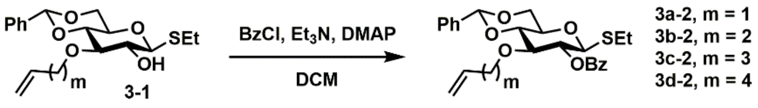

General procedure: Similar to a previous report,[7] **3-1** (5.0 mmol) is dissolved in DCM (40 mL). Et_3N (3.5 mL, 25.0 mmol) and DMAP (61 mg, 0.5 mmol) are added and the mixture is placed in an ice bath. At 0 °C, BzCl (0.87 mL, 7.5 mmol) is added dropwise and the reaction is stirred initially for 30 min, and then it is left to reach room temperature and stirred for an

additional 12 h. The reaction mixture is diluted with chloroform (50 mL), transferred to a separatory funnel, and washed with a 10% soln. of citric acid (2×20 mL) and brine (2×20 mL). The organic layer is dried over Na_2SO_4 and evaporated at reduced pressure. The crude product is purified by column chromatography (hex/EA 10:1) to afford **3-2** (87-92% yield) as a white solid.

*Ethyl 3-O-allyl-2-O-benzoyl-4,6-O-benzylidene-1-thio-β-D-glucopyranoside (**3a-2**)*

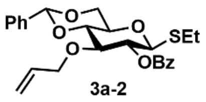

3a-1 (1.81 g, 5.1 mmol), Et_3N (3.57 mL, 25.5 mmol), DMAP (62 mg, 0.51 mmol) and BzCl (0.89 mL, 7.65 mmol) are allowed to react in DCM (40 mL) according to the general procedure detailed above to afford **3a-2** (2.1 g, 87% yield) as a white solid. R*f* (hex/ EA 5:1) = 0.50. ^{1}H NMR (400 MHz, $CDCl_3$), δ = 8.15 – 8.09 (m, 2H), 7.66 – 7.59 (m, 1H), 7.56 – 7.47 (m, 4H), 7.44 – 7.37 (m, 3H), 5.73 (dddd, *J* = 16.8, 10.3, 6.2, 5.2 Hz, 1H), 5.62 (s, 1H), 5.32 (dd, *J* = 10.0, 8.6 Hz, 1H), 5.21 – 5.14 (m, 1H), 5.07 – 5.01 (m, 1H), 4.68 (d, *J* = 10.1 Hz, 1H), 4.43 (dd, *J* = 10.5, 5.0 Hz, 1H), 4.34 (ddt, *J* = 12.9, 5.2, 1.4 Hz, 1H), 4.14 (ddt, *J* = 13.0, 6.2, 1.4 Hz, 1H), 3.92 – 3.77 (m, 3H), 3.59 (td, *J* = 9.6, 5.0 Hz, 1H), 2.76 (qd, *J* = 7.5, 3.1 Hz, 2H), 1.26 (t, *J* = 7.4 Hz, 3H). ^{13}C NMR (101 MHz, $CDCl_3$), δ = 165.18, 137.18, 134.64, 133.24, 129.87, 129.82, 129.04, 128.45, 128.29, 126.01, 117.21, 101.24, 84.34, 81.34, 79.70, 73.51, 71.97, 70.84, 68.65, 24.06, 14.83.

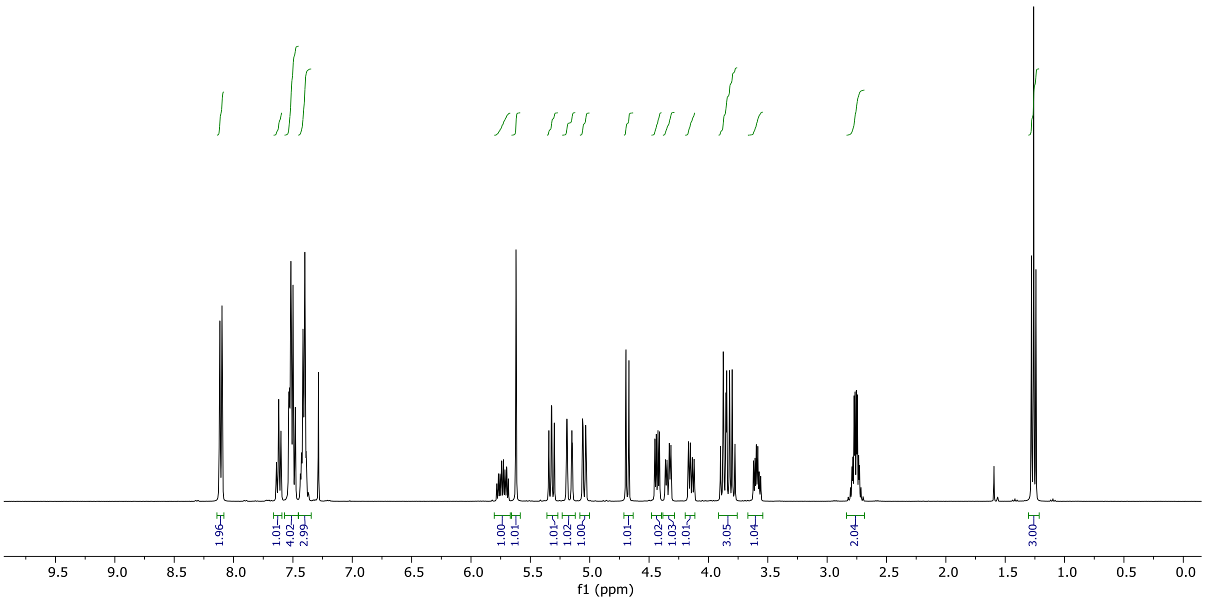

Figure S24. ^{1}H NMR (101 MHz, $CDCl_3$) spectrum of **3a-2**.

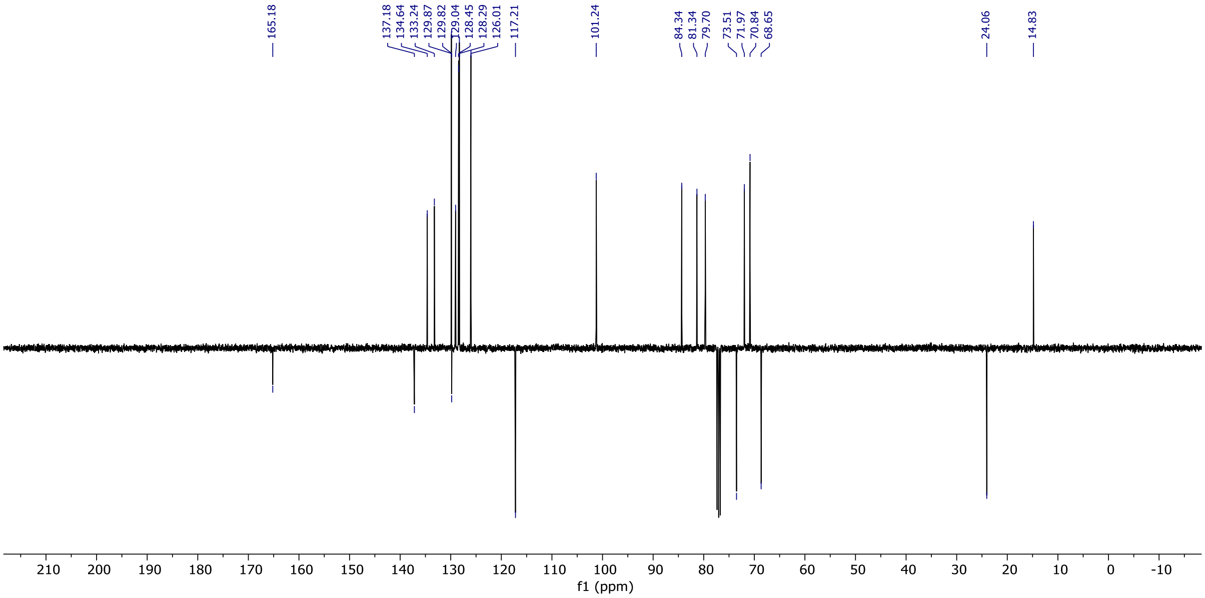

Figure S25. ^{13}C APT NMR (101 MHz, $CDCl_3$) spectrum of **3a-2**.

*Ethyl 2-O-benzoyl-4,6-O-benzylidene-3-O-(but-3-en-1-yl)-1-thio-β-D-glucopyranoside (**3b-1**)*

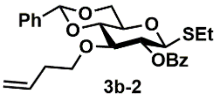

3b-1 (1.77 g, 4.8 mmol), Et_3N (3.36 mL, 24.0 mmol), DMAP (58 mg, 0.48 mmol) and BzCl (0.83 mL, 7.2 mmol) are allowed to react in DCM (40 mL) according to the general procedure detailed above to afford **3b-2** (2.1 g, 92% yield) as a white solid. R*f* (hex/ EA 5:1) = 0.52. ^{1}H NMR (400 MHz, $CDCl_3$), δ = 8.13 – 8.08 (m, 2H), 7.65 – 7.59 (m, 1H), 7.54 – 7.46 (m, 4H), 7.44 – 7.37 (m, 3H), 5.70 – 5.57 (m, 2H), 5.34 – 5.27 (m, 1H), 4.89 (dq, *J* = 17.2, 1.6 Hz, 1H), 4.76 (ddt, *J* = 10.3, 2.2, 1.2 Hz, 1H), 4.68 (d, *J* = 10.1 Hz, 1H), 4.43 (dd, *J* = 10.5, 4.9 Hz, 1H), 3.96 – 3.89 (m, 1H), 3.88 – 3.75 (m, 3H), 3.64 – 3.55 (m, 2H), 2.76 (qd, *J* = 7.5, 3.1 Hz, 2H), 2.20 (pt, *J* = 6.4, 1.4 Hz, 2H), 1.26 (t, *J* = 7.5 Hz, 3H). ^{13}C NMR (101 MHz, $CDCl_3$), δ = 165.18, 137.18, 134.99, 133.21, 129.89, 129.84, 129.76, 129.03, 128.51, 128.44, 128.28, 128.18, 126.00, 116.20, 101.20, 84.33, 81.20, 72.58, 71.95, 70.90, 68.65, 34.48, 24.01, 14.83.

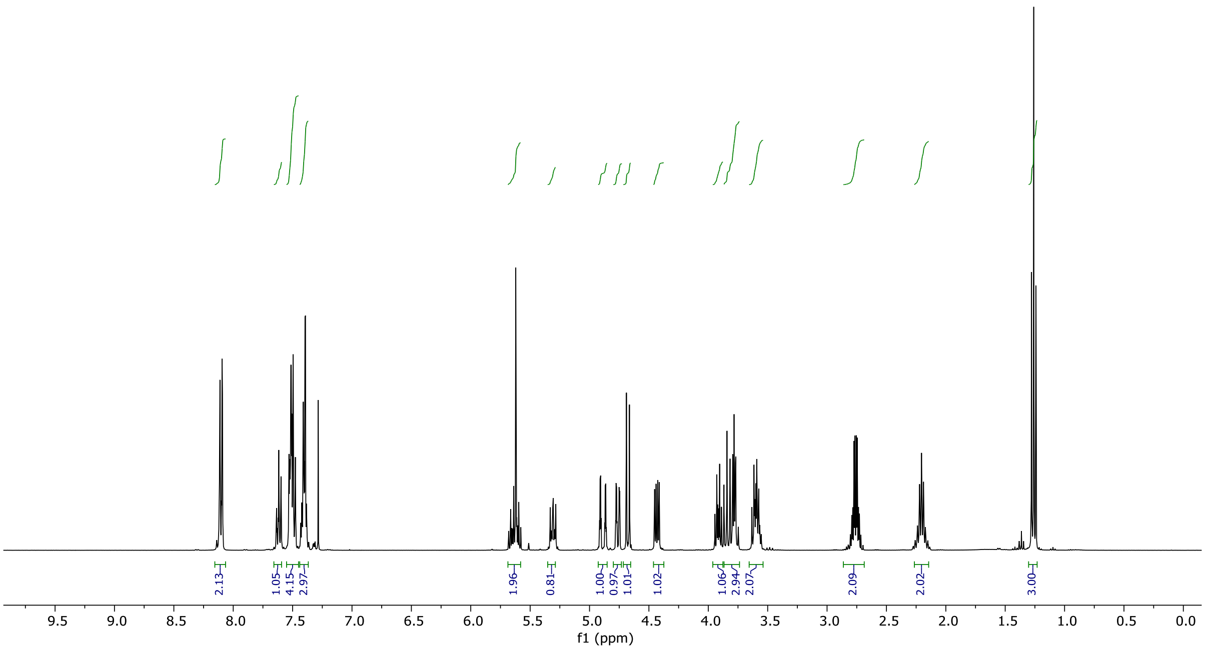

Figure S26. ^{1}H NMR (101 MHz, $CDCl_3$) spectrum of **3b-2**.

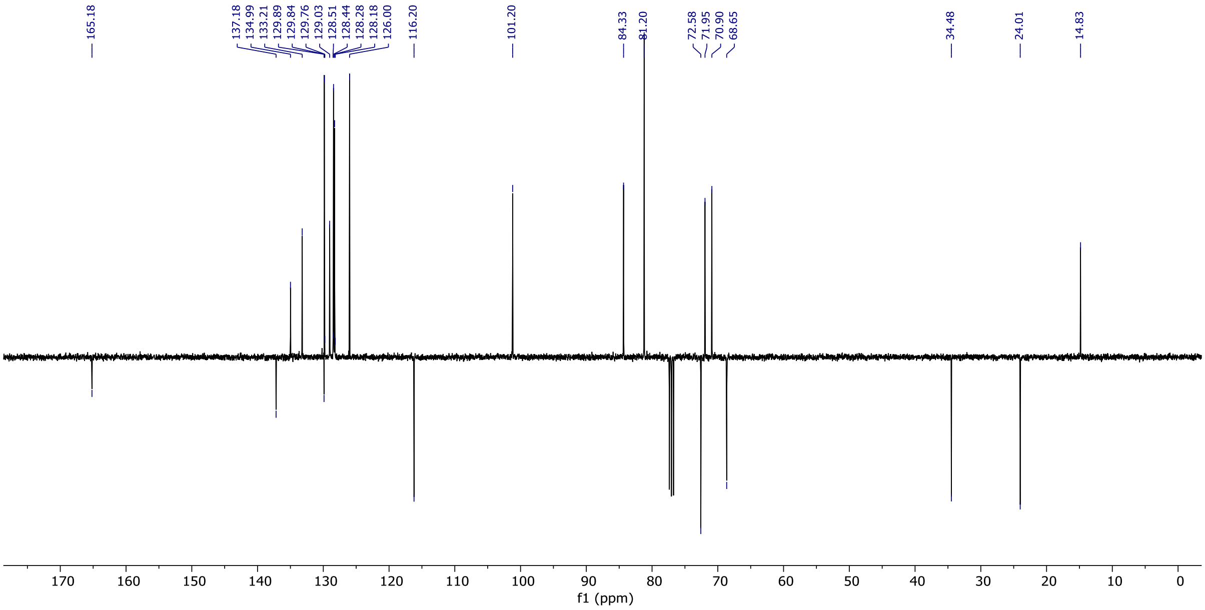

Figure S27. ^{13}C APT NMR (101 MHz, $CDCl_3$) spectrum of **3b-2**.

*Ethyl 2-O-benzoyl-4,6-O-benzylidene-3-O-(pent-4-en-1-yl)-1-thio-β-D-glucopyranoside(**3c-1**)*

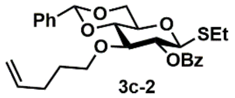

3c-2

3c-1 (0.9 g, 2.37 mmol), Et_3N (1.7 mL, 11.9 mmol), DMAP (29 mg, 0.24 mmol) and BzCl (0.41 mL, 3.6 mmol) are allowed to react in DCM (20 mL) according to the general procedure detailed above to afford **3c-2** (1.05 g, 91% yield) as a white solid. R*f* (hex/ EA 6:1) = 0.40. ^{1}H NMR (400 MHz, $CDCl_3$), δ = 8.14 – 8.07 (m, 2H), 7.67 – 7.58 (m, 1H), 7.55 – 7.46 (m, 4H), 7.43 – 7.37 (m, 3H), 5.65 – 5.55 (m, 2H), 5.34 – 5.26 (m, 1H), 4.84 – 4.78 (m, 1H), 4.76 (t, *J* = 1.4 Hz, 1H), 4.68 (d, *J* = 10.0 Hz, 1H), 4.43 (dd, *J* =

10.5, 4.9 Hz, 1H), 3.90 – 3.81 (m, 2H), 3.79 – 3.73 (m, 2H), 3.63 – 3.53 (m, 2H), 2.76 (qd, *J* = 7.4, 3.5 Hz, 2H), 2.05 – 1.84 (m, 2H), 1.61 – 1.49 (m, 3H), 1.26 (t, *J* = 7.4 Hz, 3H). ^{13}C NMR (101 MHz, $CDCl_3$), δ = 165.21, 138.10, 137.18, 133.23, 129.85, 129.01, 128.46, 128.27, 126.01, 114.45, 101.21, 84.33, 81.24, 80.99, 72.40, 72.08, 70.91, 68.65, 29.87, 29.18, 24.02, 14.83.

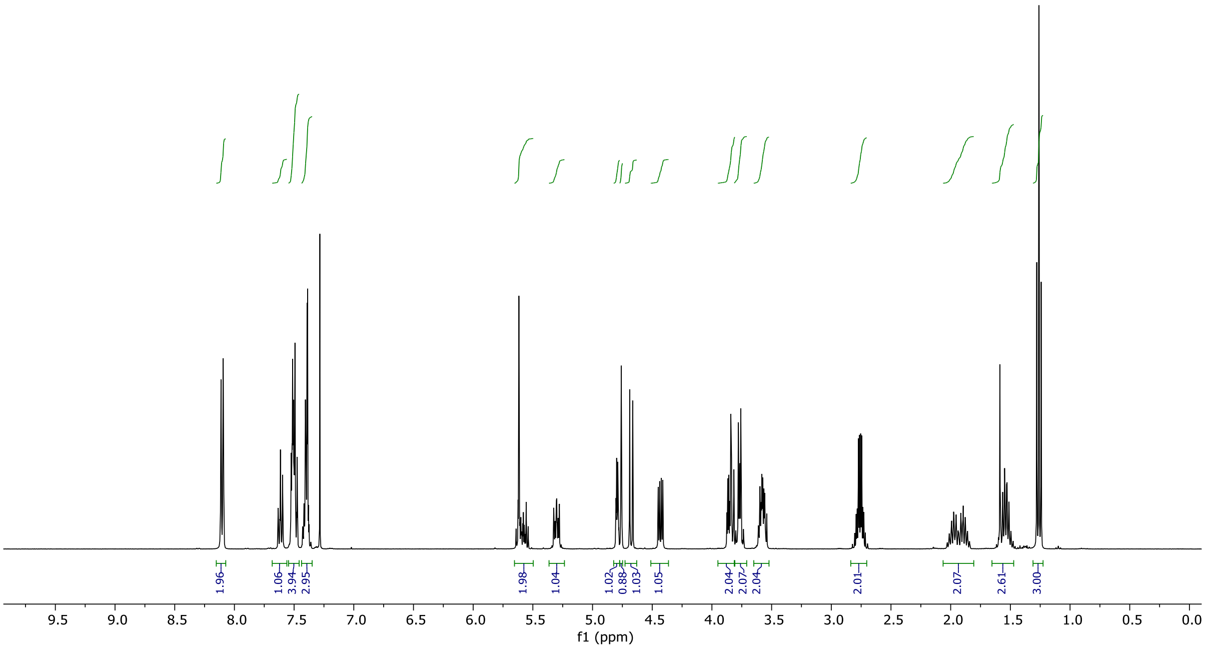

Figure S28. ^{1}H NMR (101 MHz, $CDCl_3$) spectrum of **3c-2**.

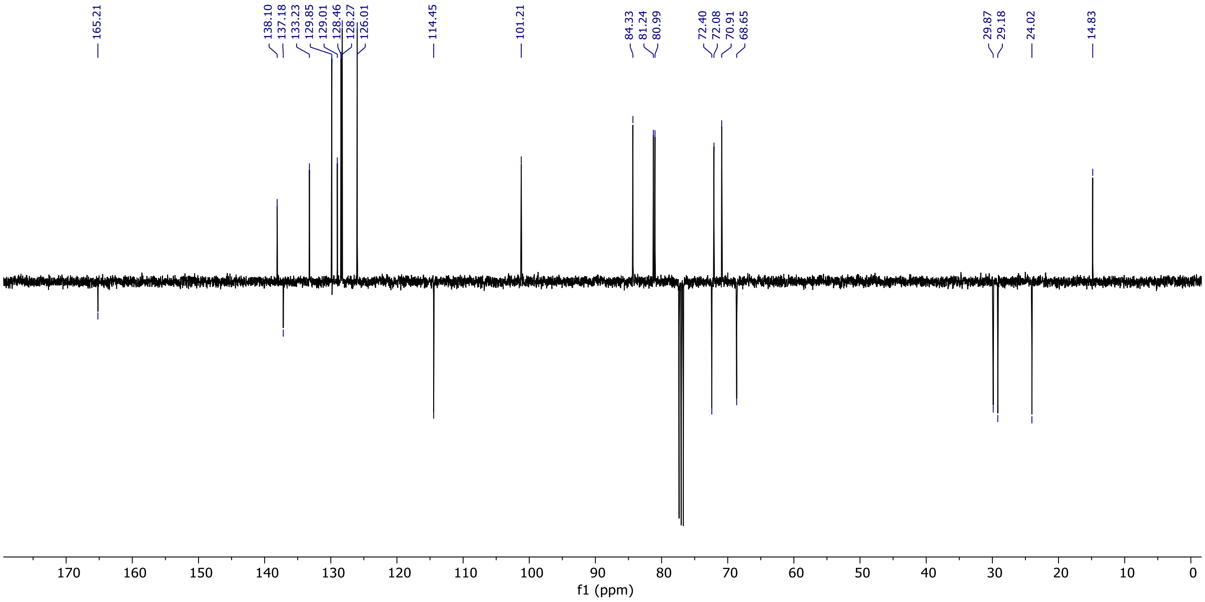

Figure S29. ^{13}C APT NMR (101 MHz, $CDCl_3$) spectrum of **3c-2**.

Ethyl 2-O-benzoyl-4,6-O-benzylidene-3-O-(hex-5-en-1-yl)-1-thio-β-D-glucopyranoside (**3c-1**)

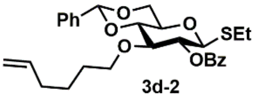

3d-2

3d-1 (2.1 g, 5.33 mmol), Et_3N (3.7 mL, 26.7 mmol), DMAP (65 mg, 0.53 mmol) and BzCl (0.93 mL, 8.0 mmol) are allowed to react in DCM (40 mL) according to the general procedure detailed above to afford **3d-2** (2.45 g, 92% yield) as a white solid. R*f* (hex/ EA 5:1) = 0.52. ^{1}H NMR (400 MHz, $CDCl_3$), δ = 8.13 – 8.08 (m, 2H), 7.64 – 7.59 (m, 1H), 7.55 – 7.47 (m, 4H), 7.44 – 7.37 (m, 3H), 5.61 (s, 1H), 5.60 – 5.49 (m, 1H), 5.33 – 5.27 (m, 1H), 4.86 – 4.81 (m, 1H), 4.79 (t, *J* = 1.4 Hz, 1H), 4.68 (d, *J* = 10.0 Hz, 1H), 4.43 (dd, *J* = 10.5, 5.0 Hz, 1H), 3.84 (ddd, *J* = 10.4, 7.3, 2.5 Hz, 2H), 3.79 – 3.73 (m, 2H), 3.64 – 3.49 (m, 2H), 2.76 (qd, *J* = 7.5, 3.4 Hz, 2H), 1.83 (tdq, *J* = 9.1, 6.7, 1.5 Hz, 2H), 1.52 – 1.40 (m, 2H), 1.36 – 1.30 (m, 1H), 1.26 (t, *J* = 7.5 Hz, 3H), 1.22 – 1.15 (m, 1H). ^{13}C NMR (101 MHz, $CDCl_3$), δ = 165.18, 138.62, 137.20, 133.22, 129.85, 129.01, 128.46, 128.41, 128.27, 126.00, 114.23, 101.21, 84.33, 81.26, 81.02, 73.02, 72.06, 70.92, 68.65, 33.30, 29.52, 25.14, 24.01, 14.83.

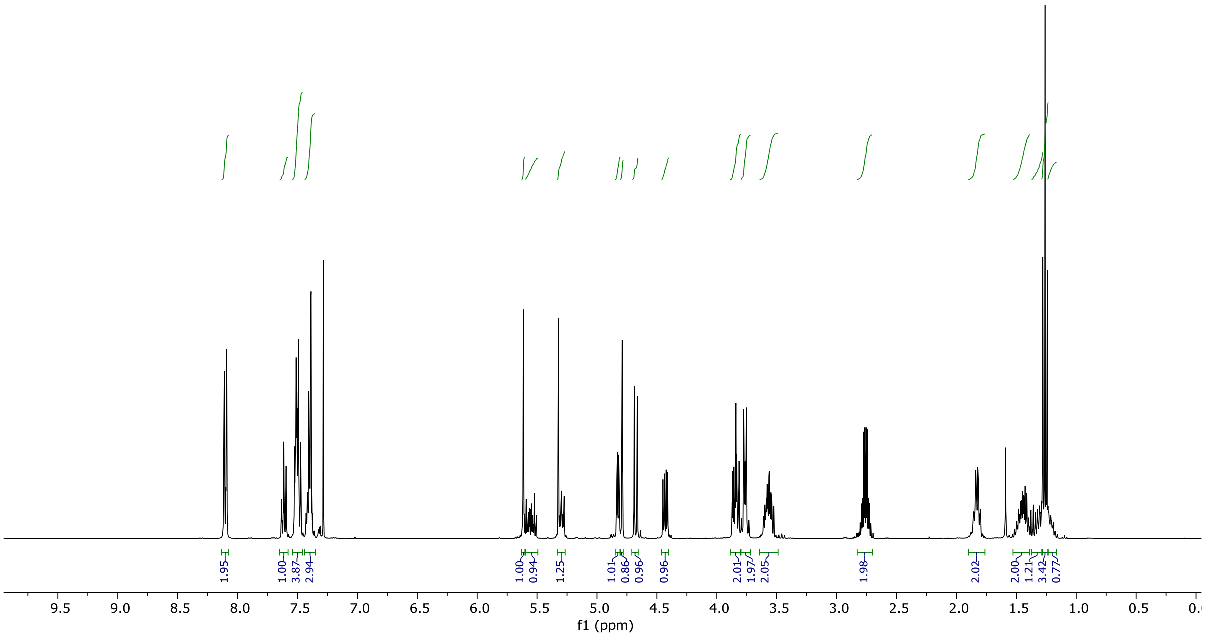

Figure S30. ^{13}C APT NMR (101 MHz, $CDCl_3$) spectrum of **3d-2**.

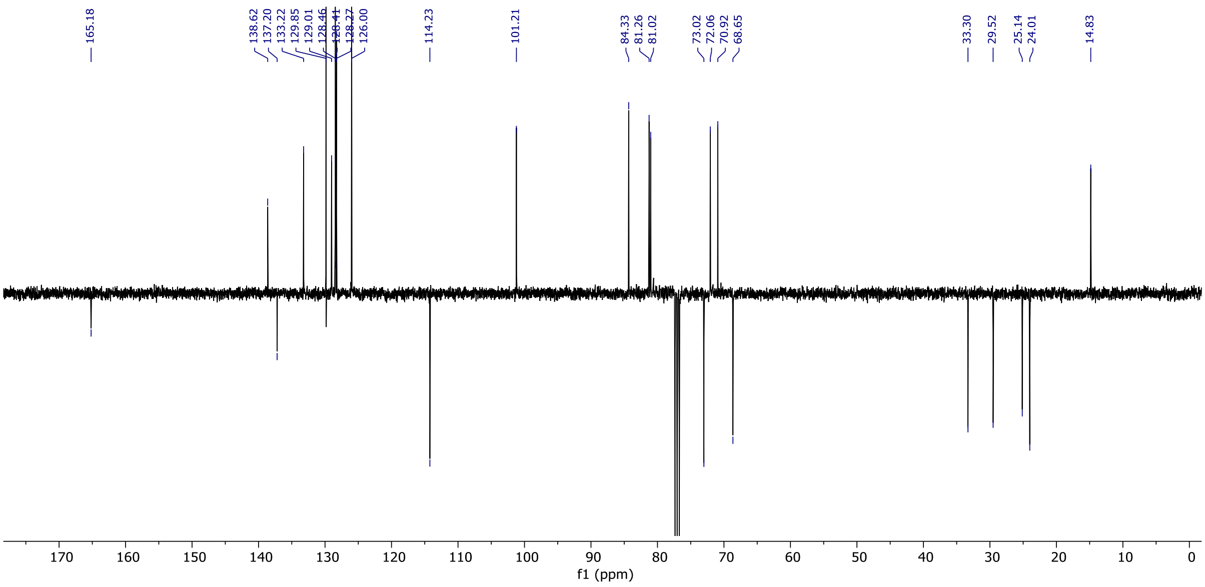

Figure S31. ^{13}C APT NMR (101 MHz, $CDCl_3$) spectrum of **3d-2**.

*Ethyl 3-O-alkenyl-2-O-benzoyl-1-thio-β-D-glucopyranoside (**3-3**)*

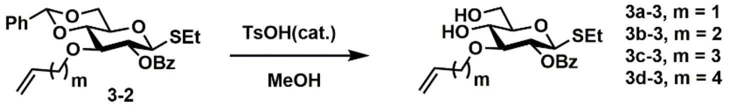

General procedure:[5] **3-2** (5.0 mmol) is suspended in MeOH (40 mL), and in the presence of TsOH (130 mg, 0.75 mmol) the reaction mixture is subjected to an ultrasonic bath for 30 min. The clear solution is quenched with Et_3N (1 mL) and the volatiles are evaporated under reduced pressure. The crude product is purified by column chromatography (DCM/MeOH 30:1) to afford **3-3** (87-92% yield) as a pale yellow syrup.

*Ethyl 3-O-allyl-2-O-benzoyl-1-thio-β-D-glucopyranoside (**3a-3**)*

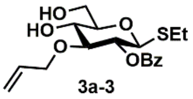

3a-2 (2.0 g, 4.39 mmol) and TsOH (114 mg, 0.66 mmol) are allowed to react in MeOH (40 mL) according to the general procedure detailed above to afford **3a-3** (1.47 g, 91% yield) as a white solid. R*f* (DCM/ MeOH 20:1) = 0.61. ^{1}H NMR (400 MHz, DMSO-*d*$_6$), δ = 8.04 – 7.96 (m, 2H), 7.73 – 7.63 (m, 1H), 7.56 (t, *J* = 7.7 Hz, 2H), 5.68 (ddt, *J* = 17.3, 10.6, 5.4 Hz, 1H), 5.47 (d, *J* = 5.9 Hz, 1H), 5.05 (dq, *J* = 17.3, 1.8 Hz, 1H), 4.93 (q, *J* = 1.6 Hz, 1H), 4.89 – 4.84 (m, 1H), 4.72 (d, *J* = 10.1 Hz, 1H), 4.65 (t, *J* = 5.8 Hz, 1H), 4.25 (ddt, *J* = 13.1, 5.2, 1.5 Hz, 1H), 4.05 (ddt, *J* = 13.2, 5.7, 1.6 Hz, 1H), 3.72 (ddd, *J* = 11.9, 5.7, 1.8 Hz, 1H), 3.58 (t, *J* = 8.8 Hz, 1H), 3.50 (dt, *J* = 11.7, 5.7 Hz, 1H), 3.44 – 3.37 (m, 1H), 2.74 – 2.58 (m, 2H), 1.14 (t, *J* = 7.4 Hz, 3H). ^{13}C NMR (101 MHz, DMSO-*d*$_6$), δ = 165.21, 135.98, 133.98, 130.03, 129.75, 129.28, 116.32, 83.79, 82.68, 81.57, 73.34, 72.63, 70.23, 61.18, 23.74, 15.29.

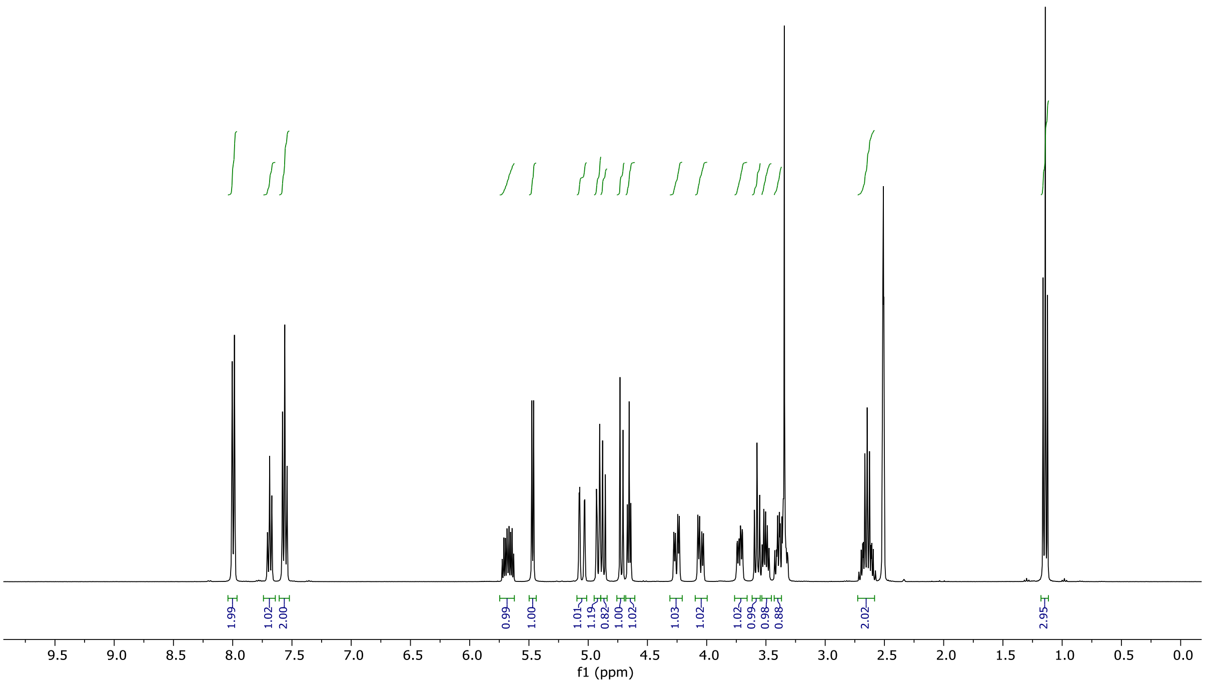

Figure S32. ^{1}H NMR (101 MHz, $CDCl_3$) spectrum of **3a-3**.

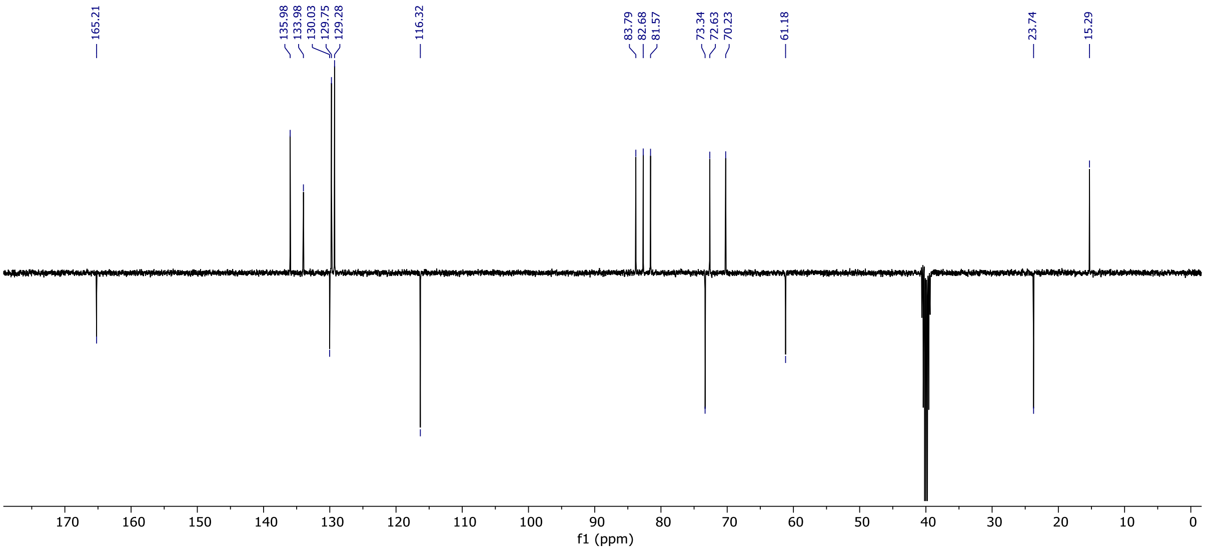

Figure S33. ^{13}C APT NMR (101 MHz, $CDCl_3$) spectrum of **3a-3**.

*Ethyl 2-O-benzoyl-3-O-(but-3-en-1-yl)-1-thio-β-D-glucopyranoside (**3b-3**)*

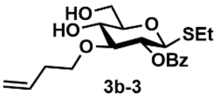

3b-3

3b-2 (2.1 g, 4.47 mmol) and TsOH (116 mg, 0.67 mmol) are allowed to react in MeOH (40 mL) according to the general procedure detailed above to afford **3b-3** (1.52 g, 89% yield) as a white solid. R*f* (DCM/ MeOH 20:1) = 0.65. ^{1}H NMR (400 MHz, $CDCl_3$), δ = 8.12 – 8.06 (m, 2H), 7.62 (ddt, *J* = 8.0, 6.9, 1.3 Hz, 1H), 7.53 – 7.46 (m, 2H), 5.71 (ddt, *J* = 17.0, 10.2, 6.8 Hz, 1H), 5.21 (dd, *J* = 10.0, 9.1 Hz, 1H), 5.06 – 4.96 (m, 2H), 4.61 (d, *J* = 10.0 Hz, 1H), 3.98 (ddd, *J* = 11.9, 6.2, 3.4 Hz, 1H), 3.90 – 3.77 (m, 2H), 3.72 – 3.62 (m, 2H), 3.55 – 3.45 (m, 2H), 2.72 (qd, *J* = 7.5, 3.3 Hz, 2H), 2.24 (qt, *J* = 6.3, 1.4 Hz,

2H), 1.25 (t, J = 7.5 Hz, 3H). ^{13}C NMR (101 MHz, $CDCl_3$), δ = 165.21, 135.41, 133.35, 129.83, 129.74, 128.53, 117.19, 84.90, 83.87, 79.49, 72.28, 71.92, 70.43, 62.76, 34.66, 24.19, 14.88.

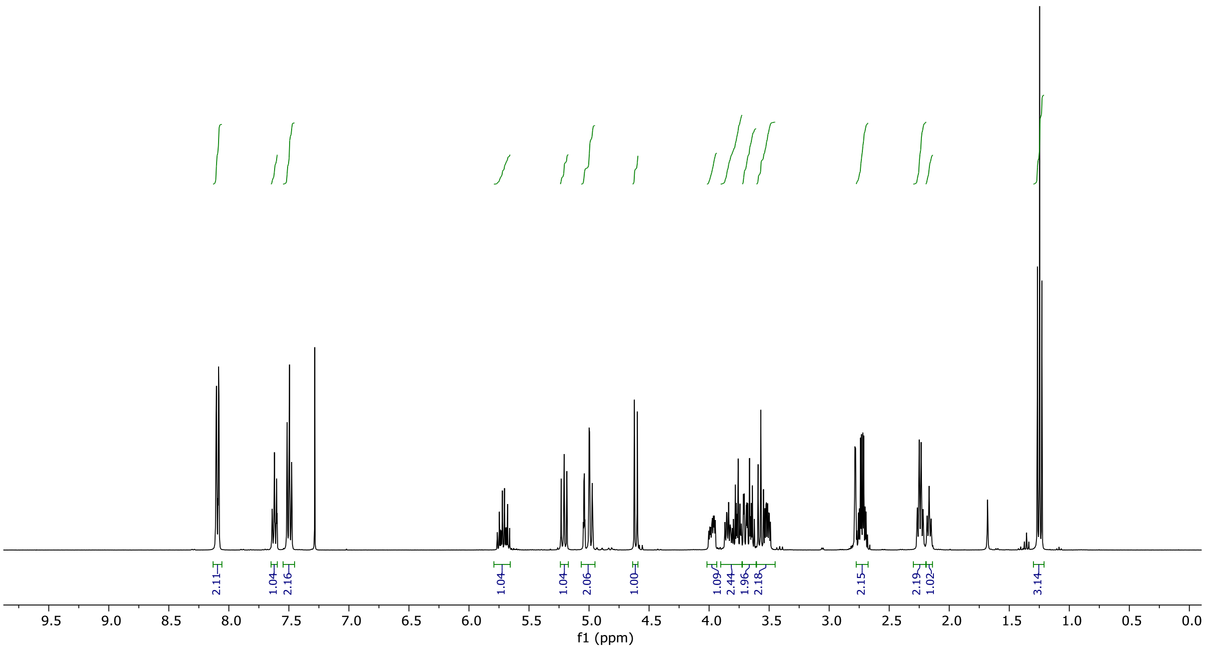

Figure S34. ^{1}H NMR (101 MHz, $CDCl_3$) spectrum of **3b-3**.

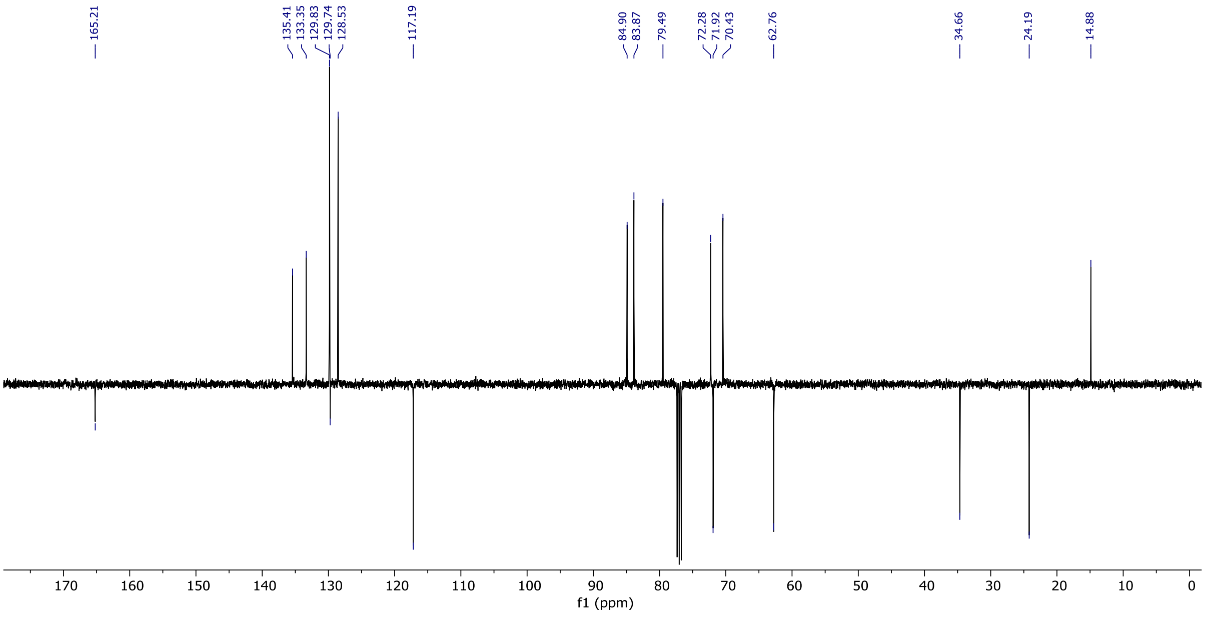

Figure S35. ^{13}C APT NMR (101 MHz, $CDCl_3$) spectrum of **3b-3**.

*Ethyl 2-O-benzoyl-3-O-(pent-4-en-1-yl)-1-thio-β-D-glucopyranoside (**3c-3**)*

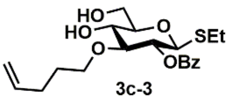

3c-3

3c-2 (1.05 g, 2.17 mmol) and TsOH (56 mg, 0.33 mmol) are allowed to react in MeOH (20 mL) according to the general procedure detailed above to afford **3c-3** (0.75 g, 86% yield) as a white solid. Rf (DCM/ MeOH 20:1) = 0.65. ^{1}H NMR (400 MHz, $CDCl_3$), δ = 8.12 – 8.08 (m, 2H), 7.67 – 7.59 (m, 1H), 7.49 (t, J = 7.8 Hz, 2H), 5.66

(ddt, J = 16.9, 10.2, 6.7 Hz, 1H), 5.21 (dd, J = 10.0, 9.0 Hz, 1H), 4.93 – 4.80 (m, 2H), 4.61 (d, J = 10.0 Hz, 1H), 3.98 (ddd, J = 11.9, 6.3, 3.5 Hz, 1H), 3.85 (ddd, J = 12.0, 7.0, 5.1 Hz, 1H), 3.76 – 3.63 (m, 2H), 3.59 – 3.46 (m, 2H), 2.73 (qd, J = 7.5, 3.0 Hz, 2H), 2.67 (d, J = 2.8 Hz, 1H), 2.16 (t, J = 6.7 Hz, 1H), 2.08 – 1.89 (m, 2H), 1.65 – 1.52 (m, 2H), 1.25 (t, J = 7.5 Hz, 3H). ^{13}C NMR (101 MHz, $CDCl_3$), δ = 165.22, 138.13, 133.33, 129.84, 129.76, 128.51, 114.77, 84.43, 83.90, 79.48, 72.36, 72.24, 70.47, 62.75, 30.12, 29.23, 24.19, 14.88.

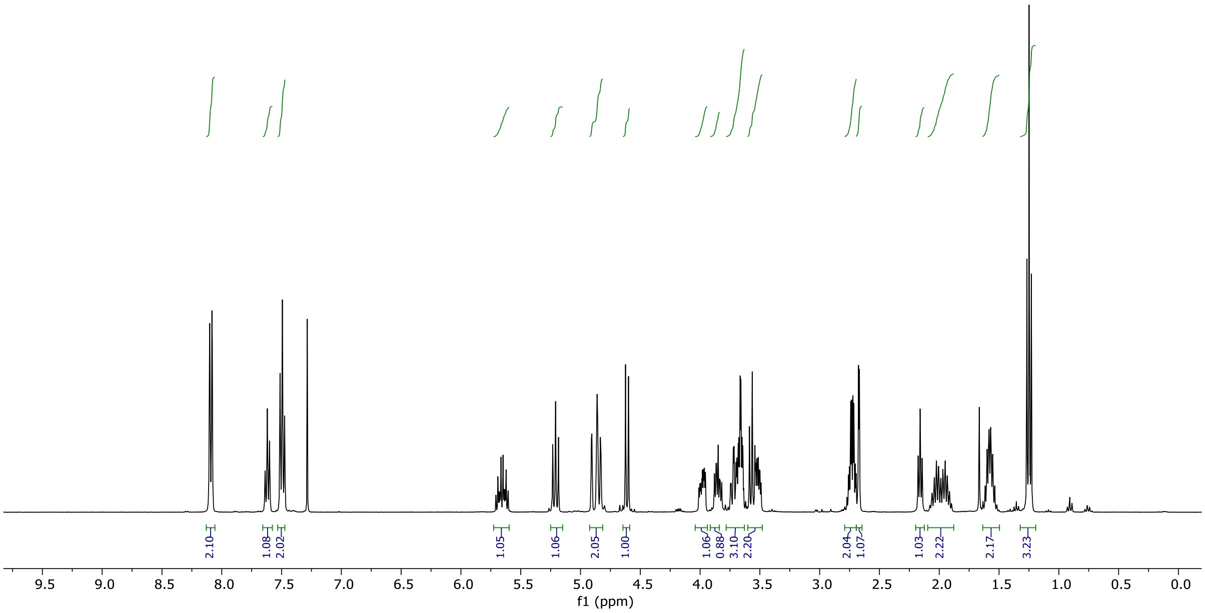

Figure S36. ^{1}H NMR (101 MHz, $CDCl_3$) spectrum of **3c-3**.

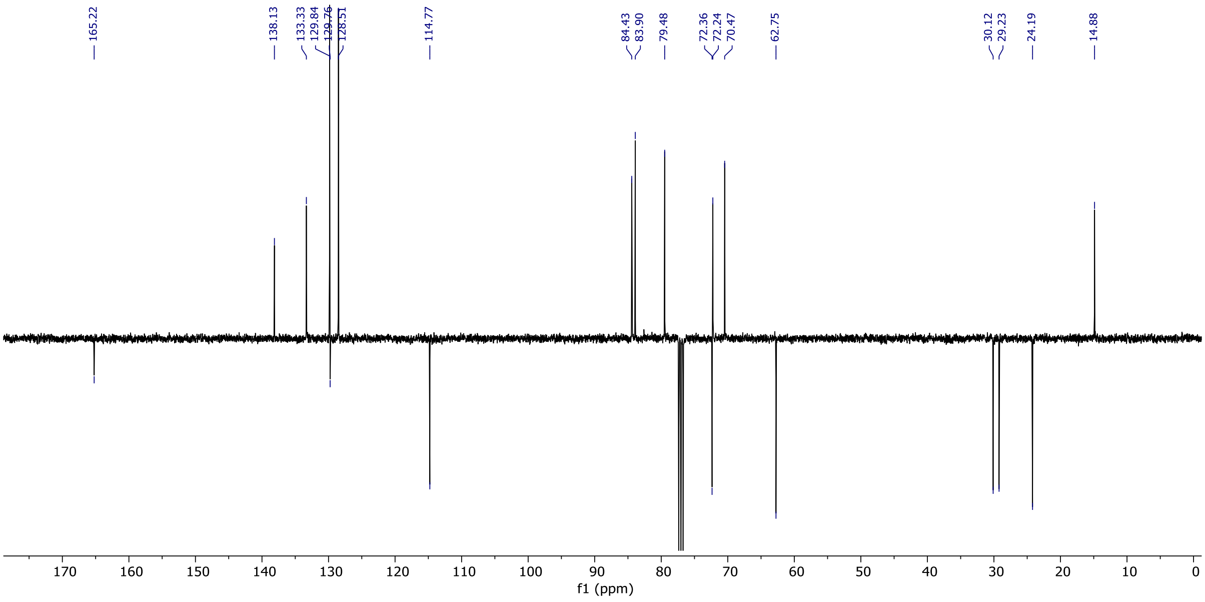

Figure S37. ^{13}C APT NMR (101 MHz, $CDCl_3$) spectrum of **3c-3**.

*Ethyl 2-O-benzoyl-3-O-(hex-5-en-1-yl)-1-thio-β-D-glucopyranoside (**3d-3**)*

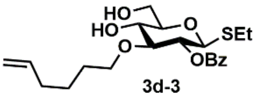

3d-3

3d-2 (2.45 g, 4.92 mmol) and TsOH (128 mg, 0.74 mmol) are allowed to react in MeOH (40 mL) according to the general procedure detailed above to afford **3d-3** (1.80 g, 89% yield) as a white solid. R*f* (DCM/ MeOH 20:1) = 0.68. ^{1}H NMR (400 MHz, $CDCl_3$), δ = 8.13 – 8.07 (m, 2H), 7.65 – 7.58 (m, 1H), 7.53 – 7.45 (m, 2H), 5.65 (ddt, *J* = 15.7, 10.7, 6.6 Hz, 1H), 5.21 (dd, *J* = 10.0, 9.1 Hz, 1H), 4.92 – 4.87 (m, 1H), 4.86 (t, *J* = 1.4 Hz, 1H), 4.61 (d, *J* = 10.0 Hz, 1H), 3.98 (ddd, *J* = 12.0, 6.2, 3.4 Hz, 1H), 3.85 (ddd, *J* = 12.1, 7.1, 5.1 Hz, 1H), 3.72 (td, *J* = 9.3, 2.9 Hz, 1H), 3.66 (td, *J* = 6.4, 3.3 Hz, 2H), 3.57 (t, *J* = 9.1 Hz, 1H), 3.51 (ddd, *J* = 9.7, 5.1, 3.4 Hz, 1H), 2.77 – 2.65 (m, 3H), 2.21 (t, *J* = 6.7 Hz, 1H), 1.95 – 1.85 (m, 2H), 1.53 – 1.42 (m, 2H), 1.32 (dd, *J* = 14.9, 7.4 Hz, 1H), 1.25 (t, *J* = 7.4 Hz, 3H). ^{13}C NMR (101 MHz, $CDCl_3$), δ = 165.21, 138.46, 133.32, 129.83, 129.76, 128.51, 114.55, 84.44, 83.88, 79.51, 72.95, 72.20, 70.49, 62.72, 33.30, 29.66, 25.17, 24.17, 14.88.

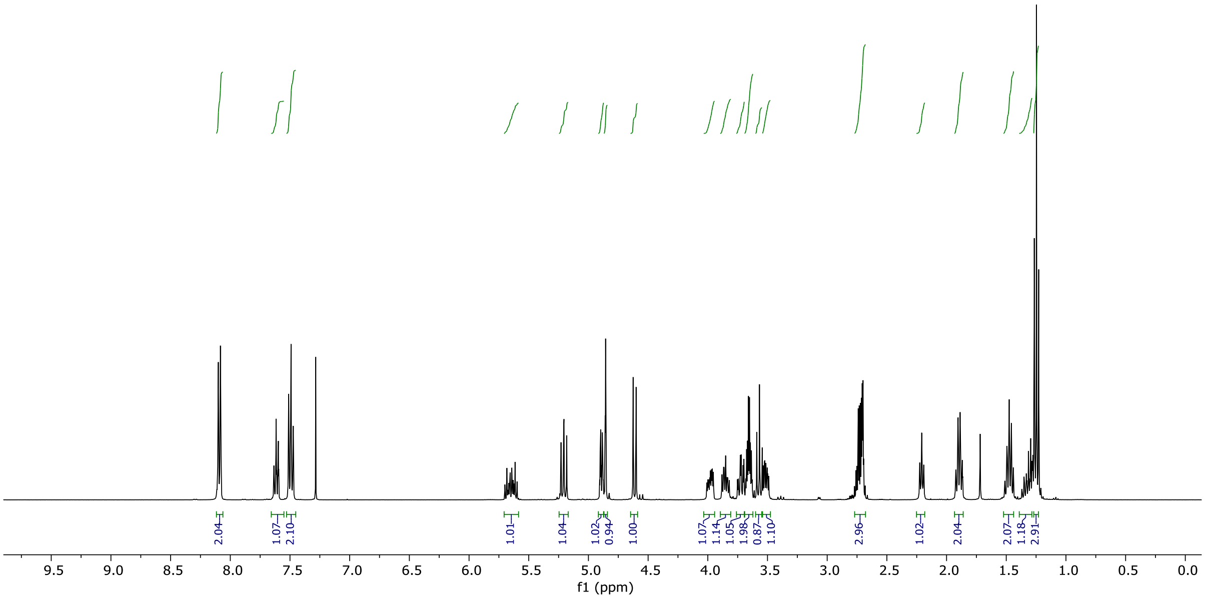

Figure S38. ^{1}H NMR (101 MHz, $CDCl_3$) spectrum of **3d-3**.

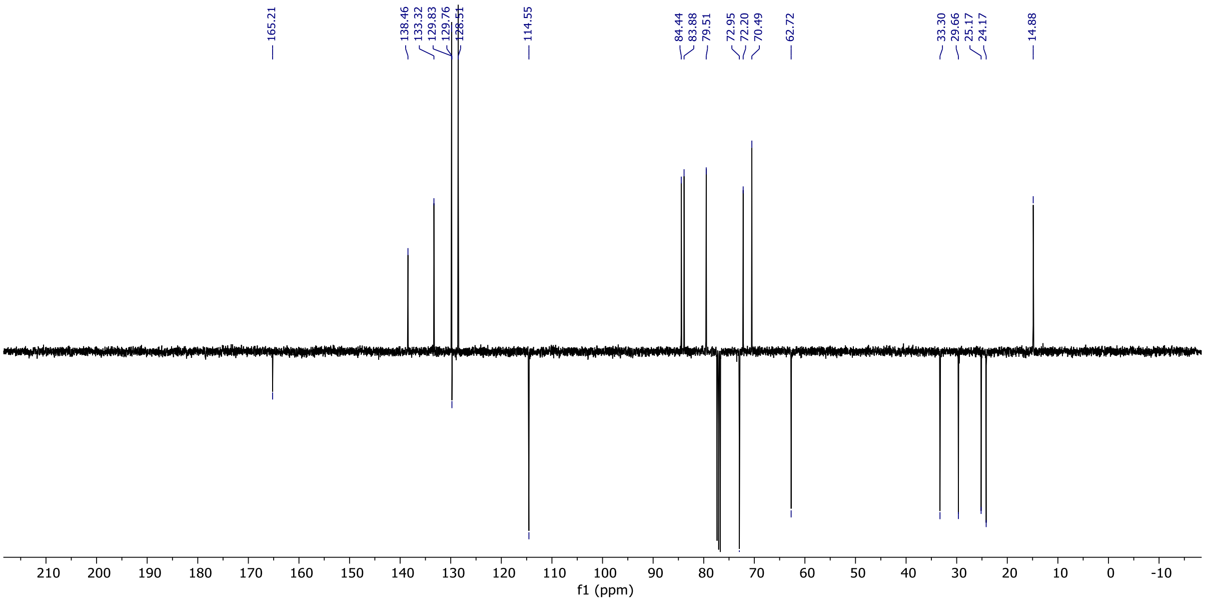

Figure S39. ^{13}C APT NMR (101 MHz, $CDCl_3$) spectrum of **3d-3**.

*Ethyl 3-O-alkenyl-2-O-benzoyl-4-O-benzyl-1-thio-β-D-glucopyranoside (**3-5**)*

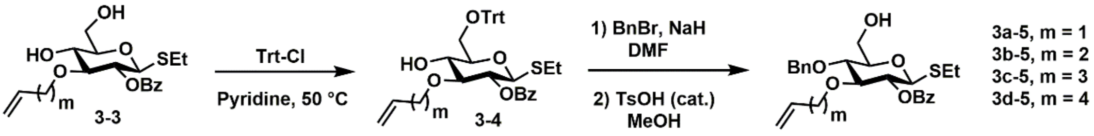

General procedure:[5] **3-3** (3.0 mmol) is dissolved with Trt-Cl (1.67 g, 6.0 mmol) in pyridine (40 mL), and the reaction mixture is stirred at 50°C overnight. The reaction is quenched with MeOH (5 mL) and the volatiles are evaporated at reduced pressure. The crude is dissolved in ethyl acetate (50 mL) and washed with a 10% soln. of $NaHCO_3$ (2×20 mL) and brine (2×20 mL). The organic layer is dried over Na_2SO_4, evaporated at reduced pressure, and placed under a high vacuum overnight. Without further purification, the tritylated product is then dissolved together with benzyl bromide (0.71 mL, 6 mmol) and a catalytic amount of TBAI (110 mg, 0.3 mmol) in DMF (20 mL). The reaction mixture is placed in an ice bath, and at 0°C, NaH (60% in mineral oil, 0.24 g, 6.0 mmol) is added portion-wise. After completion, indicated by TLC analysis (from 6 to 12 h), the reaction is quenched with a 10% soln. of citric acid (10 mL) and diluted with ethyl acetate (50 mL). The organic layer was washed with water (2×20 mL), brine (2×20 mL), and dried over Na_2SO_4. After removing the solvent, the crude is suspended in MeOH/DCM 3:1 (40 mL), and in the presence of TsOH (130 mg, 0.75 mmol) the reaction mixture is subjected to an ultrasonic bath for 30 min. The clear solution is quenched with Et_3N (1 mL) and the volatiles are evaporated under reduced pressure. The crude product is purified by column chromatography (hex/EA 4:1) to afford **3-5** (52-69% yield) as a white solid.

*Ethyl 3-O-allyl-2-O-benzoyl-4-O-benzyl-1-thio-β-D-glucopyranoside (**3a-5**)*

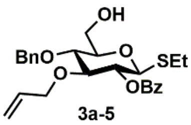

3a-5

According the procedure detailed above, **3a-3** (2.25 g, 6.11 mmol) in dissolved in pyridine (60 mL) and tritylated with Trt-Cl (3.4 g, 12.2 mmol). Without further purification, the product is allowed to react with BnBr (1.45 mL, 12.2 mmol), TBAI (224 mg, 0.61 mmol) and NaH (60% in mineral oil, 0.49 g, 12.2 mmol) in DMF (30 mL) as described. Trityl group is removed with TsOH (264 mg, 1.53 mmol) in MeOH/DCM 3:1 (60 mL), and the final product is purified by column chromatography (hex/EA 4:1) to afford **3a-5** (1.93 g, 69% yield) as a white solid. R*f* (hex/EA 3:1) = 0.45. ^{1}H NMR (400 MHz, $CDCl_3$) δ = 8.15 – 8.07 (m, 2H), 7.68 – 7.58 (m, 1H), 7.54 – 7.47 (m, 2H), 7.42 – 7.32 (m, 5H), 5.77 (ddt, *J* = 17.2, 10.3, 5.8 Hz, 1H), 5.24 (dd, *J* = 10.1, 9.0 Hz, 1H), 5.14 (dd, *J* = 17.2, 1.6 Hz, 1H), 5.09 – 5.01 (m, 1H), 4.91 (d, *J* = 10.9 Hz, 1H), 4.70 (d, *J* = 11.0 Hz, 1H), 4.59 (d, *J* = 10.1 Hz, 1H), 4.27 (ddt, *J* = 12.3, 5.7, 1.3 Hz, 1H), 4.17 (ddt, *J* = 12.4, 6.0, 1.4 Hz, 1H), 3.93 (ddd, *J* = 12.0, 6.2, 2.7 Hz, 1H), 3.81 – 3.66 (m, 3H), 3.48 (ddd, *J* = 9.6, 4.8, 2.7 Hz, 1H), 2.73 (qd, *J* = 7.5, 2.4 Hz, 2H), 1.97 (dd, *J* = 7.6, 6.2 Hz, 1H), 1.25 (t, *J* = 7.4 Hz, 3H). ^{13}C NMR (101 MHz, $CDCl_3$), δ = 165.22, 137.80, 134.54, 133.26, 129.85, 128.57, 128.48, 128.24, 128.08, 117.36, 83.88, 83.72, 79.67, 75.22, 74.15, 72.43, 62.10, 24.17, 14.91.

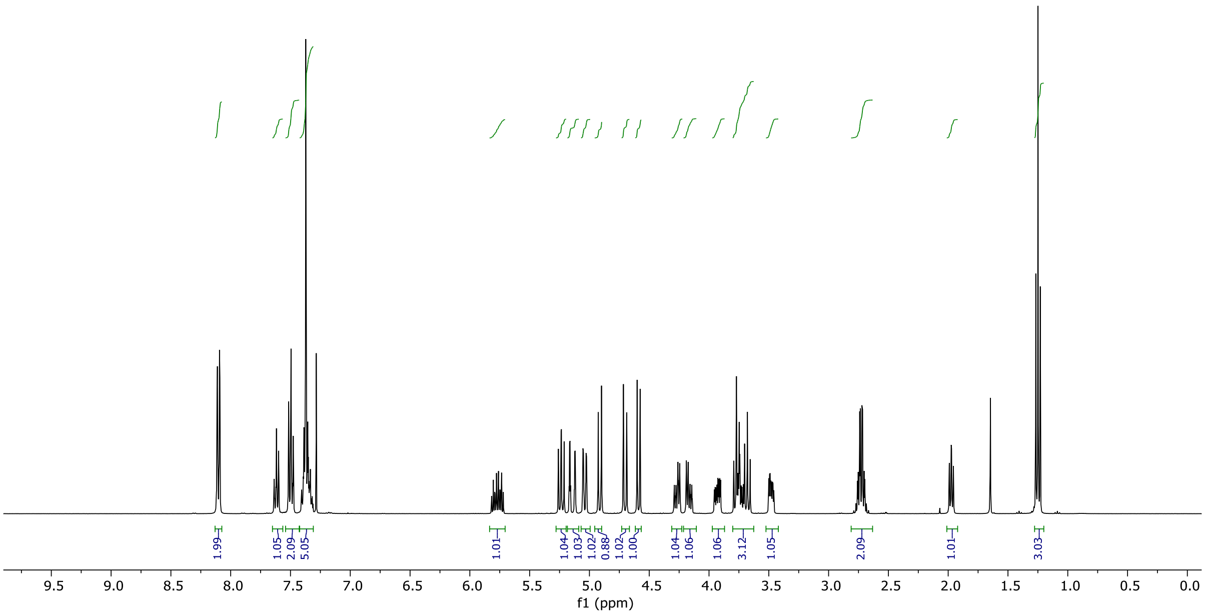

Figure 40 ^{1}H NMR (101 MHz, $CDCl_3$) spectrum of **3a-5**.

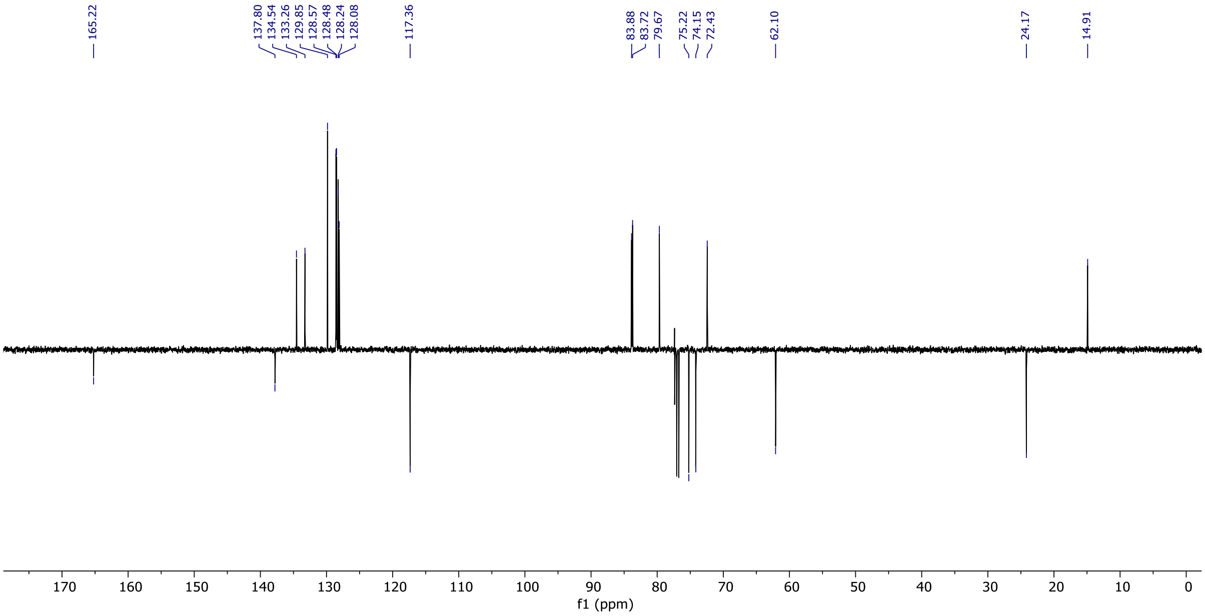

Figure S41. ^{13}C APT NMR (101 MHz, $CDCl_3$) spectrum of **3a-5**.

*Ethyl 2-O-benzoyl-4-O-benzyl-3-O-(but-3-en-1-yl)-1-thio-β-D-glucopyranoside (**3b-5**)*

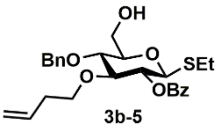

3b-5

3b-3 (1.53 g, 4.0 mmol) in dissolved in pyridine (40 mL) and tritylated with Trt-Cl (2.23 g, 8.0 mmol). Without further purification, the product is allowed to react with BnBr (0.95 mL, 8.0 mmol), TBAI (145 mg, 0.4 mmol) and NaH (60% in mineral oil, 0.32 g, 8.0 mmol) in DMF (20 mL) as described. Trityl group is removed with TsOH (173 mg, 1.0 mmol) in MeOH/DCM 3:1 (40 mL), and the final product is purified by column chromatography (hex/EA 4:1) to afford **3b-5** (1.15 g, 61% yield) as a white solid. R*f* (hex/EA 3:1) = 0.45. ^{1}H NMR (400 MHz, $CDCl_3$), δ = 8.14 – 8.07 (m, 2H), 7.65 – 7.58 (m, 1H), 7.54 – 7.47 (m, 2H), 7.41 – 7.30 (m, 5H), 5.63 (ddt, *J* = 17.0, 10.2, 6.8 Hz, 1H), 5.23 (dd, *J* = 10.0, 8.7 Hz, 1H), 4.94 – 4.88 (m, 2H), 4.86 – 4.81 (m, 1H), 4.70 (d, *J* = 11.0 Hz, 1H), 4.58 (d, *J* = 10.0 Hz, 1H), 3.96 – 3.90 (m, 1H), 3.82 (dt, *J* = 9.0, 6.6 Hz, 1H), 3.77 – 3.61 (m, 4H), 3.47 (ddd, *J* = 9.1, 4.8, 2.6 Hz, 1H), 2.73 (qd, *J* = 7.5, 2.3 Hz, 2H), 2.21 (qq, *J* = 6.7, 1.4 Hz, 2H), 1.97 (tt, *J* = 6.6, 2.2 Hz, 1H), 1.25 (t, *J* = 7.5 Hz, 3H). ^{13}C NMR (101 MHz, $CDCl_3$), δ = 165.26, 137.86, 134.82, 133.24, 129.89, 129.82, 128.57, 128.48, 128.15, 128.05, 116.50, 84.92, 83.70, 79.70, 77.29, 76.73, 75.14, 72.94, 72.43, 62.12, 34.75, 24.12, 14.90.

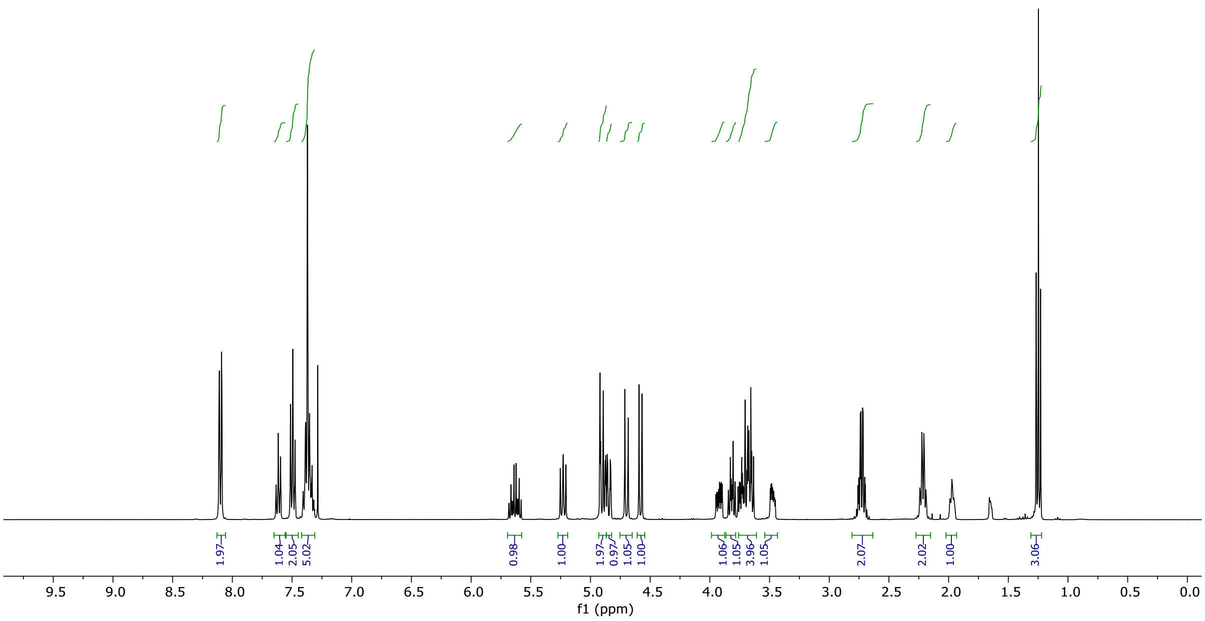

Figure S42. ^{1}H NMR (101 MHz, $CDCl_3$) spectrum of **3b-5**.

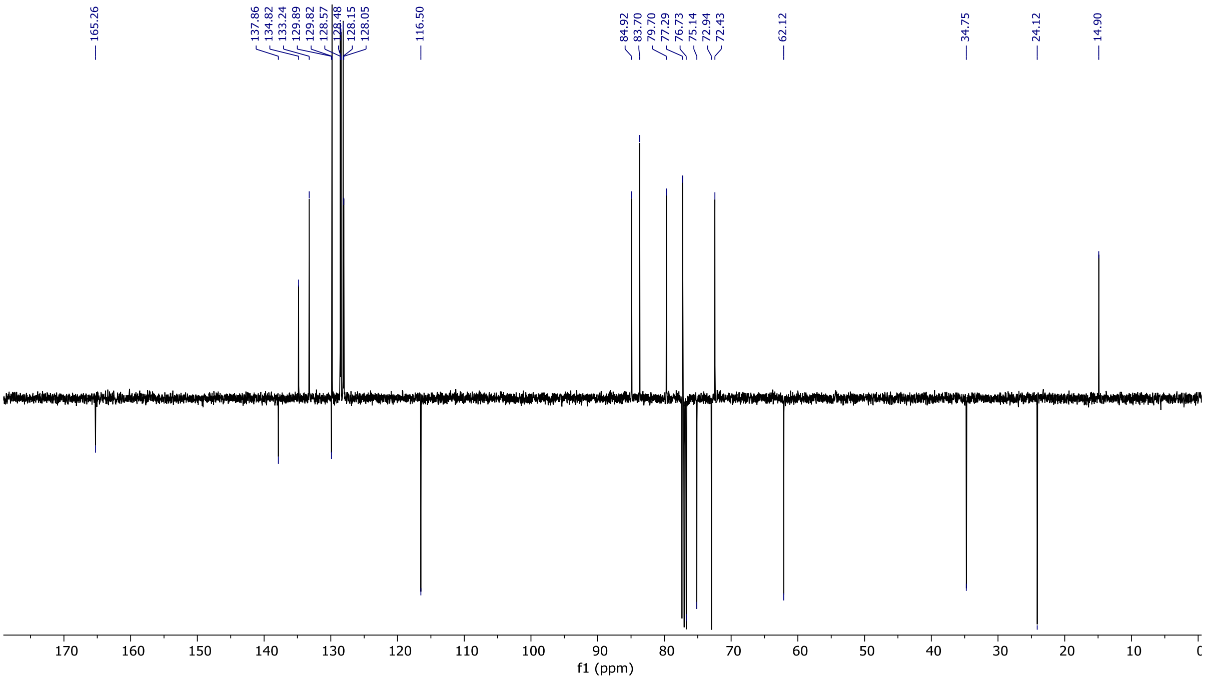

Figure S43. ^{13}C APT NMR (101 MHz, $CDCl_3$) spectrum of **3b-5**.

*Ethyl 2-O-benzoyl-4-O-benzyl-3-O-(pent-4-en-1-yl)-1-thio-β-D-glucopyranoside (**3c-5**)*

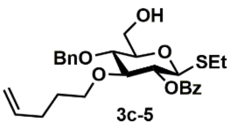

3c-5

3c-3 (1.42 g, 3.6 mmol) in dissolved in pyridine (40 mL) and tritylated with Trt-Cl (2.0 g, 7.2 mmol). Without further purification, the product is allowed to react with BnBr (0.85 mL, 7.2 mmol), TBAI (132 mg, 0.36 mmol) and NaH (60% in mineral oil, 0.29 g, 7.2 mmol) in DMF (20 mL) as described. Trityl

group is removed with TsOH (156 mg, 0.9 mmol) in MeOH/DCM 3:1 (40 mL), and the final product is purified by column chromatography (hex/EA 4:1) to afford **3c-5** (1.18 g, 68% yield) as a white solid. R*f* (hex/EA 3:1) = 0.47. ^{1}H NMR (400 MHz, $CDCl_3$), δ = 8.13 – 8.06 (m, 2H), 7.65 – 7.58 (m, 1H), 7.54 – 7.47 (m, 2H), 7.41 – 7.33 (m, 5H), 5.59 (ddt, *J* = 16.9, 10.2, 6.6 Hz, 1H), 5.26 – 5.18 (m, 1H), 4.90 (d, *J* = 10.9 Hz, 1H), 4.85 – 4.76 (m, 2H), 4.70 (d, *J* = 11.0 Hz, 1H), 4.58 (d, *J* = 10.1 Hz, 1H), 3.92 (ddd, *J* = 12.1, 6.2, 2.6 Hz, 1H), 3.82 – 3.58 (m, 5H), 3.47 (ddd, *J* = 9.1, 4.7, 2.6 Hz, 1H), 2.73 (qd, *J* = 7.4, 2.4 Hz, 2H), 2.03 – 1.90 (m, 2H), 1.92 – 1.83 (m, 1H), 1.62 – 1.50 (m, 2H), 1.25 (t, *J* = 7.5 Hz, 3H). ^{13}C NMR (101 MHz, $CDCl_3$), δ = 165.27, 137.93, 137.84, 133.24, 129.84, 128.57, 128.48, 128.15, 128.06, 114.59, 84.78, 83.70, 79.69, 77.39, 75.14, 72.95, 72.46, 62.12, 30.03, 29.45, 24.13, 14.91.

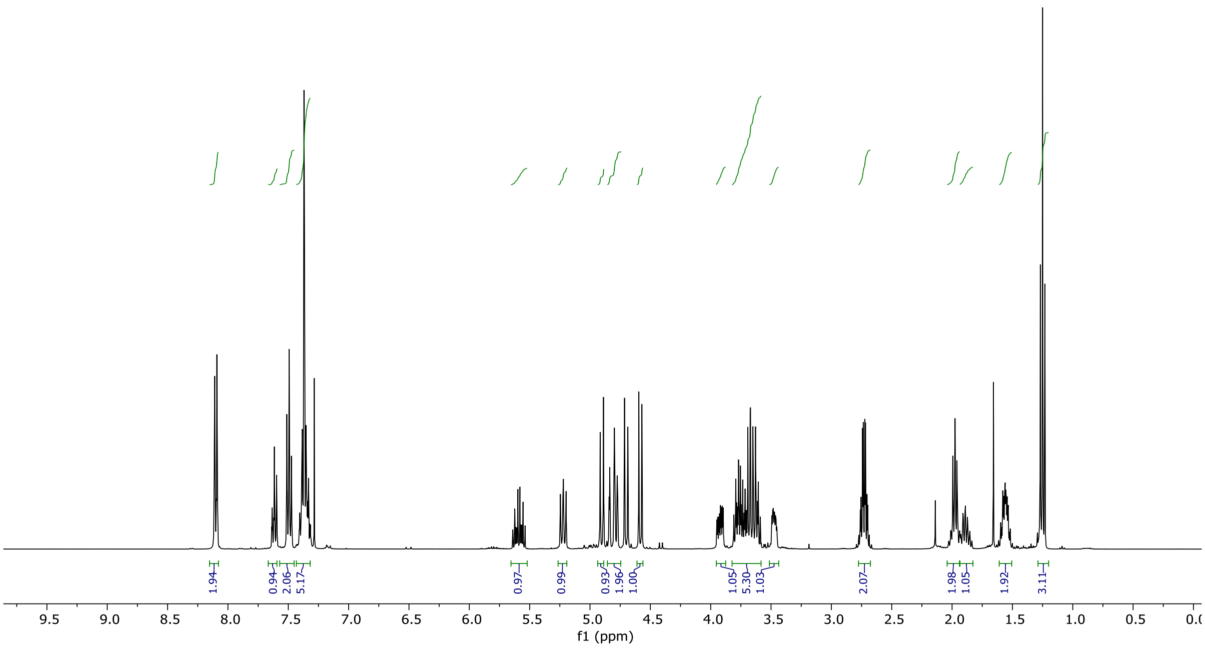

Figure S44. ^{1}H NMR (101 MHz, $CDCl_3$) spectrum of **3c-5**.

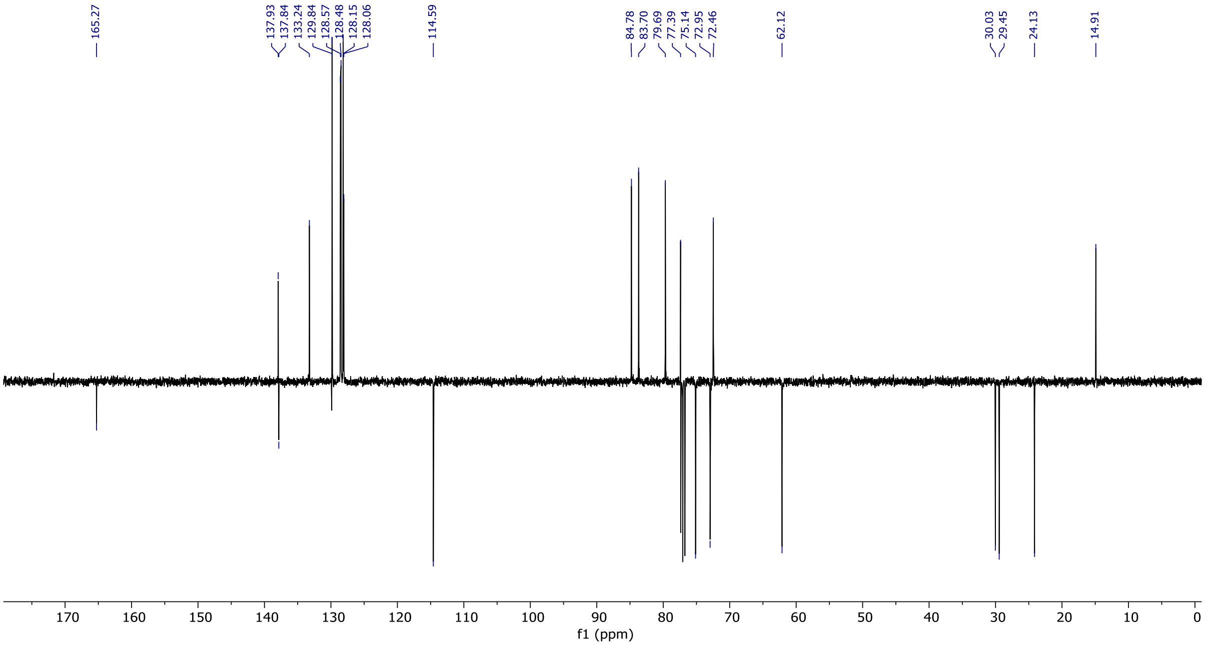

Figure S45. ^{13}C APT NMR (101 MHz, $CDCl_3$) spectrum of **3c-5**.

Ethyl 2-O-benzoyl-4-O-benzyl-3-O-(hex-5-en-1-yl)-1-thio-β-D-glucopyranoside (**3d-5**)

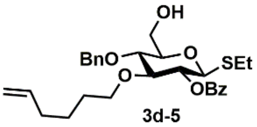

3d-5

3d-3 (1.8 g, 4.39 mmol) in dissolved in pyridine (50 mL) and tritylated with Trt-Cl (2.44 g, 8.8 mmol). Without further purification, the product is allowed to react with BnBr (1.04 mL, 8.8 mmol), TBAI (161 mg, 0.44 mmol) and NaH (60% in mineral oil, 0.35 g, 8.8 mmol) in DMF (30 mL) as described. Trityl group is removed with TsOH (190 mg, 1.1 mmol) in MeOH/DCM 3:1 (50 mL), and the final product is purified by column chromatography (hex/EA 4:1) to afford **3d-5** (1.14 g, 52% yield) as a white solid. R*f* (hex/EA 2:1) = 0.49. ^{1}H NMR (400 MHz, $CDCl_3$), δ = 8.13 – 8.08 (m, 2H), 7.64 – 7.59 (m, 1H), 7.54 – 7.45 (m, 2H), 7.43 – 7.32 (m, 5H), 5.68 – 5.54 (m, 1H), 5.25 – 5.18 (m, 1H), 4.90 (d, *J* = 11.0 Hz, 1H), 4.84 (dq, *J* = 14.3, 1.7 Hz, 2H), 4.70 (d, *J* = 10.9 Hz, 1H), 4.58 (d, *J* = 10.0 Hz, 1H), 3.92 (ddd, *J* = 12.1, 6.2, 2.7 Hz, 1H), 3.82 – 3.57 (m, 5H), 3.47 (ddd, *J* = 9.1, 4.7, 2.6 Hz, 1H), 2.73 (qd, *J* = 7.5, 2.6 Hz, 2H), 1.95 (dd, *J* = 7.6, 6.2 Hz, 1H), 1.85 (tdt, *J* = 8.0, 6.7, 1.4 Hz, 2H), 1.46 (dp, *J* = 8.6, 6.4 Hz, 2H), 1.36 – 1.29 (m, 2H), 1.26 (d, *J* = 7.4 Hz, 3H). ^{13}C NMR (101 MHz, $CDCl_3$), δ = 165.25, 138.50, 137.85, 133.25, 129.84, 128.57, 128.48, 128.14, 128.05, 114.41, 84.76, 83.70, 79.68, 77.41, 75.12, 73.47, 72.43, 62.14, 33.33, 29.78, 25.25, 24.11, 14.91.

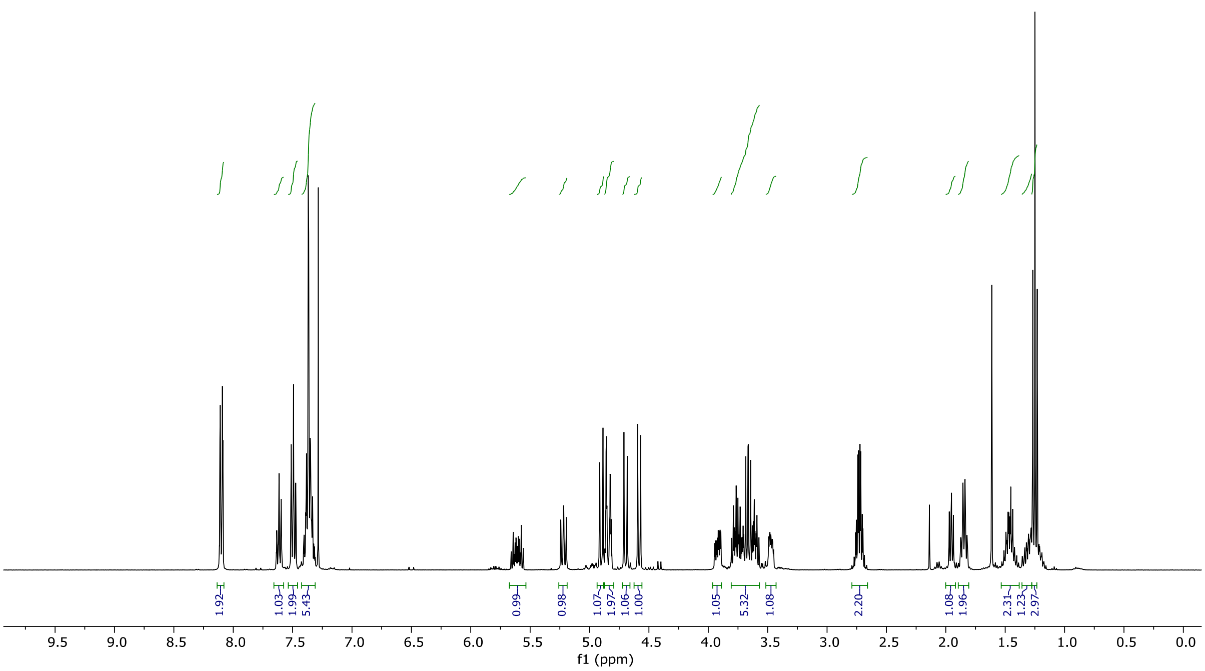

Figure S46. ^{1}H NMR (101 MHz, $CDCl_3$) spectrum of **3d-5**.

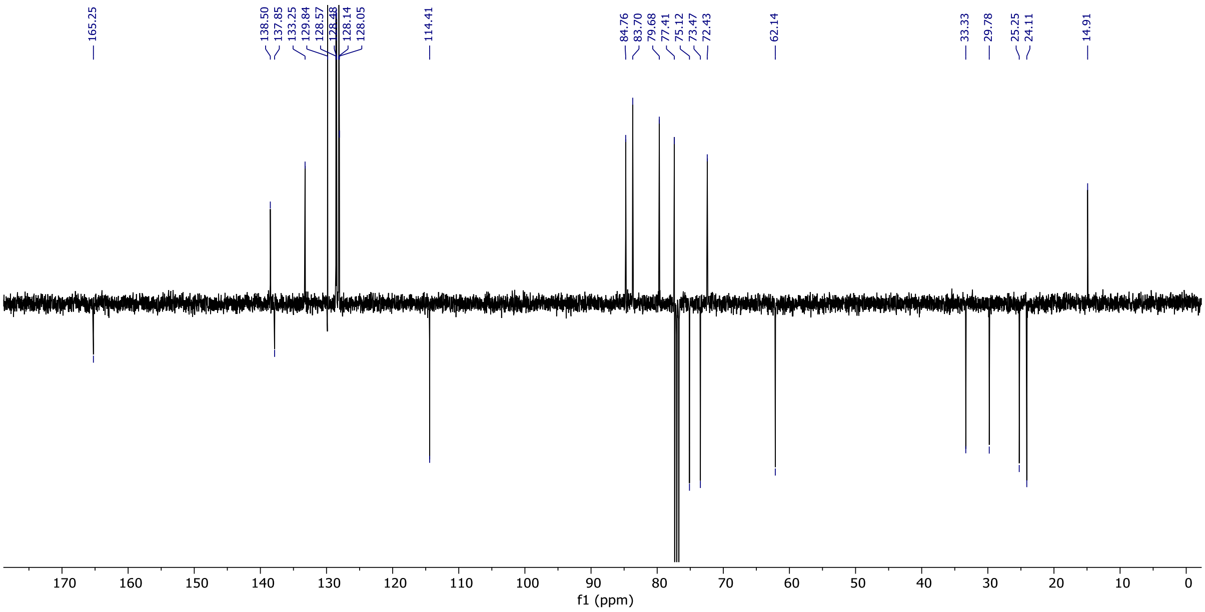

Figure S47. ^{13}C APT NMR (101 MHz, $CDCl_3$) spectrum of **3d-5**.

*Ethyl 3-O-alkenyl-2-O-benzoyl-4-O-benzyl-6-O-fluorenylmethoxycarbonyl-1-thio-β-D-glucopyranoside (**3-6**)*

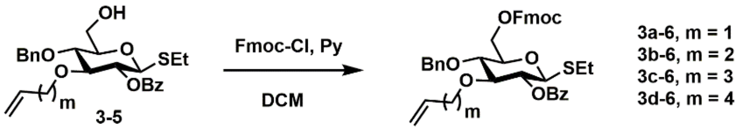

Similar to a previous report,[7] **3-5** (3.0 mmol) is dissolved in DCM (30 mL), pyridine (0.48 mL, 6.0 mmol) is added and the mixture is placed in an ice bath. At 0 °C, Fmoc-Cl (0.93 mL, 3.6

mmol) is added portion-wise and the reaction is stirred initially for 30 min, and then it is left to reach room temperature and stirred for an additional 4 h. The reaction mixture is diluted with chloroform (50 mL), transferred to a separatory funnel, and washed with a 10% soln. of citric acid (2×20 mL) and brine (2×20 mL). The organic layer is dried over Na_2SO_4 and evaporated at reduced pressure. The crude product is purified by column chromatography (hex/EA 6:1) to afford **3-6** (62-91% yield) as a white solid.

*Ethyl 3-O-allyl-2-O-benzoyl-4-O-benzyl-6-O-fluorenylmethoxycarbonyl-1-thio-β-D-glucopyranoside (**3a-6**)*

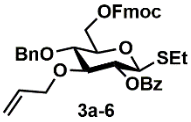

3a-6

3a-5 (1.93 g, 4.2 mmol), pyridine (0.67 mL, 8.4 mmol) and BzCl (1.3 mL, 5.0 mmol) are allowed to react in DCM (30 mL) according to the general procedure detailed above to afford **3a-6** (2.6 g, 91% yield) as a white solid. R*f* (hex/EA 4:1) = 0.50. HR-MS *m/z* = 703.2390 $[M+Na]^+$, calcd for $C_{40}H_{40}NaO_8S$: 703.2342. ^1H NMR (400 MHz, $CDCl_3$), δ = 8.16 – 8.07 (m, 2H), 7.80 (dq, *J* = 7.6, 1.0 Hz, 2H), 7.69 – 7.59 (m, 3H), 7.54 – 7.48 (m, 2H), 7.47 – 7.41 (m, 2H), 7.38 – 7.30 (m, 7H), 5.79 (ddt, *J* = 17.2, 10.3, 5.8 Hz, 1H), 5.30 (dd, *J* = 10.0, 9.1 Hz, 1H), 5.16 (dq, *J* = 17.2, 1.6 Hz, 1H), 5.06 (dq, *J* = 10.4, 1.3 Hz, 1H), 4.94 (d, *J* = 10.9 Hz, 1H), 4.67 (d, *J* = 10.9 Hz, 1H), 4.58 (d, *J* = 10.0 Hz, 1H), 4.50 (dd, *J* = 11.6, 1.8 Hz, 1H), 4.42 (dd, *J* = 7.5, 2.8 Hz, 2H), 4.36 – 4.25 (m, 3H), 4.23 – 4.15 (m, 1H), 3.79 (ddd, *J* = 8.9, 7.0, 1.5 Hz, 1H), 3.70 – 3.65 (m, 2H), 2.78 – 2.65 (m, 2H), 1.24 (t, *J* = 7.4 Hz, 3H). ^{13}C NMR (101 MHz, $CDCl_3$), δ = 165.21, 154.98, 143.39, 143.31, 141.30, 137.54, 134.45, 133.28, 129.87, 129.83, 128.60, 128.49, 128.27, 128.13, 127.93, 127.22, 125.25, 125.21, 120.08, 117.48, 84.04, 83.62, 77.22, 77.15, 75.24, 74.21, 72.30, 70.03, 66.63, 46.71, 24.11, 14.94.

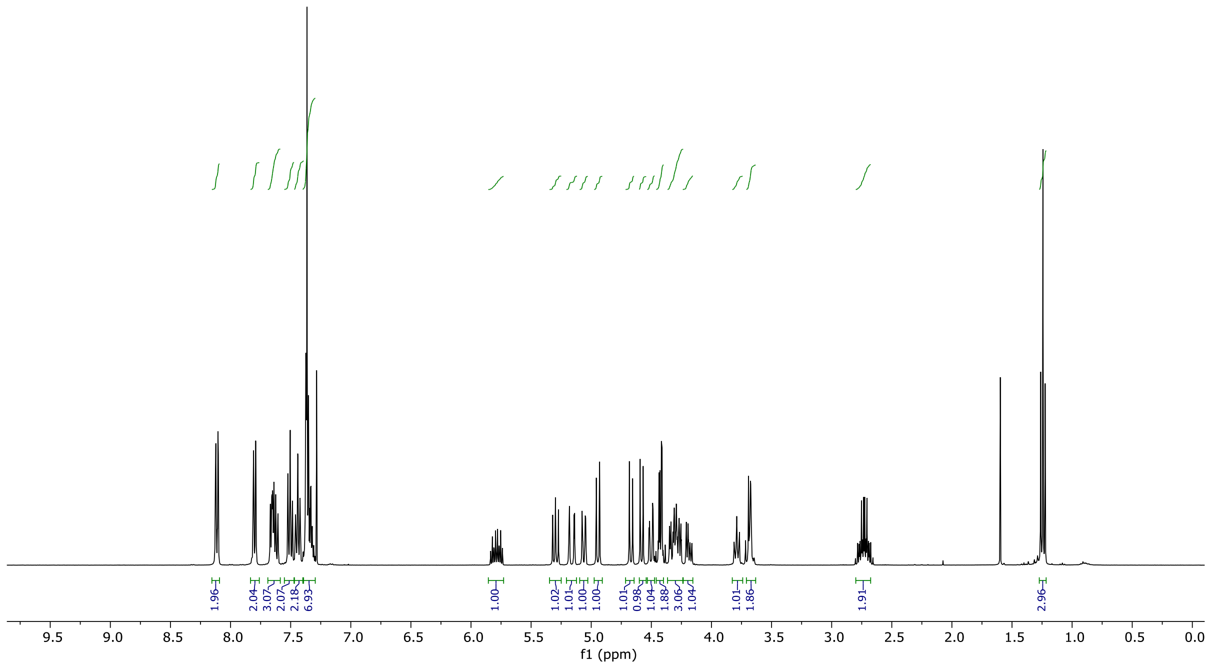

Figure S48. ^{1}H NMR (101 MHz, $CDCl_3$) spectrum of **3a-6**.

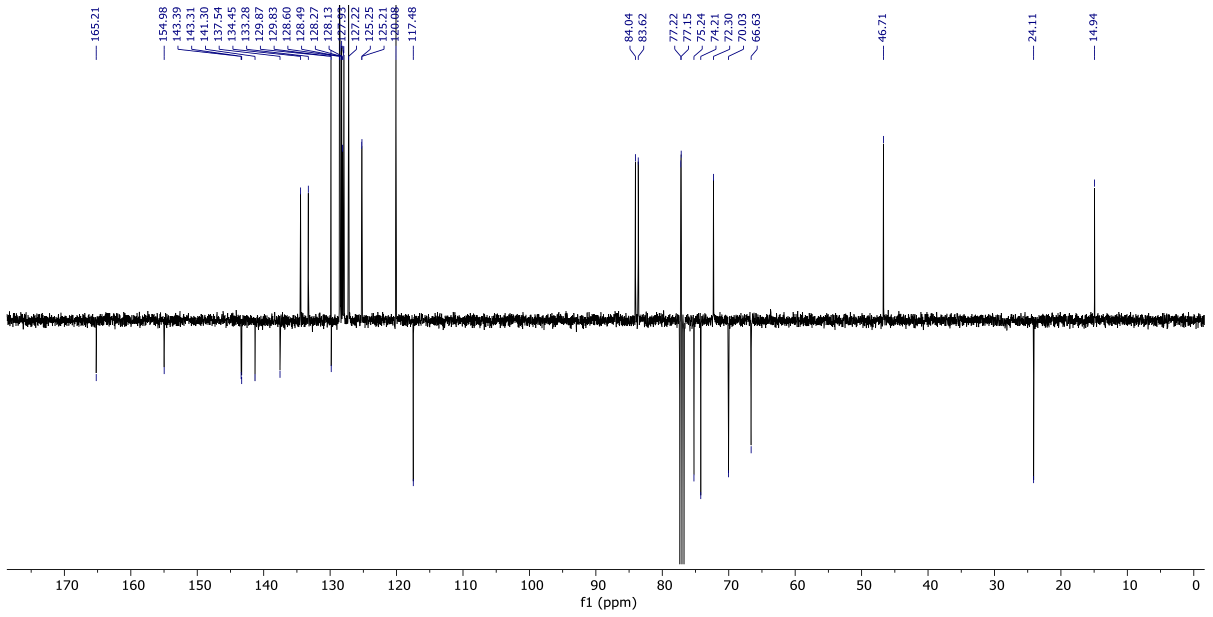

Figure S49. ^{13}C APT NMR (101 MHz, $CDCl_3$) spectrum of **3a-6**.

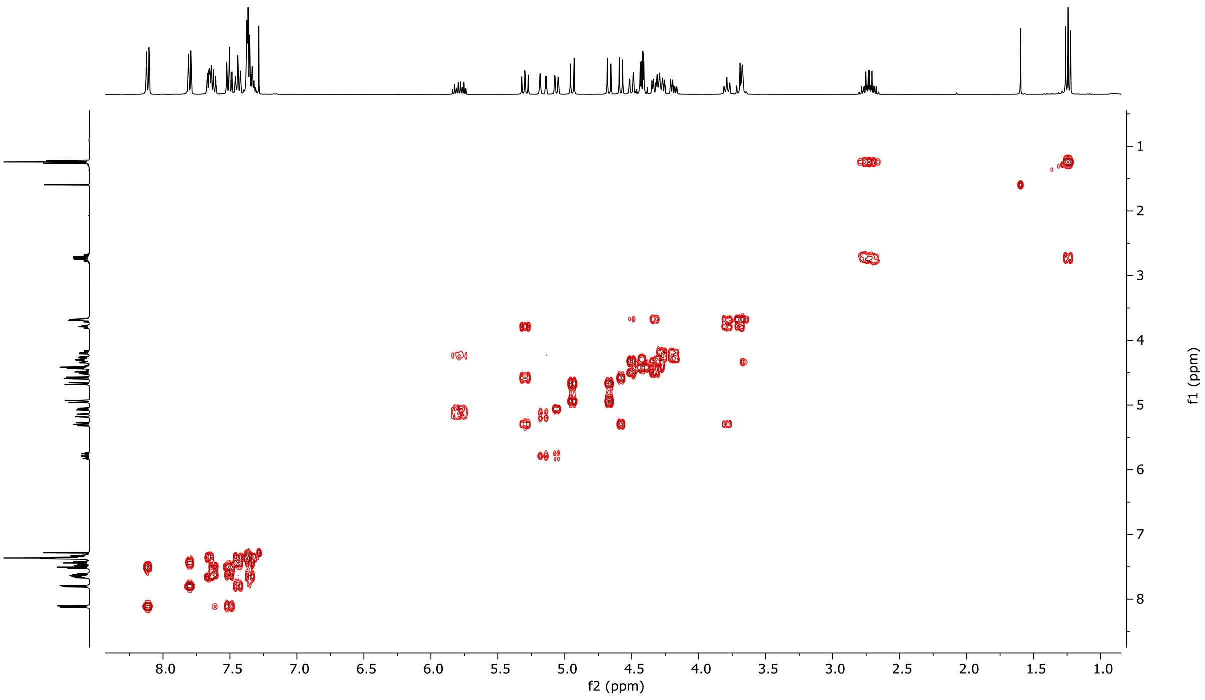

Figure S50. COSY NMR (400 MHz, $CDCl_3$) spectrum of **3a-6**.

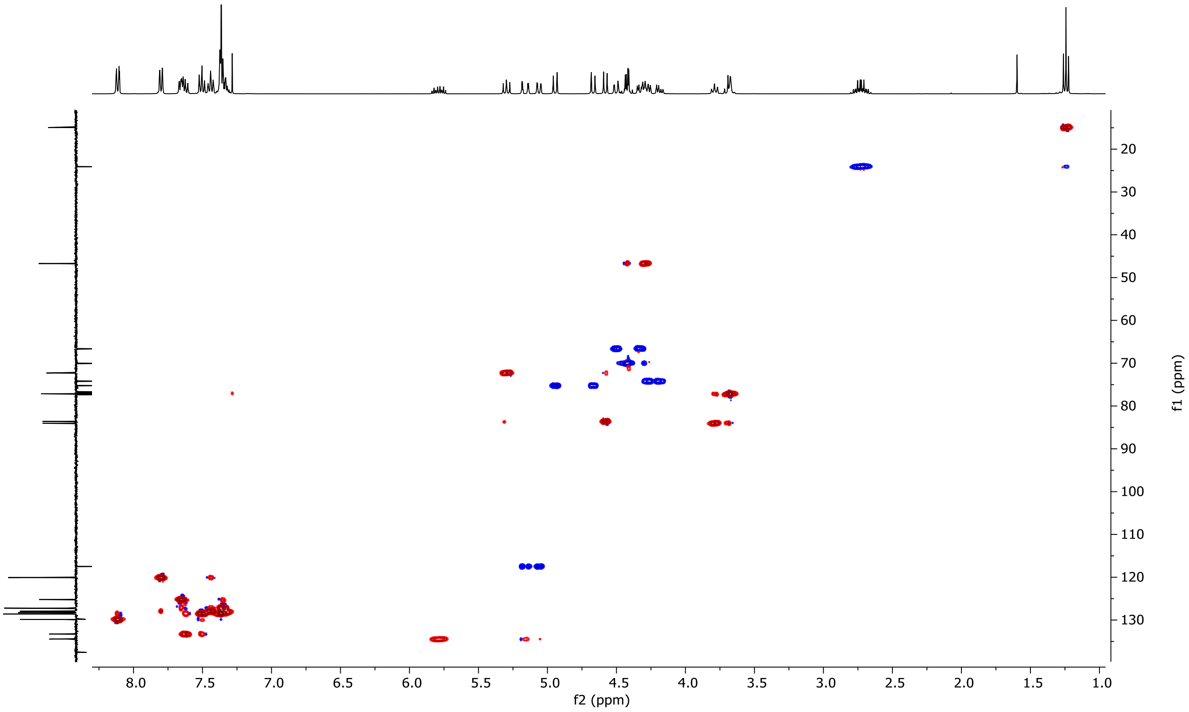

Figure S51. HSQC NMR (400 MHz, $CDCl_3$) spectrum of **3a-6**.

*Ethyl 2-O-benzoyl-4-O-benzyl-3-O-(but-3-en-yl)-6-O-fluorenylmethoxycarbonyl-1-thio-β-D-glucopyranoside (**3b-6**)*

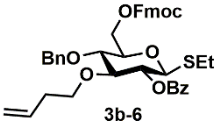

3b-6

3b-5 (1.15 g, 2.43 mmol), pyridine (0.39 mL, 4.9 mmol) and BzCl (0.75 mL, 2.9 mmol) are allowed to react in DCM (30 mL) according to the general procedure detailed above to afford **3b-6** (1.45 g, 86% yield) as a white solid. R*f* (hex/EA 4:1) = 0.50. HR-MS *m/z* = 717.2543 $[M+Na]^+$, calcd for $C_{41}H_{42}NaO_8S$: 717.2498. ^1H NMR (400 MHz, $CDCl_3$), δ = 8.15 – 8.09 (m, 2H), 7.80 (dq, *J* = 7.5, 0.9 Hz, 2H), 7.69 – 7.59 (m, 3H), 7.54 – 7.49 (m, 2H), 7.46 – 7.41 (m, 2H), 5.65 (ddt, *J* = 17.0, 10.2, 6.8 Hz, 1H), 5.29 (dd, *J* = 10.0, 8.7 Hz, 1H), 4.98 – 4.89 (m, 2H), 4.87 (ddt, *J* = 10.2, 2.1, 1.1 Hz, 1H), 4.67 (d, *J* = 11.0 Hz, 1H), 4.57 (d, *J* = 10.1 Hz, 1H), 4.49 (dd, *J* = 11.7, 1.5 Hz, 1H), 4.42 (dd, *J* = 7.5, 2.9 Hz, 2H), 4.31 (ddd, *J* = 14.8, 8.3, 4.9 Hz, 2H), 3.82 (dt, *J* = 9.0, 6.5 Hz, 1H), 3.74 – 3.64 (m, 3H), 2.74 (dtt, *J* = 14.9, 7.4, 3.7 Hz, 2H), 2.23 (dddd, *J* = 8.1, 6.7, 5.3, 1.4 Hz, 2H), 1.24 (t, *J* = 7.4 Hz, 3H). ^{13}C NMR (101 MHz, $CDCl_3$), δ = 165.24, 154.98, 143.40, 143.32, 141.30, 137.61, 134.77, 133.26, 129.88, 129.84, 128.60, 128.50, 128.19, 128.10, 127.93, 127.22, 125.26, 125.22, 120.08, 116.59, 85.10, 83.59, 77.17, 77.11, 75.17, 73.00, 72.29, 70.03, 66.63, 46.71, 34.77, 24.06, 14.93.

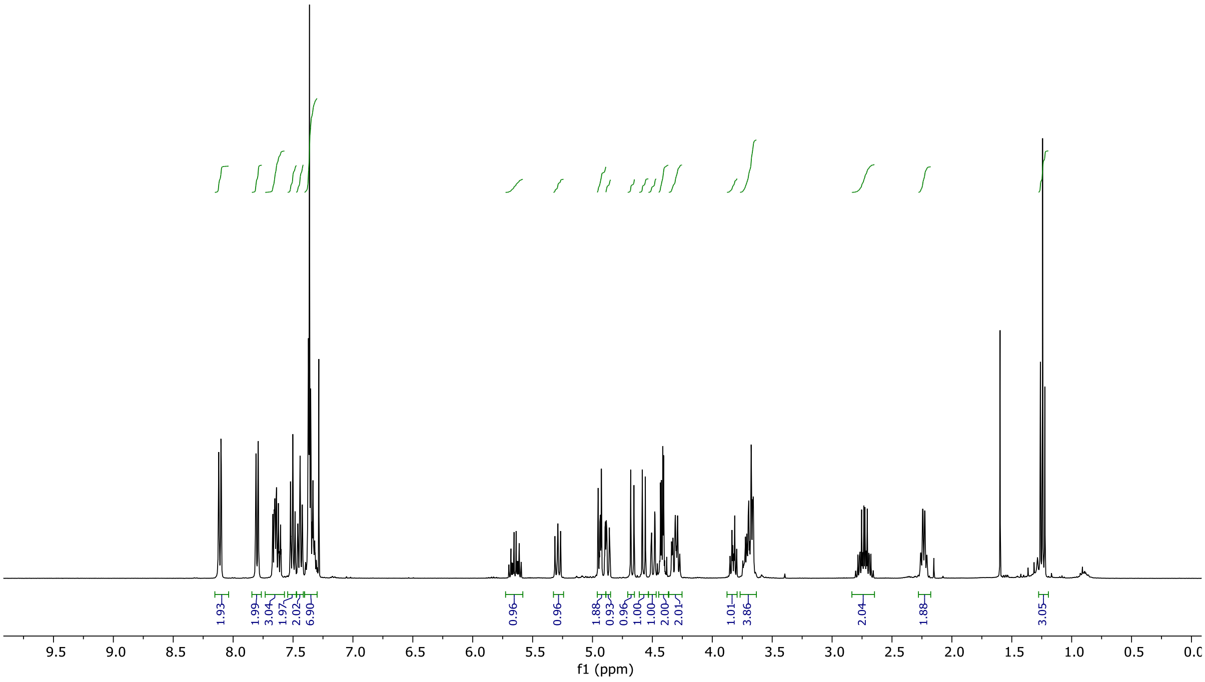

Figure S52. ^1H NMR (101 MHz, $CDCl_3$) spectrum of **3b-6**.

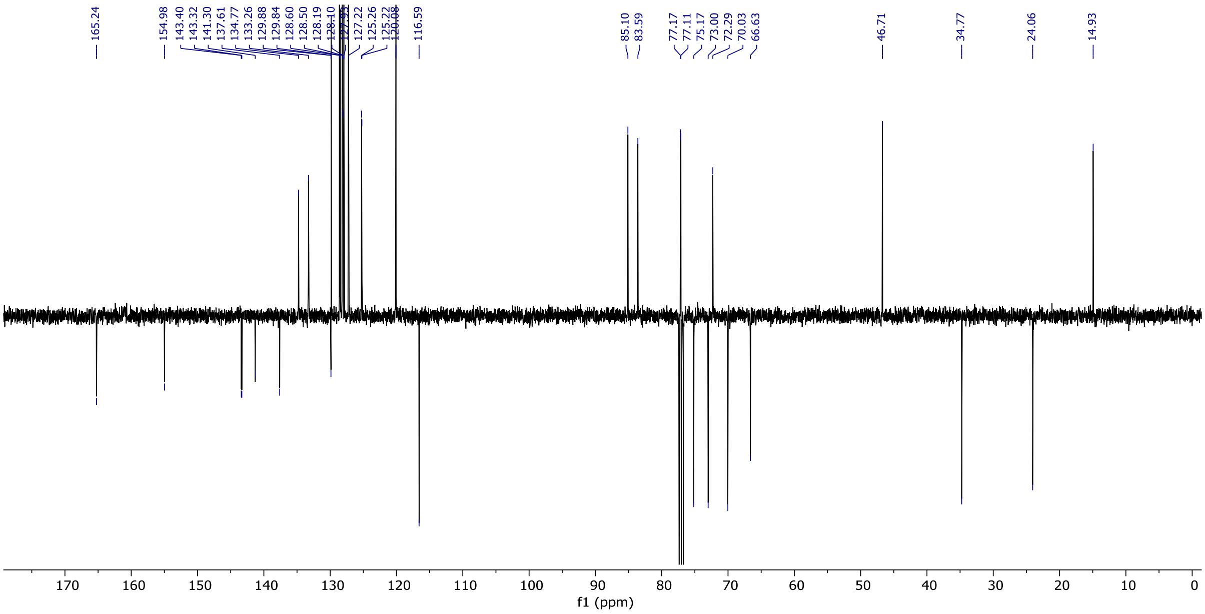

Figure S53. ^{13}C APT NMR (101 MHz, $CDCl_3$) spectrum of **3b-6**.

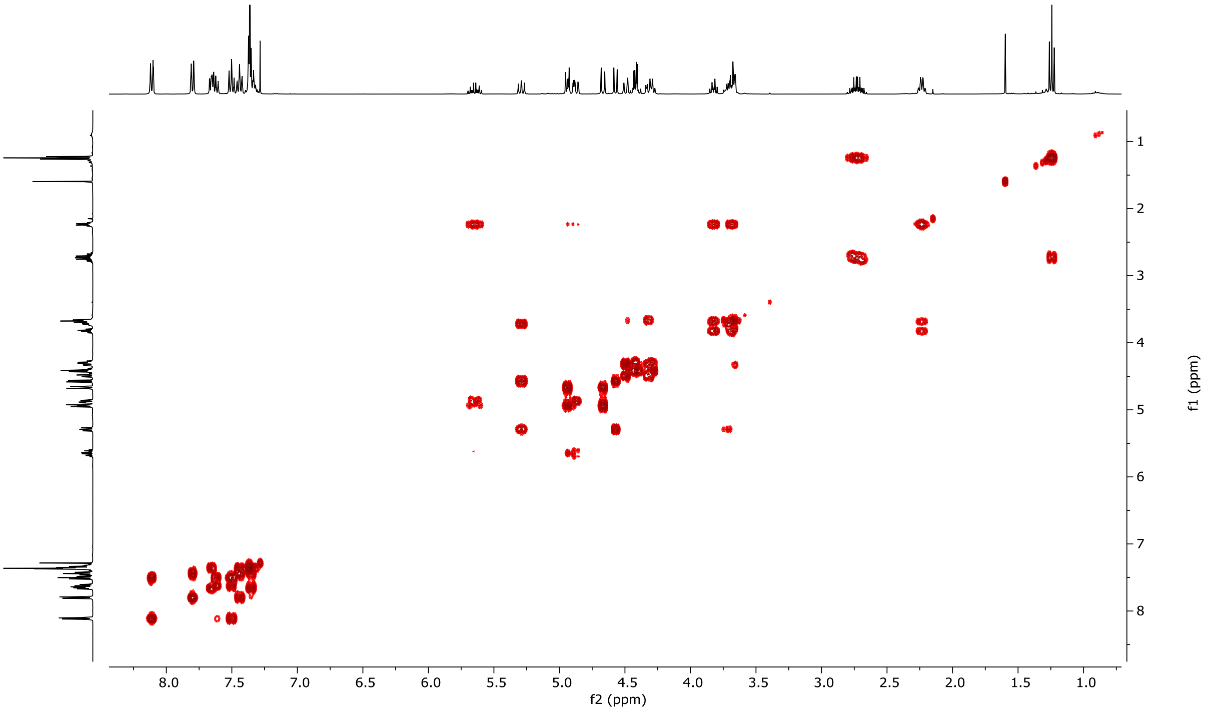

Figure S54. COSY NMR (400 MHz, $CDCl_3$) spectrum of **3b-6**.

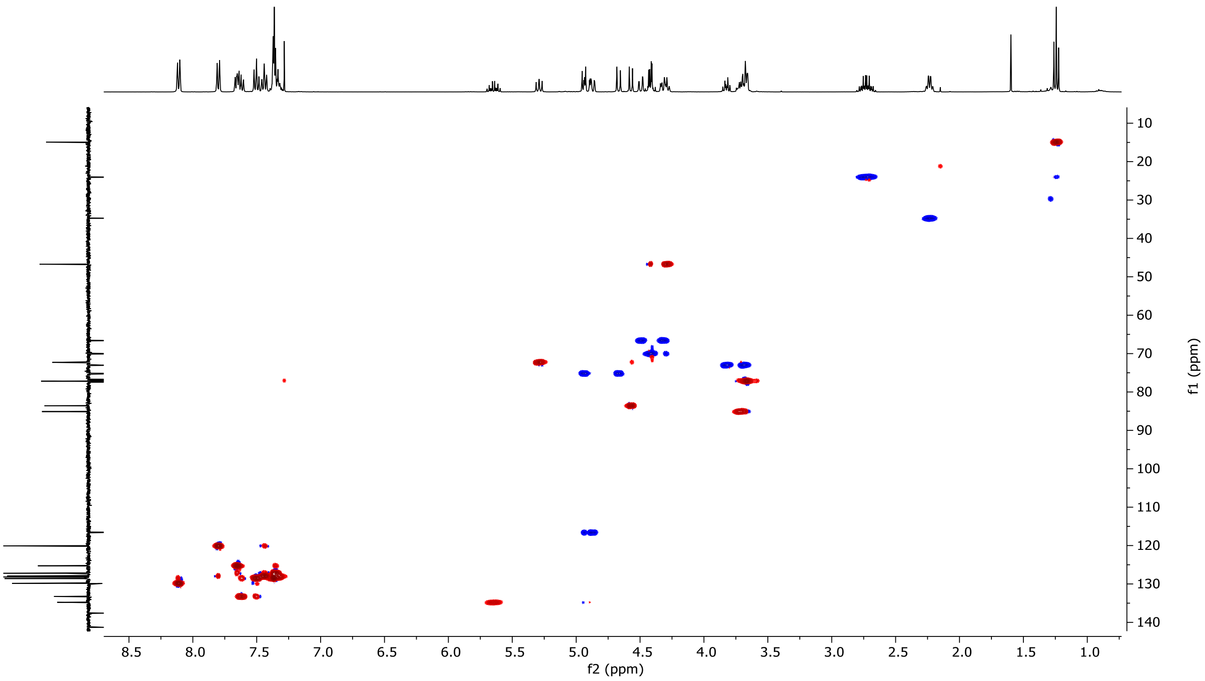

Figure S55. HSQC NMR (400MHz, $CDCl_3$) spectrum of **3b-6**.

*Ethyl 2-O-benzoyl-4-O-benzyl-6-O-fluorenylmethoxycarbonyl-3-O-(pent-4-en-yl)-1-thio-β-D-glucopyranoside (**3c-6**)*

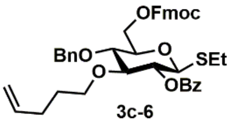

3c-6

3c-5 (1.18 g, 2.4 mmol), pyridine (0.38 mL, 4.8 mmol) and BzCl (0.74 mL, 2.9 mmol) are allowed to react in DCM (30 mL) according to the general procedure detailed above to afford **3c-6** (1.07 g, 62% yield) as a white solid. R*f* (hex/EA 4:1) = 0.53. HR-MS *m/z* = 731.2631 $[M+Na]^+$, calcd for $C_{42}H_{44}NaO_8S$: 731.2655. ^1H NMR (400 MHz, $CDCl_3$), δ = 8.14 – 8.08 (m, 2H), 7.80 (dp, *J* = 7.6, 1.0 Hz, 3H), 7.68 – 7.58 (m, 4H), 7.54 – 7.47 (m, 2H), 7.43 (dq, *J* = 7.5, 1.3 Hz, 3H), 7.40 – 7.32 (m, 8H), 5.60 (ddt, *J* = 16.9, 10.2, 6.6 Hz, 1H), 5.28 (dd, *J* = 10.0, 8.7 Hz, 1H), 5.08 – 4.97 (m, 1H), 4.93 (d, *J* = 10.9 Hz, 1H), 4.87 – 4.76 (m, 2H), 4.67 (d, *J* = 11.1 Hz, 1H), 4.57 (d, *J* = 10.0 Hz, 1H), 4.51 – 4.47 (m, 1H), 4.45 – 4.37 (m, 3H), 4.34 – 4.24 (m, 3H), 3.80 (ddd, *J* = 9.2, 6.3, 2.8 Hz, 1H), 3.72 – 3.62 (m, 4H), 2.81 – 2.65 (m, 2H), 2.05 – 1.94 (m, 1H), 1.92 – 1.84 (m, 1H), 1.70 (dddd, *J* = 10.9, 8.4, 6.5, 4.1 Hz, 1H), 1.61 – 1.55 (m, 3H), 1.24 (t, *J* = 7.4 Hz, 3H). ^{13}C NMR (101 MHz, $CDCl_3$), δ = 165.25, 154.98, 143.32, 141.30, 137.88, 137.58, 133.26, 129.85, 128.59, 128.49, 128.19, 128.11, 127.92, 127.21, 125.26, 125.21, 120.08, 114.65, 84.96, 84.69, 83.60, 77.21, 77.16, 75.15, 73.02, 72.31, 70.02, 66.64, 46.71, 30.15, 30.03, 29.54, 29.46, 24.07, 14.93.

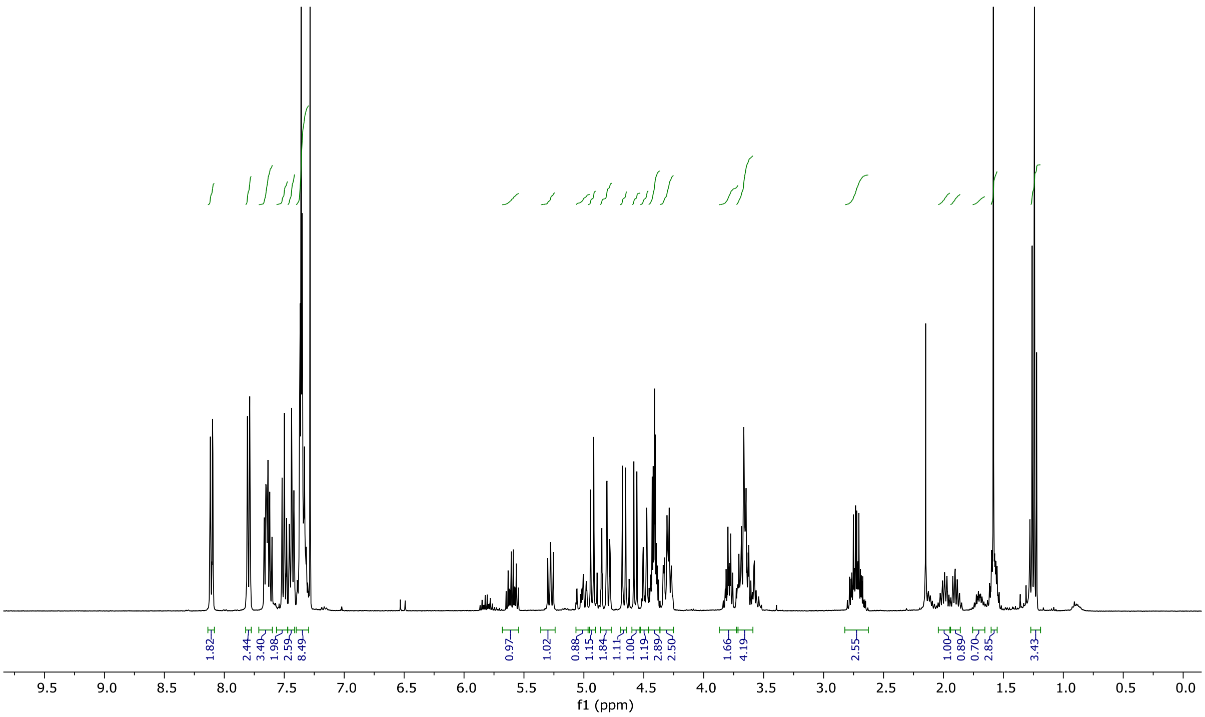

Figure S56. ^{1}H NMR (101 MHz, $CDCl_3$) spectrum of **3c-6**.

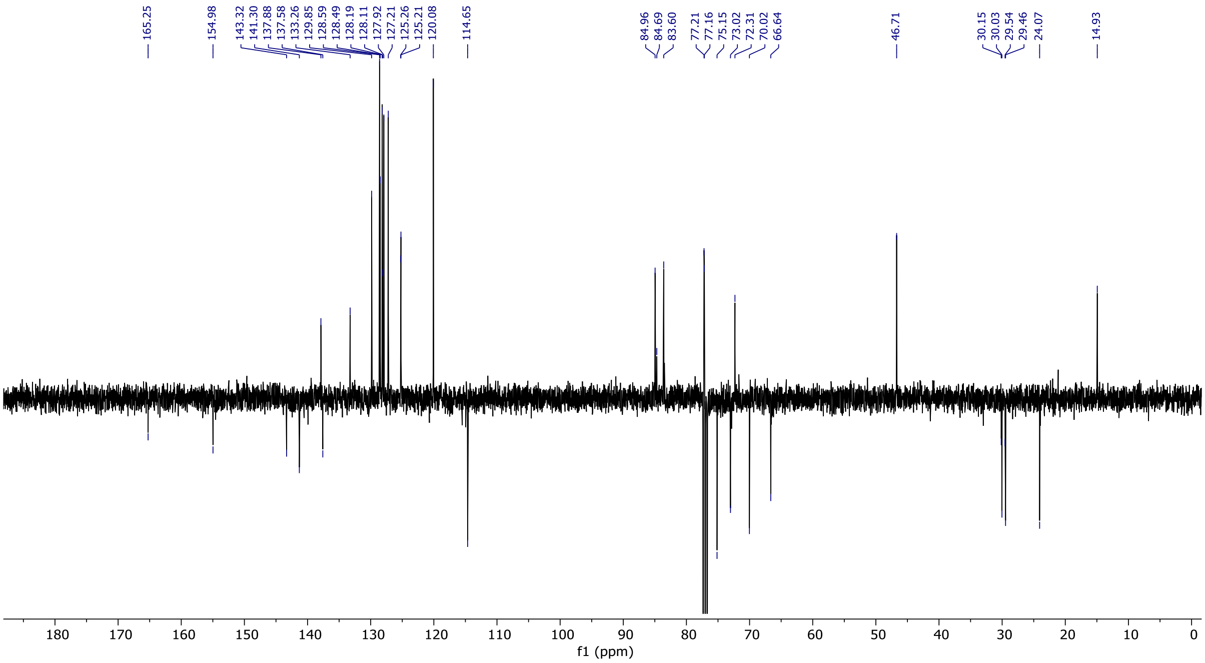

Figure S57. ^{13}C APT NMR (101 MHz, $CDCl_3$) spectrum of **3c-6**.

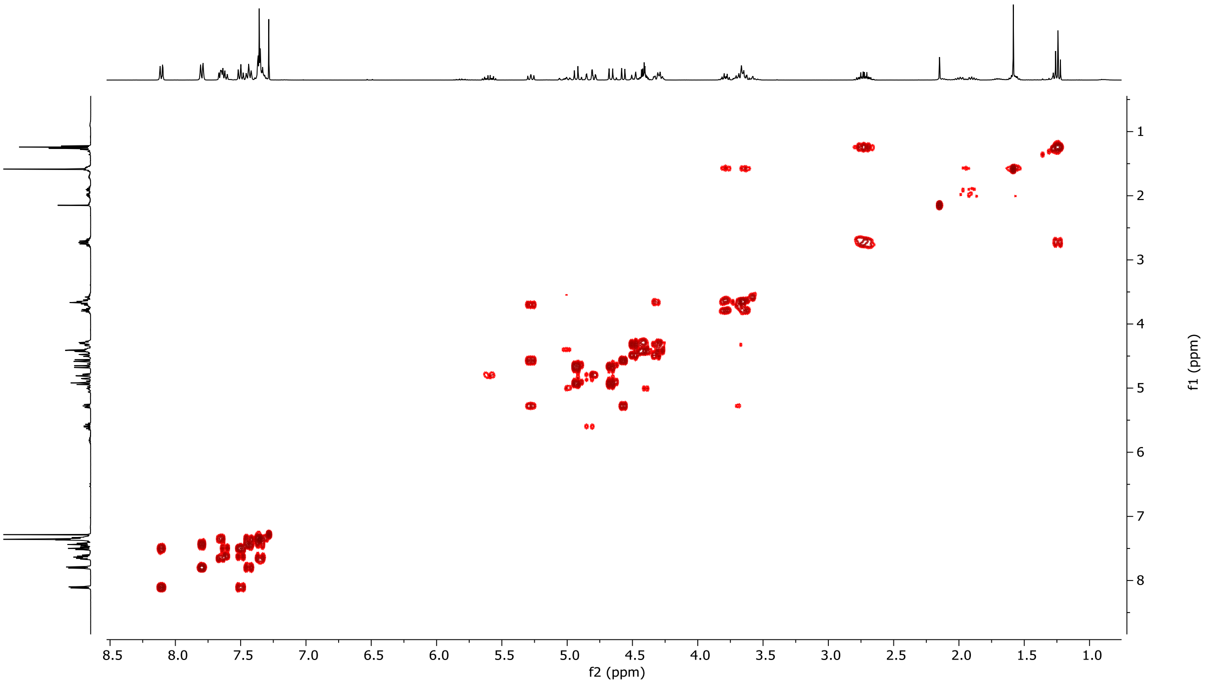

Figure S58. COSY NMR (400 MHz, $CDCl_3$) spectrum of **3c-6**.

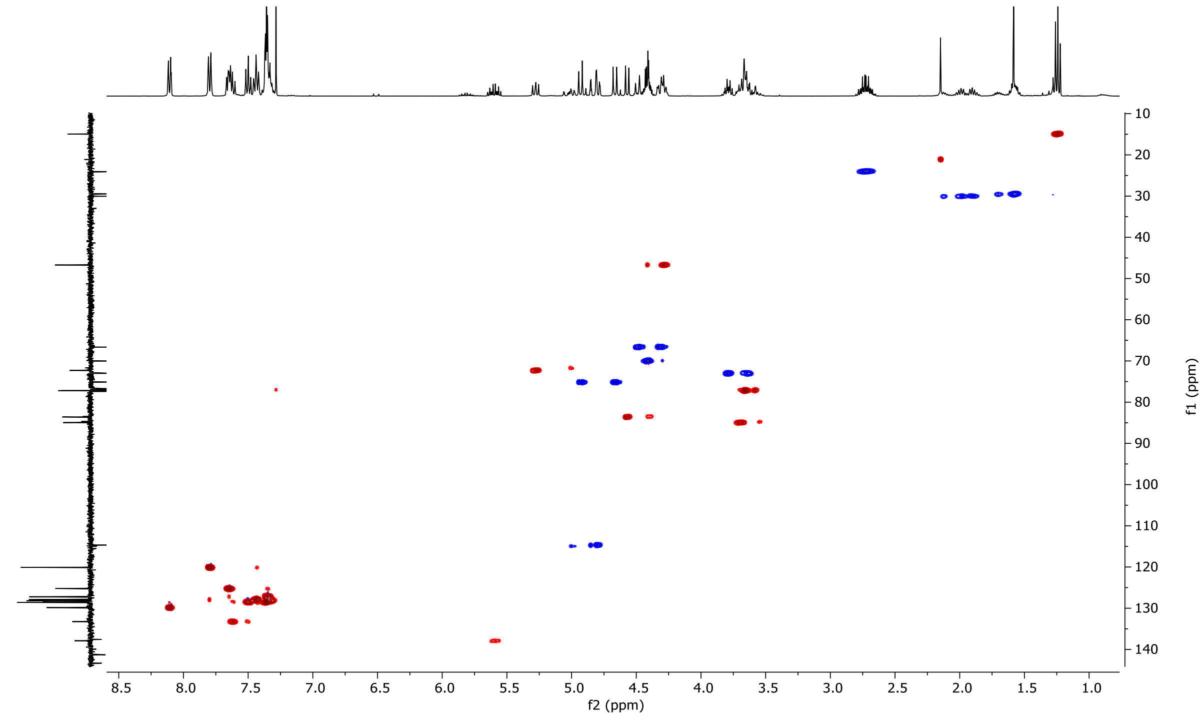

Figure S59. HSQC NMR (400MHz, $CDCl_3$) spectrum of **3c-6**.

\

*Ethyl 2-O-benzoyl-4-O-benzyl-6-O-fluorenylmethoxycarbonyl-3-O-(hext-5-en-yl)-1-thio-β-D-glucopyranoside (**3d-6**)*

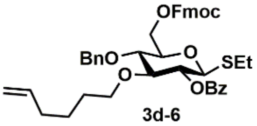

3d-5 (1.13 g, 2.26 mmol), pyridine (0.36 mL, 4.5 mmol) and BzCl (0.70 mL, 2.7 mmol) are allowed to react in DCM (30 mL) according to the general procedure detailed above to afford **3d-6** (1.24 g, 76% yield) as a white solid. R*f* (hex/EA 4:1) = 0.55. HR-MS *m/z* = 745.2819 [M+Na]$^+$, calcd for $C_{43}H_{46}NaO_8S$: 745.2811. ^{1}H NMR (400 MHz, CDCl), δ = 8.15 – 8.08 (m, 2H), 7.83 – 7.79 (m, 2H), 7.69 – 7.58 (m, 3H), 7.54 – 7.48 (m, 2H), 7.46 – 7.41 (m, 2H), 7.39 – 7.31 (m, 6H), 5.69 – 5.57 (m, 1H), 5.31 – 5.24 (m, 1H), 4.93 (d, *J* = 10.9 Hz, 1H), 4.89 – 4.82 (m, 2H), 4.67 (d, *J* = 11.0 Hz, 1H), 4.59 (s, 0H), 4.49 (dd, *J* = 11.5, 1.5 Hz, 1H), 4.45 – 4.40 (m, 2H), 3.79 (dt, *J* = 9.1, 6.2 Hz, 1H), 3.73 – 3.55 (m, 3H), 2.79 – 2.67 (m, 2H), 1.86 (dddd, *J* = 7.9, 6.8, 5.5, 1.2 Hz, 2H), 1.55 – 1.42 (m, 2H), 1.36 – 1.26 (m, 0H), 1.23 (t, *J* = 7.4 Hz, 3H). ^{13}C NMR (101 MHz, $CDCl_3$), δ = 165.23, 154.98, 143.40, 143.32, 141.30, 138.48, 137.61, 133.26, 129.85, 128.59, 128.49, 128.17, 128.10, 127.92, 127.22, 125.26, 125.22, 120.08, 114.45, 84.94, 83.59, 77.23, 77.17, 75.14, 73.53, 72.29, 70.02, 66.65, 46.71, 33.34, 29.80, 25.26, 24.05, 14.94.

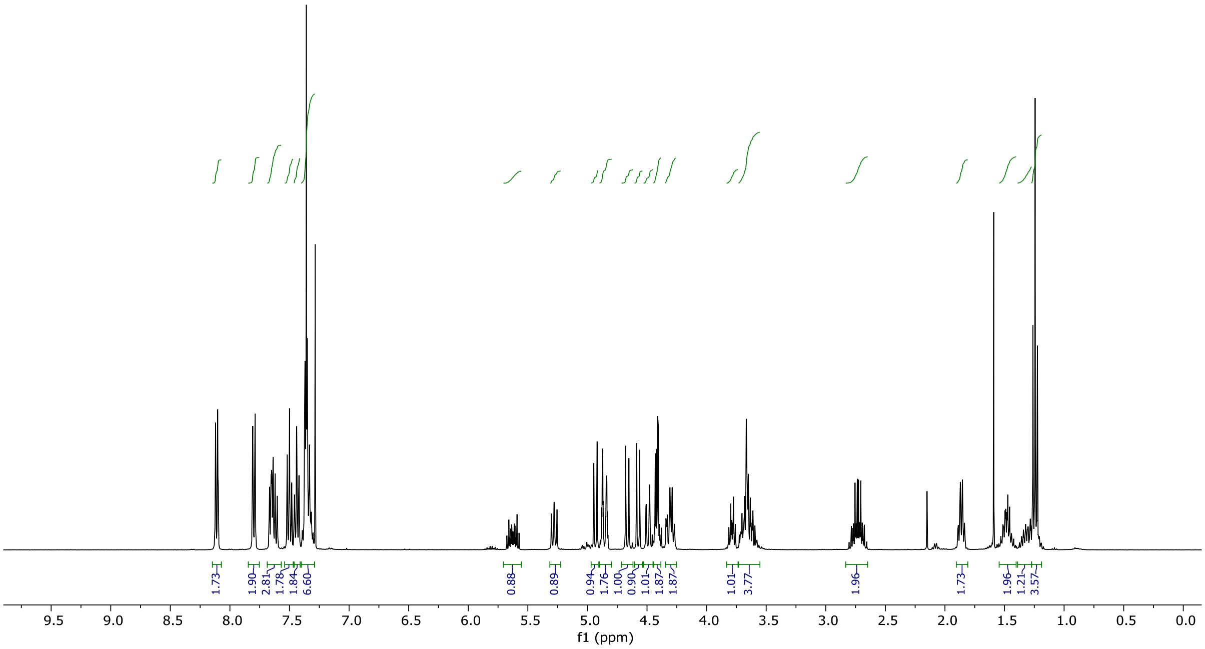

Figure S60. ^{1}H NMR (101 MHz, $CDCl_3$) spectrum of **3d-6**.

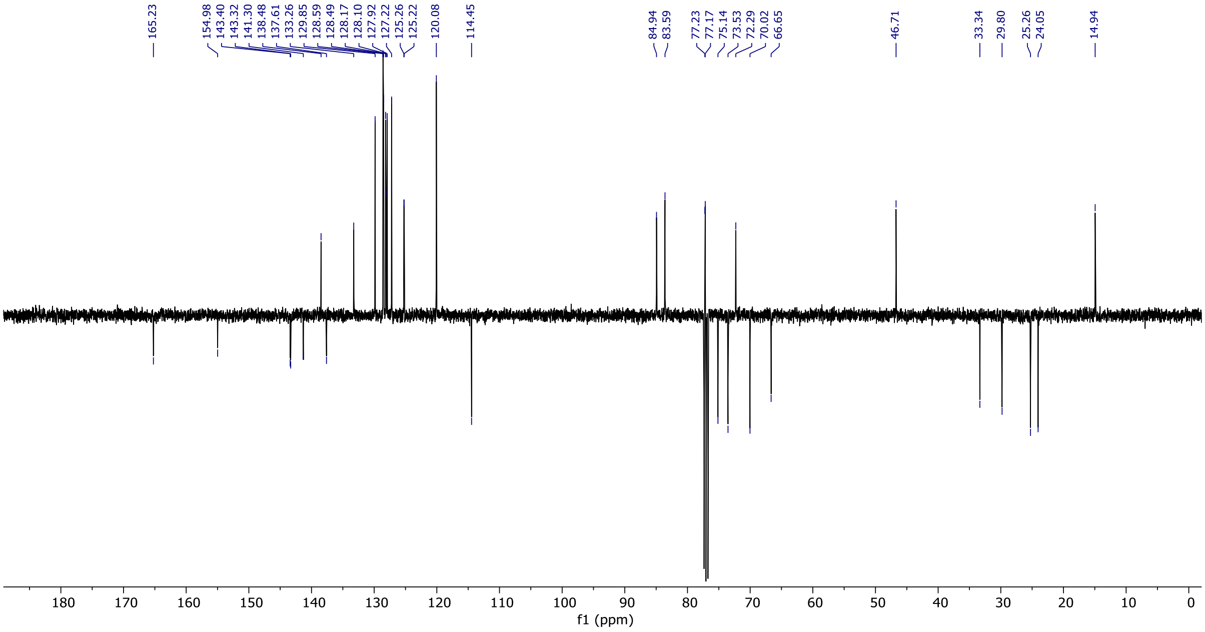

Figure S61. ^{13}C APT NMR (101 MHz, $CDCl_3$) spectrum of **3d-6**.

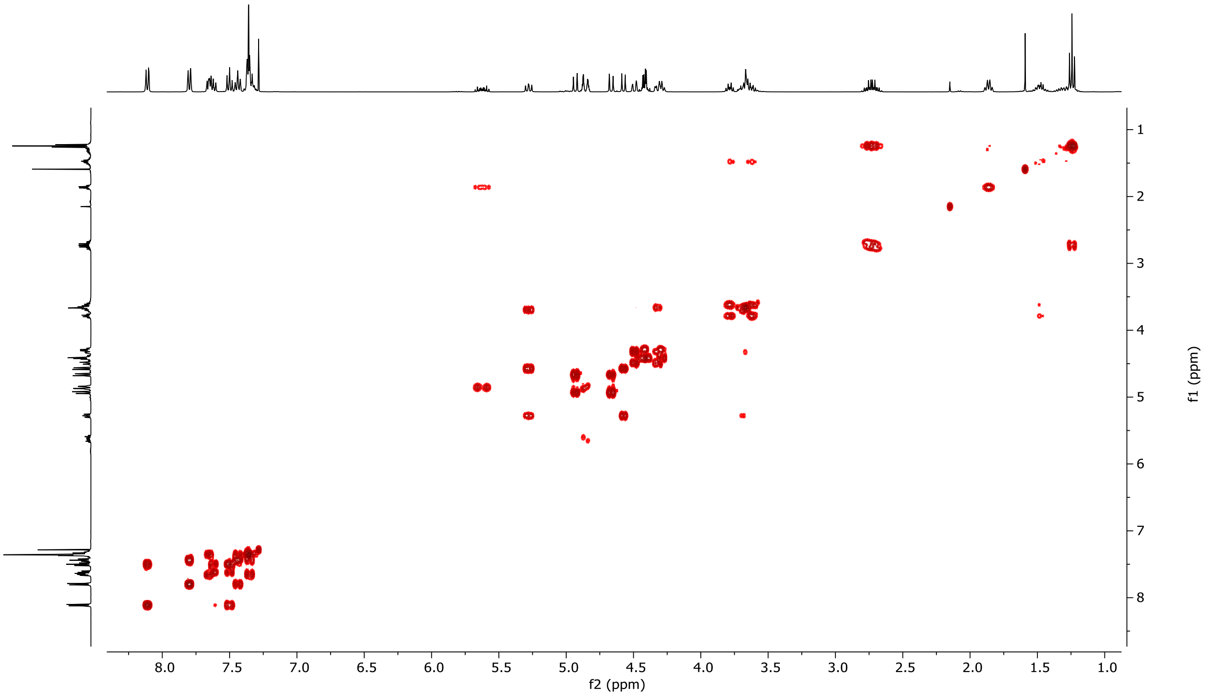

Figure S62. COSY NMR (400 MHz, $CDCl_3$) spectrum of **3d-6**.

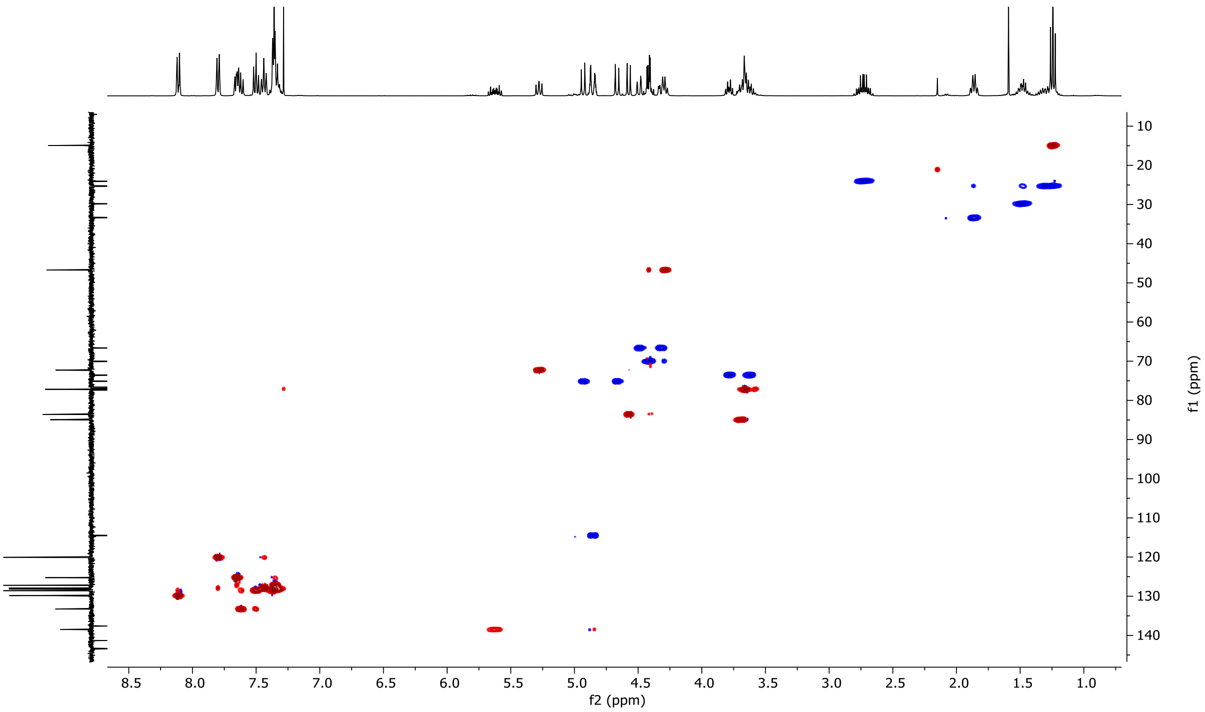

Figure S63. HSQC NMR (400MHz, $CDCl_3$) spectrum of **3d-6**.

*Dibutylphosphoryloxy 2,3-di-O-benzoyl-4-O-benzyl-6-O-fluorenylmethoxycarbonyl-β-D-glucopyranoside (**4b**)*

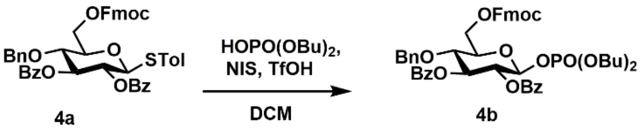

Similar to a previous report,[8] **4a** (2.17 g, 2.68 mmol) is coevaporated with toluene and dried in high vacuum overnight. It is dissolved in anhydrous DCM (40 mL), and under an argon atmosphere, dibutylphosphate (1.06 mL, 5.37 mmol) and 4Å molecular sieves are added. The suspension is stirred for 1 h and then placed in an ice bath. NIS (1.0 g, 4.02 mmol) and TfOH (24 µL, 0.27 mmol) are added, and the mixture is stirred at 0 °C for 4 h. The reaction is carefully quenched with a 5% soln. of $NaHCO_3$ (10 mL), diluted with chloroform, allowed to reach room temperature and filtered out. The mixture is sequentially washed with a 10% soln. of $Na_2S_2O_3$ (20 mL) and brine (20 mL). The organic layer is dried over Na_2SO_4 and concentrated under reduced pressure. The crude mixture is purified by column chromatography (Hex/EA 2:1) to afford the compound **4b** (1.94 g, 81%) as a sticky colourless solid. R*f* (hex/EA 2:1) = 0.25. ^1H NMR (400 MHz, $CDCl_3$), δ = 7.98 (ddd, *J* = 8.5, 3.2, 1.3 Hz, 4H), 7.85 – 7.77 (m, 2H), 7.68 (ddd, *J* = 7.3, 4.8, 1.0 Hz, 2H), 7.59 – 7.50 (m, 2H), 7.48 – 7.35 (m, 9H), 7.23 – 7.11 (m, 5H), 5.78 (t, *J* = 9.3 Hz, 1H), 5.60 – 5.48 (m, 2H), 4.66 – 4.55 (m, 3H), 4.52 – 4.37 (m, 3H), 4.32 (t, *J* = 7.3 Hz, 1H), 4.12 – 3.98 (m, 3H), 3.93 (ddd, *J* = 9.8, 3.9, 2.0 Hz, 1H), 3.85 – 3.68 (m, 2H),

1.67 – 1.53 (m, 2H), 1.43 – 1.24 (m, 5H), 1.13 – 0.99 (m, 2H), 0.88 (t, *J* = 7.4 Hz, 3H). ^{13}C NMR (101 MHz, $CDCl_3$), δ = 165.45, 165.19, 154.87, 143.36, 143.22, 141.33, 136.67, 133.47, 133.39, 129.91, 129.82, 129.17, 128.85, 128.49, 128.45, 128.36, 128.18, 127.99, 127.29, 127.26, 125.22, 125.16, 120.13, 96.44, 96.39, 75.09, 74.91, 74.58, 73.79, 71.88, 71.79, 70.18, 68.09, 68.03, 67.95, 67.89, 65.62, 46.72, 32.04, 31.97, 31.82, 31.75, 18.55, 18.26, 13.55, 13.39. ^{31}P NMR (162 MHz, $CDCl_3$), δ = -2.80.

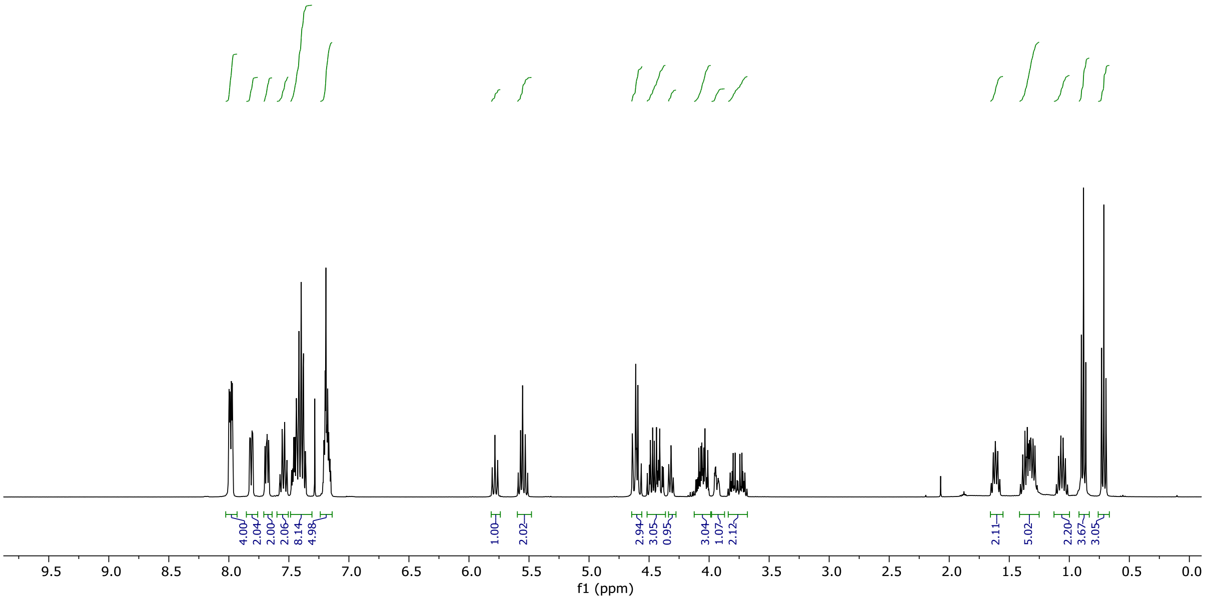

Figure S64. ^{1}H NMR (400 MHz, $CDCl_3$) spectrum of **4b**.

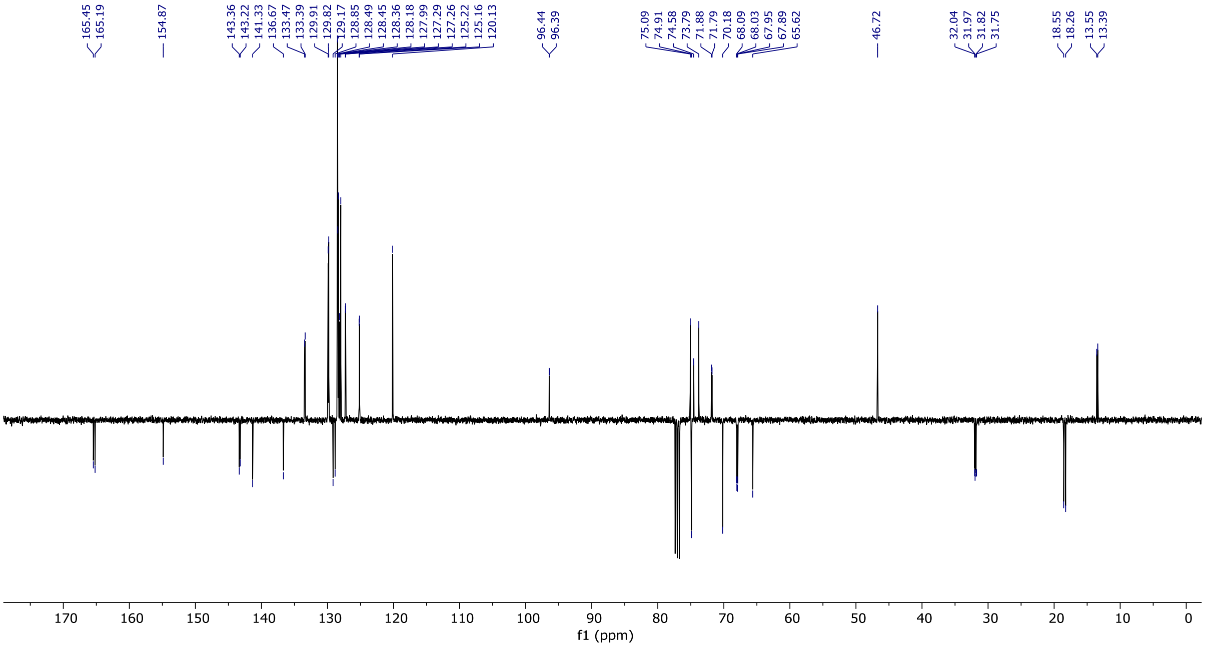

Figure S65. ^{13}C APT NMR (101 MHz, $CDCl_3$) spectrum of **4b**.

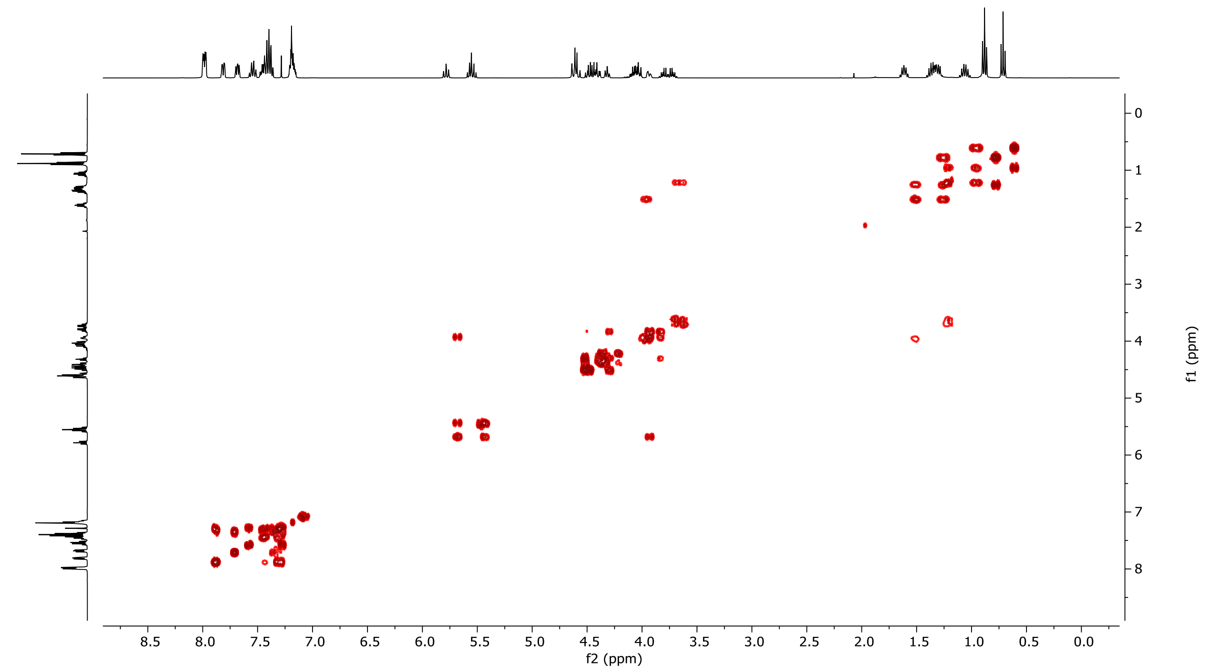

Figure S66. COSY NMR (400 MHz, $CDCl_3$) spectrum of **4b**.

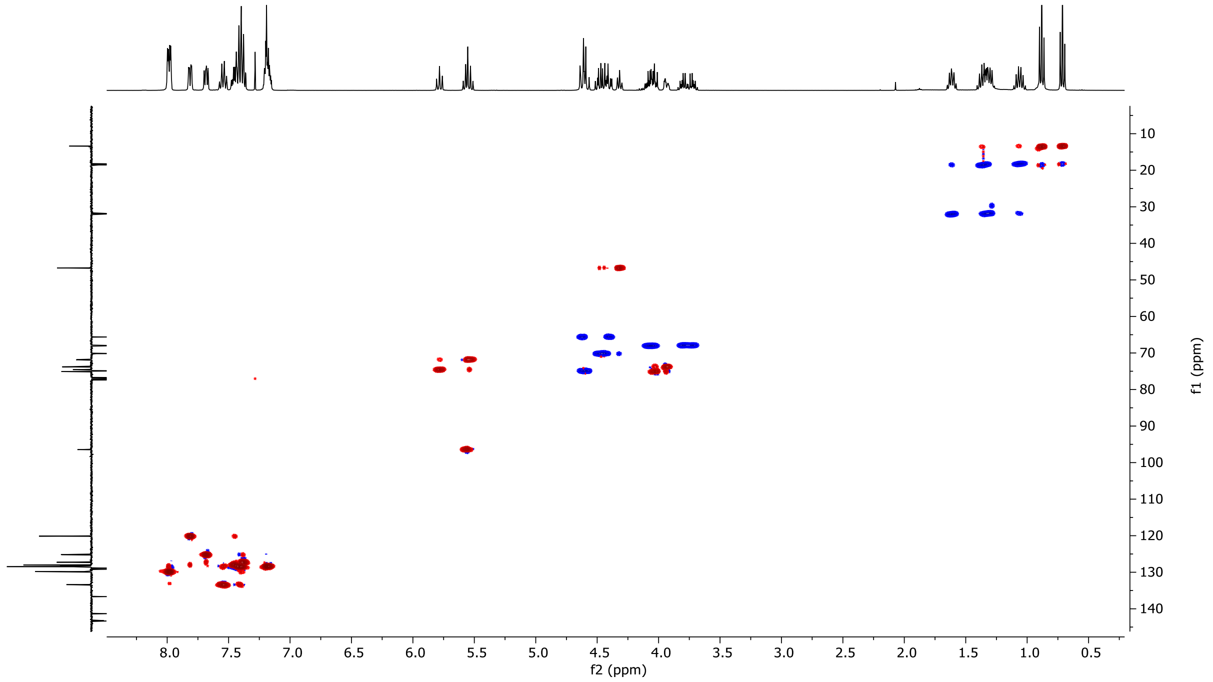

Figure S67. HSQC NMR (400MHz, $CDCl_3$) spectrum of **4b**.

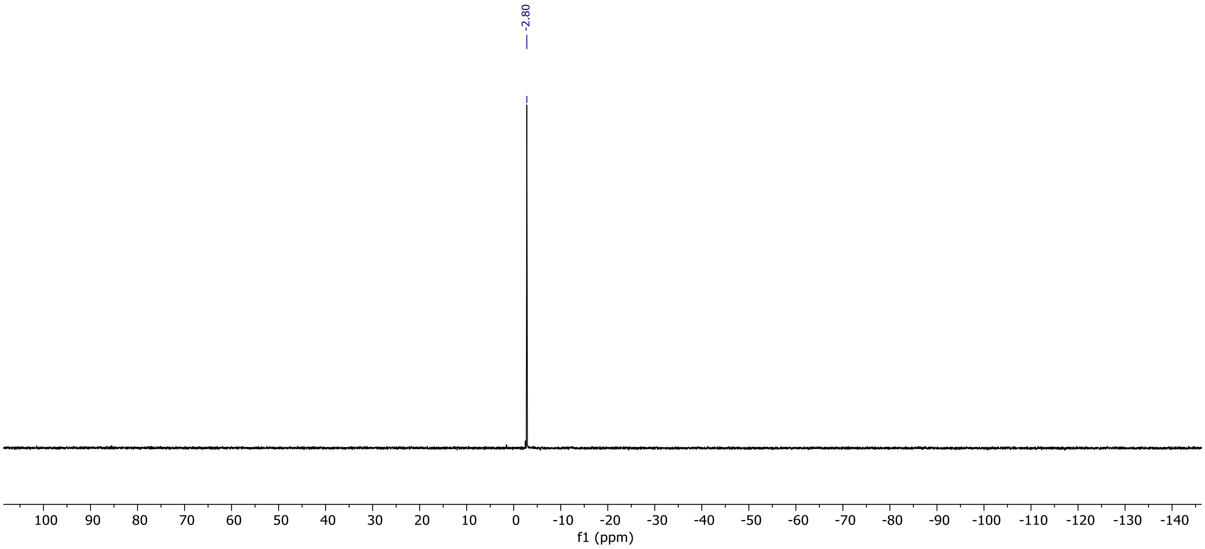

Figure S68. ^{31}P NMR (162 MHz, $CDCl_3$) spectrum of **4b**.

3. Automated glycan assembly

Solvents used for dissolving building blocks, activator, acid wash (TMSOTf), and capping solutions were taken from an anhydrous solvent system (JC Meyer-solvent systems). Other solvents used were HPLC grade. The building blocks were co-evaporated three times with toluene and dried for 2 h under a high vacuum before use. All solutions were freshly prepared and kept under argon during the automation run. Final yields were calculated based on the resin loading. Resin loading was determined by performing one glycosylation (Module C) followed by DBU-promoted Fmoc-cleavage and determination of dibenzofulvene formation by measuring its UV absorbance.[3,9]

3.1. Preparation of reagent solutions

- **Building block solution**: Building block (0.09 mmol) was dissolved in DCM (1 mL), for **4b** 0.06 mmol were dissolved in DCM (1 mL).
- **NIS/TfOH activator solution A**: Recrystallized NIS (1.35 g, 6.0 mmol) was dissolved in 40 mL of a 2:1 v/v mixture of anhydrous DCM and anhydrous dioxane. Then, triflic acid (55 µL, 0.6 mmol) was added. The solution is kept at 0°C for the duration of the automation run.
- **NIS/TfOH activator solution B**: Recrystallized NIS (0.79 g, 3.2 mmol) of was dissolved in 40 mL of a 2:1 v/v mixture of anhydrous DCM and anhydrous dioxane. Then, triflic acid (55 µL, 0.6 mmol) was added. The solution is kept at 0°C for the duration of the automation run.
- **Fmoc deprotection solution**: A solution of 20% piperidine in DMF (v/v) was prepared.
- **TMSOTf solution**: TMSOTf (0.45 mL, 2.49 mmol) was dissolved in DCM (40 mL).
- **Capping solution**: A 50 mL solution of 10% acetic anhydride and 2% methanesulfunic acid in DCM (v/v) was prepared.
- **Grubbs 1st gen. solution**: Grubbs catalyst 1st generation (25 mg, 30 µmol) was dissolved in previously degassed DCE (20 mL).
- **Grubbs 2st gen. solution**: Grubbs catalyst 2nd generation (26 mg, 30 µmol) was dissolved in previously degassed DCE (20 mL).

3.2. Modules for Automated Solid-Phase Synthesis

Module A: Resin preparation for synthesis

All automated syntheses were performed on a 0.015 mmol scale. Resin (**1**, 43 mg, 0.35 mmol/g) was placed in the reaction vessel and swollen in DCM for 20 min at room temperature before synthesis. During this time, all reagent lines needed for the synthesis were washed and primed. After swelling, the resin was washed with DMF, THF, and DCM (three times each with 2 mL for 25 s).

Module B: Acidic wash (TMSOTf solution)

The resin was swollen in DCM (2 mL) and the temperature of the reaction vessel was adjusted to -20 °C. Upon reaching the low temperature, TMSOTf solution (1 mL, 0.06 mmol) was added dropwise to the reaction vessel. After bubbling for 3 min, the acidic solution was drained and the resin was washed with DCM (2 mL) for 25 s.

Action	Cycles	Solution	Amount	T (°C)	Incubation time
Cooling	-	-	-	-20	-
Deliver	1	DCM	2 mL	-20	-
Deliver	1	TMSOTf solution	1 mL	-20	3 min
Wash	1	DCM	1 mL	-20	25 s

Module C1: Thioglycoside glycosylation × 1 cycle (NIS: 0.15 mmol/mL)

The standard thioglycoside-based glycosylation conditions are used for building blocks **2a**, **3a**, and **4a** as previously reported.[10] The building block solution (0.09 mmol of BB in 1 mL of DCM per glycosylation) was delivered to the reaction vessel. After the set temperature was reached, the reaction was started by dropwise addition of the activator solution (1.0 mL, 0.15 mmol). After completion of the reaction, the solution is drained and the resin was washed with DCM, DCM/dioxane (1:2, v/v, 3 mL for 20 s), and DCM (twice, each with 2 mL for 25 s). The temperature of the reaction vessel is increased to 25°C for the next module.

Action	Cycles	Solution	Amount	T (°C)	Incubation time
Cooling	-	-	-	-20	~15 min
Deliver	1	BB solution	1 mL	-20	-
Deliver	1	Activator solution A	1 mL	-20	3 min
Reaction time 1	1	-	-	-20	10 min

Reaction time 2	1	-	-	0	30 min
Wash	1	DCM	2 mL	0	5 s
Wash	1	DCM / Dioxane 2:1	2 mL	0	20 s
Heating	-	-	-	25	-
Wash	2	DCM	2 mL	>0	25 s

Module C2: Thioglycoside glycosylation × 2 cycles (NIS: 0.09 mmol/mL)

These glycosylation conditions are used for building blocks containing alkene chains longer than allyl (**2b and 3b-d**). The building block solution (0.09 mmol of BB in 1 mL of DCM per glycosylation) was delivered to the reaction vessel. After the set temperature was reached, the reaction was started by dropwise addition of the activator solution (1.0 mL, 0.09 mmol). After completion of the reaction, the solution is drained and the resin was washed with DCM, DCM/dioxane (1:2, v/v, 3 mL for 20 s), and DCM (twice, each with 2 mL for 25 s). The temperature of the reaction vessel is increased to 25°C for the next module.

Action	Cycles	Solution	Amount	T (°C)	Incubation time
Cooling	-	-	-	-25	-
Deliver	1	BB solution	1 mL	-25	-
Deliver	1	Activator solution B	1 mL	-25	3 min
Reaction time 1	1	-	-	-25	25 min
Reaction time 2	1	-	-	-10	10 min
Wash	1	DCM	2 mL	-10	5 s
Wash	1	DCM / Dioxane 2:1	2 mL	-10	20 s
Cooling	-	-	-	-25	15 min
Deliver	1	BB solution	1 mL	-25	-
Deliver	1	Activator solution B	1 mL	-25	3 min
Reaction time 1	1	-	-	-25	25 min
Reaction time 2	1	-	-	-10	10 min
Wash	1	DCM	2 mL	-10	5 s
Wash	1	DCM / Dioxane 2:1	2 mL	-10	20 s
Heating	-	-	-	25	-
Wash	2	DCM	2 mL	>0	25 s

Module C3: Phosphate glycosylation × 1 cycle

The standard phosphate-based glycosylation conditions are used only for building blocks **4b** as previously reported.[11] **4b** (0.06 mmol in 1 mL of DCM per glycosylation) was delivered to the reaction vessel. After the set temperature was reached, the reaction was started by dropwise addition of the TMSOTf solution (1.0 mL, 0.06 mmol). After completion of the reaction, the solution was drained and the resin was washed with DCM (six times, each with 2 mL for 25 s). The temperature of the reaction vessel was increased to 25 °C for the next module.

Action	Cycles	Solution	Amount	T (°C)	Incubation time
Cooling	-	-	-	-30	-
Deliver	1	BB solution	1 mL	-30	-
Deliver	1	TMSOTf solution	1 mL	-30	3 min
Reaction time 1	1	-	-	-30	5 min
Reaction time 2	1	-	-	-10	30 min
Wash	1	DCM	2 mL	0	5 s
Heating	-	-	-	25	-
Wash	6	DCM	2 mL	>0	25 s

Module D: Capping

The resin was washed twice with DMF (2 mL, 25 s) and the temperature of the reaction vessel was adjusted to 25 °C. Pyridine solution (2 mL, 10% in DMF) was delivered into the reaction vessel. After 1 min, the reaction solution was drained and the resin was washed with DCM (three times with 3 mL for 25 s). Capping solution (4 mL) was delivered into the reaction vessel. After 20 min, the reaction solution was drained and the resin was washed with DCM (three times with 3 mL for 25 s).

Action	Cycles	Solution	Amount	T (°C)	Incubation time
Heating	-	-	-	25	-
Wash	2	DMF	2 mL	25	25 s
Deliver	1	10% Py./DMF	2 mL	25	1 min
Wash	3	DCM	2 mL	25	25 s
Deliver	1	Capping solution	4 mL	25	20 min
Wash	3	DCM	2 mL	-20	25 s

Module E: Fmoc deprotection

The resin was washed with DMF (three times with 2 mL for 25 s) and the temperature of the reaction vessel was adjusted to 25 °C. 2 mL of Fmoc deprotection solution was delivered to the reaction vessel and kept under Ar bubbling. After 5 min, the reaction solution was drained and the resin was washed with DMF (three times with 2 mL for 25 s) and DCM (five times each with 2 mL for 25 s). The temperature of the reaction vessel was decreased to -20 °C for the next module.

Action	Cycles	Solution	Amount	T (°C)	Incubation time
Wash	3	DMF	2 mL	25	25 s
Deliver	1	Fmoc depr.	2 mL	25	5 min
Wash	3	DMF	2 mL	25	25 s
Wash	5	DCM	2 mL	25	25 s
Cooling	1	-	-	-20	-

Module F1: Ring-closing Metathesis RT (Method A)

The resin was washed with DCE (three times with 2 mL for 25 s) and the temperature of the reaction vessel was adjusted to 25 °C. Grubbs' 1st gen. catalyst solution (2 mL, 3 µmol, 20 mol% respect to the resin loading) was delivered to the reaction vessel and kept under Ar bubbling. After 30 min, DCE (1 mL) was added to the reaction vessel and kept under Ar bubbling for an additional 30 min. The reaction solution was drained and the resin was washed sequentially with DCE, DMF, THF and DCM (twice with 2 mL for 25 s each.

Action	Cycles	Solution	Amount	T (°C)	Incubation time
Wash	3	DCE	2 mL	25	25 s
Deliver	1	Grubbs 1st gen.	2 mL	25	-
Reaction time	1	-	-	25	30 min
Deliver	1	DCE	1 mL	25	-
Reaction time	1	-	-	25	30 min
Wash	2	DCE	2 mL	25	25 s
Wash	2	DMF	2 mL	25	25 s
Wash	2	THF	2 mL	25	25 s
Wash	2	DCM	2 mL	25	25 s

Module F2: Ring-closing Metathesis MW 60 °C (Method B)

This experiment was performed in a TempDUO home-built synthesizer developed as reported previously.[1] The resin was washed with DCE (three times with 2 mL for 25 s) and the temperature of the reaction vessel was adjusted to 60 °C by MW heating. Grubbss 2nd gen. catalyst solution (2 mL, 3 µmol, 20 mol% respect to the resin loading) was delivered to the reaction vessel and kept under Ar bubbling at 60 °C. After 20 min, DCE (1 mL) was added to the reaction vessel and kept under Ar bubbling at 60 °C for an additional 20 min. The reaction solution was drained and the resin was washed sequentially with DCE, DMF, THF, and DCM (twice with 2 mL for 25 s each).

Action	Cycles	Solution	Amount	T (°C)	Incubation time
Wash	3	DCE	2 mL	25	25 s
Deliver	1	Grubbs 2st gen.	2 mL	60	-
Reaction time	1	-	-	60	20 min
Deliver	1	DCE	1 mL	60	-
Reaction time	1	-	-	60	20 min
Wash	2	DCE	2 mL	25	25 s
Wash	2	DMF	2 mL	25	25 s
Wash	2	THF	2 mL	25	25 s
Wash	2	DCM	2 mL	25	25 s

3.3. Post-solid-phase manipulations

Module G: Cleavage from the solid support

Oligosaccharides were cleaved from the solid support using a continuous-flow photoreactor. The resin-bound glycan (~50 mg) was suspended in DCM (10 mL) and passed through the photoreactor (Mercury lamp, 450W) at 0.5 mL/min and RT, as described previously.[9] For stapled oligosaccharides, due to slight darkening of the resin after RCM, it was required an additional photocleavage cycle. For that, the resin is collected, suspended in DCM (10 mL) and passed through the photoreactor one more time. The two DCM solutions were collected and evaporated under reduced pressure to afford the crude stapled glycan.

Module H: Methanolysis

The protected oligosaccharide was dissolved in DCM (1.0 mL). A solution of NaOMe in MeOH (0.5 M, 1 mL) was added and the mixture was stirred at room temperature for 16 h. The reaction was neutralized with Amberlite IRC120H and concentrated *in vacuo*. The crude compound was used for the hydrogenolysis reaction without further purification.

Module I: Hydrogenolysis

The crude compound was dissolved in 2 mL of EA: *t*BuOH: H_2O (1:0.5:0.5). Pd-C (10%, 0.1 g) was added and the reaction was stirred under H_2 atmosphere for 12 h. The reaction was filtered through celite and washed with *t*BuOH and H_2O. The filtrates were concentrated *in vacuum*, and dissolved in 3.0 mL of water for RP-HPLC purification.

Module J: HPLC analysis and purification

Analytical traces of crude and pure compounds were collected using an analytic RP-HPLC Agilent 1200 Series (**Methods 1, 2**). Purification of the crudes was conducted using a preparative RP-HPLC Agilent 1200 Series (**Methods 3, 4**).

- **Method 1:** (Hypercarb column, 150 x 4.6 mm, 3 µm) flow rate of 0.7 mL/min with ACN/H_2O (0.1% formic acid) as eluents [isocratic 100 % H_2O (0.1% formic acid) (5 min), linear gradient to 100% ACN (30 min)].
- **Method 2:** (Phenomenex, luna C5 column, 250 x 4.6 mm, 5 µm), flow rate of 0.5 mL /min with ACN/H_2O (0.1% formic acid) as eluents [isocratic 5% ACN (5 min), linear gradient to 100% ACN (30 min)].
- **Method 3:** (Hypercarb column, 150 x 10 mm, 5 µm), flow rate of 3.5 mL /min with H_2O (0.1% formic acid) as eluents [isocratic 100 % H_2O (0.1% formic acid) (5 min), linear gradient to 100% ACN (30 min)].
- **Method 4:** (Phenomenex, Luna C5 column, 250 x 10 mm, 5 µm), flow rate of 4 mL /min with ACN/H_2O (0.1% formic acid) as eluents [isocratic 5% ACN (5 min), linear gradient to 100% ACN (5 min)].

Following final purification, all final products were lyophilized on a Christ Alpha 2-4 LD plus freeze dryer before characterization.

3.4 Oligosaccharide synthesis

Synthesis and analytical data of 8a

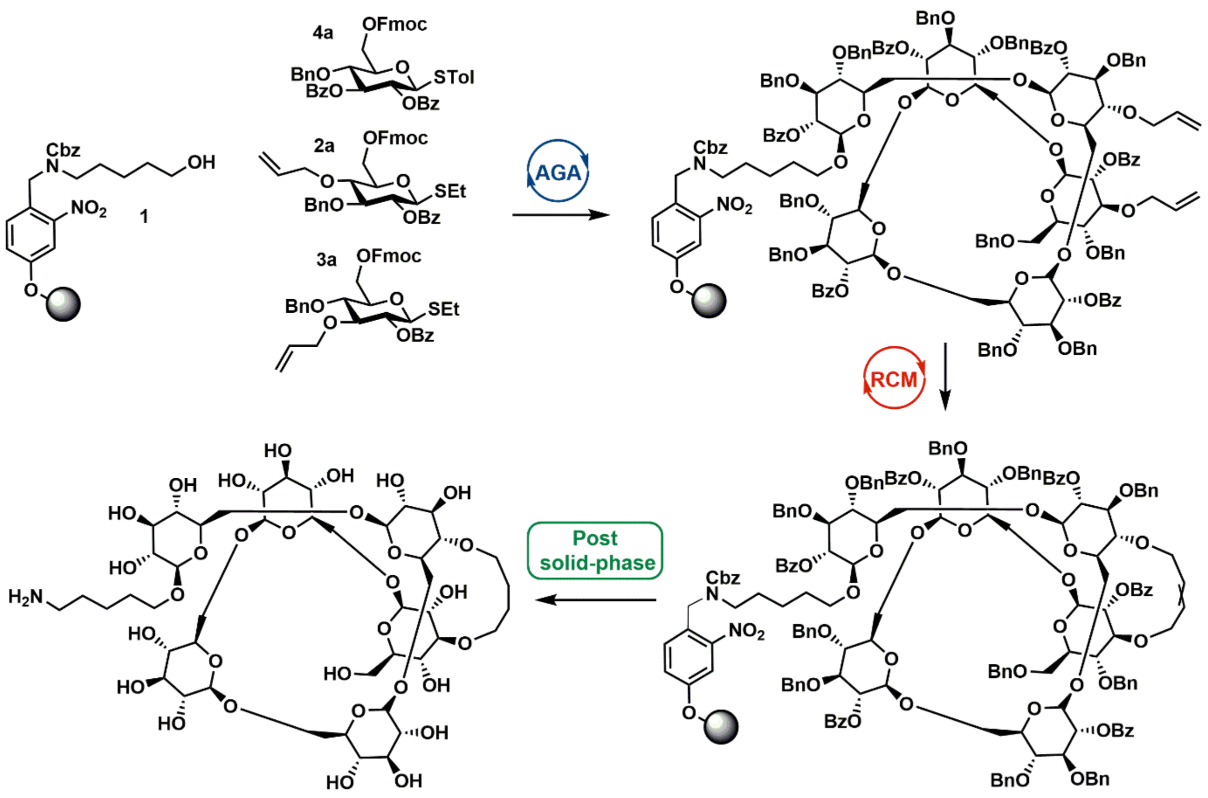

Step	Module	BB/reagent	Repeat	Notes
AGA	A	**1** (0.015 mmol)	1	Resin swelling
	B, C1, D, E	**4a** (0.09 mmol)	1	C1: NIS (0.15 mmol)
	B, C1, D, E	**2a** (0.09 mmol)	1	C1: NIS (0.15 mmol)
	B, C1, D, E	**4a** (0.09 mmol)	3	C1: NIS (0.15 mmol)
	B, C1, D, E	**3a** (0.09 mmol)	1	C1: NIS (0.15 mmol)
RCM	F2	Grubbs 2nd gen.	6	Method B (MW 60°C)
Post solid-phase	G	-	2	-
	H, I	-	-	-
	J	-	-	Methods 1 and 3

After automated glycan assembly, ring-closing metathesis, photo-cleavage, global deprotection, purification, and lyophilisation was obtained **8a** as a white solid (2.0 mg, 12%).

R_t (Method 1) = 19.2 min.

HR-MS *m/z* = 1130.4888 $[M+H]^+$, calcd for $C_{45}H_{80}NO_{31}$: 1130.4714.

^{1}H NMR (700 MHz, D_2O), δ = 4.49 – 4.42 (m, 6H), 4.39 (d, *J* = 8.1 Hz, 1H), 4.17 – 4.12 (m, 4H), 4.08 (dd, *J* = 11.6, 2.4 Hz, 1H), 3.89 (dd, *J* = 11.6, 4.4 Hz, 1H), 3.87 – 3.85 (m, 1H), 3.84 – 3.79 (m, 3H), 3.79 – 3.74 (m, 4H), 3.70 (dt, *J* = 9.3, 6.4 Hz, 1H), 3.65 (dd, *J* = 12.3, 5.8 Hz, 1H), 3.62 – 3.59 (m, 2H), 3.58 – 3.51 (m, 6H), 3.50 – 3.45 (m, 1H), 3.45 – 3.40 (m, 6H), 3.40 – 3.35 (m, 4H), 3.33 – 3.28 (m, 3H), 3.27 – 3.23 (m, 4H), 3.19 (t, *J* = 8.0 Hz, 1H), 1.66 – 1.55 (m, 8H), 1.41 – 1.35 (m, 2H).

^{13}C NMR (176 MHz, D_2O), δ = 103.35, 103.27, 102.94, 102.82, 102.23, 102.09, 83.46, 77.96, 75.82, 75.73, 75.55, 75.52, 75.37, 74.97, 74.92, 74.88, 74.30, 74.00, 73.13, 73.08, 72.68, 72.60, 71.33, 70.19, 69.47, 69.45, 69.06, 68.91, 68.91, 68.76, 68.50, 68.18, 67.98, 60.68, 39.37, 28.18, 26.42, 26.01, 25.81, 22.10.

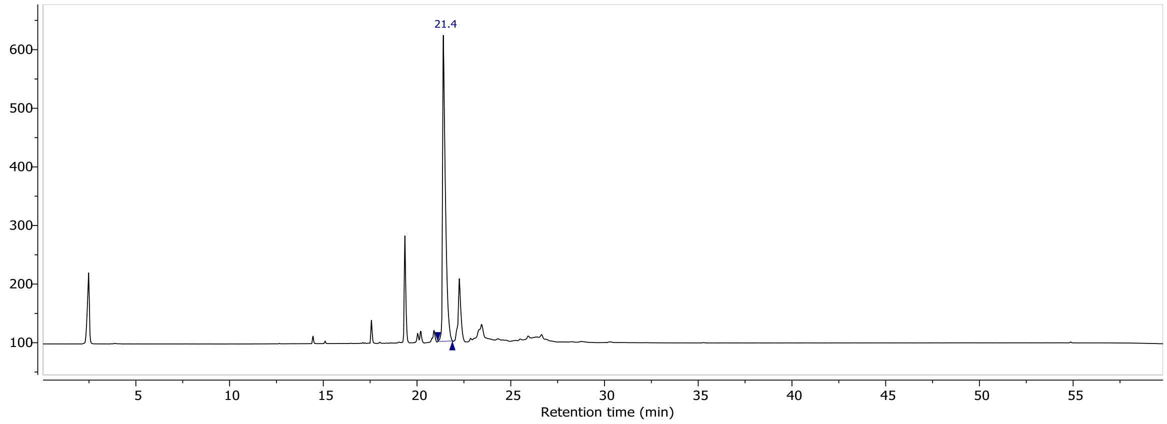

Figure S69. RP-HPLC trace of crude **8a**.

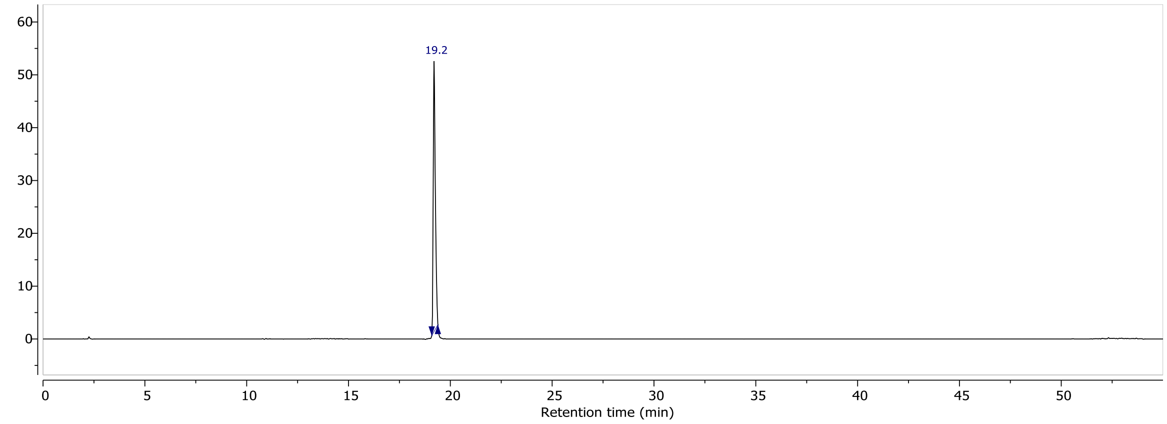

Figure S70. RP-HPLC trace of pure **8a**.

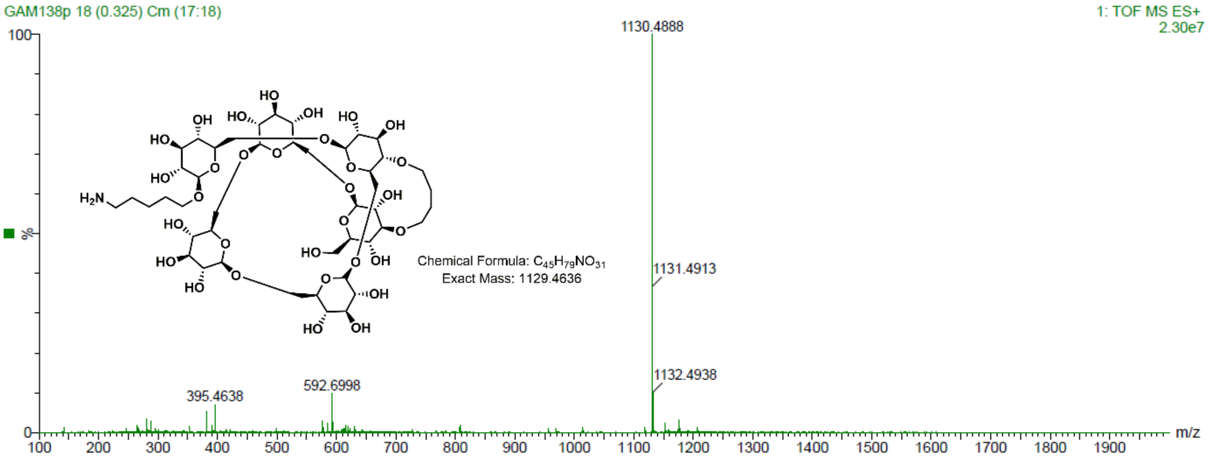

Figure S71. HR-MS of **8a**.

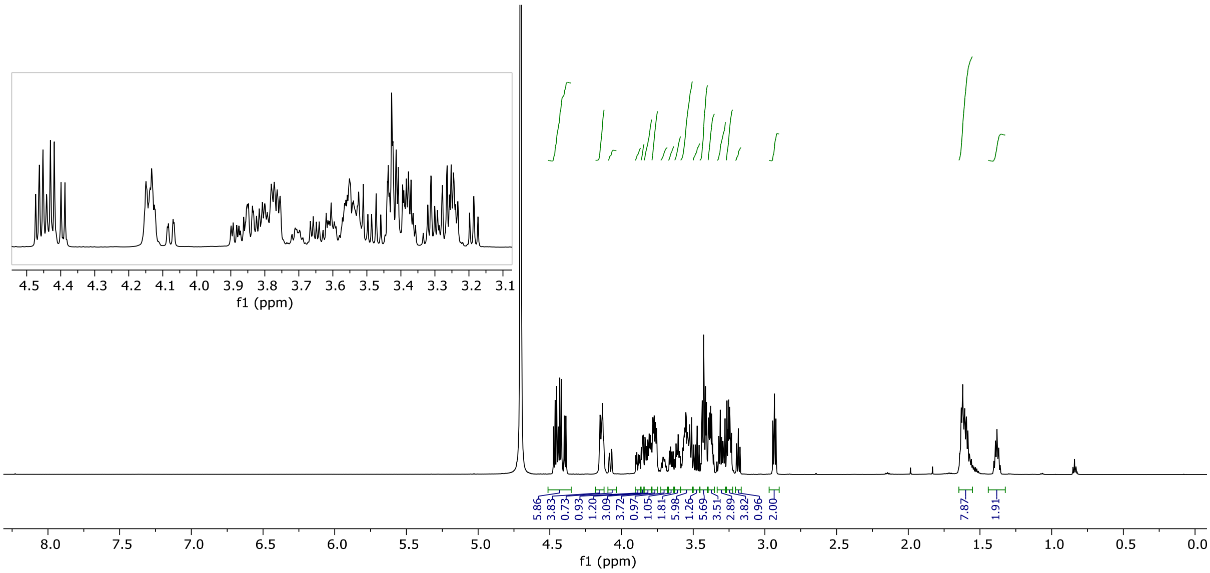

Figure 72 ^{1}H NMR (700 MHz, D_2O) spectrum of **8a**.

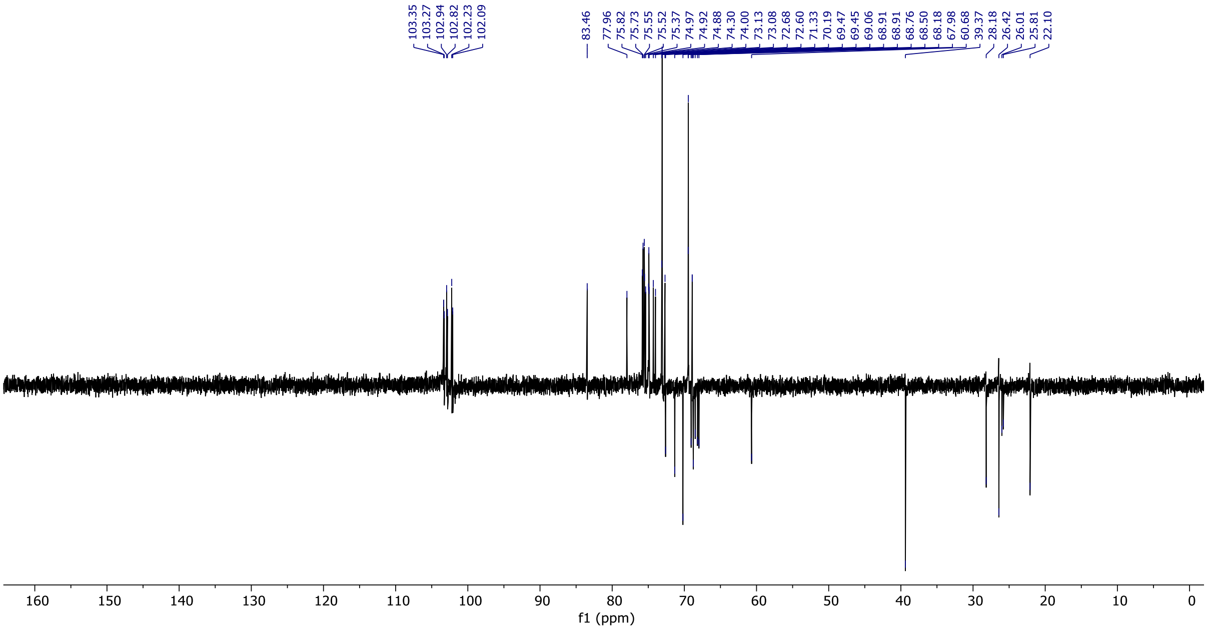

Figure S73. ^{13}C-APT NMR (700 MHz, D_2O) spectrum of **8a**.

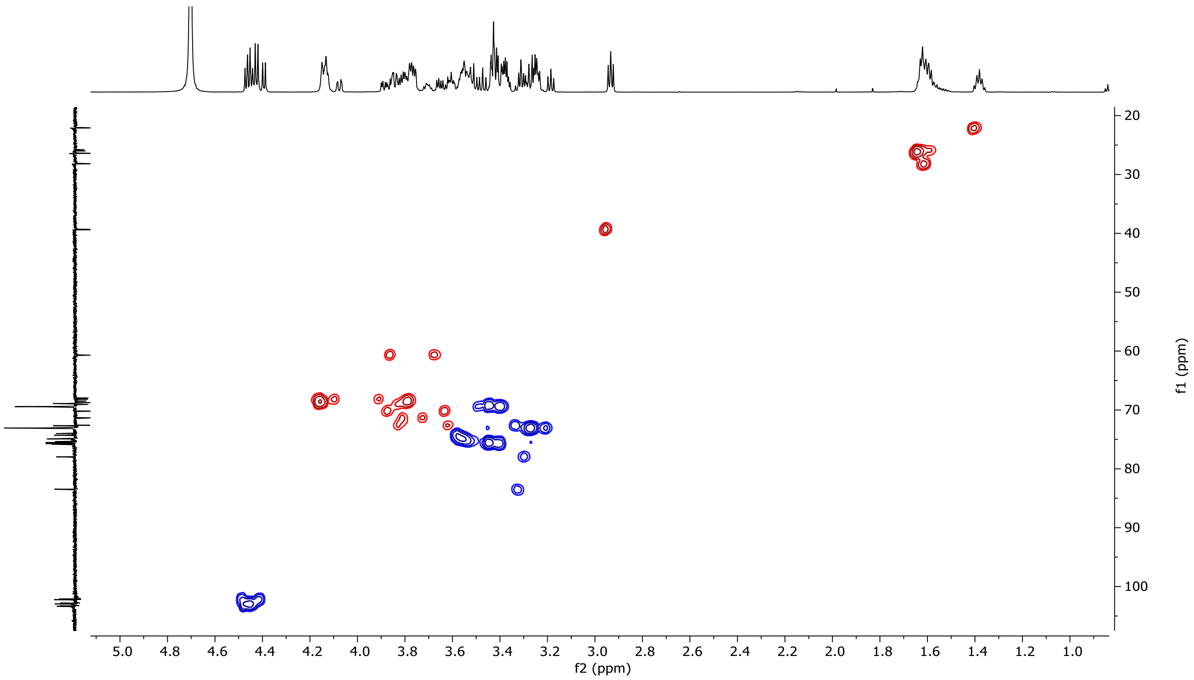

Figure S74. HSQC (700 MHz, D_2O) spectrum of **8a**.

Synthesis and analytical data of 8b

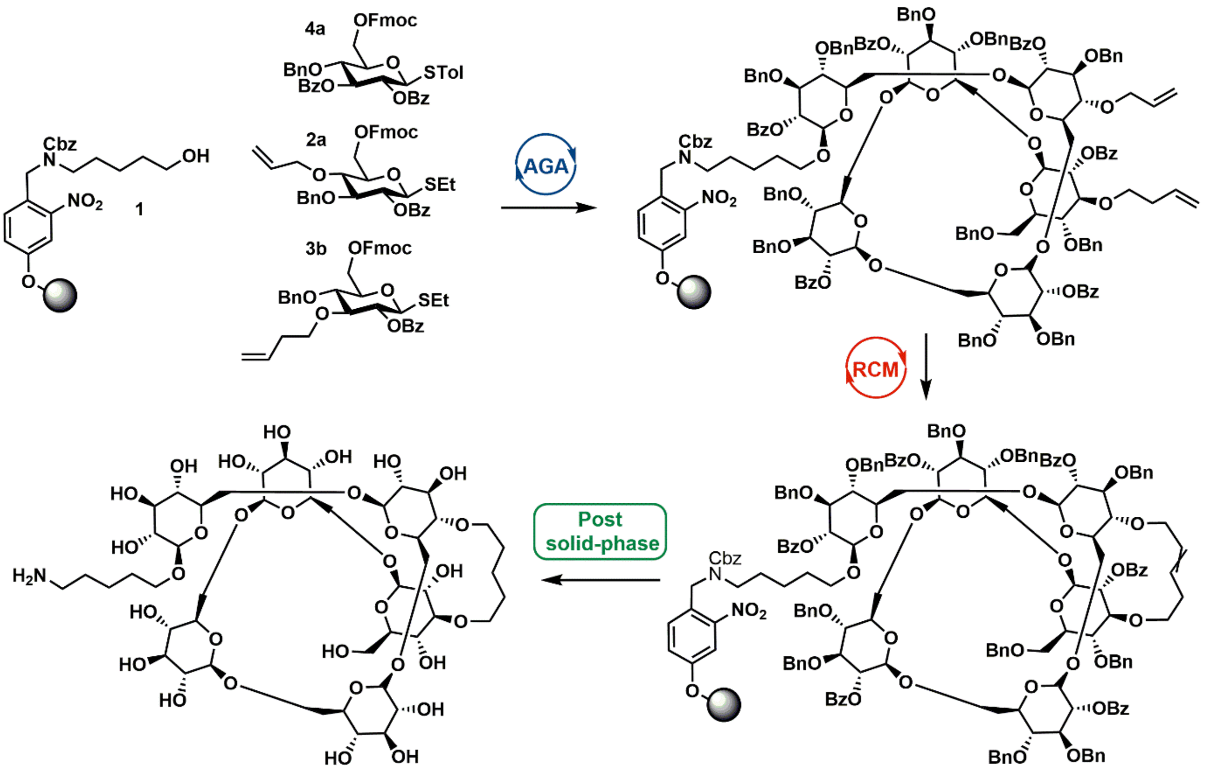

Step	Module	BB/reagent	Repeat	Notes
AGA	A	**1** (0.015 mmol)	1	Resin swelling
	B, C1, D, E	**4a** (0.09 mmol)	1	C1: NIS (0.015 mmol)
	B, C1, D, E	**2a** (0.09 mmol)	1	C1: NIS (0.015 mmol)
	B, C1, D, E	**4a** (0.09 mmol)	3	C1: NIS (0.015 mmol)
	B, C1, D, E	**3b** (0.09 mmol)	1	C1: NIS (0.015 mmol)
RCM	F2	Grubbs 2nd gen.	3	Method B (MW 60°C)
Post solid-phase	G	-	2	
	H, I	-	-	
	J	-	-	Methods 1 and 3

After automated glycan assembly, ring-closing metathesis, photo-cleavage, global deprotection, purification, and lyophilisation was obtained **8b** as a white solid (1.7 mg, 10%).

R_t (Method 1) = 20.3 min.

HR-MS m/z = 1144.5081 $[M+H]^+$, calcd for $C_{46}H_{82}NO_{31}$: 1144.4871.

^{1}H NMR (600 MHz, D_2O), δ = 4.70 (s, 6H), 4.20 – 4.07 (m, 4H), 4.04 (dd, J = 11.9, 2.4 Hz, 1H), 3.88 – 3.81 (m, 4H), 3.78 – 3.67 (m, 6H), 3.65 – 3.56 (m, 4H), 3.55 – 3.43 (m, 5H), 3.43

– 3.31 (m, 10H), 3.30 – 3.19 (m, 7H), 3.16 (t, *J* = 7.9 Hz, 1H), 2.91 (d, *J* = 7.5 Hz, 2H), 1.63 – 1.54 (m, 5H), 1.54 – 1.43 (m, 2H), 1.42 – 1.31 (m, 3H).

^{13}C NMR (151 MHz, D_2O), δ = 103.40, 103.02, 102.92, 102.81, 102.29, 102.18, 83.56, 83.56, 77.45, 75.76, 75.66, 75.57, 75.53, 75.43, 74.86, 74.75, 74.15, 73.81, 73.21, 73.03, 73.00, 72.98, 72.66, 71.90, 70.15, 69.62, 69.44, 69.37, 69.16, 69.04, 68.97, 68.72, 68.67, 68.36, 67.61, 60.66, 60.62, 39.32, 29.03, 28.87, 28.14, 27.36, 26.38, 25.47, 22.51, 22.05.

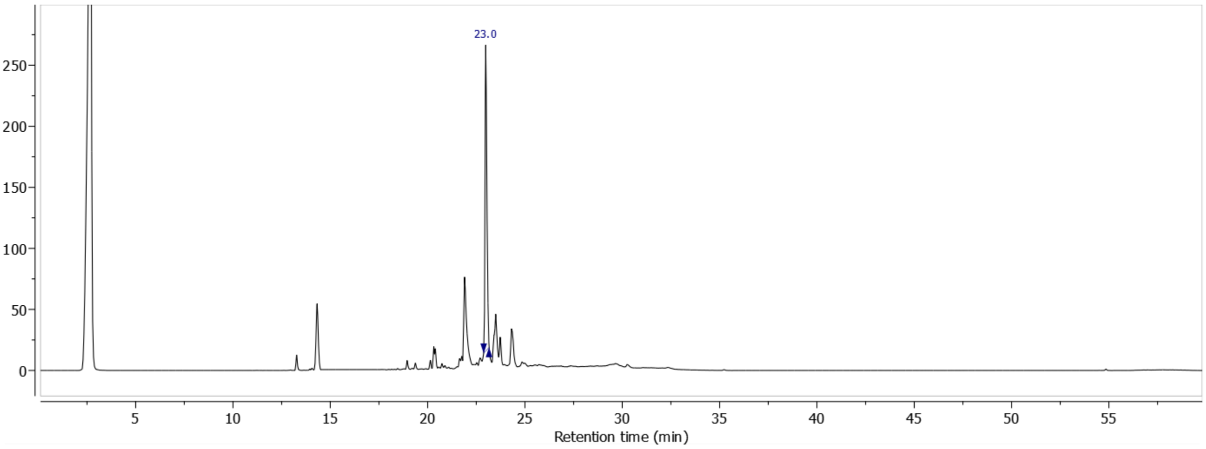

Figure S75. RP-HPLC trace of crude **8b**.

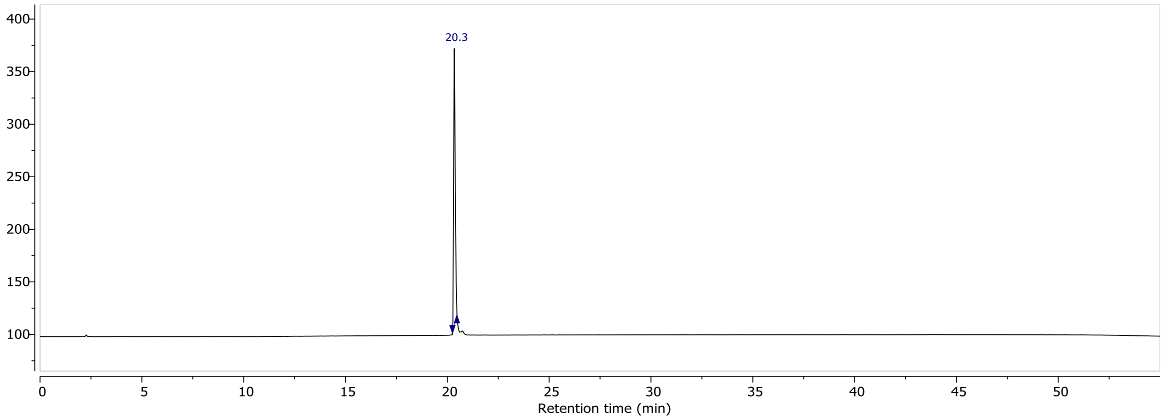

Figure S76. RP-HPLC trace of pure **8b**.

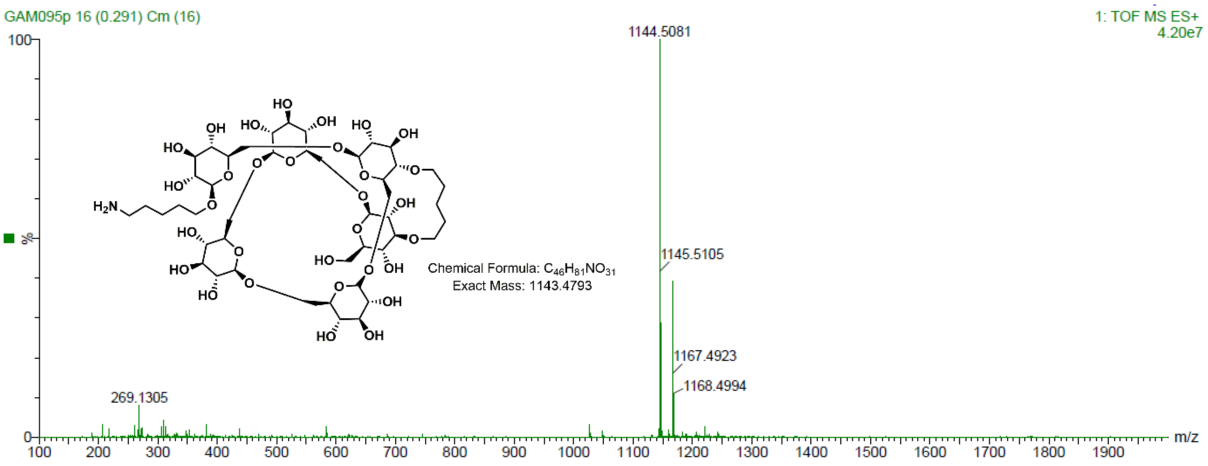

Figure S77. HR-MS of **8b**.

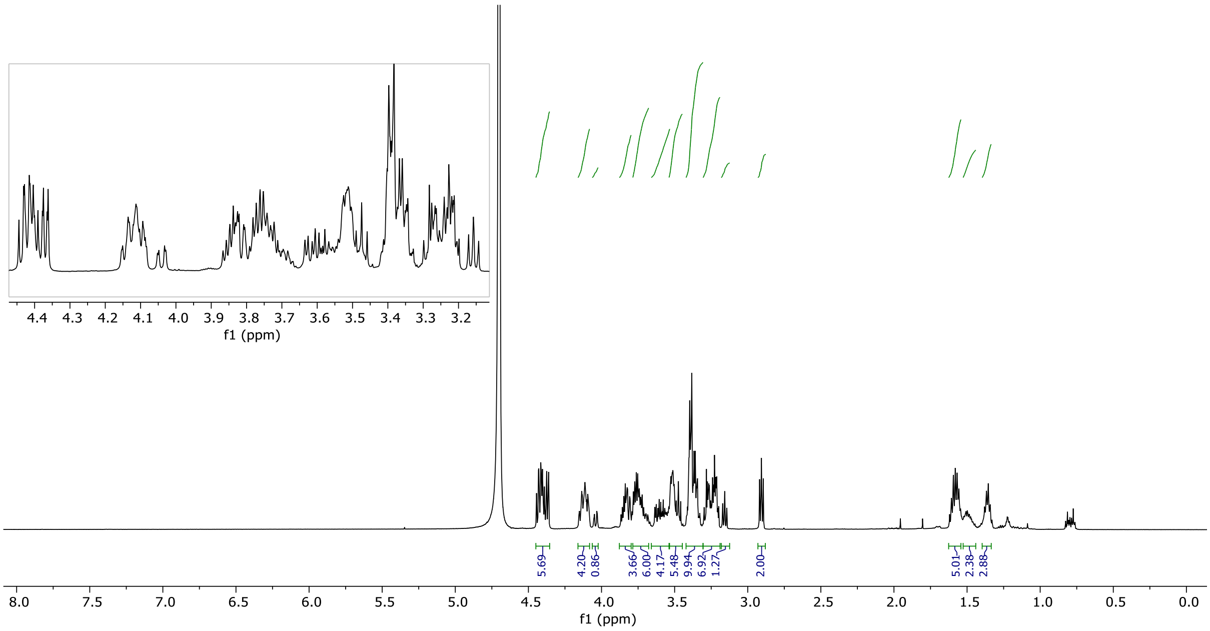

Figure S78. ^{1}H NMR (700 MHz, D_2O) spectrum of **8b**.

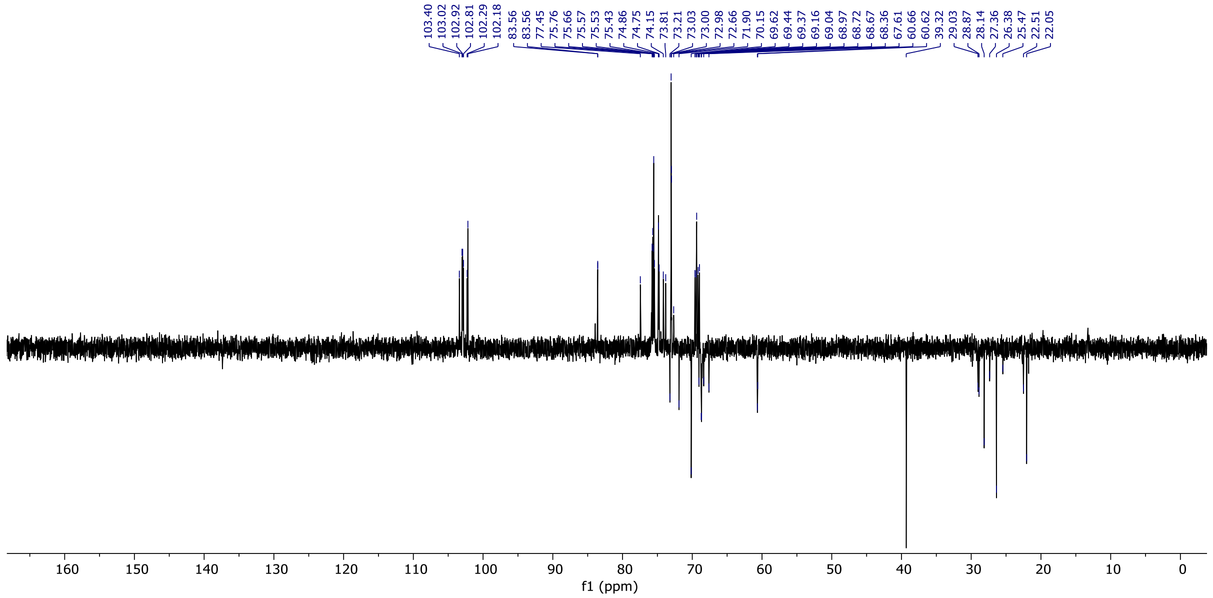

Figure S79. ^{13}C- APT NMR (700 MHz, D_2O) spectrum of **8b**.

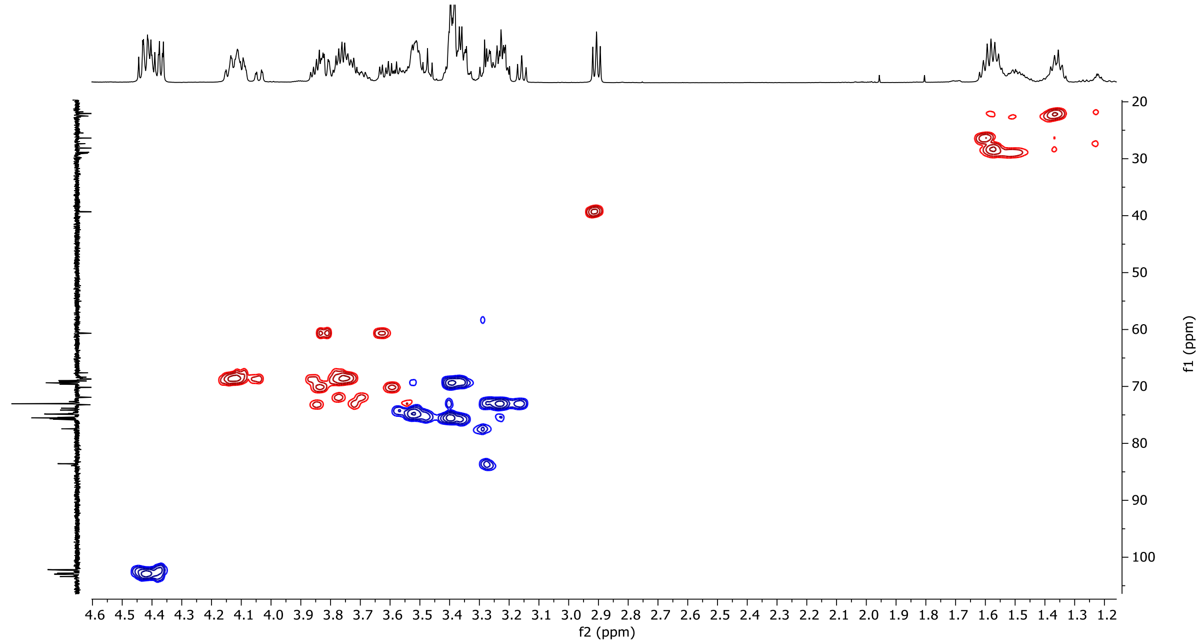

Figure S80. HSQC (700 MHz, D_2O) spectrum of **8b**.

Synthesis and analytical data of 8c

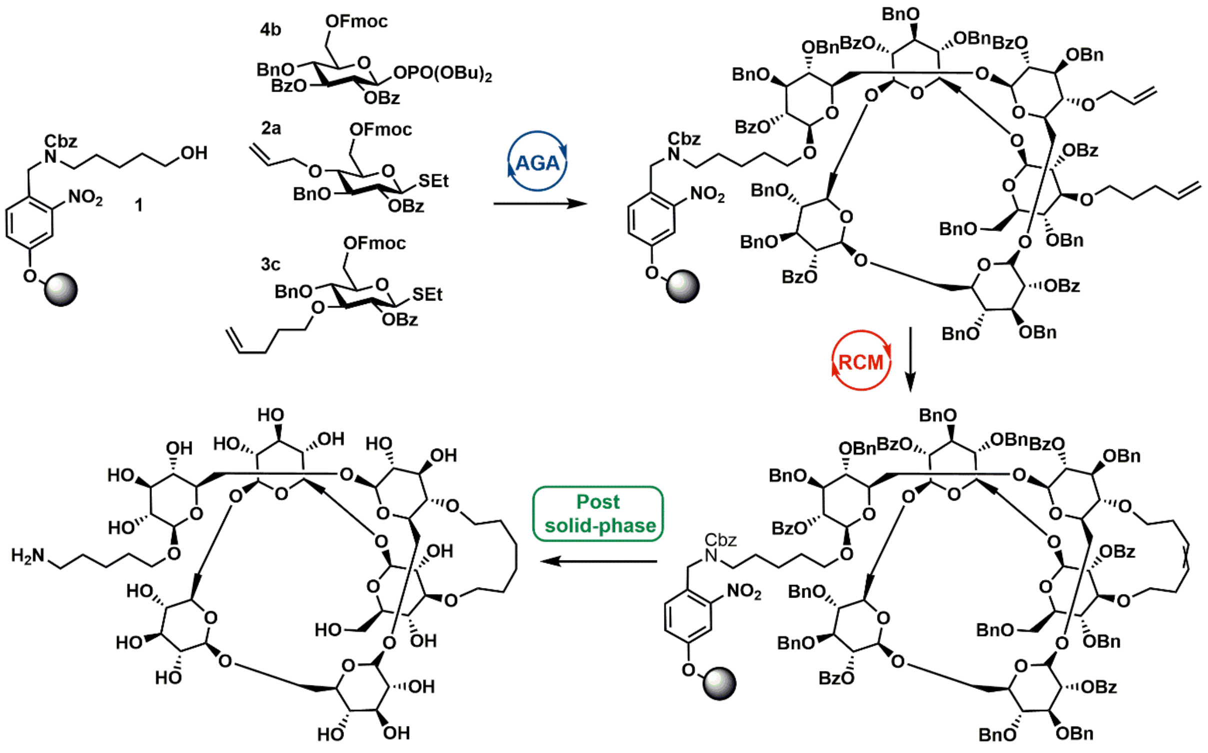

Step	Module	BB/reagent	Repeat	Notes
AGA	A	**1** (0.015 mmol)	1	Resin swelling
	B, C3, D, E	**4b** (0.06 mmol)	1	C3: TMSOTf (0.06 mmol)
	B, C1, D, E	**2a** (0.09 mmol)	1	C1: NIS (0.15 mmol)
	B, C3, D, E	**4b** (0.06 mmol)	3	C3: TMSOTf (0.06 mmol)
	B, C2, D, E	**3c** (0.09 mmol)	1	C2: NIS (0.09 mmol)
RCM	F2	Grubbs 2nd gen.	3	Method B (MW 60°C)
Post solid-phase	G	-	2	
	H, I	-	-	
	J	-	-	Methods 1 and 3

After automated glycan assembly, ring-closing metathesis, photo-cleavage, global deprotection, purification, and lyophilisation was obtained **8c** as a white solid (2.6 mg, 15%).

R_t (Method 1) = 21.1 min.

HR-MS m/z = 1158.5058 $[M+H]^+$, calcd for $C_{47}H_{84}NO_{31}$: 1158.5027.

^{1}H NMR (600 MHz, D_2O), δ = 4.45 – 4.39 (m, 4H), 4.37 (d, J = 8.3 Hz, 2H), 4.14 – 4.06 (m, 5H), 3.87 – 3.79 (m, 3H), 3.79 – 3.70 (m, 6H), 3.70 – 3.65 (m, 1H), 3.65 – 3.55 (m, 3H), 3.55

– 3.48 (m, 4H), 3.48 – 3.42 (m, 3H), 3.42 – 3.31 (m, 8H), 3.31 – 3.25 (m, 3H), 3.26 – 3.18 (m, 4H), 3.16 (d, *J* = 8.4 Hz, 1H), 2.91 (d, *J* = 7.5 Hz, 1H), 1.63 – 1.54 (m, 4H), 1.55 – 1.47 (m, 4H), 1.35 (p, *J* = 7.8 Hz, 2H), 1.32 – 1.25 (m, 4H).

^{13}C NMR (151 MHz, D_2O) δ 103.35, 103.13, 103.02, 102.81, 102.30, 102.18, 83.56, 77.65, 75.85, 75.66, 75.54, 75.51, 75.43, 74.89, 74.85, 74.23, 73.86, 73.05, 73.03, 72.97, 72.48, 71.59, 70.15, 69.36, 69.28, 68.76, 68.70, 68.66, 68.36, 68.10, 60.62, 39.31, 29.18, 28.88, 28.14, 26.38, 24.85, 22.05.

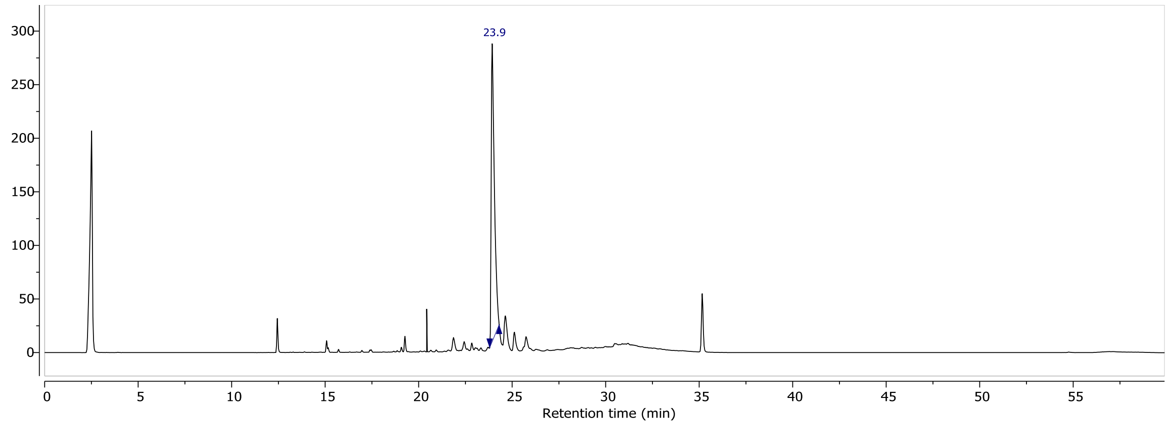

Figure S81. RP-HPLC trace of crude **8c**.

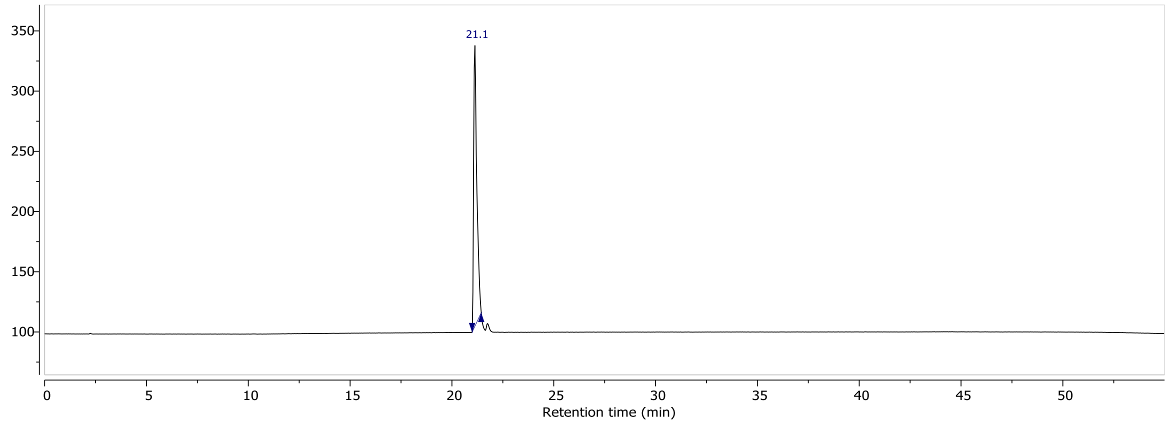

Figure S82. RP-HPLC trace of pure **8c**.

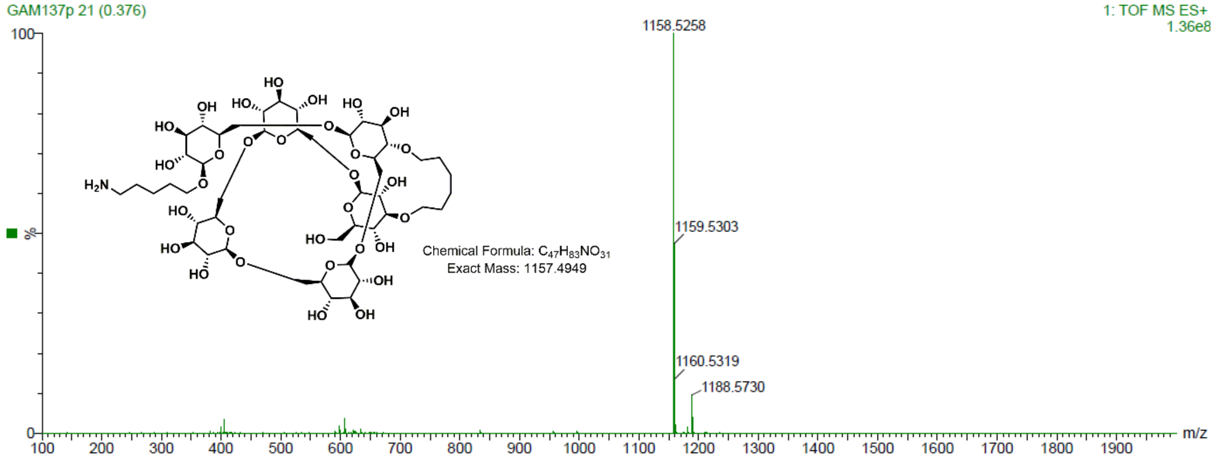

Figure S83. HR-MS of **8c**.

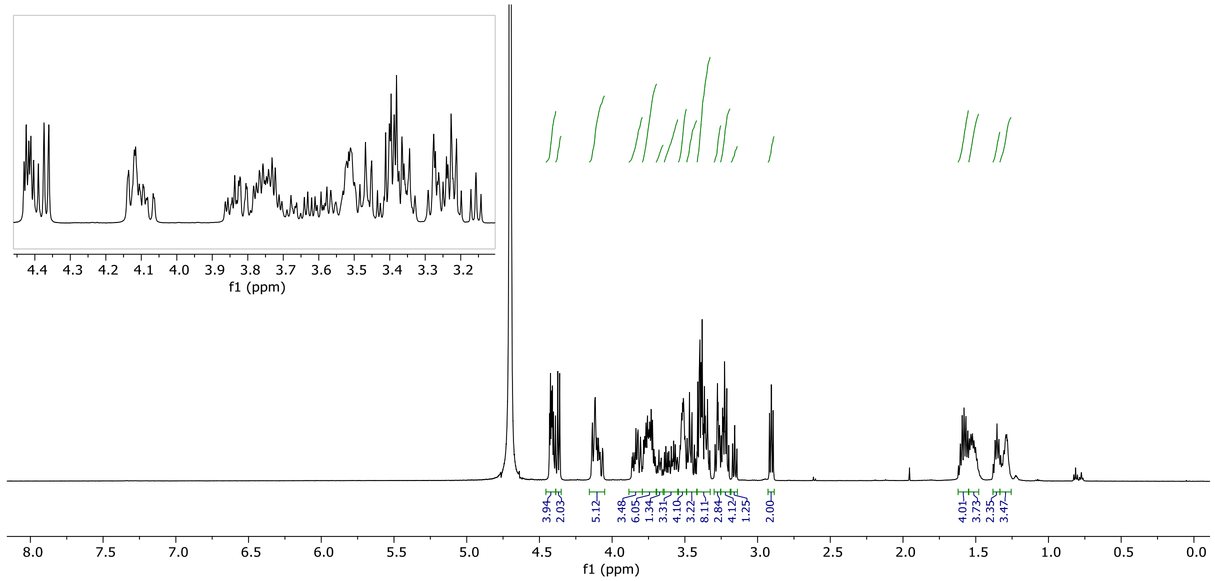

Figure S84. ^{1}H NMR (700 MHz, D_2O) spectrum of **8c**.

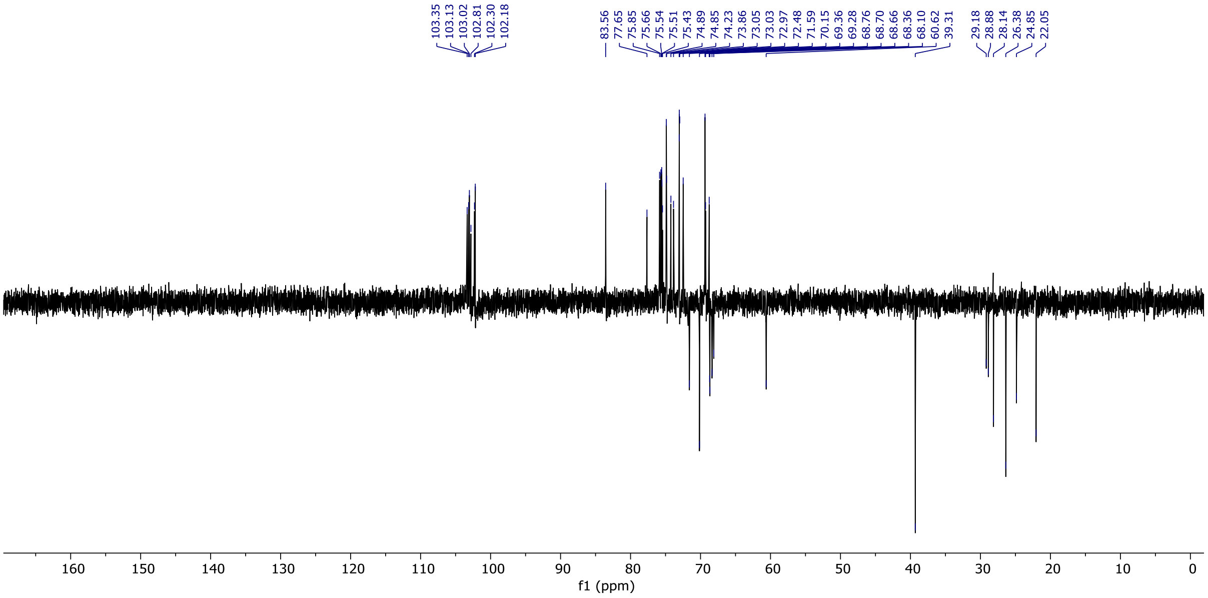

Figure S85. ^{13}C-APT NMR (700 MHz, D_2O) spectrum of **8c**.

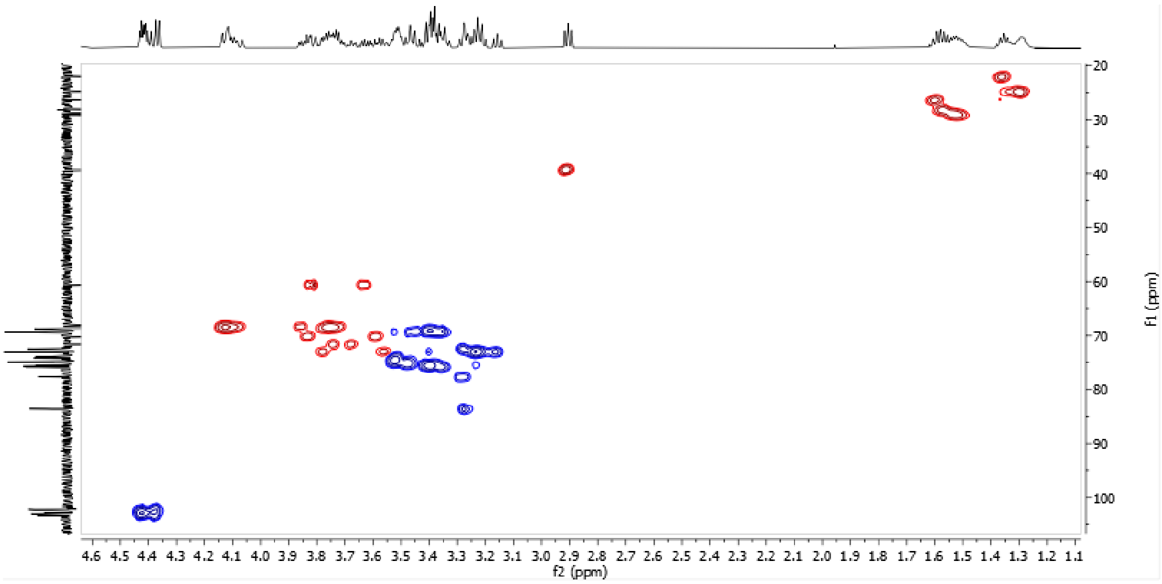

Figure S86. HSQC (700 MHz, D_2O) spectrum of **8c**.

Synthesis and analytical data of 8d

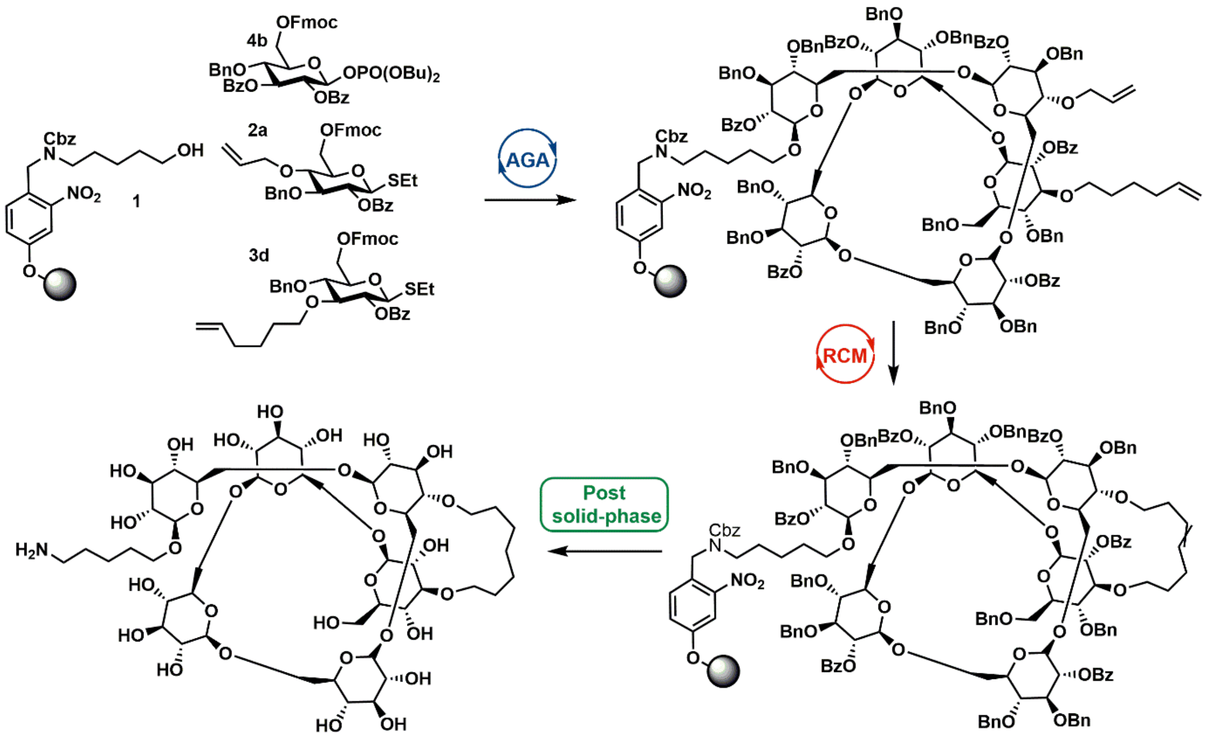

Step	Module	BB/reagent	Repeat	Notes
**AGA**	A	**1** (0.015 mmol)	1	Resin swelling
	B, C3, D, E	**4b** (0.06 mmol)	1	C3: TMSOTf (0.06 mmol)
	B, C1, D, E	**2a** (0.09 mmol)	1	C1: NIS (0.15 mmol)
	B, C3, D, E	**4b** (0.06 mmol)	3	C3: TMSOTf (0.06 mmol)
	B, C2, D, E	**3d** (0.09 mmol)	1	C2: NIS (0.09 mmol)
**RCM**	F2	Grubbs 2nd gen.	3	Method B (MW 60°C)
**Post solid-phase**	G	-	2	
	H, I	-	-	
	J	-	-	Methods 1 and 3

After automated glycan assembly, ring-closing metathesis, photo-cleavage, global deprotection, purification, and lyophilisation was obtained **8d** as a white solid (2.3 mg, 13%).

R_t (Method 1) = 27.3 min.

HR-MS m/z = 1172.5410 $[M+H]^+$, calcd for $C_{48}H_{86}NO_{31}$: 1172.5184.

^{1}H NMR (700 MHz, D_2O) δ 4.47 – 4.41 (m, 5H), 4.39 (d, J = 8.0 Hz, 2H), 4.18 – 4.08 (m, 7H), 3.90 – 3.81 (m, 4H), 3.80 – 3.74 (m, 7H), 3.73 – 3.67 (m, 1H), 3.67 – 3.59 (m, 4H), 3.58 – 3.49

(m, 10H), 3.45 – 3.36 (m, 14H), 3.31 – 3.22 (m, 8H), 3.18 (t, *J* = 8.6 Hz, 1H), 2.93 (t, *J* = 7.6 Hz, 2H), 1.66 – 1.56 (m, 5H), 1.54 (q, *J* = 6.7 Hz, 4H), 1.40 – 1.36 (m, 2H), 1.36 – 1.31 (m, 2H), 1.30 – 1.26 (m, 3H).

^{13}C NMR (176 MHz, D_2O), δ = 103.35, 103.21, 103.17, 103.03, 102.87, 102.59, 102.24, 83.71, 77.66, 75.92, 75.73, 75.62, 75.60, 75.55, 75.47, 75.07, 74.96, 74.88, 74.49, 73.92, 73.21, 73.10, 73.08, 73.02, 72.95, 72.57, 72.26, 70.19, 69.55, 69.43, 69.20, 69.02, 68.72, 68.68, 68.43, 60.71, 39.38, 29.22, 28.95, 28.48, 28.19, 27.94, 26.42, 25.29, 24.93, 22.11.

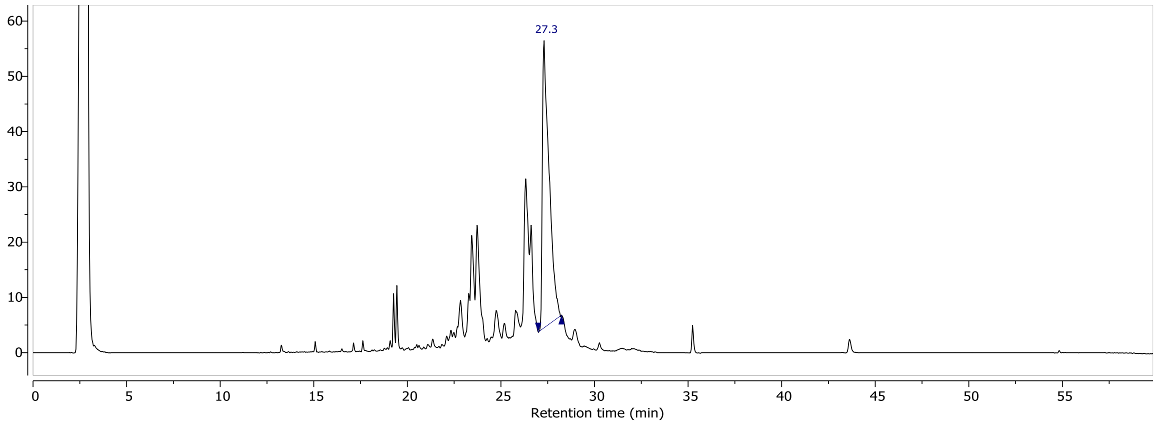

Figure S87. RP-HPLC trace of crude **8d**.

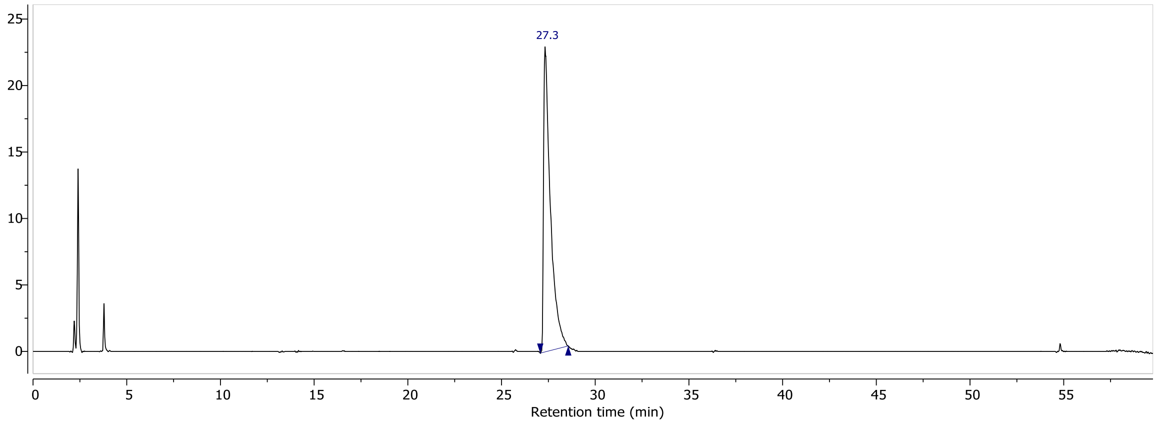

Figure S88. RP-HPLC trace of pure **8d**.

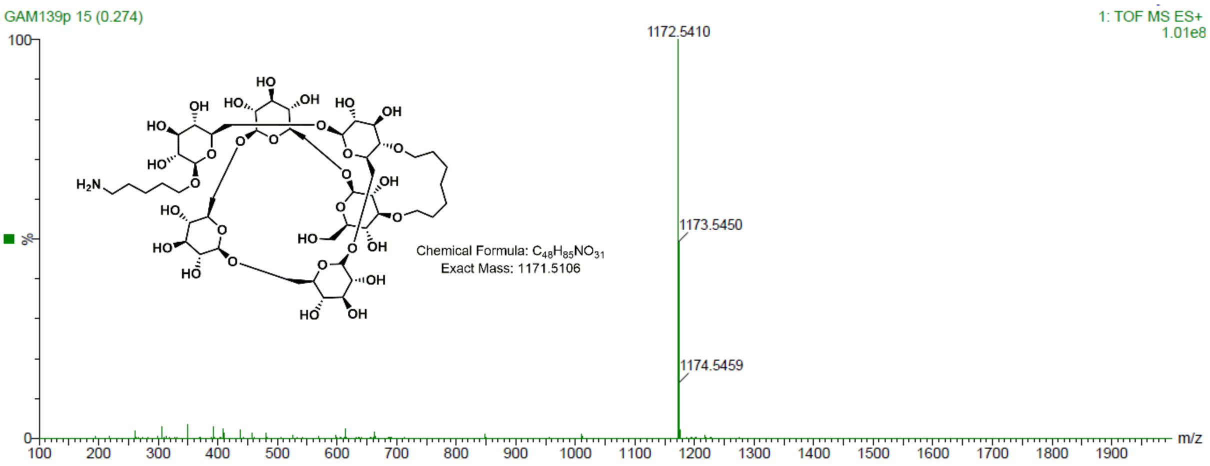

Figure S89. HR-MS of **8d**.

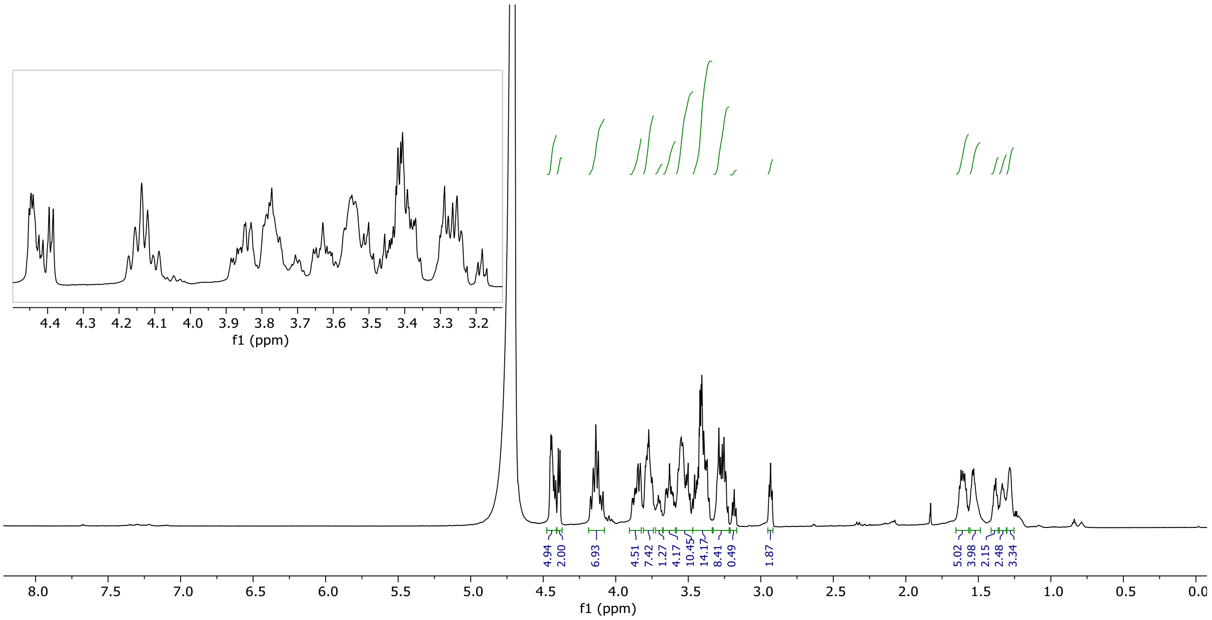

Figure S90. ^{1}H NMR (700 MHz, D_2O) spectrum of **8d**.

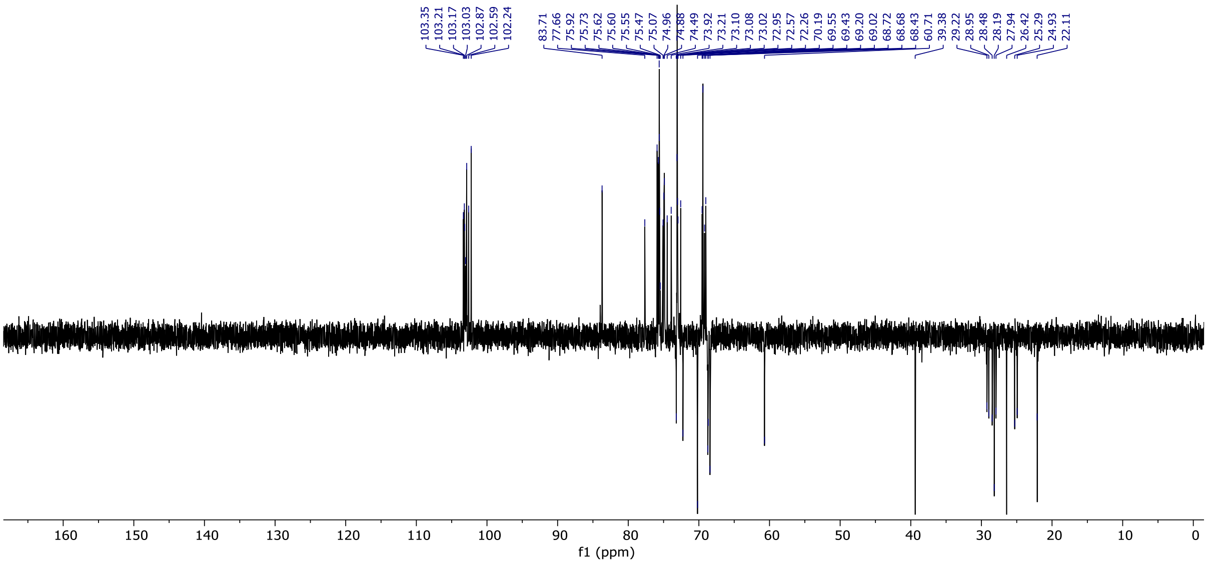

Figure S91. ^{13}C-APT NMR (700 MHz, D_2O) spectrum of **8d**.

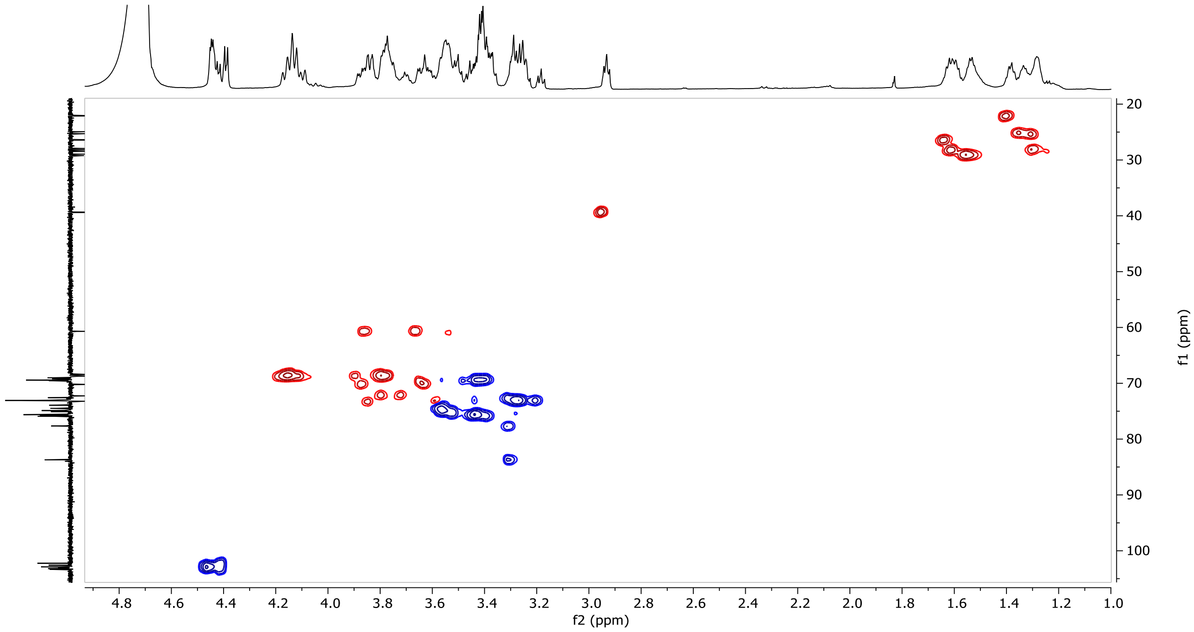

Figure S92. HSQC (700 MHz, D_2O) spectrum of **8d**.

Synthesis and analytical data of 8e

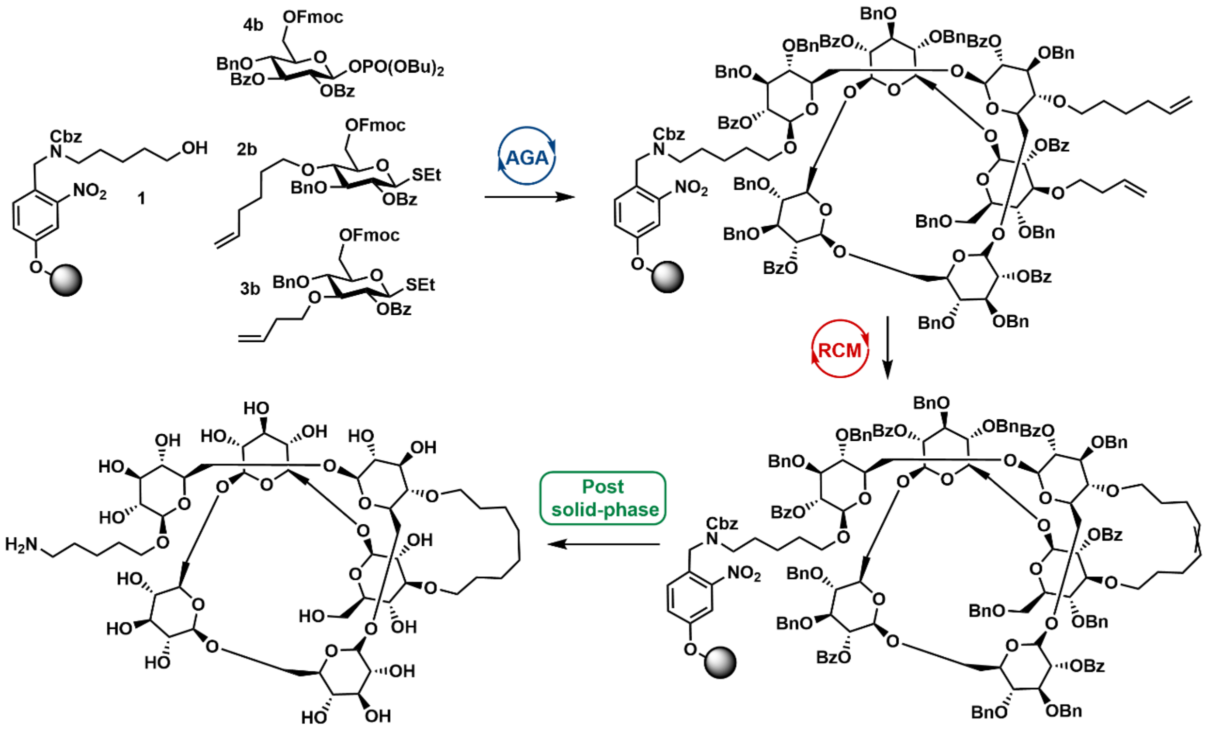

Step	Module	BB/reagent	Repeat	Notes
AGA	A	**1** (0.015 mmol)	1	Resin swelling
	B, C3, D, E	**4b** (0.06 mmol)	1	C3: TMSOTf (0.06 mmol)
	B, C2, D, E	**2b** (0.09 mmol)	1	C2: NIS (0.09 mmol)
	B, C3, D, E	**4b** (0.06 mmol)	3	C3: TMSOTf (0.06 mmol)
	B, C2, D, E	**3b** (0.09 mmol)	1	C2: NIS (0.09 mmol)
RCM	F2	Grubbs 1st gen.	3	Method A (25°C)
Post solid-phase	G	-	2	
	H, I	-	-	
	J	-	-	Methods 2 and 4

After automated glycan assembly, ring-closing metathesis, photo-cleavage, global deprotection, purification, and lyophilisation was obtained **8e** as a white solid (2.7 mg, 15%).

R_t (Method 2) = 11.6 min.

HR-MS m/z = 1186.5576 $[M+H]^+$, calcd for $C_{49}H_{88}NO_{31}$: 1186.5340.

^1H NMR (600 MHz, D_2O) δ 4.44 – 4.35 (m, 6H), 4.16 – 4.07 (m, 5H), 3.86 – 3.79 (m, 3H), 3.80 – 3.69 (m, 6H), 3.65 – 3.56 (m, 4H), 3.55 – 3.48 (m, 4H), 3.47 – 3.42 (m, 2H), 3.42 – 3.33

(m, 9H), 3.31 – 3.18 (m, 7H), 3.16 (t, J = 8.4 Hz, 1H), 2.91 (t, J = 7.5 Hz, 2H), 1.58 (dp, J = 14.4, 7.2 Hz, 4H), 1.53 – 1.46 (m, 4H), 1.35 (p, J = 7.8 Hz, 2H), 1.32 – 1.17 (m, 6H).

^{13}C NMR (176 MHz, D_2O) δ 103.27, 103.12, 102.82, 102.79, 102.48, 102.14, 83.59, 77.45, 75.82, 75.61, 75.50, 75.44, 75.35, 74.85, 74.79, 74.26, 73.77, 73.31, 73.17, 72.99, 72.93, 72.39, 71.96, 70.10, 69.52, 69.29, 69.24, 69.08, 68.75, 68.59, 68.51, 68.37, 68.18, 60.58, 39.27, 29.17, 28.99, 28.10, 28.05, 26.34, 25.06, 24.85, 22.03.

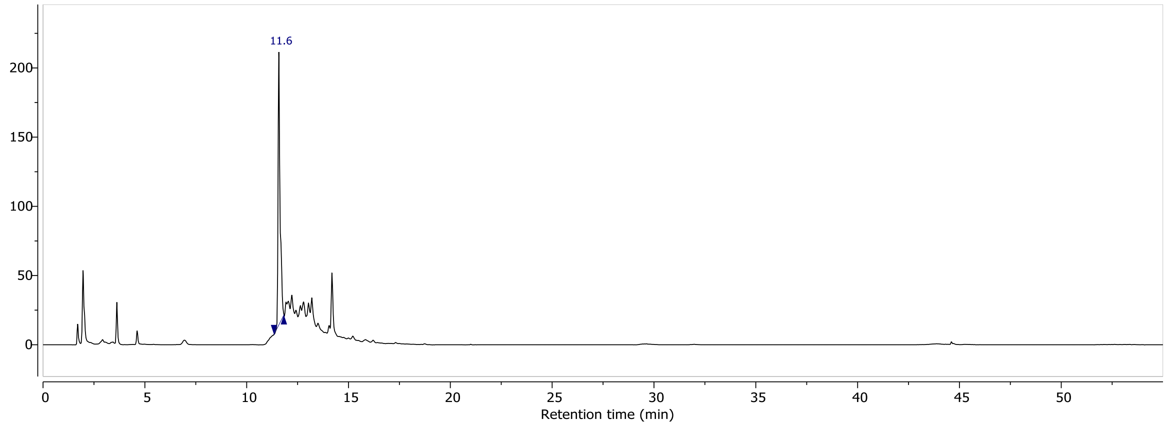

Figure S93. RP-HPLC trace of crude **8e**.

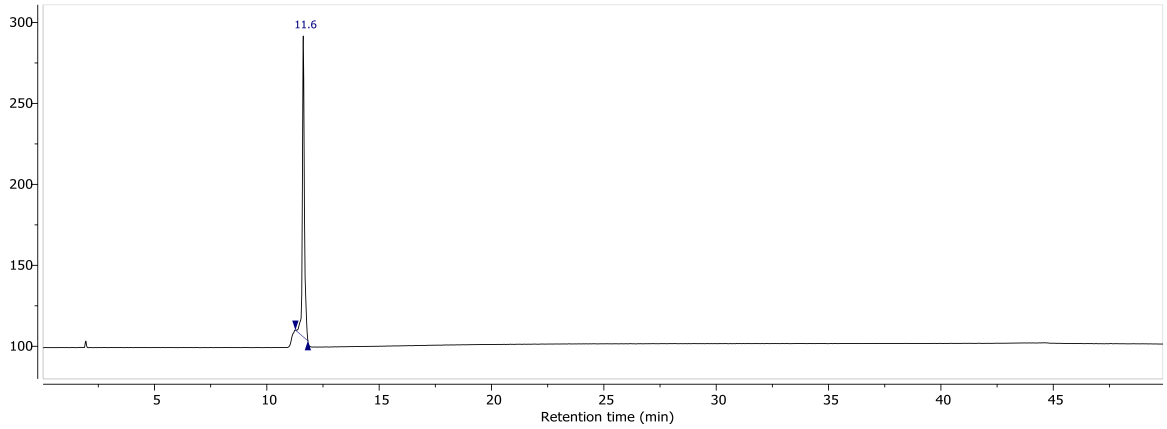

Figure S94. RP-HPLC trace of pure **8e**.

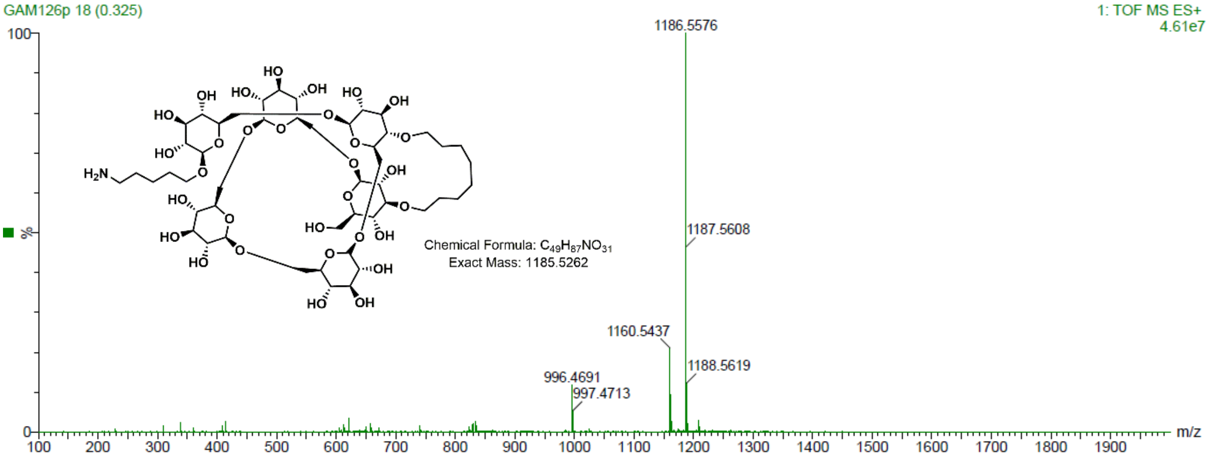

Figure S95. HR-MS of **8e**.

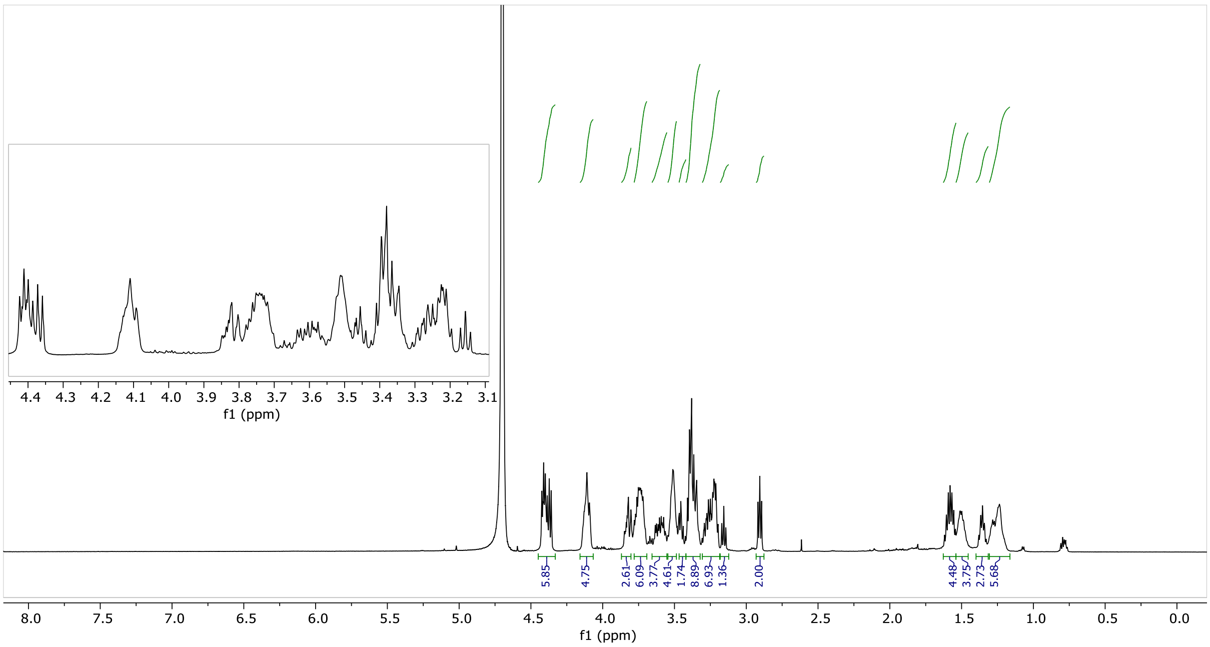

Figure S96. ^{1}H NMR (700 MHz, D_2O) spectrum of **8e**.

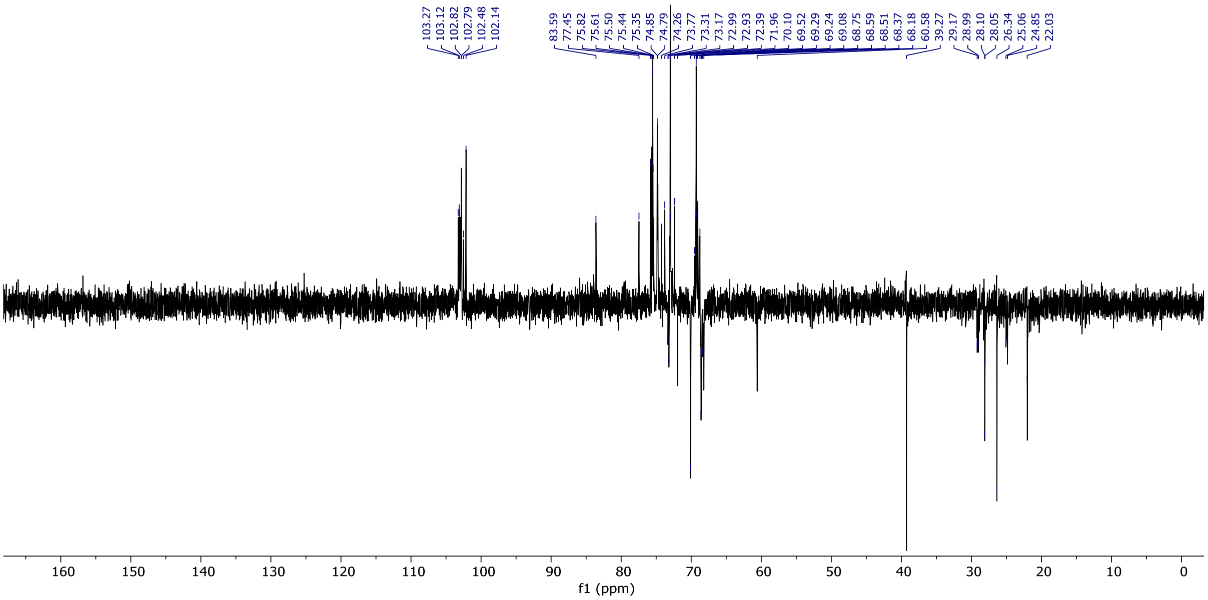

Figure S97. ^{13}C NMR (700 MHz, D_2O) spectrum of **8e**.

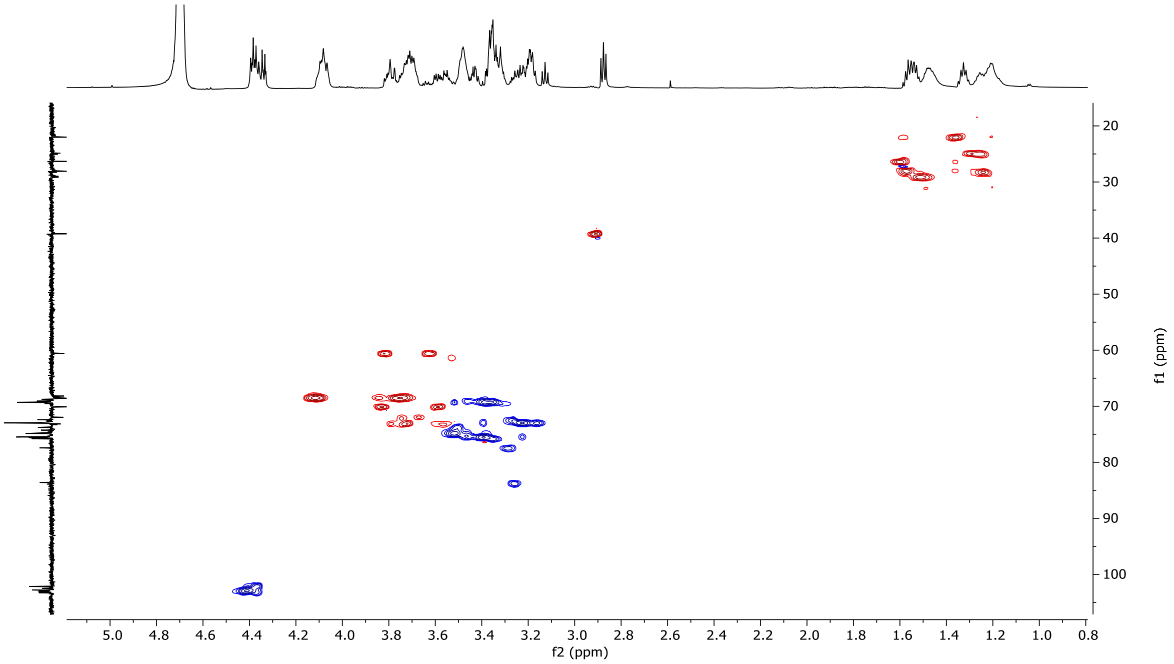

Figure S98. HSQC (700 MHz, D_2O) spectrum of **8e**.

Synthesis and analytical data of 8f

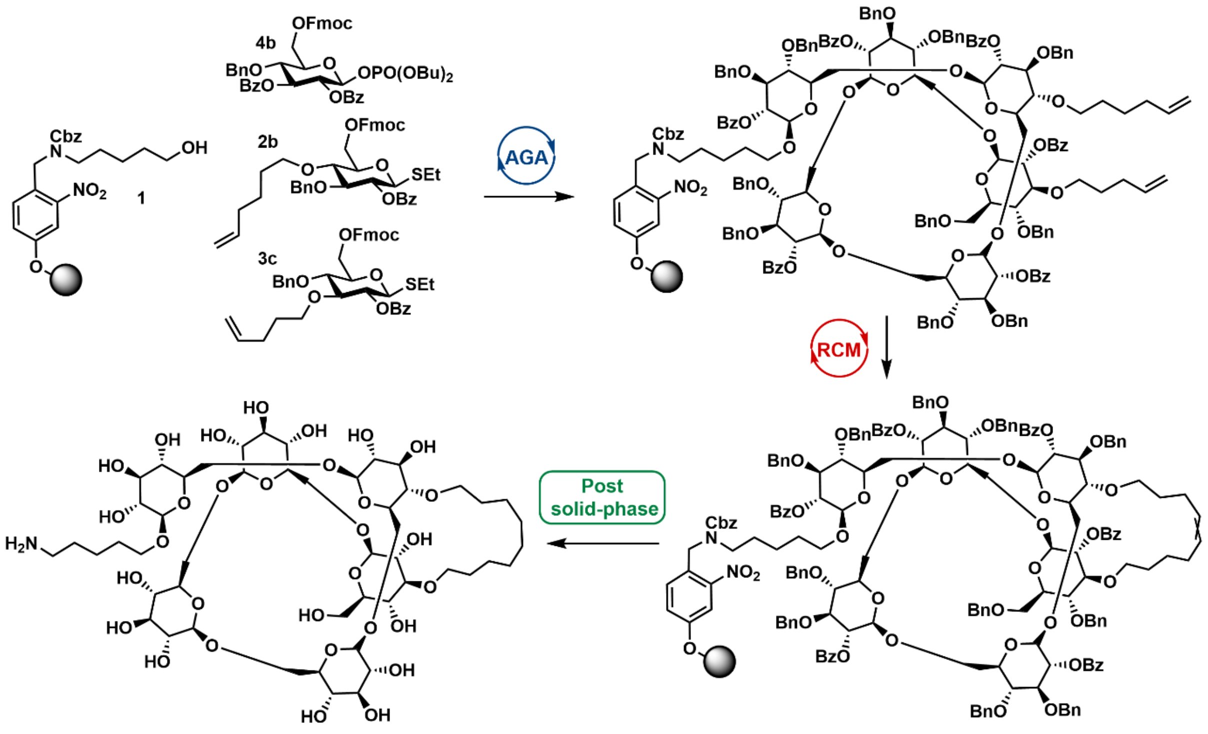

Step	Module	BB/reagent	Repeat	Notes
AGA	A	**1** (0.015 mmol)	1	Resin swelling
	B, C3, D, E	**4b** (0.06 mmol)	1	C3: TMSOTf (0.06 mmol)
	B, C2, D, E	**2b** (0.09 mmol)	1	C2: NIS (0.09 mmol)
	B, C3, D, E	**4b** (0.06 mmol)	3	C3: TMSOTf (0.06 mmol)
	B, C2, D, E	**3c** (0.09 mmol)	1	C2: NIS (0.09 mmol)
RCM	F2	Grubbs 1st gen.	3	Method A (25°C)
Post solid-phase	G	-	2	
	H, I	-	-	
	J	-	-	Methods 2 and 4

After automated glycan assembly, ring-closing metathesis, photo-cleavage, global deprotection, purification, and lyophilisation was obtained **8f** as a white solid (2.5 mg, 14%).

R_t (Method 2) = 12.6 min.

HR-MS *m/z* = 1200.5734 $[M+H]^+$, calcd for $C_{50}H_{90}NO_{31}$: 1200.5497.

^{1}H NMR (700 MHz, D_2O) δ 4.39 – 4.34 (m, 4H), 4.32 (d, *J* = 8.6 Hz, 2H), 4.10 – 4.04 (m, 5H), 3.80 – 3.75 (m, 3H), 3.73 – 3.64 (m, 6H), 3.62 (q, *J* = 7.4 Hz, 1H), 3.57 (dd, *J* = 12.9, 5.3 Hz, 1H), 3.55 – 3.50 (m, 2H), 3.46 (d, *J* = 6.7 Hz, 5H), 3.44 – 3.40 (m, 2H), 3.37 – 3.28 (m, 10H),

3.25 – 3.14 (m, 8H), 3.11 (t, *J* = 8.5 Hz, 1H), 2.86 (t, *J* = 7.4 Hz, 2H), 1.53 (dq, *J* = 16.2, 7.7, 7.2 Hz, 4H), 1.49 – 1.42 (m, 4H), 1.31 (p, *J* = 7.9 Hz, 2H), 1.24 (d, *J* = 6.9 Hz, 2H), 1.23 – 1.14 (m, 8H).

^{13}C NMR (176 MHz, D_2O), δ = 103.26, 103.09, 103.06, 102.92, 102.77, 102.48, 102.12, 83.86, 83.58, 77.46, 75.79, 75.58, 75.46, 75.43, 74.89, 74.80, 74.75, 74.34, 73.75, 73.19, 72.96, 72.89, 72.82, 72.45, 72.19, 70.09, 69.36, 69.25, 69.05, 68.99, 68.89, 68.56, 68.51, 68.24, 68.21, 60.54, 39.24, 29.12, 28.85, 28.37, 28.08, 27.80, 26.33, 25.21, 24.83, 22.00.

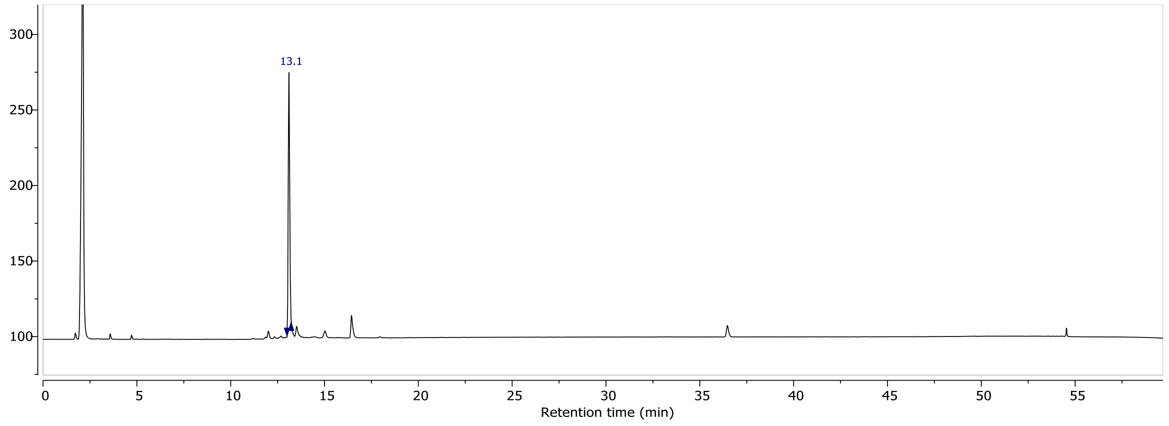

Figure S99. RP-HPLC trace of crude **8f**.

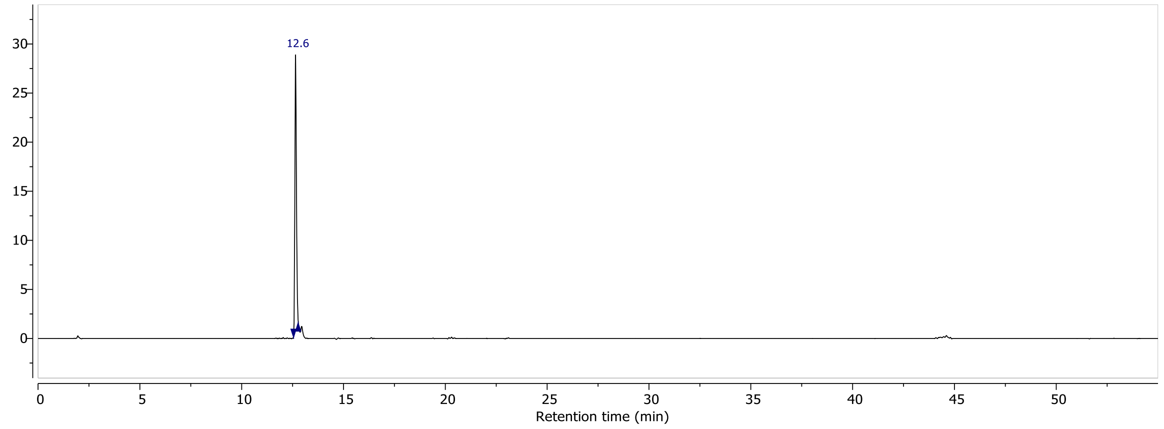

Figure S100. RP-HPLC trace of pure **8f**.

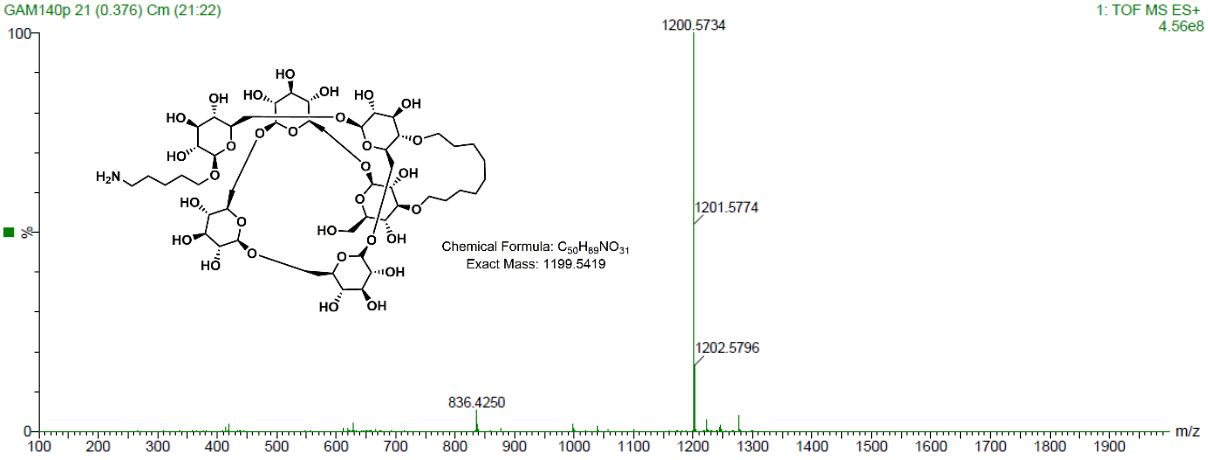

Figure S101. HR-MS of **8f**.

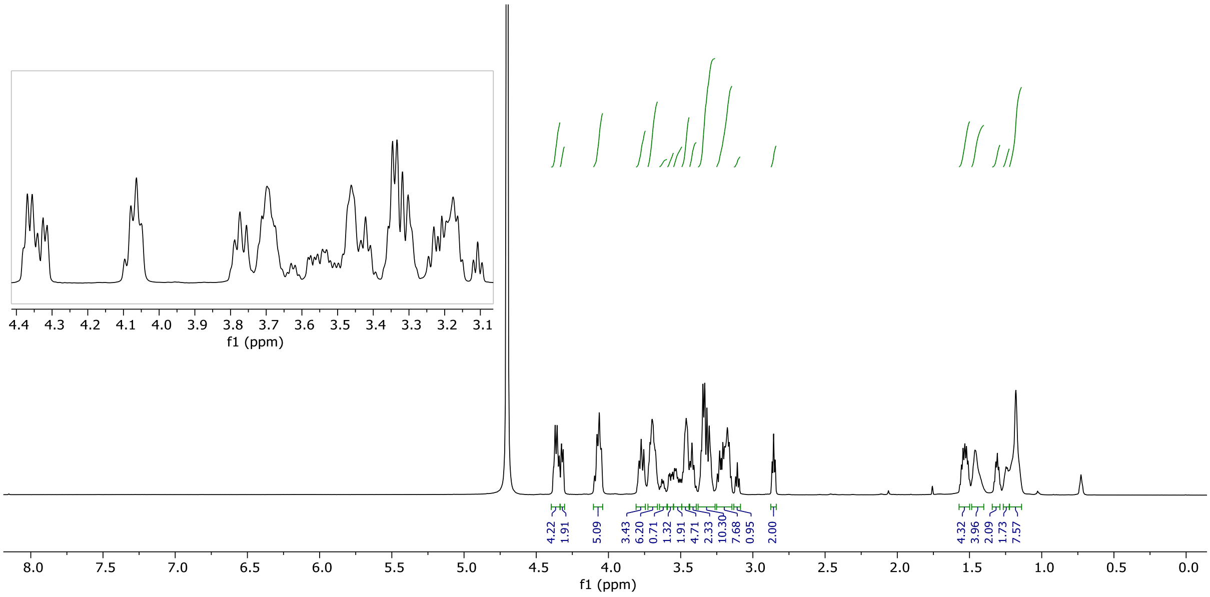

Figure S102. ^{1}H NMR (700 MHz, D_2O) spectrum of **8f**.

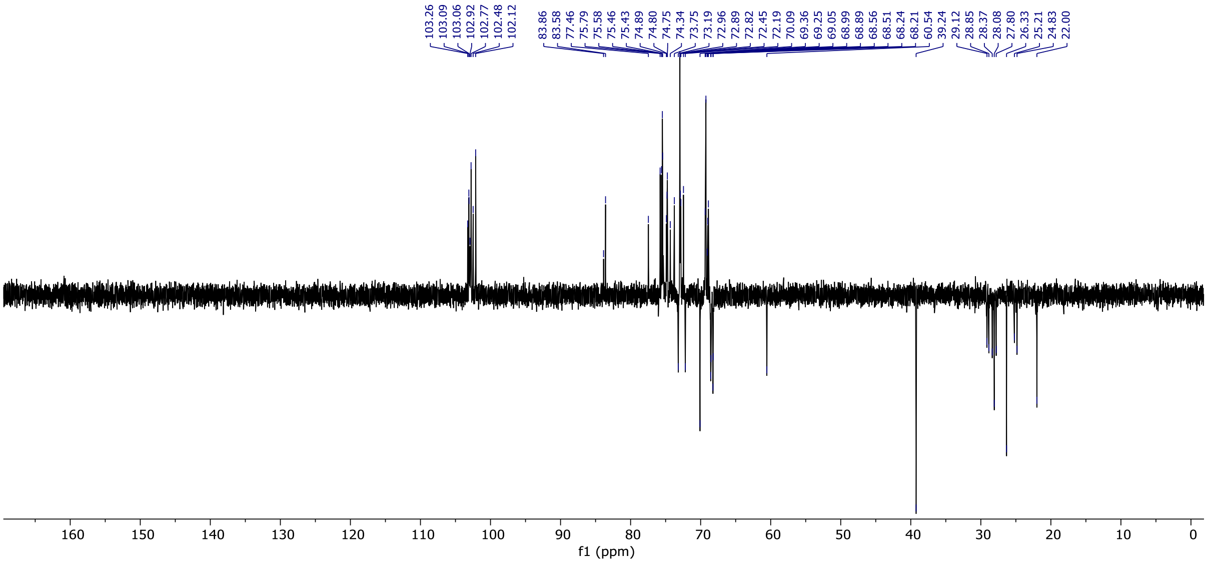

Figure S103. ^{13}C-APT NMR (700 MHz, D_2O) spectrum of **8f**.

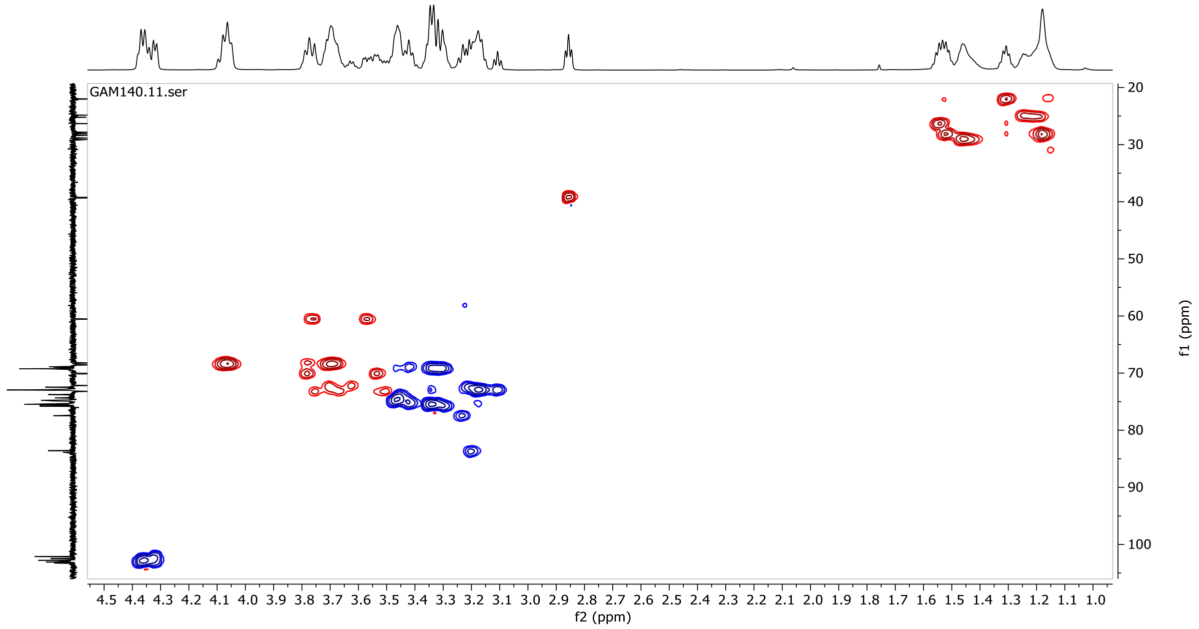

Figure S104. HSQC (700 MHz, D_2O) spectrum of **8f**.

Synthesis and analytical data of 8g

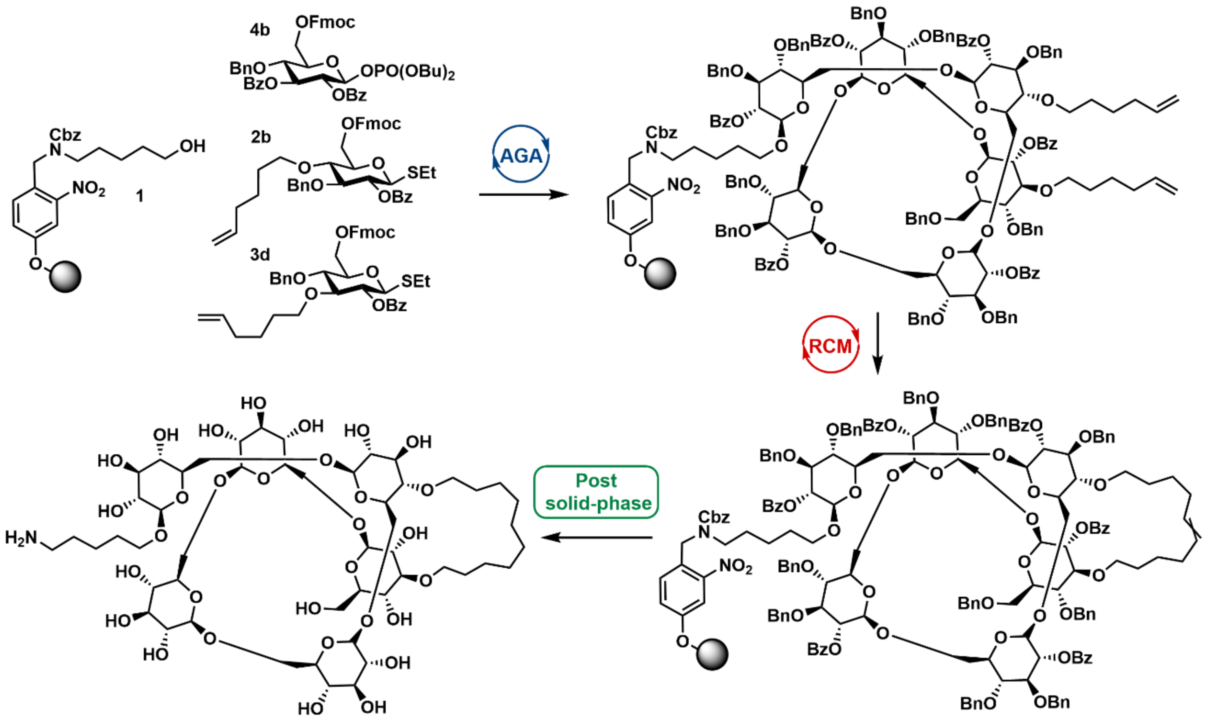

Step	Module	BB/reagent	Repeat	Notes
AGA	A	**1** (0.015 mmol)	1	Resin swelling
	B, C3, D, E	**4b** (0.06 mmol)	1	C3: TMSOTf (0.06 mmol)
	B, C2, D, E	**2b** (0.09 mmol)	1	C2: NIS (0.09 mmol)
	B, C3, D, E	**4b** (0.06 mmol)	3	C3: TMSOTf (0.06 mmol)
	B, C2, D, E	**3d** (0.09 mmol)	1	C2: NIS (0.09 mmol)
RCM	F2	Grubbs 1st gen.	3	Method A (25°C)
Post solid-phase	G	-	2	
	H, I	-	-	
	J	-	-	Methods 2 and 4

After automated glycan assembly, ring-closing metathesis, photo-cleavage, global deprotection, purification, and lyophilisation was obtained **8g** as a white solid (3.1 mg, 17%).

R_t (Method 2) = 13.6 min.

HR-MS *m/z* = 1214.4908 $[M+H]^+$, calcd for $C_{51}H_{92}NO_{31}$: 1214.5653.

^{1}H NMR (700 MHz, D_2O), δ = 4.70 (s, 4H), 4.37 (d, *J* = 8.6 Hz, 2H), 4.16 – 4.09 (m, 5H), 3.86 – 3.79 (m, 5H), 3.78 – 3.71 (m, 6H), 3.71 – 3.65 (m, 1H), 3.62 (dd, *J* = 12.4, 5.7 Hz, 1H), 3.60 – 3.55 (m, 2H), 3.54 – 3.49 (m, 5H), 3.50 – 3.45 (m, 3H), 3.43 – 3.33 (m, 11H), 3.30 – 3.19

(m, 8H), 3.16 (t, J = 8.5 Hz, 1H), 2.91 (t, J = 7.6 Hz, 2H), 1.63 – 1.54 (m, 4H), 1.54 – 1.45 (m, 4H), 1.36 (p, J = 7.8 Hz, 2H), 1.32 – 1.27 (m, 2H), 1.26 – 1.18 (m, 10H).

^{13}C NMR (176 MHz, D_2O), δ = 103.26, 103.19, 103.12, 102.83, 102.63, 102.18, 83.71, 77.49, 75.86, 75.66, 75.55, 74.97, 74.84, 74.43, 73.85, 73.20, 73.03, 72.91, 72.51, 72.36, 70.14, 69.43, 69.35, 69.27, 69.04, 68.96, 68.64, 68.45, 68.36, 68.24, 60.64, 39.31, 29.20, 28.95, 28.35, 28.14, 28.04, 26.38, 25.29, 24.89, 22.05.

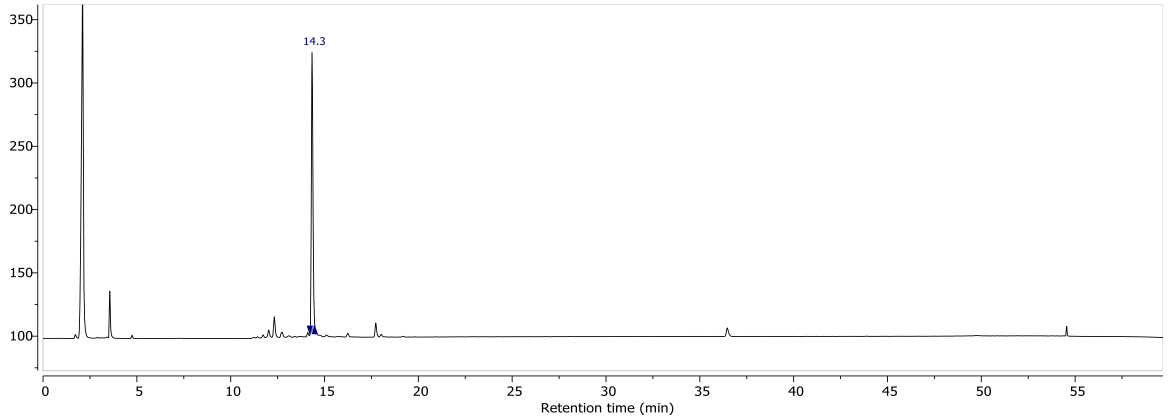

Figure S105. RP-HPLC trace of crude **8g**.

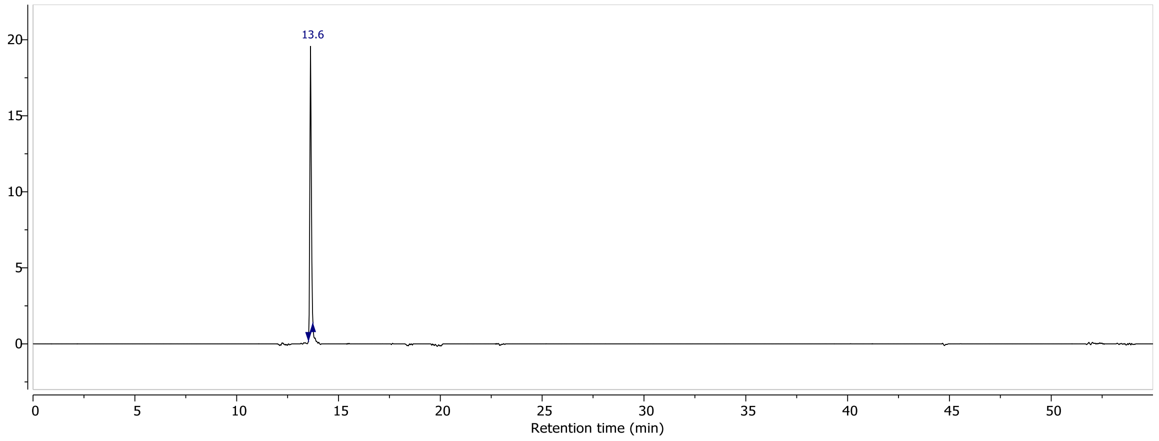

Figure S106. RP-HPLC trace of pure **8g**.

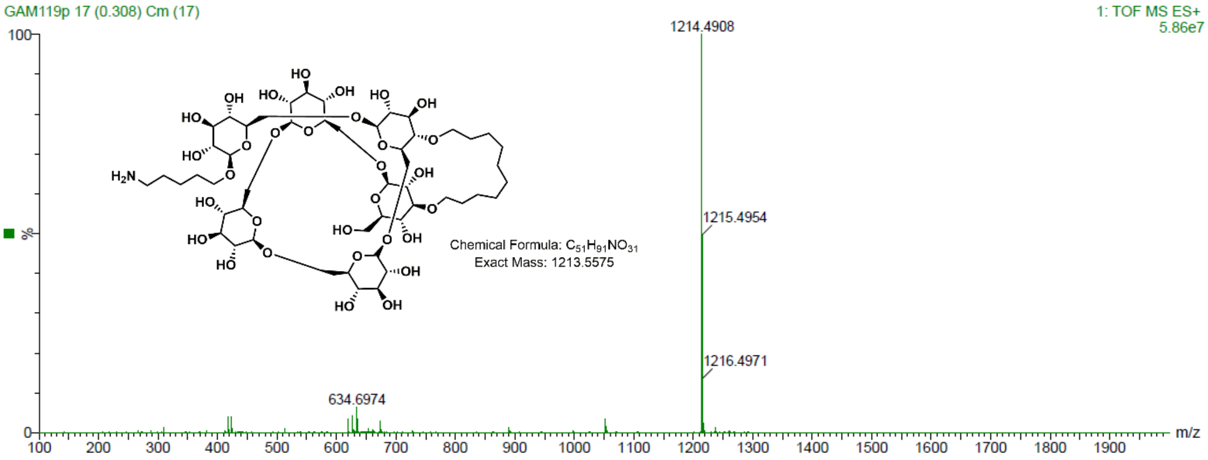

Figure S107. HR-MS of **8g**.

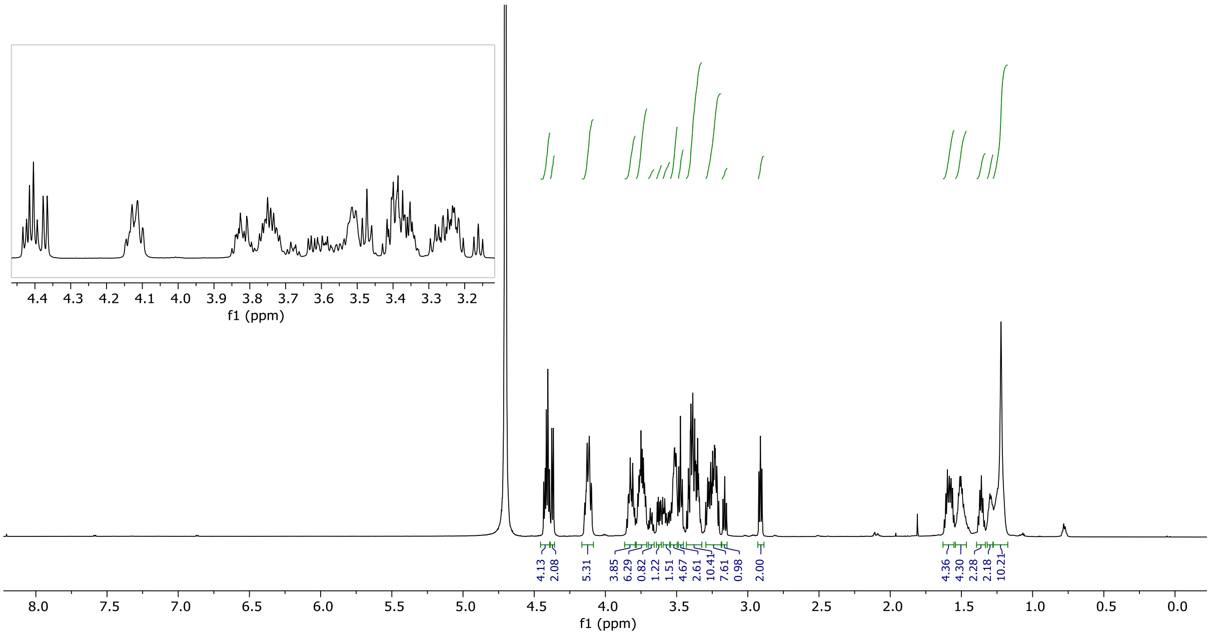

Figure S108. ^{1}H NMR (700 MHz, D_2O) spectrum of **8g**.

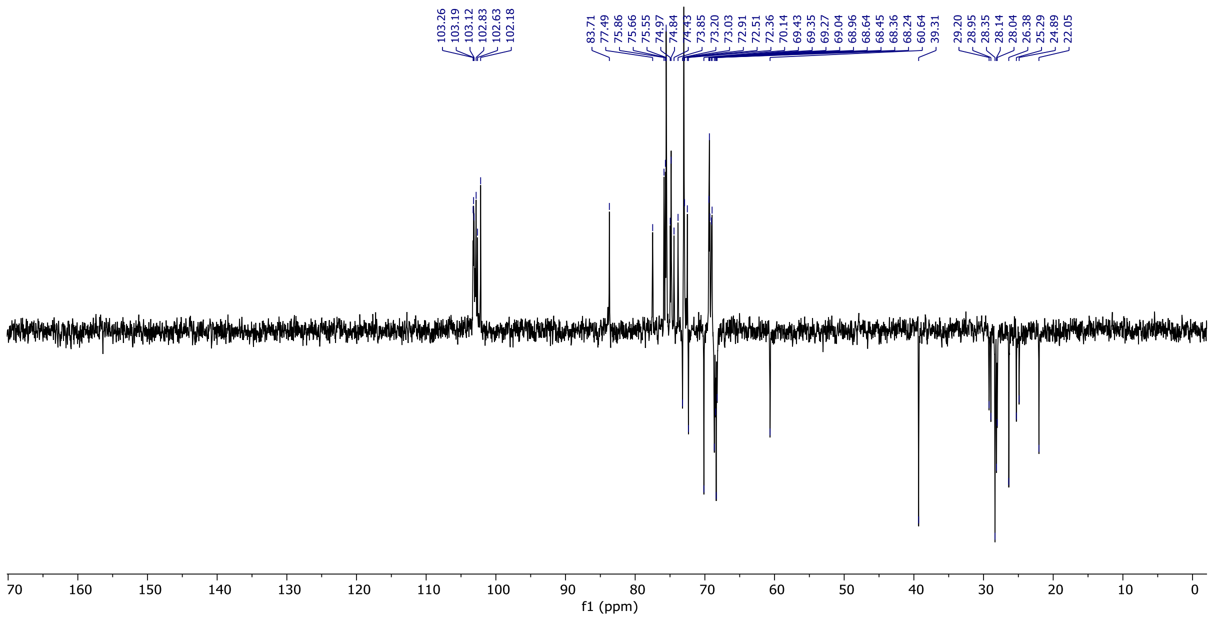

Figure S109. ^{13}C-APT NMR (700 MHz, D_2O) spectrum of **8g**.

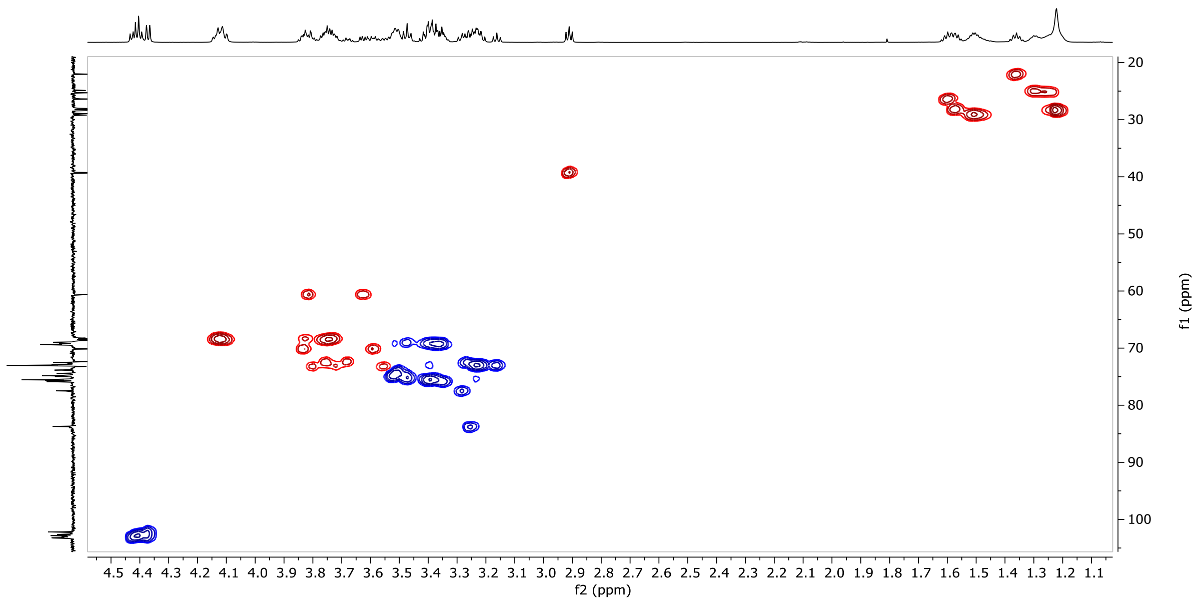

Figure S110. HSQC (700 MHz, D_2O) spectrum of **8g**.

Synthesis and analytical data of 9

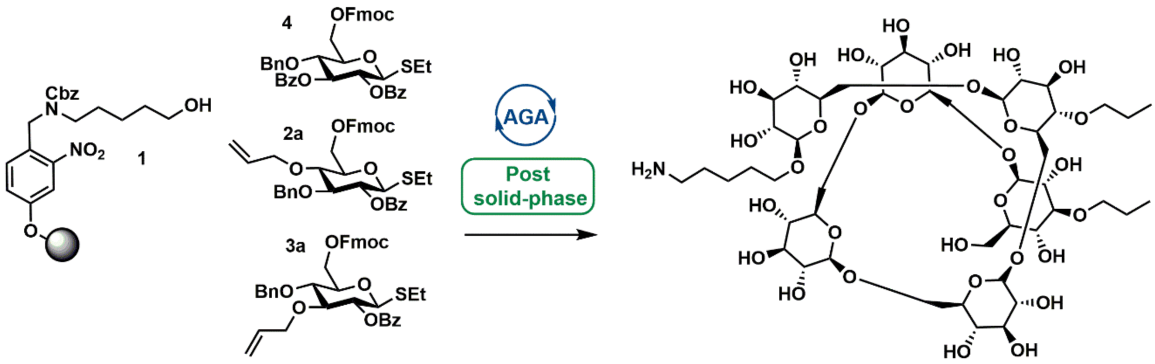

Step	Module	BB/reagent	Repeat	Notes
AGA	A	**1** (0.015 mmol)	1	Resin swelling
	B, C1, D, E	**4a** (0.09 mmol)	1	C1: NIS (0.15 mmol)
	B, C1, D, E	**2a** (0.09 mmol)	1	C1: NIS (0.15 mmol)
	B, C1, D, E	**4a** (0.09 mmol)	3	C1: NIS (0.15 mmol)
	B, C1, D, E	**3a** (0.09 mmol)	1	C1: NIS (0.15 mmol)
Post solid-phase	G	-	1	Standard cleavage
	H, I	-	-	
	J	-	-	Methods 1 and 3

After automated glycan assembly, photo-cleavage, global deprotection, purification, and lyophilisation was obtained **9** as a white solid (4.0 mg, 23 %).

R_t (Method 1) = 20.6 min.

HR-MS *m/z* = 1160.5361 $[M+H]^+$, calcd for $C_{51}H_{92}NO_{31}$: 1160.5184.

^1H NMR (700 MHz, D_2O), δ = 4.41 – 4.36 (m, 5H), 4.34 (d, *J* = 8.0 Hz, 1H), 4.11 – 4.06 (m, 5H), 3.82 – 3.77 (m, 3H), 3.76 – 3.69 (m, 6H), 3.67 – 3.61 (m, 4H), 3.61 – 3.54 (m, 3H), 3.52 – 3.45 (m, 6H), 3.45 – 3.40 (m, 1H), 3.38 – 3.30 (m, 12H), 3.25 – 3.21 (m, 4H), 3.21 – 3.16 (m, 3H), 3.13 (t, *J* = 8.5 Hz, 1H), 2.87 (t, *J* = 7.5 Hz, 2H), 1.55 (ddd, *J* = 15.5, 12.2, 7.1 Hz, 4H), 1.48 (h, *J* = 7.2 Hz, 4H), 1.33 (h, *J* = 7.5, 6.7 Hz, 2H), 0.78 (t, *J* = 7.4 Hz, 6H).

^{13}C NMR (176 MHz, D_2O), δ = 102.94, 102.91, 102.84, 102.82, 102.13, 83.87, 83.78, 75.80, 75.63, 75.48, 74.89, 74.81, 74.77, 72.98, 72.96, 72.94, 72.60, 72.55, 70.10, 69.32, 69.29, 69.04, 68.80, 68.68, 68.53, 60.55, 39.26, 28.11, 26.35, 22.60, 22.03, 9.64.

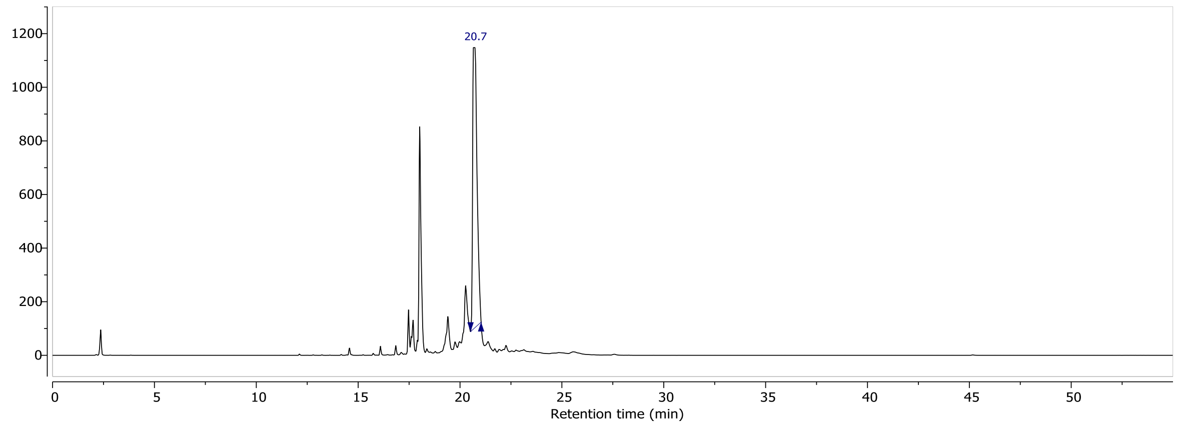

Figure S11. RP-HPLC trace of crude **9**.

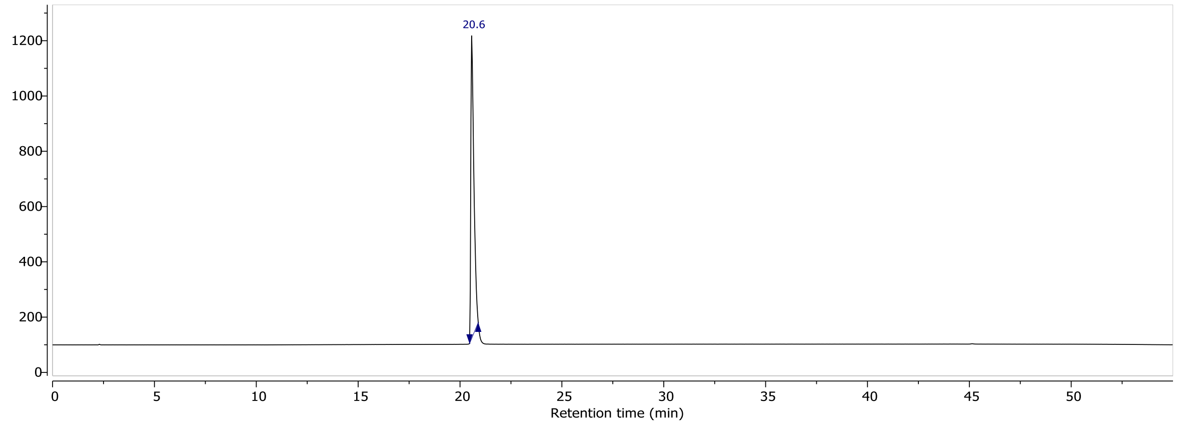

Figure S112. RP-HPLC trace of pure **9**.

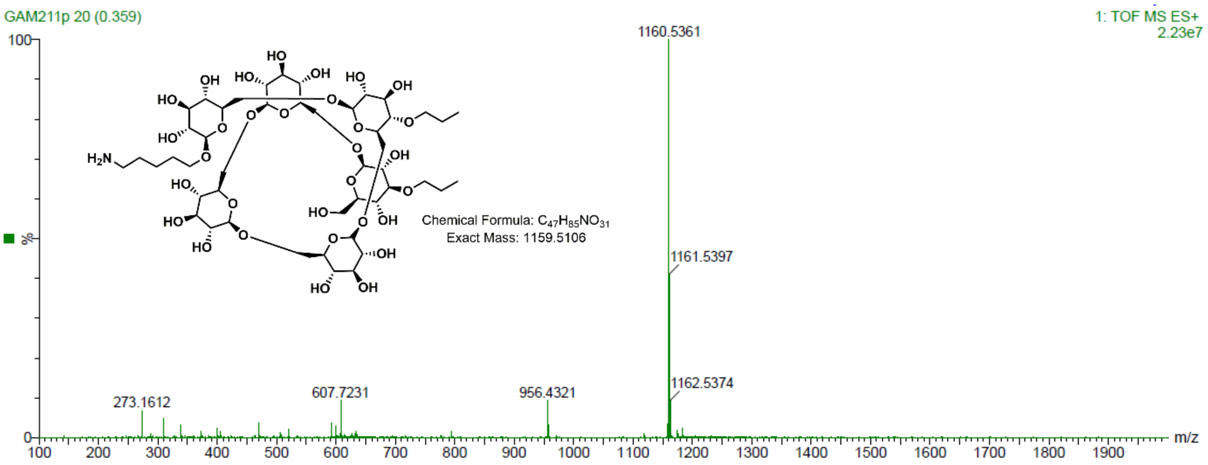

Figure S113. HR-MS of **9**.

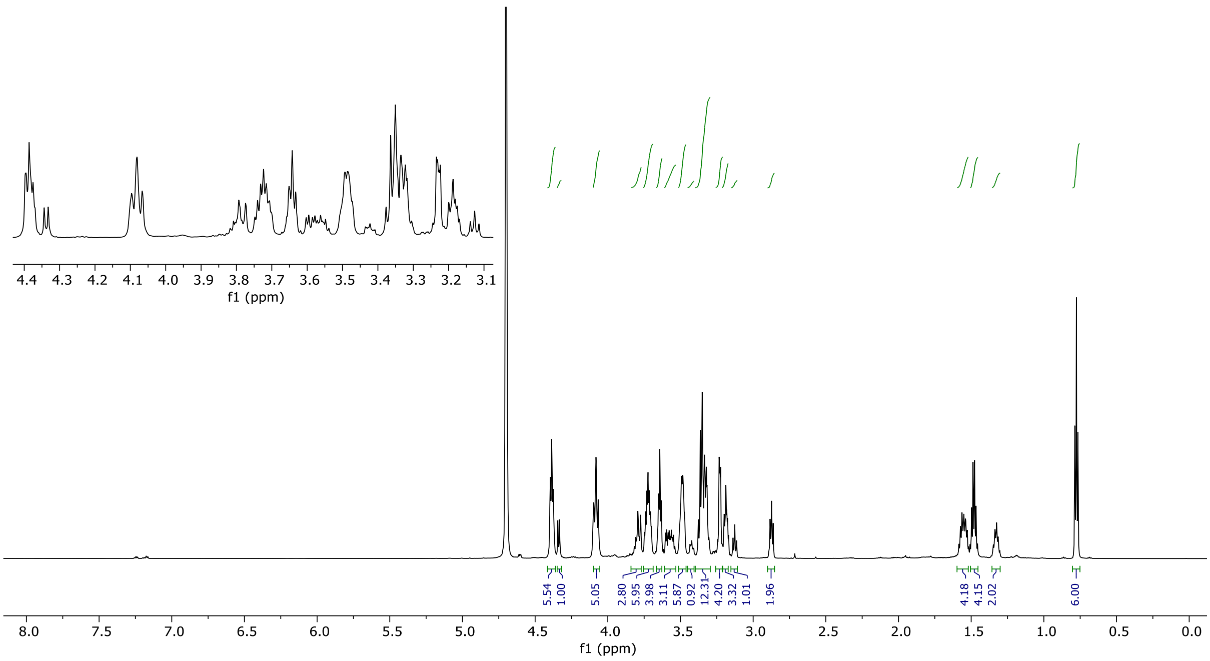

Figure S114. ^{1}H NMR (700 MHz, D_2O) spectrum of **9**.

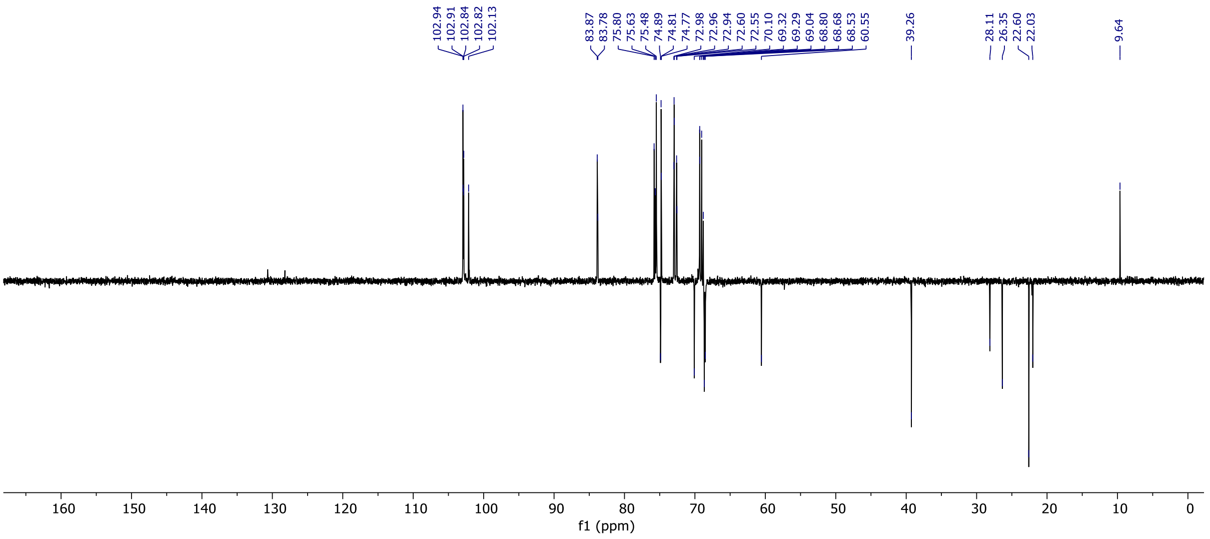

Figure S115. ^{13}C-APT NMR (700 MHz, D_2O) spectrum of **9**.

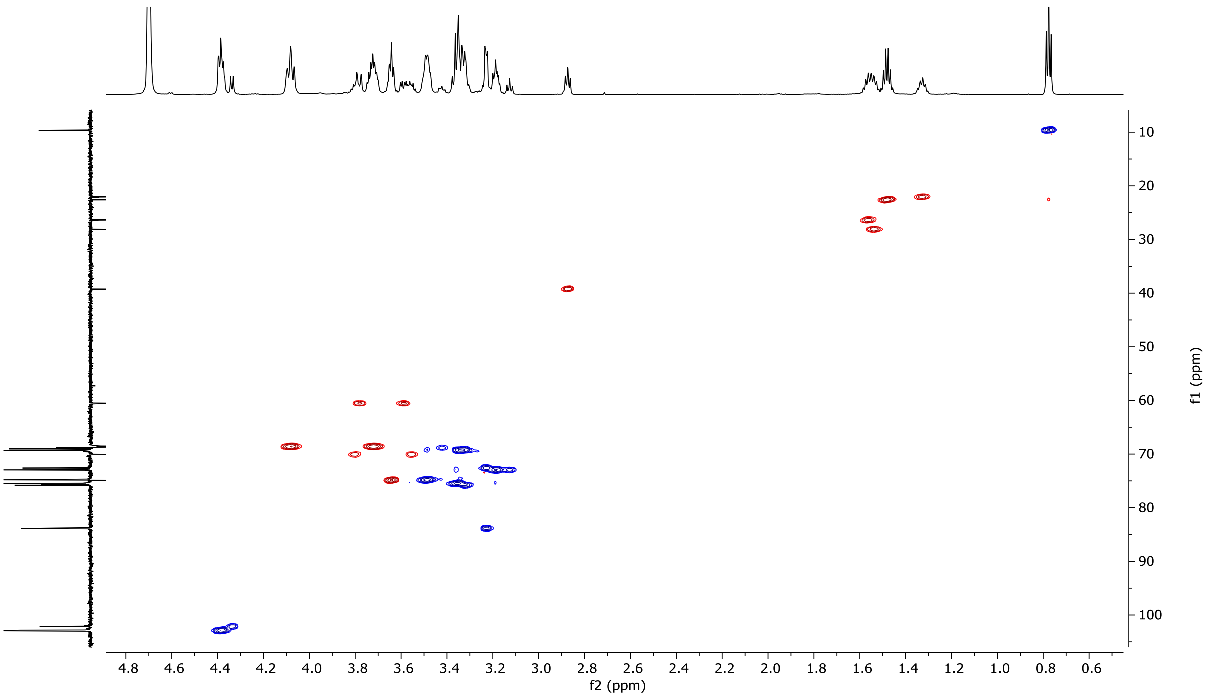

Figure S116. HSQC (700 MHz, D_2O) spectrum of **9**.

Synthesis and analytical data of 10

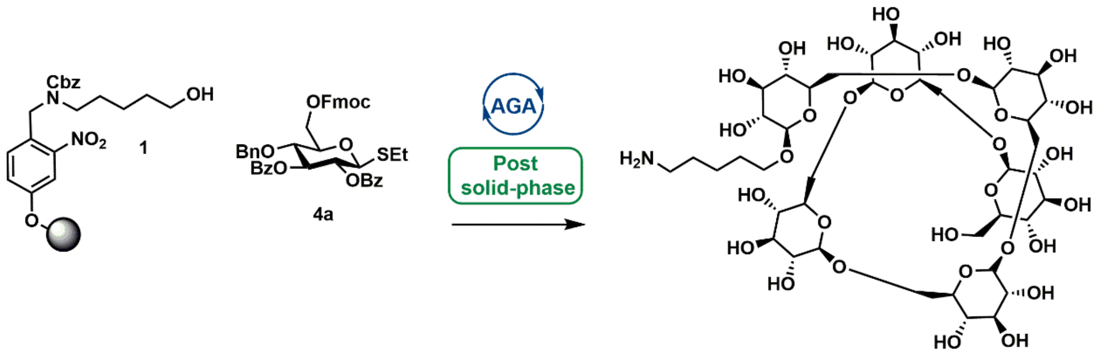

Step	Module	BB/reagent	Repeat	Notes
**AGA**	A	**1** (0.015 mmol)	1	Resin swelling
	B, C1, D, E	**4a** (0.09 mmol)	6	C1: NIS (0.15 mmol)
**Post solid-phase**	G	-	1	Standard cleavage
	H, I	-	-	
	J	-	-	Methods 1 and 3

After automated glycan assembly, photo-cleavage, global deprotection, purification and lyophilisation was obtained **10** as a white solid (3.0 mg, 19%).

R_t (Method 1) = 15.9 min.

HR-MS *m/z* = 1076.3608 $[M+H]^+$, calcd for $C_{41}H_{74}NO_{31}$: 1076.4245.

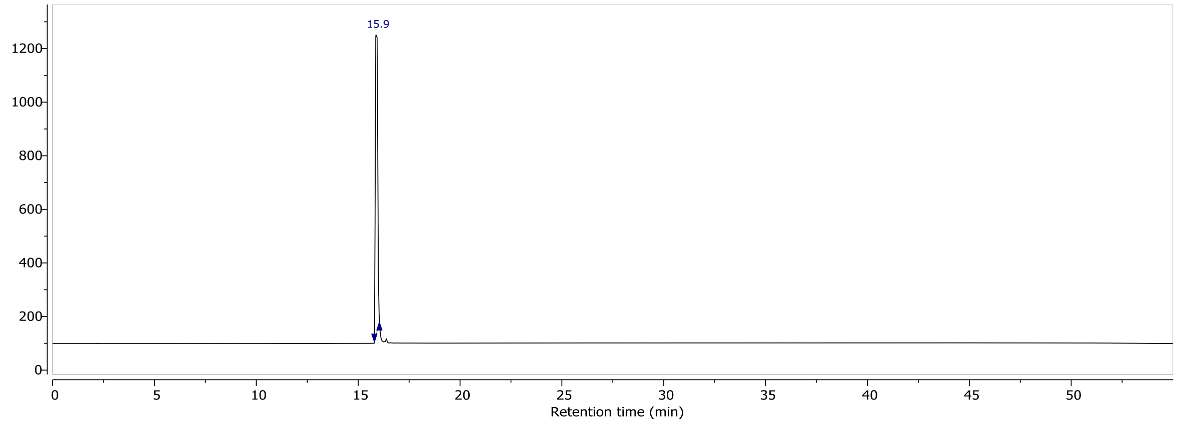

Figure S117 RP-HPLC trace of pure **10**.

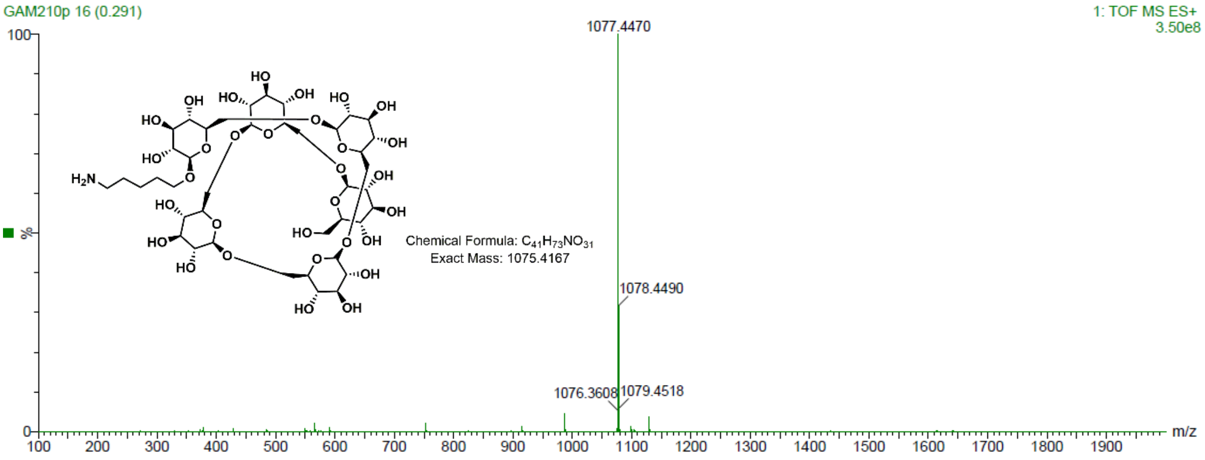

Figure S118. HR-MS of **10**.

Synthesis and analytical data of 11

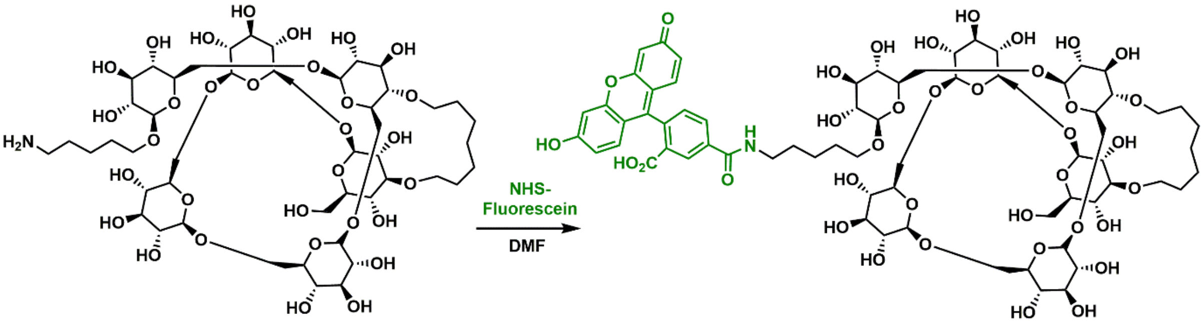

In a 2 mL vial, **8d** (0.65 mg, 0.55 µmol) is mixed with 5-carboxyfluorescein *N*-succinimidyl ester (0.77 mg, 1.65 µmol), and DIPEA (0.58 µL, 3.3 µmol) in DMF (200 µL). The mixture is stirred for 4 h and the completion is checked by RP-HPLC and ESI-MS analysis. The solvent is evaporated by a gentle stream of nitrogen and the crude mixture is dissolved in water (2 mL) and purified by RP-HPLC (Method D) to afford **11** (0.39 mg, 46%) as a pale yellow solid.

R_t (Method B) = 18.6 min.

HR-MS *m/z* = 1528.5725 [M-H]$^-$, calcd for $C_{69}H_{94}NO_{37}$: 1528.5505.

^{1}H NMR (700 MHz, D_2O) δ 8.32 (s, 1H), 7.94 (dd, *J* = 8.2, 1.7 Hz, 1H), 7.91 (d, *J* = 8.1 Hz, 1H), 7.53 (d, *J* = 1.8 Hz, 1H), 7.37 (d, *J* = 9.0 Hz, 1H), 7.10 (d, *J* = 9.2 Hz, 1H), 6.82 (dd, *J* = 9.3, 2.4 Hz, 1H), 6.79 (s, 1H), 6.73 (s, 1H), 6.70 (dd, *J* = 9.2, 2.3 Hz, 1H), 4.38 – 4.35 (m, 1H), 4.28 (dd, *J* = 8.3, 4.5 Hz, 2H), 4.22 (d, *J* = 8.0 Hz, 1H), 4.12 (dd, *J* = 9.3, 3.2 Hz, 2H), 4.08 (dd, *J* = 11.7, 2.2 Hz, 1H), 4.00 (s, 1H), 3.88 – 3.83 (m, 1H), 3.82 – 3.77 (m, 2H), 3.74 (dt, *J* = 9.2, 5.9 Hz, 2H), 3.71 – 3.65 (m, 4H), 3.61 (q, *J* = 5.6, 4.8 Hz, 3H), 3.60 – 3.52 (m, 3H), 3.50 (d, *J* = 7.9 Hz, 3H), 3.44 – 3.33 (m, 10H), 3.32 – 3.21 (m, 15H), 3.20 – 3.08 (m, 8H), 3.08 – 3.03 (m, 2H), 2.93 (s, 1H), 1.57 – 1.48 (m, 5H), 1.48 (s, 3H), 1.40 (q, *J* = 9.6, 6.5 Hz, 3H), 1.32 – 1.26 (m, 3H), 1.23 (d, *J* = 7.0 Hz, 1H), 1.20 – 1.13 (m, 5H).

^{13}C NMR (176 MHz, D_2O), δ = 103.85, 103.44, 103.16, 102.96, 102.81, 102.38, 83.26, 77.21, 77.21, 75.73, 75.60, 75.45, 75.01, 74.52, 74.04, 73.57, 73.22, 72.97, 72.89, 72.79, 72.21, 70.57, 69.98, 69.04, 69.03, 69.00, 68.96, 68.32, 68.02, 67.96, 67.65, 60.83, 31.45, 29.24, 28.89, 28.38, 28.03, 27.99, 27.73, 25.56, 25.27, 22.08.

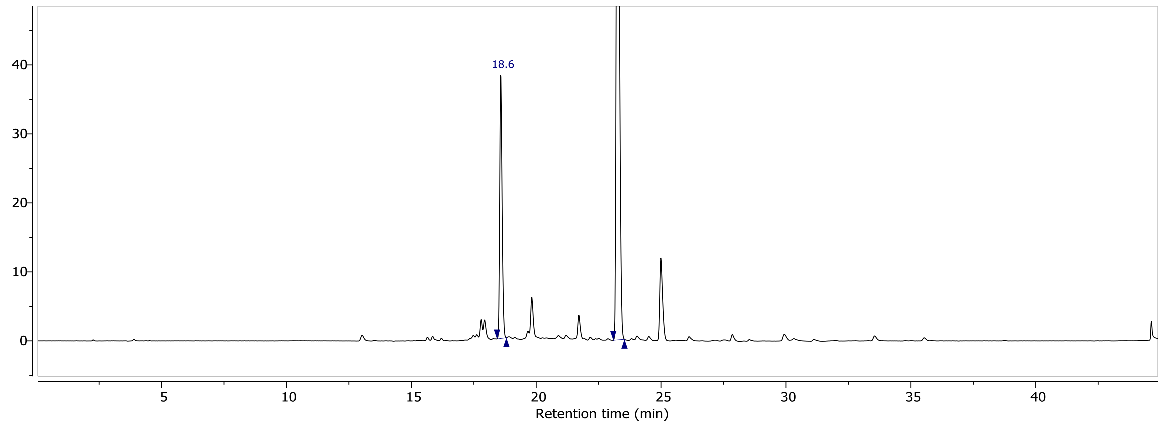

Figure S119. RP-HPLC trace of crude **11**.

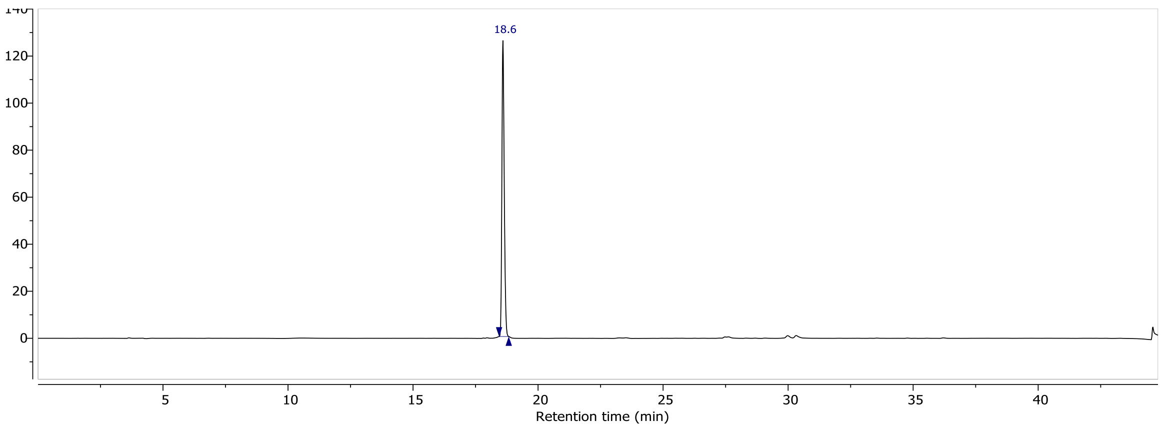

Figure S120. RP-HPLC trace of pure **11**.

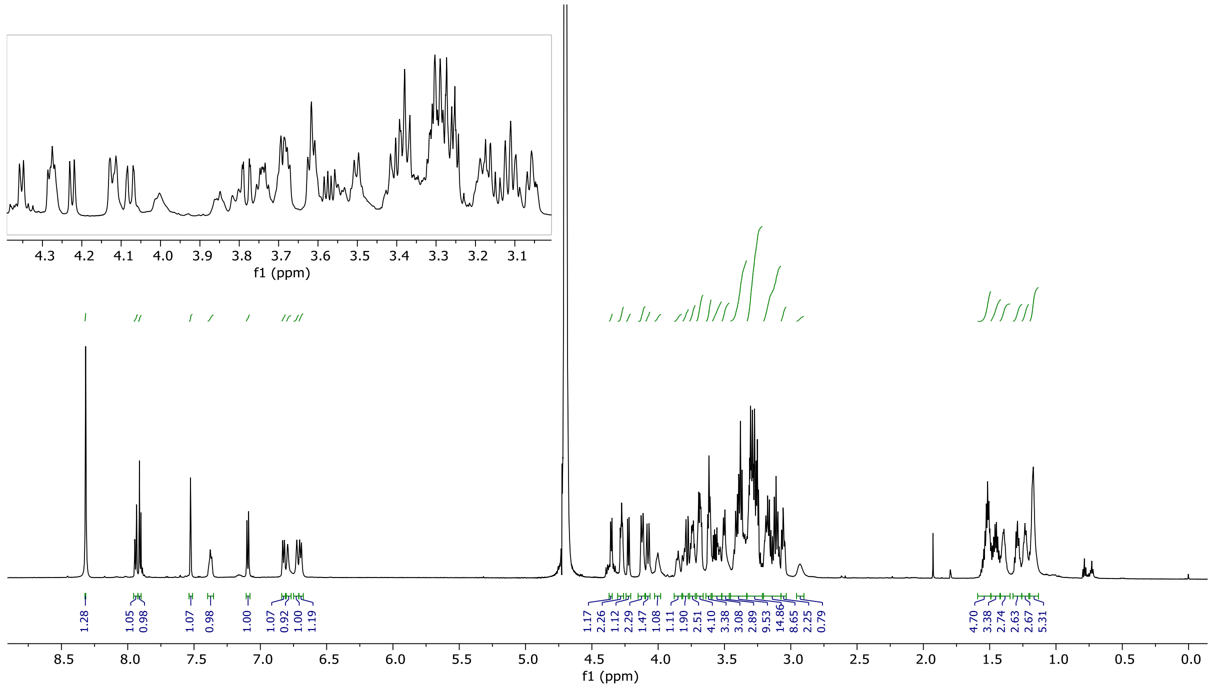

Figure S121. ^{13}C NMR (700 MHz, D_2O) spectrum of **11**.

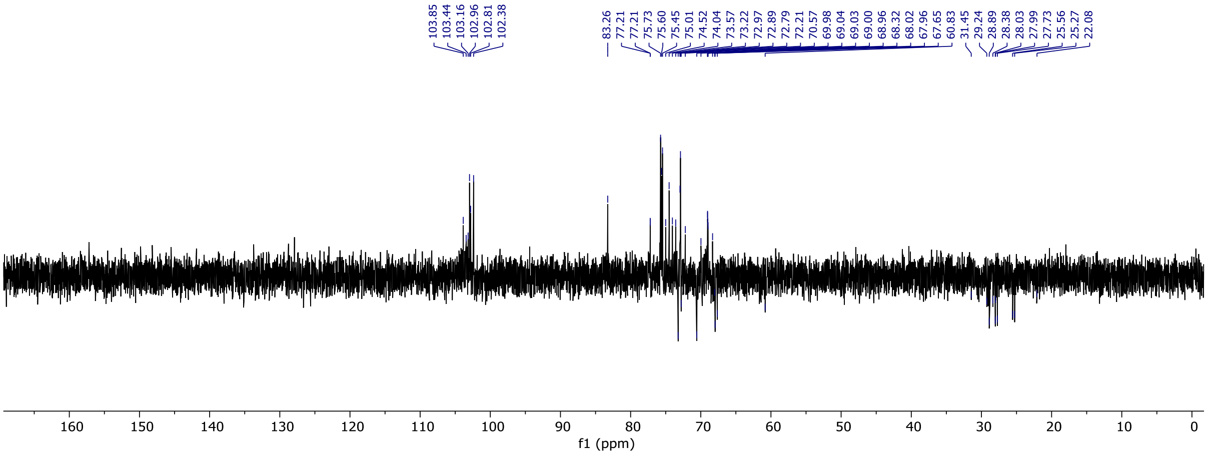

Figure S122. ^{13}C-APT NMR (700 MHz, D_2O) spectrum of **11**.

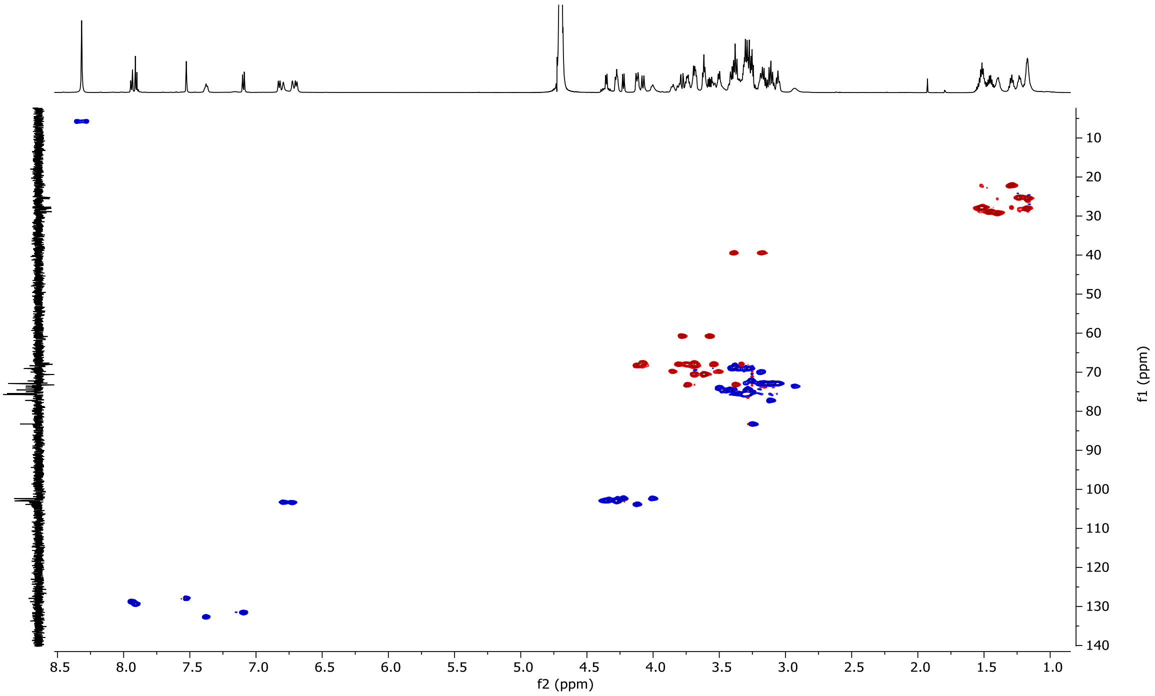

Figure S123. HSQC (700 MHz, D_2O) spectrum of **11**.

Synthesis and analytical data of 12

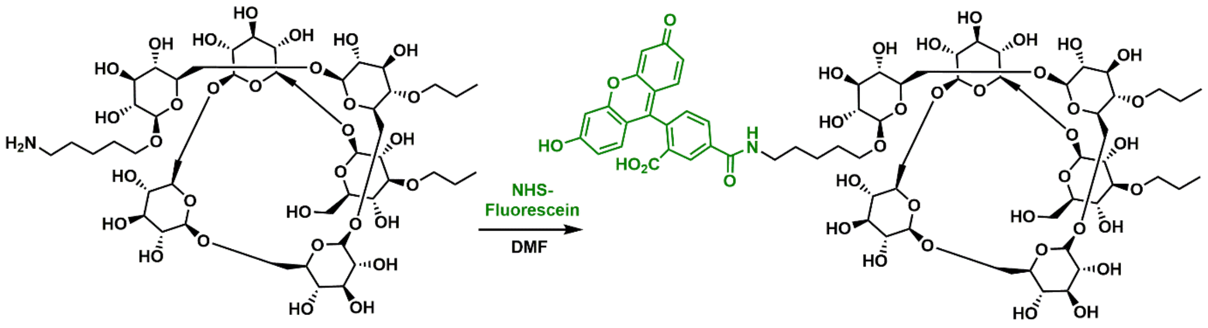

In a 2 mL vial, **9** (1.6 mg, 1.4 µmol) is mixed with 5-carboxyfluorescein *N*-succinimidyl ester (1.3 mg, 2.8 µmol), and DIPEA (1.0 µL, 5.6 µmol) in DMF (400 µL). The mixture is stirred for 4 h and the completion is checked by RP-HPLC and ESI-MS analysis. The solvent is evaporated by a gentle stream of nitrogen and the crude mixture is dissolved in water (2 mL) and purified by RP-HPLC (Method D) to afford **12** (1.2 mg, 58%) as a pale yellow solid.

R_t (Method B) = 19.3 min.

HR-MS *m/z* = 1516.5746 [M-H]$^-$, calcd for $C_{68}H_{94}NO_{37}$: 1516.5505.

^{1}H NMR (600 MHz, D_2O) δ 8.33 (s, 1H), 7.94 (d, *J* = 8.2 Hz, 2H), 7.49 (s, 2H), 7.02 (s, 3H), 6.79 (d, *J* = 22.3 Hz, 2H), 6.66 (s, 3H), 4.39 – 4.30 (m, 5H), 4.27 (d, *J* = 7.3 Hz, 1H), 4.10 – 3.98 (m, 6H), 3.79 (dd, *J* = 12.3, 2.0 Hz, 1H), 3.77 – 3.67 (m, 4H), 3.67 – 3.58 (m, 7H), 3.54 (s, 1H), 3.50 – 3.16 (m, 25H), 3.12 (dd, *J* = 9.1, 8.0 Hz, 2H), 1.59 – 1.41 (m, 8H), 1.31 (s, 2H), 0.79 (t, *J* = 7.4 Hz, 3H), 0.76 (t, *J* = 7.4 Hz, 3H).

^{13}C NMR (151 MHz, D_2O) δ 103.27, 102.97, 102.87, 102.76, 102.33, 102.30, 83.89, 83.82, 76.11, 75.84, 75.84, 75.69, 75.69, 75.57, 75.54, 75.54, 75.50, 75.50, 74.83, 74.80, 74.74, 73.04, 72.98, 72.62, 72.62, 72.51, 72.51, 70.51, 69.34, 69.05, 68.77, 68.77, 68.74, 68.71, 68.67, 68.58, 60.63.

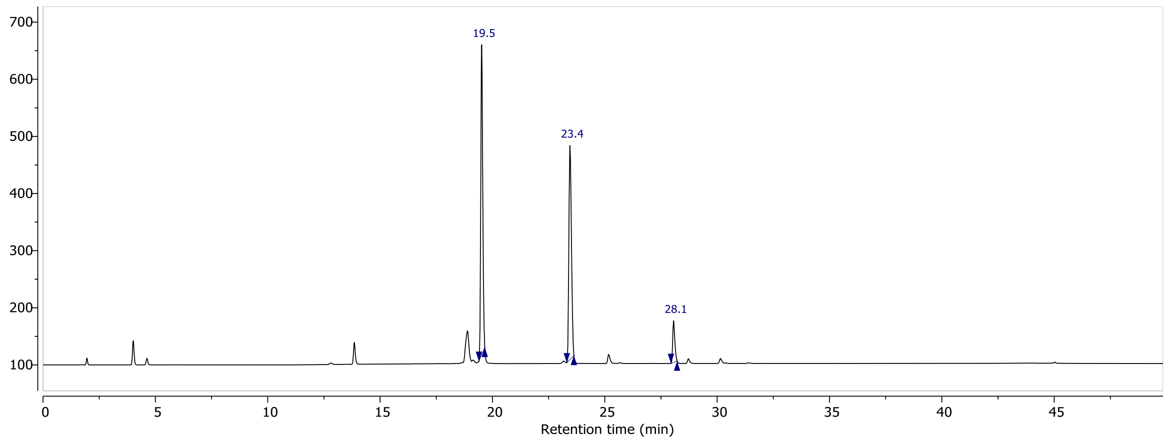

Figure S124. RP-HPLC trace of crude **12**.

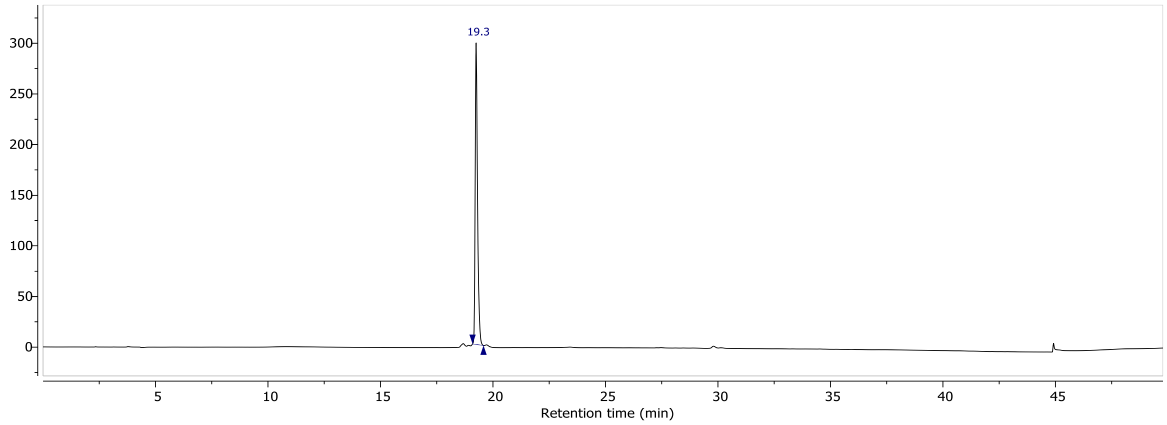

Figure S125. RP-HPLC trace of pure **12**.

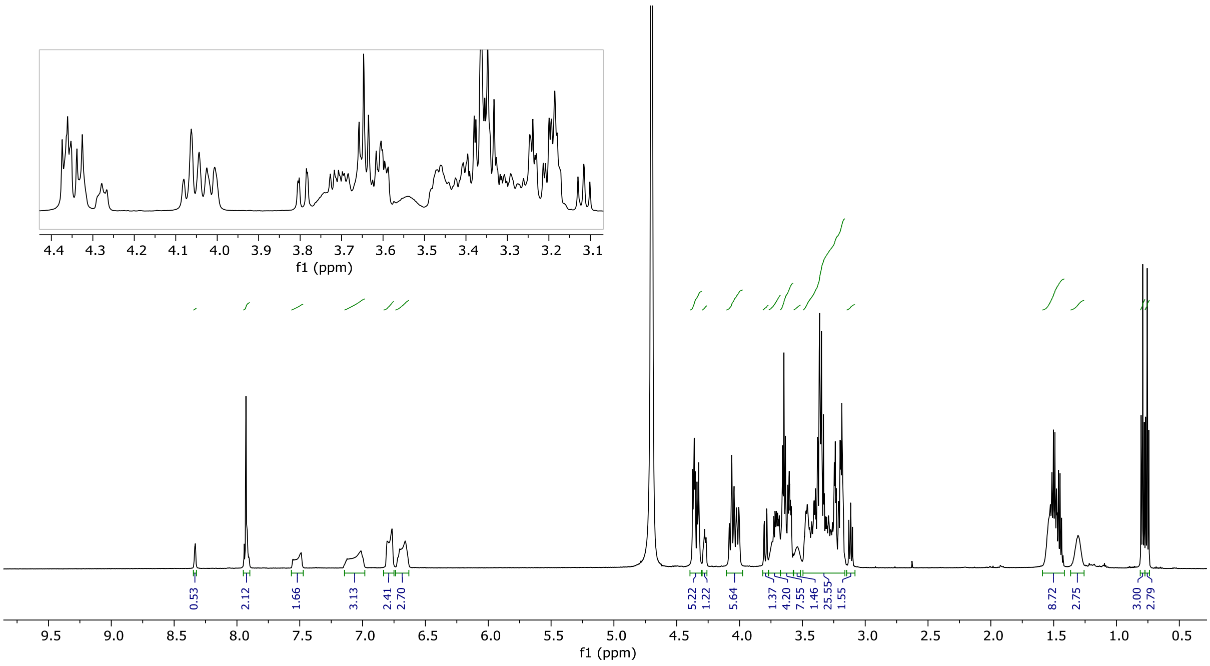

Figure S126. ^{1}H NMR (700 MHz, D_2O) spectrum of **12**.

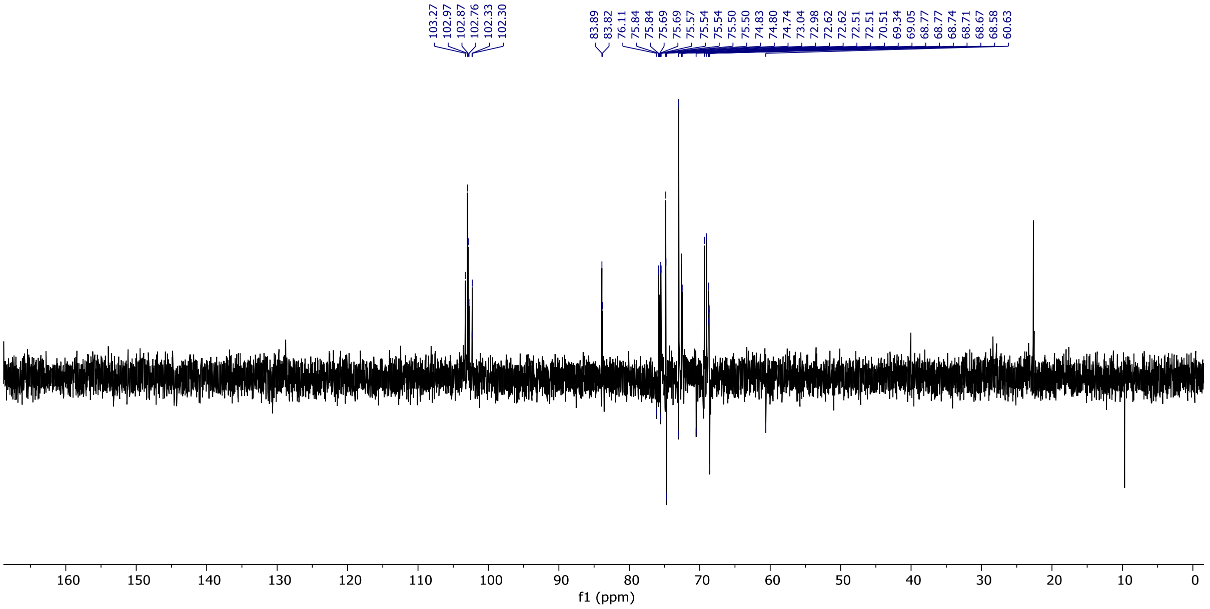

Figure S127. ^{13}C-APT NMR (700 MHz, D_2O) spectrum of **12**.

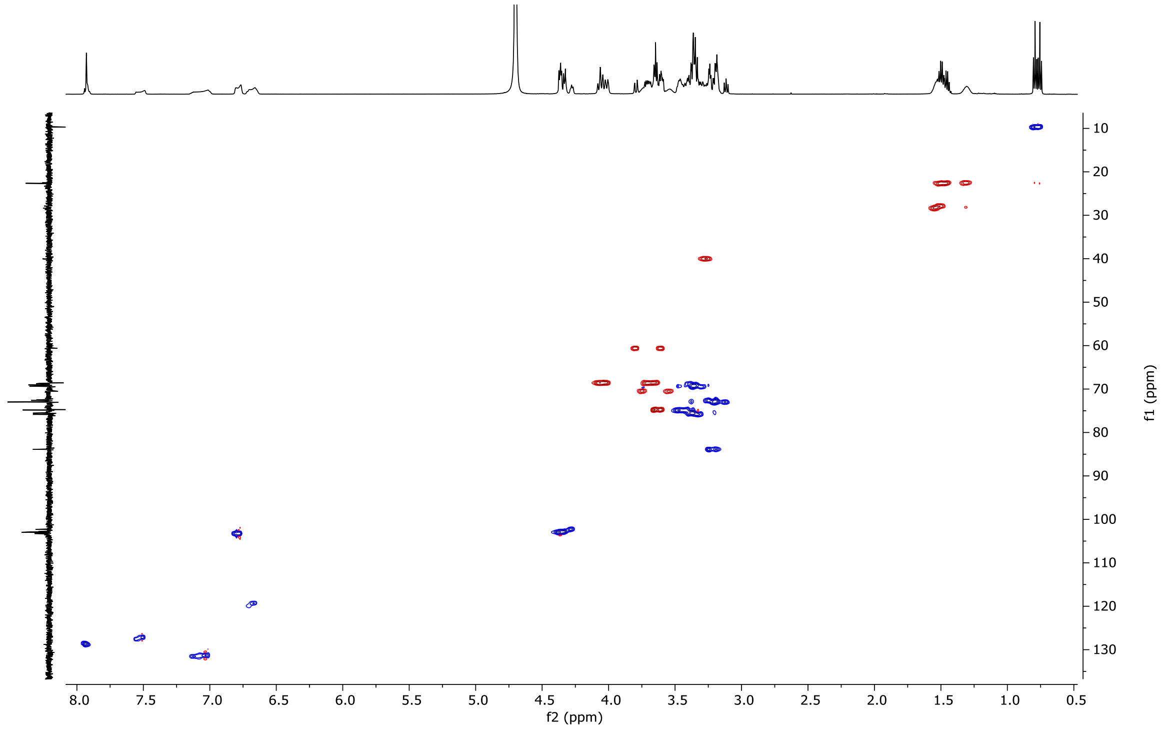

Figure S128. HSQC (700 MHz, D_2O) spectrum of **12**.

Synthesis and analytical data of 13

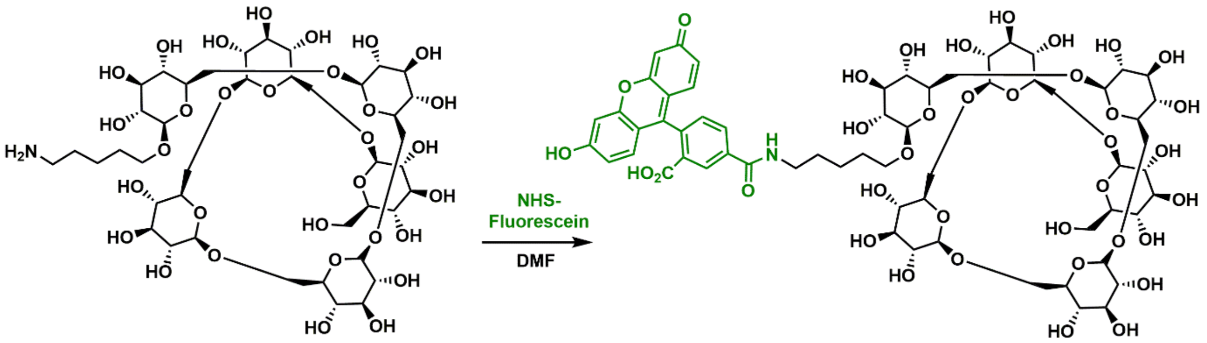

In a 2 mL vial, **10** (1.7 mg, 1.6 µmol) is mixed with 5-carboxyfluorescein *N*-succinimidyl ester (1.5 mg, 3.2 µmol), and DIPEA (1.1 µL, 6.4 µmol) in DMF (400 µL). The mixture is stirred for 4 h and the completion is checked by RP-HPLC and ESI-MS analysis. The solvent is evaporated by a gentle stream of nitrogen and the crude mixture is dissolved in water (2 mL) and purified by RP-HPLC (Method D) to afford **13** (1.5 mg, 66%) as a pale yellow solid.

R_t (Method B) = 17.7 min.

HR-MS *m/z* = 1432.4698 [M-H]$^-$, calcd for $C_{62}H_{82}NO_{37}$: 1432.4566.

^{1}H NMR (600 MHz, D_2O) δ 8.31 (s, 1H), 7.95 (d, *J* = 8.2 Hz, 1H), 7.91 (dd, *J* = 8.2, 1.6 Hz, 1H), 7.48 (d, *J* = 9.2 Hz, 1H), 6.97 (d, *J* = 9.3 Hz, 2H), 6.78 (q, *J* = 3.7, 2.4 Hz, 1H), 6.68 – 6.62 (m, 2H), 4.39 – 4.31 (m, 4H), 4.27 (d, *J* = 8.0 Hz, 1H), 4.09 – 3.99 (m, 4H), 3.80 (dd, *J* = 12.4, 2.2 Hz, 1H), 3.76 – 3.58 (m, 6H), 3.53 – 3.28 (m, 14H), 3.25 – 3.16 (m, 5H), 3.11 (t, *J* = 8.3 Hz, 1H), 1.55 – 1.43 (m, 4H), 1.32 – 1.25 (m, 2H).

^{13}C NMR (176 MHz, D_2O) δ 131.13, 128.80, 103.12, 102.90, 102.78, 102.67, 102.24, 75.81, 75.59, 75.56, 75.47, 75.45, 74.80, 74.77, 74.67, 72.97, 72.92, 72.87, 70.40, 69.29, 69.26, 69.21, 68.57, 68.45, 68.45, 60.60, 39.98, 28.29, 27.81, 22.46.

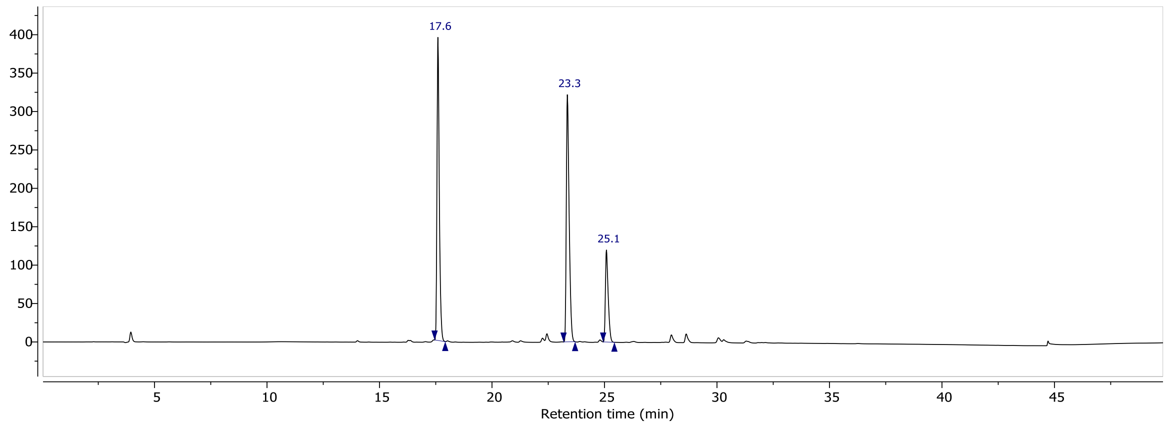

Figure S129. RP-HPLC trace of crude **13**.

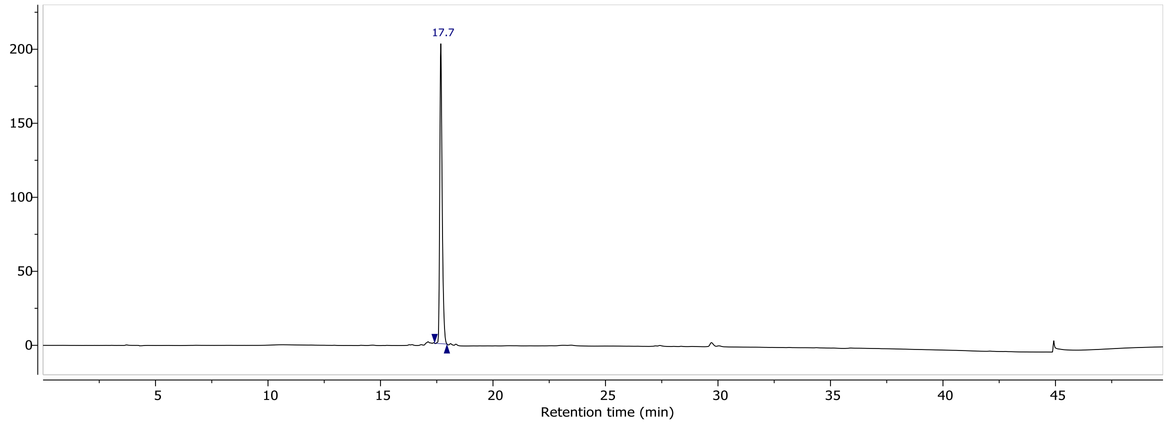

Figure S130. RP-HPLC trace of pure **13**.

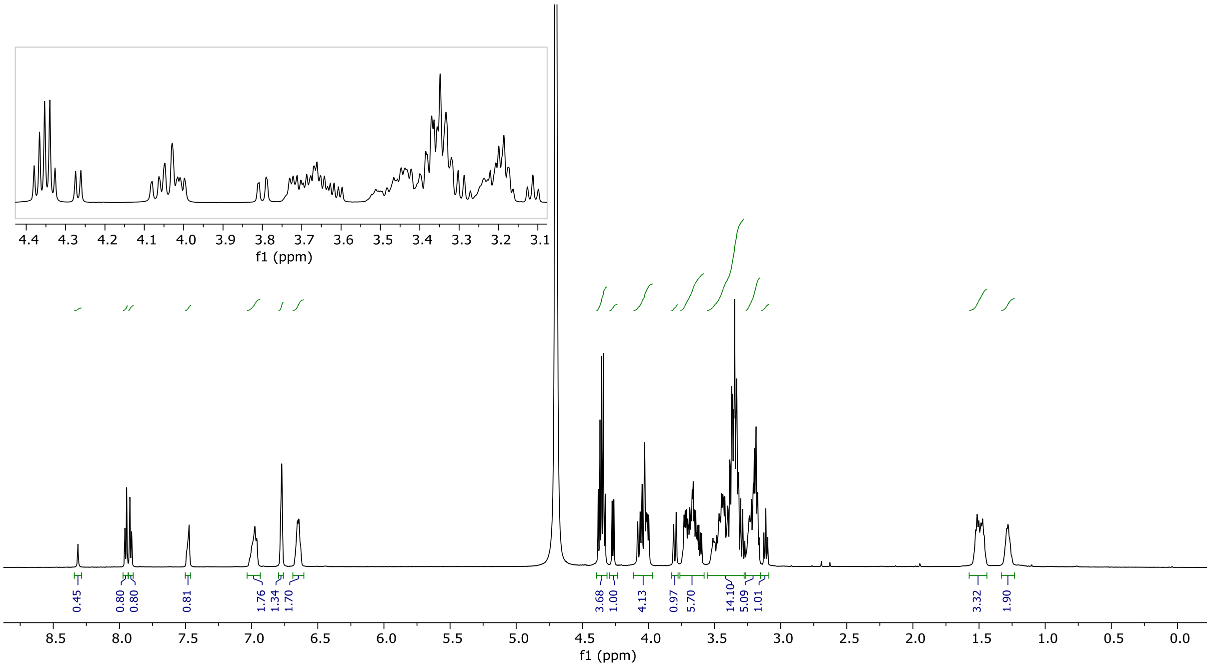

Figure S131. ^{1}H NMR (700 MHz, D_2O) spectrum of **13**.

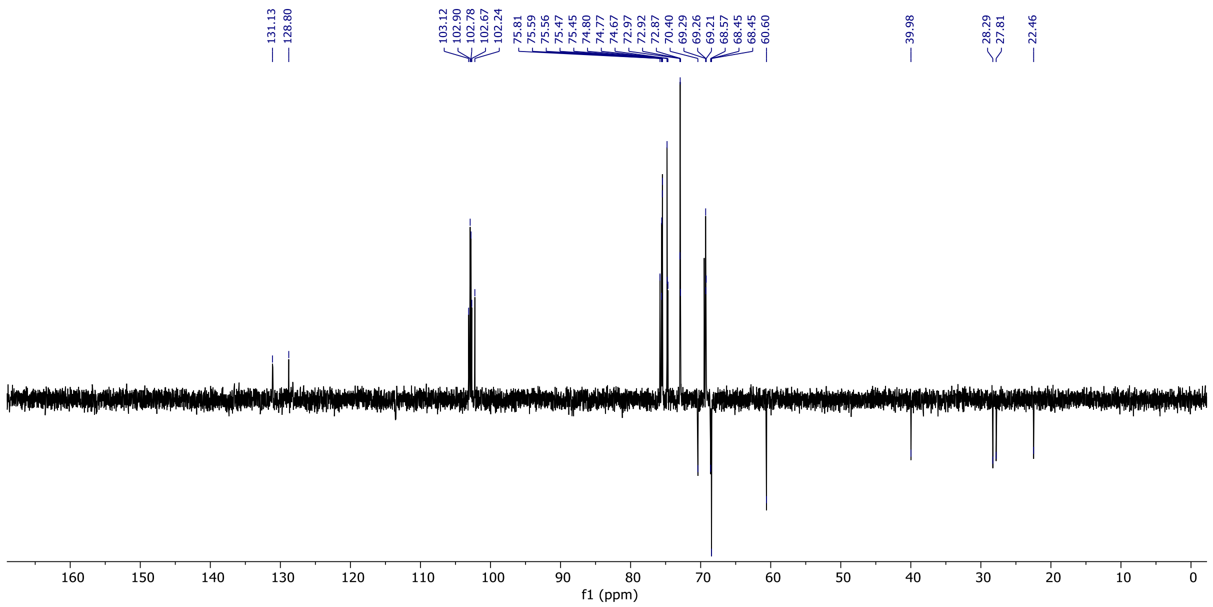

Figure S132. ^{13}C-APT NMR (700 MHz, D_2O) spectrum of **13**.

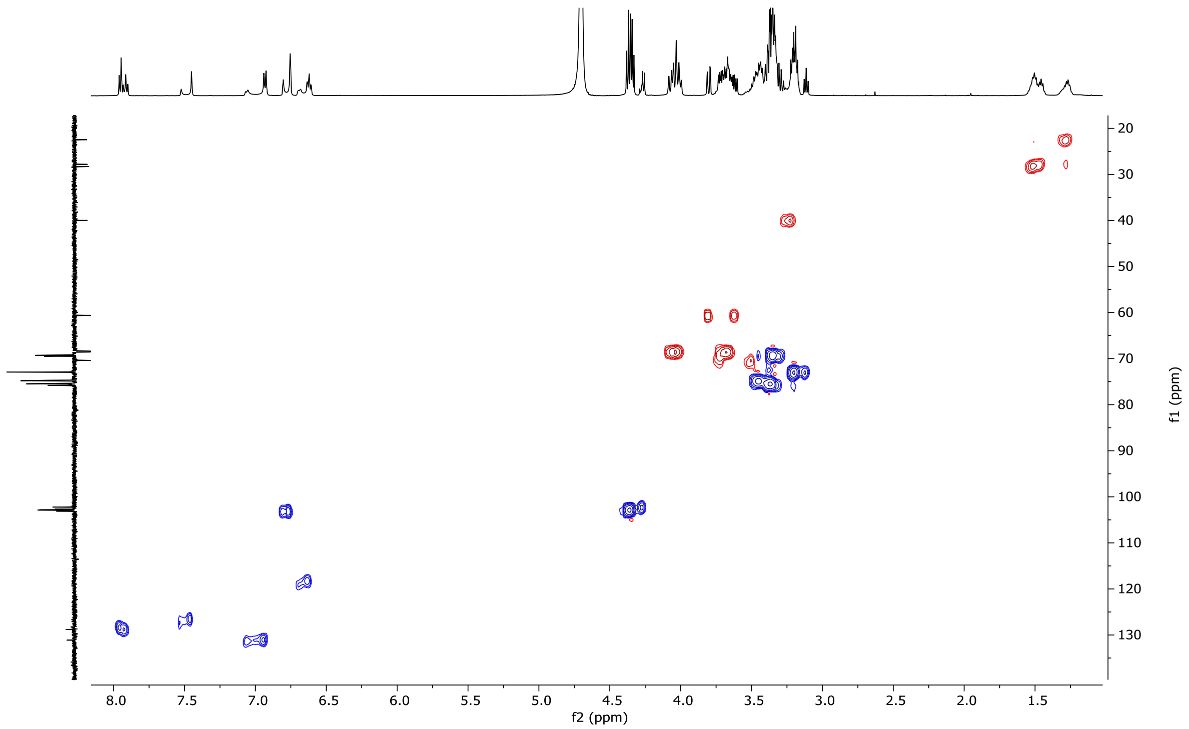

Figure S133. HSQC (700 MHz, D_2O) spectrum of **13**.

4. FACS analysis of glycan-treated cells

Jurkat cells were plated in a 96-well round bottom plate at a concentration of 2 x 10^5 cells per well in 200 µL serum-free media. Cells were incubated with 10 µM of each compound (**11**, **12**, and **13**) at 4°C in a fridge or 37°C in 5% CO_2. Cell viability of each treatment condition was monitored in a separated plate via 7-AAD live/dead staining (data not shown). After incubation, cells were harvested, washed with ice-cold PBS and the mean fluorescence was measured and analysed using a BD FACSCanto II Flow Cytometer and FlowJo Software (BD), respectively.

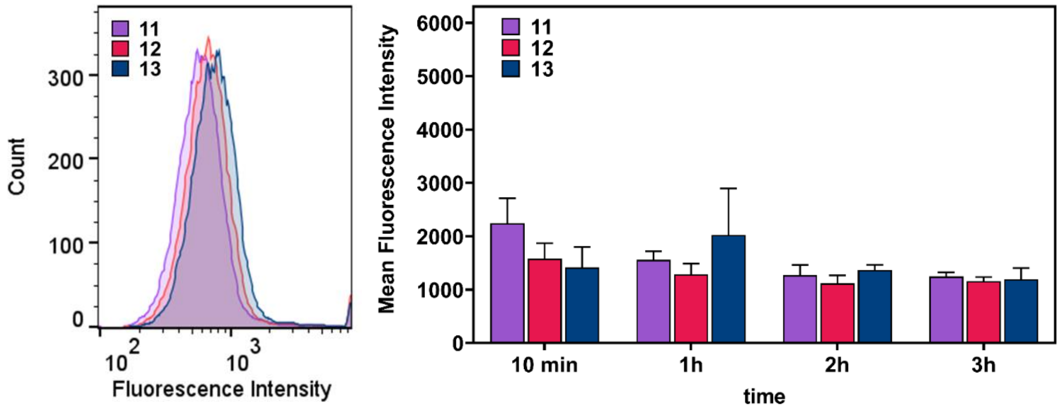

Figure S134. Flow cytometry histogram of incubation of Jurkat cells with glycans **11**, **12** and **13** at 4°C and after 3 h. Quantification of flow cytometry after 10 min, 1 h, 2 h, and 3 h of incubation.

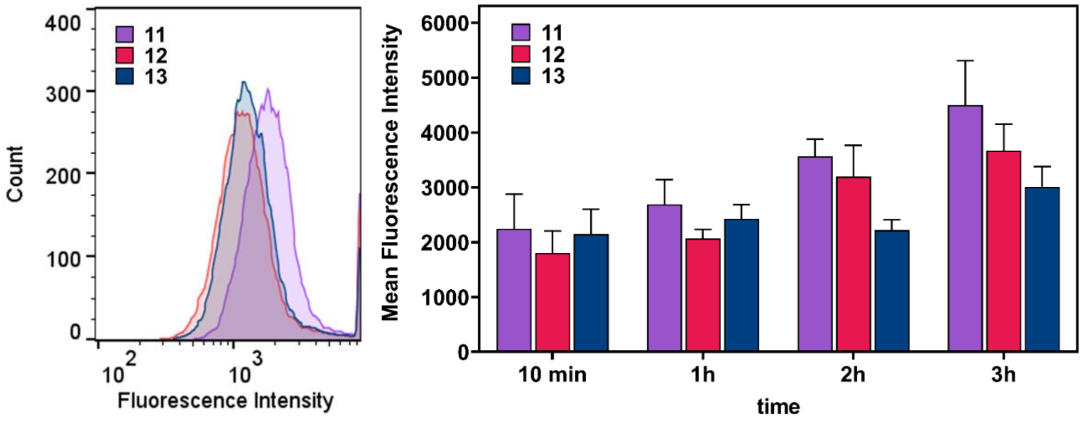

Figure S135. Flow cytometry histogram of incubation of Jurkat cells with glycans **11**, **12** and **13** at 37°C and after 3 h. Quantification of flow cytometry after 10 min, 1 h, 2 h, and 3 h of incubation.

5. Confocal microscopy

For the cell penetration studies, 1 × 10^6 Jurkat T-cell leukemia cells were washed once with serum-free RPMI and resuspended in 50 µL of fluorescein-conjugated glycans (**11**, **12**, and **13**; 20 µM) in serum-free RPMI. Cells were incubated for 3 h at 37°C. After three washes with PBS, cells were resuspended in PBS and settled on 12 mm coverslips in 24-well plates for 30 min in the dark at RT and fixed for 15 min with 4% paraformaldehyde and 0.2% glutaraldehyde. Afterward, the cells were washed three times with PBS and the coverslips were flipped onto a microscopy glass slide prepared with Roti®Mount FluorCare DAPI mounting solution (Carl Roth, Darmstadt, Berlin) and sealed with transparent nail polish. The slides were examined and imaged under a Zeiss LSM700 laser scanning confocal microscope (Zeiss). The images were analysed using Fiji software by measuring the fluorescence intensity. The statistical analysis was performed with the software GraphPad Prism 9.3.1 (GraphPad Software, Inc.).

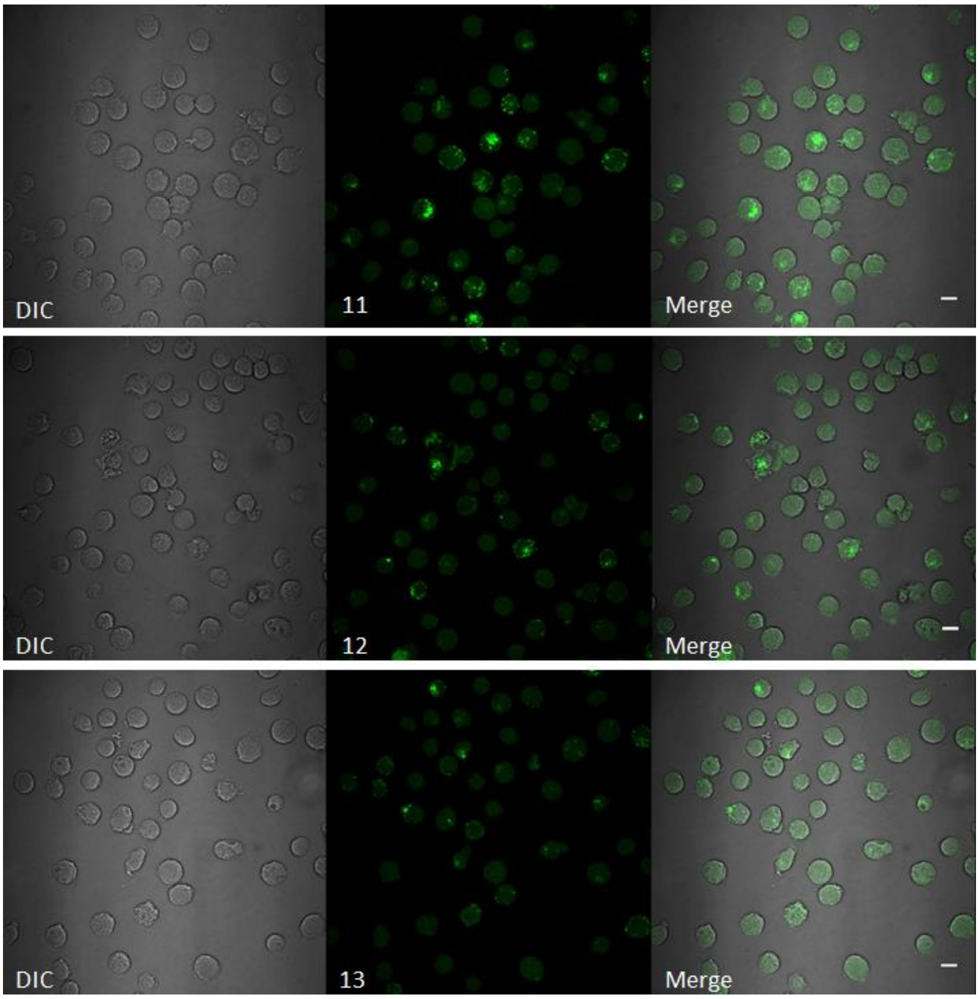

Figure S136. Confocal fluorescence microscopy images of Jurkat cells incubated with the glycans **11**, **12** and **13**, respectively, for 3 h at 37°C. Scale bars correspond to 5µm.

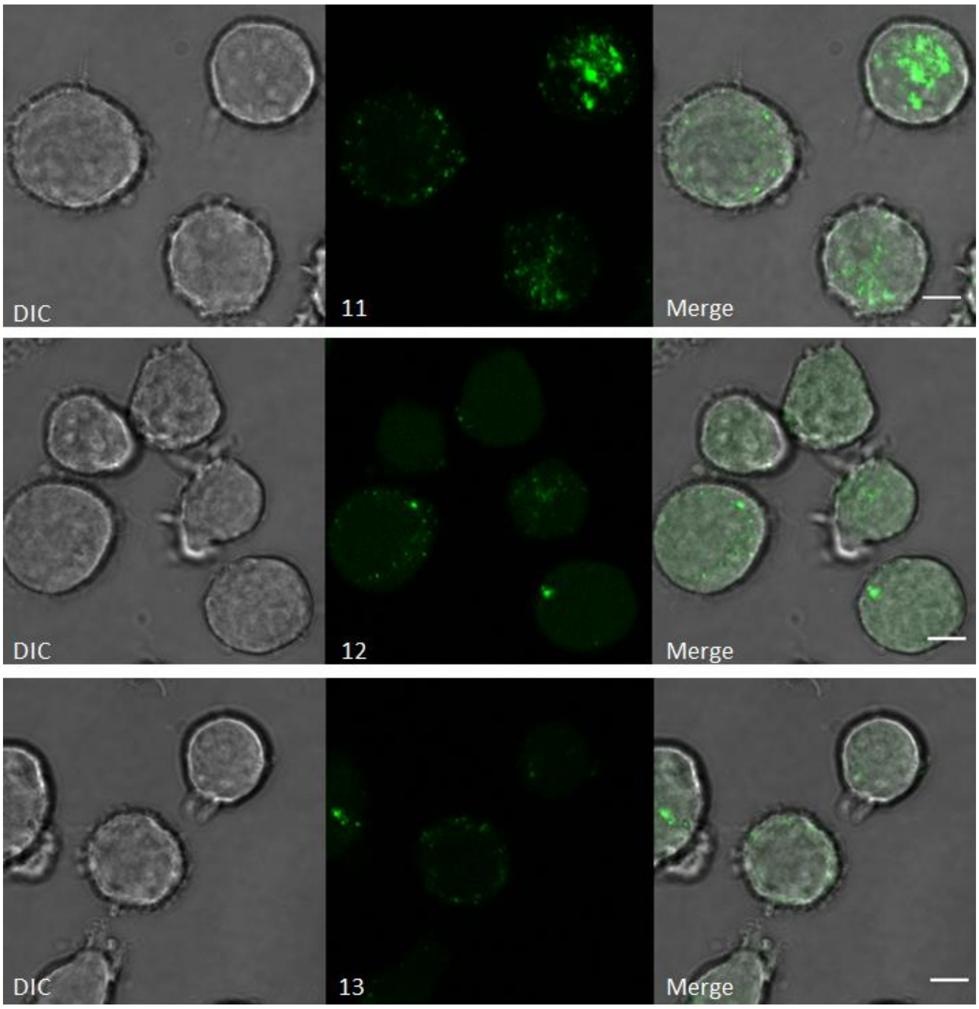

Figure S137. Zoom of the confocal fluorescence microscopy images of Jurkat cells incubated with the glycans **11**, **12** and **13**, respectively, for 3 h at 37°C. Scale bars correspond to 5µm.

6. Enzymatic stability assay

Glycans (0.5 mM) were incubated with β-(1,6)-Endoglucanase (10 µM) in Acetate buffer (20 mM, pH 5.5) in a thermic block at 60°C. The progress of the enzymatic reactions was analysed after 0, 5, 10, 15, 20, 25, 30, 40, 50, and 60 min for stapled glycan **8d**; after 0, 5, 10, 15, and 20 min for alkylated glycan **9**, and after 0, 1, 2, and 3 min for linear glycan **10**. For that, 10 µM aliquots of the reaction mixture were quenched over NaOH (0.1 M, 10 µL) and the resulting solution was analysed by HPLC-based peak detection, monitoring the SIM trace (positive mode), corresponding to m/z = 1172.5, 1160.5, and 1076.4 molecular ions $[M+H]^+$ of **8d**, **9**, and **10** respectively. The statistical analysis was performed with the software GraphPad Prism 9.3.1 (GraphPad Software, Inc.) using a nonlinear regression.

HPLC-peak integral in SIM detection m/z = 1172.5, $[M+H]^+$ of **8d.**

Time (min)	Exp. 1	Normalized (Glycan %)	Exp. 2	Normalized (Glycan %)	Exp. 3	Normalized (Glycan %)
0	4694.33	100	5863.71	100	6887.48	100
5	4036.84	85.99395	5199.21	88.66758	5628.02	81.71378
10	3614.38	76.99459	4639.35	79.1197	4730.83	68.68739
15	3057.22	65.1258	3660.42	62.42498	3972.26	57.67363
20	2641.6	56.27214	3114.72	53.11859	3475.74	50.46461
25	2213.49	47.15242	2488.46	42.43832	2724.11	39.55162
30	1706.24	36.34683	2212.54	37.73277	2411.63	35.01469
40	1163.64	24.7882	1576.01	26.87735	1543.5	22.41023
50	717.22	15.27843	1009.3	17.21265	1012.7	14.70349
60	464.58	9.89662	700.95	11.95404	655.3	9.51437

HPLC-peak integral in SIM detection m/z = 1160.5, $[M+H]^+$ of **9**.

Time (min)	Exp. 1	Normalized (Glycan %)	Exp. 2	Normalized (Glycan %)	Exp. 3	Normalized (Glycan %)
0	9063.1	100	11539.51	100	6842.75	100
5	4510.55	49.76829	7002.43	60.68221	3194.2	46.68006
10	1610.36	17.76831	2669.78	23.13599	1326.87	19.39089
15	230.56	2.54394	1006	8.71787	387.33	5.66044

HPLC-peak integral in SIM detection m/z = 1076.4, $[M+H]^+$ of **10.**

**Time (min)**	**Exp. 1**	**Normalized (Glycan %)**	**Exp. 2**	**Normalized (Glycan %)**	**Exp. 3**	**Normalized (Glycan %)**
0	10265.23	100	11206.74	100	9065.03	100
1	4596.77	44.78	5931.72	52.92993	4240.64	46.78021
2	1142.51	11.1299	1118.86	9.98381	525.97	5.80219
3	56.1	0.54651	137	1.22248	51.86	0.57209

7. References

(1) Danglad-Flores, J.; Leichnitz, S.; Sletten, E. T.; Abragam Joseph, A.; Bienert, K.; Le Mai Hoang, K.; Seeberger, P. H. Microwave-Assisted Automated Glycan Assembly. *J. Am. Chem. Soc.* **2021**, *143*, 8893–8901.

(2) Pardo-Vargas, A.; Delbianco, M.; Seeberger, P. H. Automated Glycan Assembly as an Enabling Technology. *Curr. Opin. Chem. Biol.* **2018**, *46*, 48–55.

(3) Le Mai Hoang, K.; Pardo-Vargas, A.; Zhu, Y.; Yu, Y.; Loria, M.; Delbianco, M.; Seeberger, P. H. Traceless Photolabile Linker Expedites the Chemical Synthesis of Complex Oligosaccharides by Automated Glycan Assembly. *J. Am. Chem. Soc.* **2019**, *141*, 9079–9086.

(4) Delbianco, M.; Kononov, A.; Poveda, A.; Yu, Y.; Diercks, T.; Jiménez-Barbero, J.; Seeberger, P. H. Well-Defined Oligo- and Polysaccharides as Ideal Probes for Structural Studies. *J. Am. Chem. Soc.* **2018**, *140*, 5421–5426.

(5) Fosso, M.; AlFindee, M. N.; Zhang, Q.; Nziko, V. D. P. N.; Kawasaki, Y.; Shrestha, S. K.; Bearss, J.; Gregory, R.; Takemoto, J. Y.; Chang, C. W. T. Structure-Activity Relationships for Antibacterial to Antifungal Conversion of Kanamycin to Amphiphilic Analogues. *J. Org. Chem.* **2015**, *80*, 4398–4411.

(6) Johannes, M.; Reindl, M.; Gerlitzki, B.; Schmitt, E.; Hoffmann-Röder, A. Synthesis and Biological Evaluation of a Novel MUC1 Glycopeptide Conjugate Vaccine Candidate Comprising a 4'-Deoxy-4'-Fluoro-Thomsen-Friedenreich Epitope. *Beilstein J. Org. Chem.* **2015**, *11*, 155–161.

(7) Yu, Y.; Tyrikos-Ergas, T.; Zhu, Y.; Fittolani, G.; Bordoni, V.; Singhal, A.; Fair, R. J.; Grafmüller, A.; Seeberger, P. H.; Delbianco, M. Systematic Hydrogen-Bond Manipulations To Establish Polysaccharide Structure–Property Correlations. *Angew. Chemie* **2019**, *131*, 13261–13266.

(8) Tyrikos-Ergas, T.; Bordoni, V.; Fittolani, G.; Chaube, M. A.; Grafmüller, A.; Seeberger, P. H.; Delbianco, M. Systematic Structural Characterization of Chitooligosaccharides Enabled by Automated Glycan Assembly. *Chem. - A Eur. J.* **2021**, *27*, 2321–2325.

(9) Kröck, L.; Esposito, D.; Castagner, B.; Wang, C. C.; Bindschädler, P.; Seeberger, P. H. Streamlined Access to Conjugation-Ready Glycans by Automated Synthesis. *Chem. Sci.* **2012**, *3*, 1617–1622.

(10) Joseph, A. A.; Pardo-Vargas, A.; Seeberger, P. H. Total Synthesis of Polysaccharides by Automated Glycan Assembly. *J. Am. Chem. Soc.* **2020**, *142* (19), 8561–8564.

(11) Guberman, M.; Bräutigam, M.; Seeberger, P. H. Automated Glycan Assembly of Lewis Type i and II Oligosaccharide Antigens. *Chem. Sci.* **2019**, *10*, 5634–5640.

(12) Hurevich, M.; Kandasamy, J.; Ponnappa, B. M.; Collot, M.; Kopetzki, D.; McQuade, D. T.; Seeberger, P. H. Continuous Photochemical Cleavage of Linkers for Solid-Phase Synthesis. *Org. Lett.* **2014**, *16*, 1794–1797.
